# Cross genome comparisons of serine proteases in Arabidopsis and rice

**DOI:** 10.1186/1471-2164-7-200

**Published:** 2006-08-09

**Authors:** Lokesh P Tripathi, R Sowdhamini

**Affiliations:** 1 National Centre for Biological Sciences, Tata Institute of Fundamental Research, GKVK Campus, Bellary Road, Bangalore 560 065, India

## Abstract

**Background:**

Serine proteases are one of the largest groups of proteolytic enzymes found across all kingdoms of life and are associated with several essential physiological pathways. The availability of *Arabidopsis thaliana *and rice (*Oryza sativa*) genome sequences has permitted the identification and comparison of the repertoire of serine protease-like proteins in the two plant species.

**Results:**

Despite the differences in genome sizes between *Arabidopsis *and rice, we identified a very similar number of serine protease-like proteins in the two plant species (206 and 222, respectively). Nearly 40% of the above sequences were identified as potential orthologues. Atypical members could be identified in the plant genomes for Deg, Clp, Lon, rhomboid proteases and species-specific members were observed for the highly populated subtilisin and serine carboxypeptidase families suggesting multiple lateral gene transfers. DegP proteases, prolyl oligopeptidases, Clp proteases and rhomboids share a significantly higher percentage orthology between the two genomes indicating substantial evolutionary divergence was set prior to speciation. Single domain architectures and paralogues for several putative subtilisins, serine carboxypeptidases and rhomboids suggest they may have been recruited for additional roles in secondary metabolism with spatial and temporal regulation. The analysis reveals some domain architectures unique to either or both of the plant species and some inactive proteases, like in rhomboids and Clp proteases, which could be involved in chaperone function.

**Conclusion:**

The systematic analysis of the serine protease-like proteins in the two plant species has provided some insight into the possible functional associations of previously uncharacterised serine protease-like proteins. Further investigation of these aspects may prove beneficial in our understanding of similar processes in commercially significant crop plant species.

## Background

The proper functioning of a cell is ensured by the precise regulation of protein levels that in turn are regulated by a balance between the rates of protein synthesis and degradation. Protein degradation is mediated by proteolysis, which paves the way for recycling of amino acids into the cellular pool. In addition to degradation, regulated proteolysis plays a major role in diverse cellular processes by regulating biochemical activities of proteins in concert with other cellular mechanisms such as post-translational modification[1,2]. Serine proteases are one of the largest groups of proteolytic enzymes involved in numerous regulatory processes. They catalyse the hydrolysis of specific peptide bonds in their substrates and this activity depends on a set of amino acids in the active site of the enzyme, one of which is always a serine. They include both exopeptidases that act on the termini of polypeptide chains and endopeptidases that act in the interior of polypeptide chains and belong to many different protein families that are grouped into clans[3,4]. A clan is defined as a group of families, the members of which are likely to have a common ancestor. Currently there are over 50 serine-protease families known as classified by MEROPS database[5], an information resource for peptidases and their inhibitors.

Serine proteases appear to be the largest class of proteases in plants[2]. Consistent with their regulatory role, plant serine proteases have been shown to be involved in diverse processes regulating plant development and defense responses[2,6-8]. In addition, some of plant serine carboxypeptidases (Family S10) function as acyltransferases rather than hydrolases, suggesting diversification of function to regulate secondary metabolism in plants [9-11]. However, the function and regulation of plant proteases is poorly understood primarily due to lack of identification of their physiological substrates. While there is extensive literature available on plant serine proteases, particularly in *Arabidopsis thaliana*, most of these studies are either restricted to specific families [11-13] or specific physiological aspects (please see the references above). *Arabidopsis thaliana *and *Oryza sativa *constitute model systems for Dicotyledons and Monocotyledons respectively, the major evolutionary lineages in angiosperms[14,15]. The availability of the complete genomic sequence of these two species renders it possible to carry out a comprehensive analysis of serine-protease families to gain insights into their function and to ascertain their evolutionary relationships.

The current analysis aims at the identification and analysis of serine protease families encoded in the genomes of the two plant species. The domain organization of the putative serine proteases in the two plant genomes have been analysed employing several sensitive sequence search methods [16-18] that have provided insights into different domain architectures. To understand the evolutionary relationships among the *Arabidopsis *and rice serine protease-like proteins, phylogenetic analysis was performed based at their protease domains. Cross genome comparison of the serine proteases across the two genomes has led to the identification of members likely to be conserved across the two species. Predictions of the sub-cellular localizations of serine protease-like proteins to obtain further insight into their possible functional associations were carried out using TargetP[19]. Comparison of plant serine proteases with other major eukaryotic lineages reveals selective expansion of serine protease families in plants and identification of plant specific serine proteases. This paper reports the first bioinformatics genome-wide survey of plant serine proteases and cross-comparisons of several serine protease families across monocots and dicots. Species-specific proteases and existence of evolutionary changes like lateral gene transfer and gene shuffling were observed at the catalytic domain. Further experimental characterization of *Arabidopsis *and rice serine proteases that are likely to be involved in basic functions associated with various physiological processes in plants would be useful in expanding the current understanding of these processes in regulating plant growth and development. This would prove beneficial in understanding protease processes in plants and application of this knowledge to important crop species.

## Results and discussion

Despite the differences in genome sizes between *Arabidopsis *and rice (125 Mb and 389 Mb respectively) and encoded number of genes (25,498 and 37,544 non-transposable-element-related-protein-coding[14,15] and 55,890 in total as in TIGR[20] rice database), the two plant species appear to encode a very similar number of serine protease-like proteins (206 and 222 putative numbers, respectively; Table 1; see additional files 1.2: Table S1.pdf, Table S2.pdf). These include several putative serine protease homologues that are apparently catalytically inactive, since they either lack the amino acid residues that are believed to be essential for catalysis or carry amino acid substitutions at positions corresponding to catalytically active sites. Such inactive-enzyme homologues are not restricted to serine proteases, but have been identified in diverse enzyme families, chiefly among proteins involved in signaling pathways and proteins that are apparently extracellular in function and are conserved across metazoan species. They are believed to have acquired newer and more distinct functions, in the course of evolution, thereby adding to the complexity of various regulatory networks in the cell[21]. We discuss below the occurrence, phylogenetic patterns of serine protease domains in the plant genomes by grouping according to MEROPS classification[5]. Only serine protease families with non-zero occurrence of putative members in the two genomes are discussed.

### DegP proteases (Family S1)

DegP proteases are a group of ATP-independent serine proteases that belong to MEROPS[5] family S1 (trypsin family). *E*.*Coli *DegP is a peripheral membrane protein located in the periplasmic side of the plasma membrane. It is a heat-shock protein that combines both chaperone and proteolytic activities that switch in a temperature-dependent manner. The chaperone activity is predominant at low temperatures, while the proteolytic activity takes over at higher temperatures[22]. Crystal structure of *E*.*coli *DegP reveals that the proteolytic site is inaccessible at low temperatures and thus, enzyme exists in chaperone conformation (See additional file 3: Table S3.pdf)[22]. PDZ domains are known to modulate the function and/or localization of their associated proteins and it has been shown that PDZ domains are involved in substrate recognition and binding in certain proteases [23-26].

DegP-like proteins have been identified in bacteria and eukaryotes but appear to be absent in archaea, and are believed to have spread to eukaryotic lineages via horizontal gene transfer events [27-29]. The exact functions of DegP-like proteins in plants remain unknown. A plant DegP homolog, DegP1, was first identified in *Arabidopsis *and was found to be strongly associated with the lumenal side of the thylakoid membrane. It was found to be rapidly upregulated in response to elevated temperatures and DegP homologue associated membrane fraction was found to have serine-type proteolytic activity, suggesting a possible role in chloroplast heat response[30,31]. More recently, a chloroplast DegP was shown to function in the initial cleavage of D1 protein of PSII (Photosystem II) subsequent to photoinhibition[32].

16 DegP-like proteins could be identified in *Arabidopsis *proteome, higher than an earlier estimate of 14[6]. Likewise, 15 DegP-like proteins were identified in rice. A majority of these are predicted to localise to either chloroplast or mitochondria (See additional file 2: Table S2.pdf). Of these, four gene products (At3g03380, At3g27925, At5g27660, At5g39830) from *Arabidopsis *and four (LOC_Os02g48180, LOC_Os04g38640, LOC_Os05g49380, LOC_0s11g14170) from rice are predicted to contain PDZ-like domains C-terminal to the protease domain, while the rest appear to be a single protease domain containing proteins. The absence of PDZ-like domains in majority of plant DegP-like proteins has been suggested to have as of yet unclear functional implications for these proteins[6]. We identified seven orthologous pairs of DegP-like proteins across the two genomes (See additional file 4: Table S4.pdf). *Arabidopsis *DegP1 (At3g27925) was found to be chloroplast localised and was rapidly upregulated in response to elevated temperatures[30]. We identified an At3g27925 orthologue in the rice proteome (LOC_Os05g49380) nearly identical in size that shares a high sequence similarity with At3g27925 and may function in a similar manner. The significant number of predicted DegP-like proteins in the two plant species suggests the presence of a conserved repertoire of proteins that are likely to be upregulated in response to elevated temperatures in thylakoid lumen and mitochondrial matrix in plants. Some putative gene products in both the genomes are likely to be proteolytically inactive due to either mutations or deletions in the active site residues (See additional file 5: Figure SF1.pdf). It remains unclear whether these proteolytically inactive members retain their chaperone activity at low temperatures, they may modulate the function of active gene products by regulating substrate association and complex formation.

Phylogenetic analysis of the protease domains of *Arabidopsis *and rice DegP-like proteins showed that a majority of sequences cluster into three major clades, supported by high bootstrap values, with additional members less related to the three clusters (See additional file 5: Figure SF1.pdf). Clade I, the largest of three clusters consists of 14 DegP-like proteins that are more varied in sequence and share between 35 and 95% pairwise amino acid sequence identity, Clade II consists of six closely related sequences that share between 50 and 93% pairwise sequence identity with each other. Clade II includes two sequence pairs At3g27925:LOC_Os05g49380 and At5g39830:LOC_Os04g38640 that carry a PDZ domain each C-terminal to the protease domain and share 93% and 87% pairwise sequence identity respectively. Clade III, the smallest of three clades, consists of five sequences that share between 51 and 94% pairwise sequence identity with each other. In addition, there are two pairs of PDZ domain containing proteins, At5g27660:LOC_Os11g14170 and At3g03380:LOC_Os02g48180, that share 58 and 50% pairwise sequence identity respectively.

### Subtilisins (Family S8)

The subtilisin family is the second largest family of serine proteases identified till date with over 200 known members across lineages in eubacteria, archaebacteria, eukaryotes and viruses. Crystal structures reveal that subtilisins utilize a highly conserved catalytic triad similar to the members of chymotrypsin and carboxypeptidase clans but have a different order of Asp, His and Ser residues in the sequence (D137, H168, S325) with no other structural similarity[33] posing a case of convergent evolution. MEROPS database[5] subdivides the subtilisin family into two subfamilies: S8A (subtilisin subfamily) consisting of true subtilisins and S8B (kexin subfamily) consisting of proprotein-processing enzymes. An interesting feature of the subtilisin family is that some members appear to be mosaic with little or no sequence similarity to any other known proteins[33] and with large N- and C-terminal extensions.

A large number of subtilisin-like serine proteases have been identified in various plant species, where they have been implicated in diverse processes (See additional file 3: Table S3.pdf) and all appear to correspond to S8A subfamily, while kexin-type proteins appear to be absent from plants[2,12,34,35]. It has been reported that both *Arabidopsis *and rice are characterised by significantly larger number of subtilases in comparison to other organisms such as human, *Caenorahabditis *and *Drosophila*, suggesting that expansion of the subtilase gene family in the two plant species is due to multiple duplication events[35].

We have identified 56 subtilisin-like proteins in *Arabidopsis *proteome, similar to the figures reported elsewhere[12,35] and 63 in rice. A majority of these gene products, as expected, are predicted to be secretory pathway proteins, though a few are predicted to localise to chloroplasts and mitochondria (See additional files 1, 2: Table S1.pdf, Table S2.pdf). The analysis of the domain architectures of *Arabidopsis *and rice subtilisin-like proteins reveals that most of these gene products consist of three domains: an N-terminal Subtilisin_N domain that is removed prior to activation of the enzyme, Peptidase_S8 domain and the protease associated (PA) domain, that occurs as a C-terminal insertion in the Peptidase_S8 domain of a majority of subtilisin-like proteins identified in these two plant species (See additional files 1, 2: Table S1.pdf, Table S2.pdf). The PA domain is believed to be involved in protein-protein interactions or mediate substrate recognition by peptidases and is believed to have been associated with ancestral subtilases[35,36]. In addition, most subtilisin-like proteins identified in the two plant species, carry short insertions ranging from 15–45 amino acid residues in length in the N-terminal region of their predicted protease domains. These insertions do not share any obvious sequence similarities with any other known module and are likely to be unique in their association with *Arabidopsis *and rice subtilisin-like proteins (data not shown). However, the presence of gene products in both the proteomes with only the protease domain suggests that this domain alone is sufficient for the activity of these enzymes (See additional files 1, 2: Table S1.pdf, Table S2.pdf). The *Arabidopsis *proteome contains two gene products At2g19170 and At4g30020 that appear to possess an additional module, classified as DUF1034 (Pfam[37] accession: PF06280), C-terminal to the PA domain, while a single similar gene product LOC_Os06g48650 was identified in rice proteome, suggesting a duplication of this gene in *Arabidopsis *subsequent to dicot-monocot divergence (Figure 2; see additional files 1, 2: Table S1.pdf, Table S2.pdf). The lack of diverse domain architecture in plant subtilisin-like proteins, suggests that specific expansion of subtilisin gene family in the two plant species has been facilitated by extensive gene duplication. This is further supported by the observation that only 16 orthologous pairs were identified across the two species, suggesting that the majority of subtilisin-like proteins in the two species arose after dicot-monocot divergence, possible due to gene duplication (See additional file 4: Table S4.pdf). It is likely that spatial and temporal regulation of gene expression patterns may play a major role in regulating the activity of subtilisin-like proteins in plants. The presence of a large number of subtilisin-like proteins in the two plant species suggests possible functional redundancy, while on the other hand it may be indicative of functional diversification. A significant number of plant subtilisins may have been recruited for functions in plant secondary metabolism, in a manner similar to some serine carboxypeptidase-like proteins. The subtilisin-like proteins identified here in *Arabidopsis *and rice as well as those reported from other plant species show maximum similarity to MEROPS[5] S8A subfamily of subtilisins. A few gene products are likely to be proteolytically inactive due to mutations in the catalytic residues and are most likely to be evolutionary intermediates (See additional file 6: Figure SF2.pdf).

**Figure 1:**
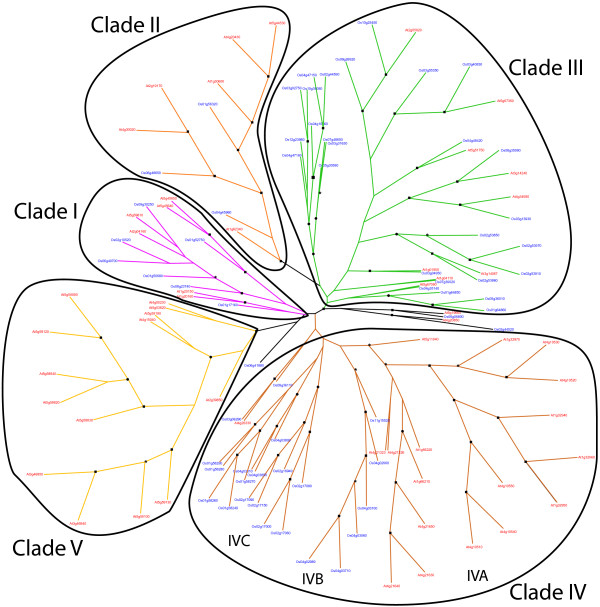
Unrooted N-J tree computed from multiple sequence alignments of *Arabidopsis *(red) and rice (blue) subtilisin domains. Subtilisin-like protease domains were aligned using ClustalW [95] program and the alignments were exported to Phylip package [96] for representing the Neighbor-Joining tree (see methods). The colors and circles represent different evolutionary clades identified in the analysis (see text for details). Clade I is represented in purple, Clade II is shaded orange, Clade III in green, Clade IV in brown and Clade V in yellow. For clarity, bootstrap values were replaced with symbols representing bootstrap percentages >50%. Bootstrap values between 50–60% are represented by an asterix, circles represent bootstrap values from 60%–80% while bootstrap values >80% are represented by rectangles. Gene names correspond to those in Additional files 1 and 2. For brevity, rice gene names have been shortened to OsXXg##### instead of LOC_OsXXg#####, XX referring to chromosome 1–12 and a 5 digit number assigned to each gene. A few species specific gene clusters were also identified in the analysis (see text for details).

Phylogenetic analysis of *Arabidopsis *and rice subtilisin-like proteins was carried out with the S8 protease domain minus the insertions. The analysis reveals the presence of five major gene clusters of which four consist of subtilisin-like proteins from both *Arabidopsis *and rice including orthologous pairs (Figure 1). Clade I consists of 13 sequences that share between 44 and 76% pairwise sequence identity with each other. Clade II, the smallest of five clusters consists of six sequences from *Arabidopsis *and three from rice that share between 48 and 84% sequence identity with each other. Clade III consists of 31 sequences from both the plant species that share between 40 and 80% sequence identity with each other. The members of Clade III may be grouped into two sub-clusters. Clade IV appears to be the largest and most diverse cluster of *Arabidopsis *and rice subtilisin-like proteins. It includes 40 sequences that share between 35 and 92% pairwise percentage identity with each other. On the basis of pairwise percentage identities between the constituent sequences, Clade IV may be further subdivided into three subclusters: Clade IVA, IVB and IVC. Clade IVA consists exclusively of *Arabidopsis *sequences that share between 47 and 91% sequence identity with each other and between 35 and 60% sequence identity with the rest of the Clade IV sequences. In contrast, Clade IVB and Clade IVC, consist exclusively of rice subtilisin-like proteins. Clade IVB the smallest of three subclusters, consists of sequences that share between 45 and 81% sequence identity with each other, while sharing between 35 and 60% sequence identity with the remaining members of Clade IV. Clade IVC sequences share between 54 and 92% sequence identity with each other and between 44 and 61% sequence identity with the rest of the constituents of Clade IV. Clade V is an exclusive cluster of *Arabidopsis *subtilisin-like proteins that are between 41 and 85% identical with each other and most likely represent an *Arabidopsis *specific subfamily of subtilisin-like proteins. Our observations appear consistent with a recent report that identifies the presence of four major clusters of orthologous groups and a fifth cluster of *Arabidopsis *sequences within the phylogenetic tree constructed with *Arabidopsis *and rice sutbilisin-like proteins[35]. We identified a few gene sub-clusters that consist exclusively of *Arabidopsis *or rice subtilisin-like gene products indicating the existence of *Arabidopsis *or rice subtilisin-like genes and by extension putative dicot- and monocot-specific subtilisin-like genes (Figure 1).

### Prolyl oligopeptidases (Family S9)

The members of Prolyl oligopeptidases (POPs) family of serine peptidases belong to the α/β hydrolase fold grouped into SC clan of serine peptidases. The family includes members of different types and with distinct specificities. For example, prolyl oligopeptidase is an endopeptidase localised to the cytosol, while dipeptidyl peptidase IV (DPPIV) is a membrane bound exopeptidase. According to MEROPS database[5], POPs may be classified into four subfamilies with prolyl oligopeptidase (S9A), dipeptidyl-peptidase IV (S9B), aminoacyl-peptidase (S9C) and glutamyl endopeptidase (S9D) being the typical examples of their respective subfamilies.

POPs have been implicated in degradation of biologically important peptides such as peptide hormones and neuropeptides associated with learning and memory and therefore have become significant targets for drug design[38]. They have been identified in organisms from all forms of life, though members of this family display differences in mutation rates and appear to have undergone several changes in their localization over the course of evolution[39,40].

We identified 23 genes encoding prolyl oligopeptidase-like proteins in *Arabidopsis *and 23 in rice. A majority of these either lack a predicted signal sequence or are predicted to localise to chloroplast and mitochondria. Only few gene products identified here are predicted to carry a β-propeller N-terminal to the protease domain (See additional files 1, 2: Table S1.pdf, Table S2.pdf). The absence of the N-terminal domain in a majority of plant POP-like proteins is intriguing since it is likely to have a functional implication for these proteins. Following a careful inspection of the amino acid residues in the vicinity of the active site serine residue, we were able to unambiguously assign a few POP-like proteins in the two plant species to different subfamilies of POP-like proteins as classified by MEROPS[5]. These include At1g76140, LOC_Os01g01830 (GG**S**NGGLL; S9A); At5g24260 (GW**S**YGGY; S9B); At2g47390, LOC_Os03g19410, LOC_Os07g48970 (GGH**S**YGAFMT; S9D) (See additional file 7: Figure SF3.pdf). We identified 14 orthologous pairs of POP-like proteins across the two plant species, suggesting high degree of conservation in the function of POP-like proteins across the two species (See additional file 4: Table S4.pdf). It also suggests that majority of these enzymes were present prior to dicot-monocot divergence, consistent with the ancient evolutionary origin of these proteins. Phylogenetic analysis, however, reveals presence of several gene clusters of *Arabidopsis *and rice POP-like proteins that share low pairwise sequence identity with each other and significant bootstrap values (Figure 3).The largest of these clusters Clade I consists of 12 POP-like gene products from the two plant species that are closely related and the pairwise percent sequence identity between any two sequences within the cluster ranges from 60 to 96%. The second major cluster Clade II is more varied in composition and consists of 10 sequences that may further be grouped into two subclusters Clade IIA and Clade IIB based or pairwise percent sequence identity between any two sequences falling into the cluster. Clade IIA includes four sequences (At1g20380, At1g76140, LOC_Os01g01830 and LOC_Os04g47360) that are between 72 and 89% identical with each other. Clade IIB includes six sequences that share between 32 and 85% pairwise sequence identity with each other. The members of two subclusters share between 22 and 32% pairwise sequence identity with each other. Clade IIA includes two sequences (At1g76140, LOC_Os01g01830) identified as members of the POP (S9A) subfamily of Prolyl oligopeptidase-like proteins (see above) suggesting that the other members of this subcluster may possibly belong to the S9A subfamily (Figure 3). No species-specific clusters were identified in this family.

### Serine carboxypeptidases (Family S10)

Serine carboxypeptidases catalyze the hydrolysis of the C-terminal bond in proteins and peptides. Crystal structures show that serine carboxypeptidases belong to the α/β hydrolase fold and possess a catalytic triad similar to members of chymotrypsin and subtilisin families in the order Ser, Asp and His (S257, D449, H508)[5].

Serine carboxypeptidase-like proteins (SCPLs) have been identified in several plant species, where they form large and diverse gene families in contrast with prokaryotes and other eukaryotes. Plant SCPLs have been implicated in several biochemical processes including secondary metabolism (See additional file 3: Table S3.pdf) and are believed to be essential to plant growth and development[2,9-11], [41-43].

**Figure 2:**
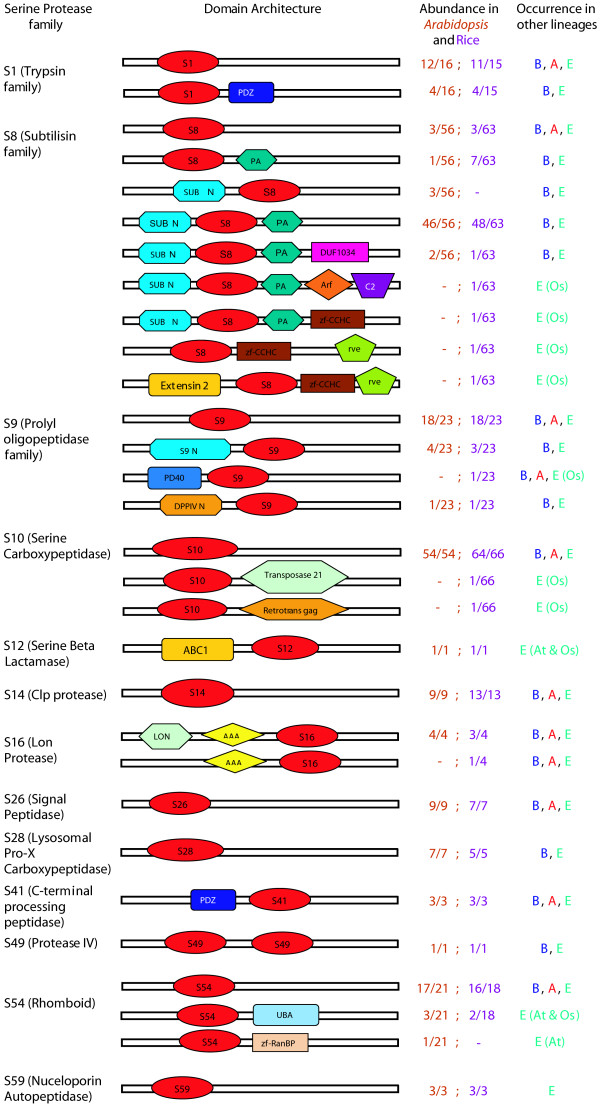
Domain Architectures identified in *Arabidopsis *and rice serine Protease-like proteins. At -*Arabidopsis thaliana*; Os – Rice (*Oryza sativa*); B- Bacteria; A- Archaea; E- Eukaryota; Sxx- Serine protease family Sxx domain, where Sxx refers to the serine protease family as per MEROPS [5] classification (see text for details). PDZ- PDZ domain (Pfam [37] accession: PF00595); PA- Protease associated domain (Pfam [37] accession: PF02225); SUB N- Subtilisin N-terminal region (Pfam [37] accession: PF005922); DUF1034- Domain of unknown function (Pfam [37] accession: PF06280); Arf- ADP-ribosylation factor family (Pfam [37] accession: PF00025); C2- C2 domain (Pfam [37] accession: PF00168); zf-CCHC- Zinc knuckle (Pfam [37] accession: PF00098); rve- Integrase core domain (Pfam accession:PF00665); Extensin 2- Extensin-like region (Pfam [37] accession: PF04554); S9 N- Prolyl oligopeptidase, N-terminal beta-propeller domain (Pfam [37] accession: PF02897); PD40- WD40-like beta propeller repeat (Pfam [37] accession: PF07676); DPPIV N- Dipeptidyl peptidase (DPP IV) N-terminal region (Pfam [37] accession: PF00930); Transposase 21- Transposase family tnp2 (Pfam [37] accession: PF02992); Retrotrans gag- Retrotransposon gag protein (Pfam [37] accession: PF03732); ABC1- ABC1 family (Pfam [37] accession: PF03109); LON- ATP-dependent protease La (LON) domain (Pfam [37] accession: PF02190); AAA- ATPase family associated with various cellular activities (Pfam [37] accession: PF00004); UBA- UBA/TN-S (ubiquitin associated) domain (Pfam [37] accession: PF000627); zf-RanBP- Zinc finger in Ran binding protein and others (Pfam [37] accession: PF00641).

**Figure 3:**
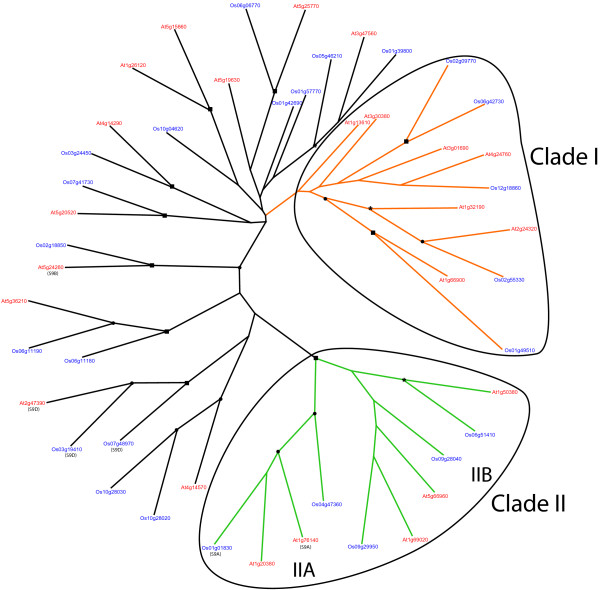
Unrooted N-J tree computed from multiple sequence alignments of *Arabidopsis *(red) and rice (blue) prolyl oligopeptidase domains. Prolyl oligopeptidase-like domains were aligned using ClustalW [95] program and the alignments were exported to Phylip package [96] for representing the Neighbor-Joining tree (see methods). The colors and circles represent the two evolutionary clades identified in the analysis (see text for details). Clade I is represented in Orange, Clade II is shaded green. For clarity, bootstrap values were replaced with symbols representing bootstrap percentages >50%. Bootstrap values between 50–60% are represented by an asterix, circles represent bootstrap values from 60%–80% while bootstrap values >80% are represented by rectangles. Gene names correspond to those in Additional files 1 and 2. For brevity, rice gene names have been shortened to OsXXg##### instead of LOC_OsXXg#####, XX referring to chromosome 1–12 and a 5 digit number assigned to each gene. Subfamily assignments where possible are indicated in parentheses below the gene name (see Figure SF3 and text for details).

We identified 54 genes coding for serine-carboxypeptidase (SCP)-like proteins in *Arabidopsis *and 66 in rice. A majority of these gene products are predicted to be secretory pathway proteins, though a number of rice SCP-like proteins are either predicted to localise to mitochondria and chloroplasts or lack a predictable signal sequence (See additional files 1, 2: Table S1.pdf, Table S2.pdf). The analysis of domain architectures of *Arabidopsis *and rice SCP-like proteins reveals that nearly all gene products identified here are single domain proteins containing a single protease domain, with the exception of LOC_Os01g22970 that carries a single zinc binding module (zf-CCHC; Pfam[37] accession: PF00098) N-terminal to the protease domain and LOC_Os06g32740 that carries a conserved transposable element module (Transposase_21; Pfam[37] accession: PF01359) C-terminal to the protease domain (Figure 2). This clearly indicates that the predicted serine carboxypeptidase domain is usually sufficient for biological activity of the majority of gene products identified in the two plant species. The lack of diverse domain architecture suggests that spatial and temporal regulation of gene expression patterns may play a major role in regulating the activity of SCP-like proteins in *Arabidopsis *and rice. We identified 17 orthologous pairs across the two species suggesting, as in the case of subtilisin-like proteins, a majority of SCP-like proteins in *Arabidopsis *and rice probably arose due to extensive gene duplication subsequent to dicot-monocot divergence and that has contributed to their forming a large gene family in the two species (Tables 2, 3; see additionals files 4, 8: Table S4.pdf, Figure SF4.pdf). The presence of a large number of SCP-like proteins in the two plant species suggests a possible functional redundancy, while on the other hand it may indicate of functional diversification, where a significant number of these proteins may have been recruited for functions in plant secondary metabolism to catalyze novel reactions (See additional file 3: Table S3.pdf). SCP-like enzymes have been shown to function as acyltransferases in several plant species including *Arabidopsis*. *Arabidopsis *SCP-like proteins SCT/SNG2 (At2g22920) and SMT/SNG1 (At5g09640) have been shown to function as acyltransferases in phenylpropanoid metabolism[41,43]. Studies have shown that *Arabidopsis *SMT is a heavily glycosylated protein that localises to central vacoules of the leaf tissue, suggesting that some SCP-like proteins identified in the two plant species may also be vacoule localised[44].

Phylogenetic analysis indicates that the majority of *Arabidopsis *and rice SCPL proteins cluster into two major clades, but there are some additional SCPL proteins that are less closely related to members of the clades (See additional file 8: Figure SF4.pdf). Clade I may further be subdivided into three subgroups, Clade IA (includes only *Arabidopsis *SCPL proteins), Clade IB (includes only rice SCPL proteins) and Clade IC (includes a pair of *Arabidopsis *and rice SCPL proteins). Clade IA includes known SCPL acyltransferases such as SMT (At2g22990) and SCT (At5g09640). Clade IA and IB consist exclusively of *Arabidopsis *and rice sequences respectively that have no identifiable orthologues in either subcluster. Therefore these two subclades may represent early diversification of certain SCPL proteins followed by their lineage specific delineation and expansion subsequent to speciation (See additional file 8: Figure SF4.pdf).

Clade II, the larger of two major clades, consists of sequences that share between 33 and 82% pairwise sequence identity with each other. Earlier observations have suggested that Clade II consists of more varied sequences in terms of pairwise sequence identity, conservation of key residues and exon/intron splice positions[45]. In contrast to Clade I, *Arabidopsis *and rice sequences falling into Clade II, are interspersed with each other with the exception of a small number of rice sequences that form an exclusive subcluster. The members of the two clades mostly share between 20 and 30% pairwise sequence identity. However, of the three subclusters identified among Clade I proteins, the members of Clade IC appear to be closer to Clade II sequences than members of Clade IA and Clade IB and share between 25 and 35% pairwise sequence identity with the members of Clade II.

### Serine beta-lactamases (Family S12)

Serine beta-lactamases are a group of hydrolases that catalyze the hydrolysis of the beta-lactam ring of β-lactam antibiotics such as penicillins. They were first identified in penicillin-resistant bacterial isolates and have been found to be widely distributed in eubacterial genomes. On the basis of sequence similarity, they have been classified into three classes A, C and D, where class B consists of metallo-beta-lactamases. Class A (penicillinase type) proteins were the first to be identified and are the most common beta-lactamases. All these proteins share sequence similarity within the class but not with members of other classes. However, proteins of different classes share significant structural similarity and are believed to be homologues that have undergone divergent evolution. Structure-based phylogeny studies suggest that beta-lactamases are ancient enzymes that probably evolved as means of protection against beta-lactam antibiotics[46]. Site-directed mutagenesis and structural analyses have helped to unravel the catalytic mechanism of β-lactamases and led to the identification of their active site residues (S93, K96, Y190)[47,48].

Serine beta-lactamases are widespread in bacterial genomes, and their corresponding genes may occur on bacterial chromosomes or on plasmids. This allows for their transfer to distant species and may account for their distribution and diversity[48]. Serine beta-lactamase homologues have been identified in eukaryotes, mainly as a consequence of ongoing genome sequencing programs. However, little function information is available about these proteins in eukaryotes.

We have identified a single serine beta-lactamase like gene product each in *Arabidopsis *and rice proteomes, both of which are predicted to localise to mitochondria. An interesting feature is the presence of a single ABC1 domain (Pfam[37] accession: PF03109) N-terminal to the serine β-lactamase domain (Figure 2; see additional files 1, 2: Table S1.pdf, Table S2.pdf). ABC1 domains are associated with a family of proteins that include members from yeast and bacteria that display a nuclear or mitochondrial subcellular location in eukaryotes. Some members of the family are believed to regulate mRNA translation and are essential for electron transfer in the bc1 complex[37] (See additional file 9: Figure SF5.pdf).

### Clp ATP-dependent proteases (Family S14)

Clp proteases are a group of ATP-dependent serine endopeptidases that belong to MEROPS[5] family S14. *E*.*coli *ClpP is an ATP-dependent serine protease that consists of a smaller protease subunit ClpP, and a larger chaperone regulatory ATPase subunit (either ClpA or ClpX). Though the protease domain is capable of proteolysis on its own, the ATPase subunits are essential for effective levels of proteolysis. Structural analysis (See additional file 3: Table S3.pdf) reveals that the catalytic triad residues Ser-His-Asp (S111, H136, D185) are enclosed in a single cavity that allows for degradation of small peptides but precludes the entry of large folded polypeptides (See additional file 3: Table S3.pdf)[6,7,49].

Clp proteases have been identified in bacteria and eukaryotes but appear to be absent from archaea. They have been implicated in protein quality control and degradation of misfolded nascent peptides[50]. Plant Clp proteases were first identified with the discovery of the plastid-encoded gene clpP1, orthologous to *E*.*coli *clpP and in most higher plants it is transcribed by genes rps12 and rps120[6,7]. Though the exact function for Clp proteases remains to be worked out, they appear to be essential for chloroplast function and viability. Clp proteases are likely to play a role in protein degradation in stroma and thylakoid membrane under particular stress conditions thereby ensuring removal of aberrant polypeptides and recycling of the nutrient pool[7,13]. They have also been implicated in shoot development[51] suggesting a broad regulatory role for Clp proteases in plants.

**Figure 4:**
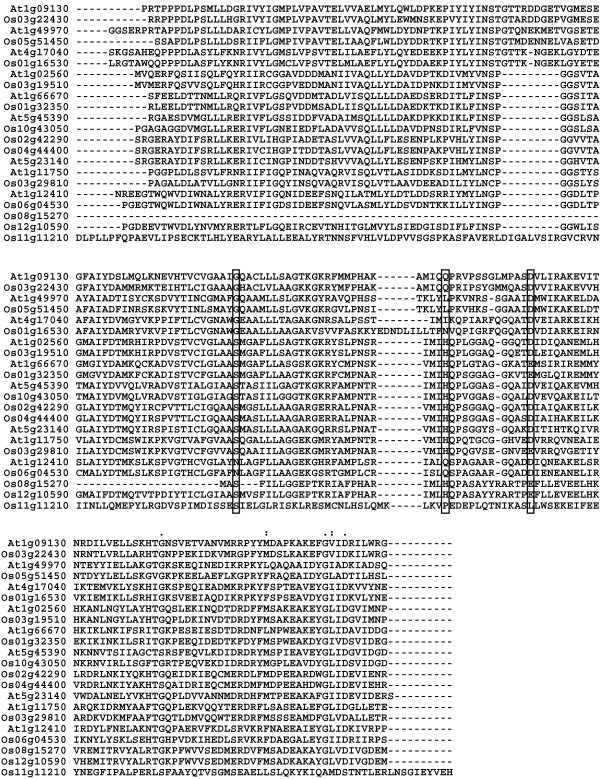
Multiple sequence alignment of the Clp protease domain region of the annotated *Arabidopsis *and rice Clp protease-like proteins. The catalytic triad residues are indicated. Gene names correspond to those in Additional files 1 and 2. For brevity, rice gene names have been shortened to OsXXg##### instead of LOC_OsXXg#####, XX referring to chromosome 1–12 and a 5 digit number assigned to each gene. Several gene products that display mutation in one or more catalytic triad residues can be visualised here (see text for details).

We have identified nine different ClpP genes encoding the proteolytic subunit of the Clp protease in the *Arabidopsis *nuclear proteome and 13 in rice nuclear proteome. Eight of the nine *Arabidopsis *ClpP-like proteins are predicted to localise to chloroplast, while At5g23140 gene product is predicted to be mitochondria localised, though experimental evidence suggests that At5g23140 gene product appears to localise to both chloroplasts and mitochondria[52]. Likewise, in rice, 11 ClpP-like proteins are predicted to be chloroplast localised, while LOC_Os08g15270 gene product is predicted to localise to mitochondria, while subcellular localization for LOC_Os12g1059 could not be determined (See additional files 1, 2: Table S1.pdf, Table S2.pdf). We observed that some ClpP gene products identified in the two species have lost the catalytic protease site and therefore do not appear to take part in proteolysis. However, they are believed to play a significant role in proper assembly of functional ClpP protein complex and potentially influence chaperone interaction (Figure 4). Interestingly, we identified orthologues for all nine *Arabidopsis *ClpP gene products in rice, suggesting high degree of evolutionary conservation of Clp function in the two species (See additional file 4: Table S4.pdf). Recently, a 325-kDa complex consisting of 10 Clp isoforms including the chloroplast encoded pClpP and nuclear encoded Clp proteins was identified in *Arabidopsis *chloroplasts and a 320-kDa homotetradecameric complex consisting of only ClpP2 gene products was identified in mitochondria. These protein complexes are believed to be involved in degradation of misfolded or unassembled peptides in an ATP-dependent manner[52]. High evolutionary conservation of ClpP-like proteins across the two species suggests that some rice ClpP homologues may participate in the assembly of a similar regulatory complex in rice chloroplasts and mitochondria.

**Figure 5:**
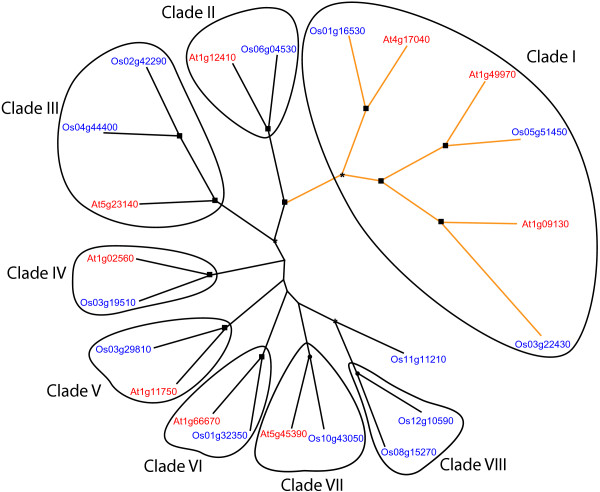
Unrooted N-J tree computed from multiple sequence alignments of *Arabidopsis *(red) and rice (blue) Clp protease domains. Clp protease-like domains were aligned using ClustalW [95] program and the alignments were exported to Phylip package [96] for representing the Neighbor-Joining tree (see methods). The colors and circles represent different evolutionary clades identified in the analysis (see text for details). Clade I is represented in orange, while clades II-VIII are shaded in black. For clarity, bootstrap values were replaced with symbols representing bootstrap percentages >50%. Bootstrap values between 50–60% are represented by an asterix, circles represent bootstrap values from 60%–80% while bootstrap values >80% are represented by rectangles. Gene names correspond to those in Additional files 1 and 2. For brevity, rice gene names have been shortened to OsXXg##### instead of LOC_OsXXg#####, XX referring to chromosome 1–12 and a 5 digit number assigned to each gene.

Phylogenetic analysis of *Arabidopsis *and rice Clp-like proteins reveals the presence of several gene clusters (Clades I-VIII) that share comparatively low pairwise sequence identity with each other, as opposed to the members within a cluster that share significant sequence similarity with each other (Figure 5). We identified eight Clp-like proteins from both *Arabidopsis *and rice that are likely to have lost their catalytic protease activity due to mutations of key residues in protease active site (Figure 4). Six of these gene products (At1g09130, At1g49970, At4g17040 from *Arabidopsis *and LOC_Os01g16530, LOC_Os03g22430, LOC_Os05g51450 from rice) fall into a single gene cluster (Clade I) and share between 36 and 72% pairwise sequence identity with each other (Figure 5). The above sequences have a 9–10 amino acid insert (L1 inserts) that are believed to affect the presentation of the substrate to the neighboring catalytic triads in ClpP proteins and also known to contribute residues to putative lateral openings likely to stimulate or inhibit exit of peptide fragments from the core[52] (Figure 4). The other ClpP-like gene products identified in the two species mostly cluster into independent gene clusters, Clades II-VIII (Figure 5). The sequences constituting the gene clusters share high pairwise sequence identity with each other, but are between 25 and 45% identical to the rest of ClpP-like proteases identified across the two species.

### Lon proteases (Family S16)

Lon proteases are also a group of ATP-dependent serine proteases. Unlike the Clp proteases, the catalytic and ATPase domains of the Lon proteases reside in the same polypeptide. They are conserved in prokaryotes and eukaryotic organelles such as chloroplast and mitochondria. *E*.*coli *Lon protease was the first ATP-dependent protease to be described and consists of three functional domains: the N-terminal domain (LON), a central ATPase domain (AAA+ module) and a C-terminal proteolytic domain (Lon_C). The N-terminal LON domain along with the AAA module is believed to impart substrate specificity to Lon proteases[6,53]. Structure and sequence analysis along with site-directed mutagenesis indicate that Lon proteases employ a Ser-Lys (S679, K/R722) catalytic dyad instead of a canonical serine protease catalytic triad[53]. Based on the conservation of residues around the catalytic residues, the Lon proteases have recently been proposed to be divided into two subfamilies: LonA and LonB[53].

Unlike Clp proteases, Lon proteases appear to be present in organisms from all forms of life. They are believed to play a significant role in removal of aberrant proteins and thereby maintain cellular stability. Interestingly, an archaeal membrane bound Lon protease was found to have both ATP-dependent and ATP-independent proteolytic activities towards folded and unfolded proteins respectively[54]. Little is known of their function in plants, but Lon1 from *Arabidopsis *has been implicated in the control of cytoplasmic male sterility[55].

We have identified four protein sequences containing the Lon proteolytic domain in *Arabidopsis *and an equal number in rice. In addition, we also identified five protein sequences containing only the Lon N-terminal domain in *Arabidopsis *and four in rice (data not shown). Of the *Arabidopsis *Lon protease-like proteins, At3g05790 and At5g26860 gene products are predicted to localise to chloroplast, while At3g05780 and At5g47040 gene products are predicted to be mitochondria localised. All four Lon protease-like proteins identified in rice are predicted to localise to chloroplasts. The Lon-protease like proteins identified in the two plant species share higher similarities with the LonA subfamily (See additional file 10: Figure SF6.pdf). The presence of LON-only sequences has been known in several prokaryotic species and its presence in *Arabidopsis *and rice proteomes suggests that LON domain may have been recruited as a general module for regulating protein-protein interactions, particularly for targeted protein degradation[56].

Sequence analysis reveals that *Arabidopsis *and rice Lon protease like sequences fall into two clusters on the basis of pairwise sequence identity (data not shown). The largest of these clusters consists of five sequences (At3g05790, At5g26860, At3g05780, LOC_Os03g19350, LOC_Os07g48960), all of which, except At3g05780 are predicted to localise to chloroplasts and share between 76 and 91% pairwise sequence identity with each other. The second cluster consists of two sequences (At5g47040 and LOC_Os09g36300) that are 82% identical to each other but predicted to have different subcellular localizations. At5g47040 is predicted to localise to mitochondria and LOC_Os09g36300 is predicted to localise to chloroplasts. The members of the two clusters display between 40 and 45% pairwise sequence identity with each other. A Lon protease like gene product from rice, LOC_Os06g05820, is less closely related and shares between 19 and 25% pairwise sequence identity with the rest of the Lon protease like gene products from the two species.

### Signal peptidases (Family S26)

Signal peptidases (SPases or leader peptidases) are a diverse group of serine endopeptidases, that belong to MEROPS[5] family S26 and are responsible for the removal of signal peptides from preproteins within the cell[57]. Signal peptides comprise the N-terminal part of the polypeptide chain. They are essential for targeting various proteins to their correct destination and are cleaved off subsequent to the transfer of the protein across the membrane. The failure to remove signal peptides often leads to protein inactivation and/or mislocalisation[57].

The Type I SPases are membrane-bound serine endopeptidases that were first identified in bacteria (*E*.*coli *lepB) and homologues have subsequently been identified in archaea, mitochondrial inner membrane, the thylakoid membrane of chloroplasts and the endoplasmic reticulum membranes of yeast and higher eukaryotes[57,58]. The Type I SPase family is further divided into two subfamilies: members of S26A subfamily employ a serine-lysine dyad for catalysis (S91, K146) and include prokaryotic, chloroplast and mitochondrial peptidases, while the members of S26B subfamily that include ER SPC and archaeal peptidases, employ a serine-histidine dyad (S44, H83) based on the mechanism for catalysis[57,58].

In bacteria, Type I SPases appear to be essential for cell viability and their inactivation invariably leads to cell death. Therefore they have been attractive targets for antibacterial drugs[57,58]. In higher plants, homologues of Type I SPases, known as thylakoidal processing peptidases have been identified and are involved in processing of protein presequences required for integration into or translocation across thylakoid membranes. TPP, an *Arabidopsis *homologue of *E*.*coli *LepB is a thylakoid membrane protein with its active site situated at the lumenal side[59,60]. An *Arabidopsis *plastidic SPase I (Plsp1, At24590) has been found to be required for complete maturation of the plastid protein translocation channel Toc75. It is proposed that Plsp1 may have multiple functions and is believed to be essential for biogenesis of plastid internal membranes[61].

**Figure 6:**
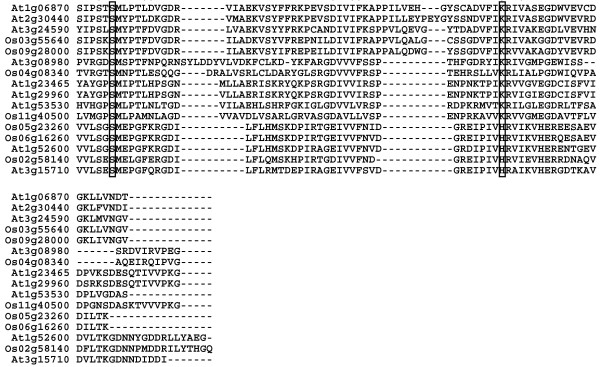
Multiple sequence alignment of the Type I Spase domain region of the annotated *Arabidopsis *and rice Type I Spase-like proteins. The catalytic dyad residues are indicated. Gene names correspond to those in Additional files 1 and 2. For brevity, rice gene names have been shortened to OsXXg##### instead of LOC_OsXXg#####, XX referring to chromosome 1–12 and a 5 digit number assigned to each gene. The variations in the second residue (K/H) of catalytic dyad can be identified here (see text for details).

We have identified nine genes coding for signal peptidase-like proteins in *Arabidopsis *and seven in rice. A majority of these gene products are predicted to localise to mitochondria, though some are predicted to be chloroplast localised, membrane bound and even secreted (See additional files 1, 2: Table S1.pdf, Table S2.pdf). *Arabidopsis *TPP (At2g30440), though predicted by TargetP[19] to be mitochondria localised, has been experimentally determined to be associated with chloroplast fraction. The enzyme when overexpressed in vitro, was found to possess catalytic activity against the lumenal targeting presequence of the 23-kDa component of photosystem II[59], suggesting a possible role for TPP and similar proteins in processing of polypeptides during their transport through cellular membranes. Most of the gene products analysed here possess Ser and Lys as active site residues and are likely to employ a serine-lysine catalytic dyad for proteolysis. Some gene products (At1g52600, At3g15710, LOC_Os05g23260, LOC_Os06g16260, LOC_Os02g58140) appear to possess Ser and His as active site residues and consequently may employ a serine-histidine catalytic dyad for proteolysis (Figure 6). We identified five orthologous pairs of signal peptidase-like proteins across the two species, suggesting conservation of signal-peptidase function in the two species (See additional file 4: Table S4.pdf).

Phylogenetic analysis indicates that *Arabidopsis *and rice Type I SPase-like proteins cluster into two major clades supported by high bootstrap values and high pairwise sequence identity (See additional file 11: Figure SF7.pdf). Clade I consists of 5 sequences (At1g06870, At2g30440, At3g24590, LOC_Os09g28000, LOC_Os03g55640) that are between 67 and 86% identical to each other. Clade II consists of 5 sequences (At1g52600, At3g15710, LOC_Os05g23260, LOC_Os06g16260, LOC_Os02g58140), that share between 67 and 96% pairwise sequence identity with each other. The members of either cluster share low sequence identity with the rest of the gene products. Interestingly, all the members of clade II carry histidine as an active site residue instead of Lysine and consequently are believed to employ a Ser-His dyad for proteolysis and possibly correspond to S26B subfamily of Type I Spases (Figure 6; see above; see additional file 11: Figure SF7.pdf). The remaining sequences carry lysine as an active site residue and are consequently believed to employ a Ser-Lys dyad for proteolysis likely to correspond to S26A subfamily of Type I Spases (Figure 6; see above; see additional file 11: Figure SF7.pdf). Clade I sequences share between 8 and 10% pairwise sequence identity with members of Clade II but share between 21 and 43% pairwise sequence identity with the remaining Type I SPase-like gene products. Clade II members appear to be less closely related and share between 8 and 15% pairwise sequence identity with the rest of Type I SPase-like gene products. Taken together these observations suggest that functional clusters of Type I SPases employing slightly varied catalytic mechanism were established early during evolution and were subsequently retained, possibly for adaptation to different physiological processes.

### Lysosomal Pro-X carboxypeptidase family (Family S28)

This family includes several eukaryotic enzymes such as lysosomal Pro-X carboxypeptidase (PCP), dipeptidyl-peptidase II and thymus specific serine peptidase and is grouped in MEROPS[5] clan SC along with MEROPS[5] peptidase families such as S9, S10, S15 and S33. The predicted active site residues for the members of this family (S179, D430, H455) occur in the same order in the sequence as that of family S10.

Members of this family have been identified exclusively in eukaryotes and have been implicated in several processes (See additional file 3: Table S3.pdf)[4,62,63]. No functional information is available regarding their role in plants.

Analysis of the *Arabidopsis *proteome revealed the presence of seven genes encoding for the members of S28 family, while five genes were identified in rice. Of these, a majority are predicted to be the members of the secretory pathway, while two rice gene products LOC_Os01g56150 and LOC_Os10g36780 are predicted to be mitochondria localised (See additional files 1, 2, 12: Table S1.pdf, Table S2.pdf, Figure SF8.pdf). We further identified three orthologous pairs across the two species. S28 protease-like proteins identified here, like their eukaryotic counterparts are believed to be involved in processing of penultimate Proline containing polypeptides in the two plant species (See additional file 4: Table S4.pdf).

**Figure 7:**
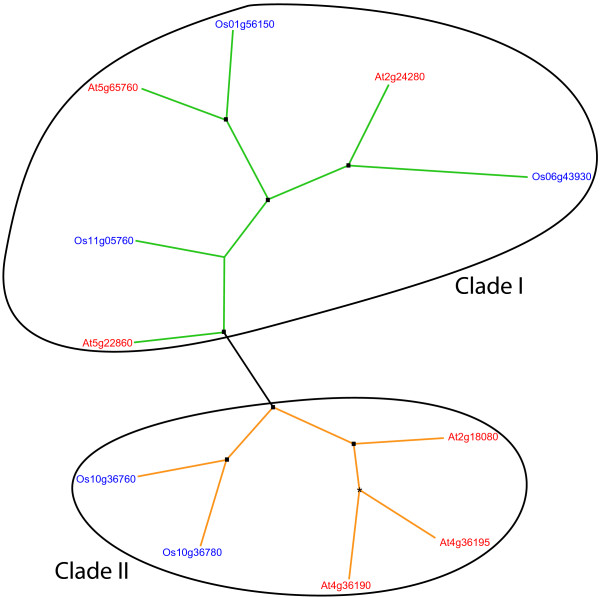
Unrooted N-J tree computed from multiple sequence alignments of *Arabidopsis *(red) and rice (blue) family S28 protease domains. S28-like protease domains were aligned using ClustalW [95] program and the alignments were exported to Phylip package [96] for representing the Neighbor-Joining tree (see methods). The colors represent the two evolutionary clades identified in the analysis (see text for details). Clade I is represented in Orange, Clade II is shaded green. For clarity, bootstrap values were replaced with symbols representing bootstrap percentages >50%. Bootstrap values between 50–60% are represented by an asterix, circles represent bootstrap values from 60%–80% while bootstrap values >80% are represented by rectangles. Gene names correspond to those in Additional files 1 and 2. For brevity, rice gene names have been shortened to OsXXg##### instead of LOC_OsXXg#####, XX referring to chromosome 1–12 and a 5 digit number assigned to each gene.

Phyolgenetic analysis reveals that *Arabidopsis *and rice proteins belonging to S28 family cluster into two clades (Figure 7). Clade I appears to be more diverse of the two clades and consists of six sequences that share between 40 and 69% pairwise sequence identities among themselves. Clade II consists of five sequences that are more closely related to each other than Clade I sequences and between 61 and 92% identical. The members of the two clusters share between 19 and 26% pairwise sequence identity with each other. These observations suggest the presence of two evolutionary clades among *Arabidopsis *and rice S28-like proteins prior to speciation.

### C-terminal processing peptidases (Family S41)

C-terminal processing peptidases (Ctps) belong to MEROPS[5] family S41. *E*.*coli *Tsp, a periplasmic endoprotease, was one of the first members of the family to be identified (See additional file 3: Table S3.pdf). It was subsequently determined that Most family members are associated with PDZ domains that are protein-protein interaction modules and are important for substrate recognition[24]. The C-terminal processing peptidase family consists of two subfamilies, which differ greatly in activity, sensitivity to inhibitors and molecular structure[5]. The two subfamilies employ different mechanisms for catalysis. While, the C-terminal processing peptidase subfamily (S41A) is found chiefly in bacteria and some eukaryotes employing a Ser-Lys catalytic dyad (S452, K477), the tricon core protease subfamily (S41B) is largely confined to archaea with few bacterial representatives employing a catalytic tetrad consisting of Ser, His, Ser and Glu residues (S745, H746, S965, E1023).

In bacteria, CPPs (such as *E*. *coli *Tsp) are believed to play an important role in degradation of improperly synthesised proteins. For instance, proteins translated from damaged mRNA are modified by addition of a C-terminal tag, which enables their recognition and degradation by CPPs[64]. In plants, Ctps have been implicated in processing D1 protein of photosystem II, which is essential for correct assembly and functioning of the plant photosynthetic apparatus[60,65,66].

We identified three genes each coding for Ctp-like proteins in *Arabidopsis *and rice proteomes. While the *Arabidopsis *proteins are all predicted to be soluble and have been shown to be translocated into thylakoid lumen, two of the three rice gene products (LOC_Os01g47450 and LOC_Os02g57060) are predicted to be mitochondria localised, while a single gene product LOC_Os06g21380 is predicted to be translocated to chloroplasts (See additional files 1, 2: Table S1.pdf, Table S2.pdf). All Ctp-like proteins identified in the two plant species carry a PDZ domain N-terminal to the protease domain (Figure 2; see additional files 1, 2: Table S1.pdf, Table S2.pdf). PDZ domains are protein-protein interaction modules that are likely to mediate substrate recognition for plant Ctp-like proteins[24]. Sequence analysis suggests that the Ctp-like proteins identified here may employ a serine-lysine catalytic dyad for proteolysis and possibly belong to S41A subfamily of C-terminal processing peptidases (See additional file 13: Figure SF9.pdf). We identified three orthologous pairs (At3g57680:LOC_Os06g21380, At4g17740:LOC_Os02g57060, At5g46390:LOC_Os01g47450) of six Ctp-like proteins across the two plant species (See additional file 4: Table S4.pdf). Each gene product displays a higher sequence identity with its orthologue in the corresponding genome than similar gene products from the same species. This suggests that these groups were most likely established early in evolution and disseminated in the two plant species through speciation. Like prokaryotic Ctps, plant Ctp-like proteins are believed to play a role in degradation of incorrectly synthesised proteins in chloroplasts and mitochondria.

### SppA/protease IV family (Family S49)

These groups of serine peptidases belong to MEROPS[5] family S49 and were first identified on the basis of their ability to degrade signal peptides that accumulate in the cytosol subsequent to their removal from precursor polypeptides by signal peptidases. Signal peptides are responsible for targeting various proteins to their correct subcellular localizations and are subsequently removed from the preproteins. The signal peptides generated following this cleavage need to be rapidly degraded since they may be harmful to cells by interfering with protein translocation or may accumulate in the membrane leading to cell lysis. *E*.*coli *SppA (protease IV), an inner membrane protein was the first protein identified with signal peptide peptidase activity (See additional file 3: Table S3.pdf)[57,67]. The predicted active site serine residue for members of Protease IV family and Clp protease family occur in the same order in the sequence: S, R/H, D and forms the basis for their assignment to SK clan[5].

Members of the protease IV family have been identified in viruses, archaea and bacteria and among the higher eukaryotes; they have been identified only in plants. Two proteins, SppA1 and SppA2 have been identified in *Synechosystis *while a single homologue SppA has been identified in *Arabidopsis *that lacks the putative transmembrane spanning segments predicted from the *E*.*coli *sequence[68].

Protease IV family appears to be represented by single members in *Arabidopsis *and rice proteomes and are not represented in any other eukaryotes. Though predicted by TargetP[19] to be mitochondria localised, experimental evidence indicates that *Arabidopsis *SppA (At1g73390) is a light-inducible membrane-bound protein with an unusual monotropic arrangement, associated with thylakoid membranes and has been proposed to be involved in light dependent degradation of photosystem II and LHCII antenna proteins[68]. A single SppA homologue in rice (LOC_Os2g49570) is also predicted to be mitochondria localised and in absence of experimental evidence, it would be tempting to speculate that the rice protein may also localise to chloroplasts. At1g73390 and LOC_Os02g49570 each carry two protease IV domains (Figure 2; see additional files 1, 2, 14: Table S1.pdf, Table S2.pdf, Figure SF10.pdf). Interestingly the N-terminal protease IV domain of At1g73390 is 63% identical to the C-terminal protease IV domain of the rice counterpart LOC_Os02g49570, while sharing only 17% identity with At1g73390 C-terminal protease IV domain and 14% identity with LOC_Os02g49570 N-terminal protease IV domain. Likewise LOC_Os02g49570 N-terminal protease IV domain is 75% identical to At1g73390 C-terminal protease IV domain and only 18% identical to LOC_Os02g49570 C-terminal protease IV domain. This clearly suggests that independent gene shuffling events subsequent to acquisition of SppA homologues in the two plant species may be responsible for the current domain architecture. Similar to their prokaryotic counterparts, they may function in association with signal-peptidases to degrade leader peptides removed from precursor proteins similar to their prokaryotic counterparts.

### Rhomboids (Family S54)

Rhomboid proteins are a family of serine proteases that cleave substrates within transmembrane domains[69,70]. Rhomboids are one of the most widespread membrane protein families and are found in archaea, bacteria and eukaryotes including plants[70,71] suggesting that while their signaling mechanism particularly protease activity may be conserved across lineages, they may have additional roles (See additional file 3: Table S3.pdf). A proposed catalytic triad comprising Asn169, Ser217 and His281 has been identified using site directed mutagenesis and each of proposed active site residues is located within a transmembrane domain[72]. However, a recent report suggests that while Ser217 and His281 along with a glycine residue (two residues N-terminal to Ser217) are essential for catalysis, Asn169 is not required for catalytic activity and that rhomboids likely function as endopeptidases with a serine-histidine dyad[73].

Rhomboid like proteins have been identified in plants, however, there was no experimental information available about them till recently. A rhomboid homologue from *Arabidopsis *AtRBL2 was recently isolated and shown to have proteolytic activity and substrate specificity[74]. AtRBL2 was shown to cleave *Drosophila *ligands Spitz and Keren but not similar proteins like TGFα, when expressed in mammalian cells, thereby releasing soluble ligands from the cell into the media. AtRBL2 along with AtRBL1, another *Arabidopsis *rhomboid homologue was found to localise to Golgi apparatus in plant cells consistent with previous observations where several eukaryotic rhomboid proteins have been predicted to localise within Golgi apparatus[72,74]. Interestingly, plants appear to lack any component of the EGF signaling pathway other than rhomboid and it has been suggested that rhomboid-like proteins in plants may be involved in processing as yet uncharacterised substrates that may be conserved in other organisms as well[74].

We identified 20 genes coding for rhomboid-like proteins in *Arabidopsis *and 18 in rice. Subsequent to multiple sequence alignment, it became evident that several proteins appeared to be catalytically inactive, owing to loss or mutation(s) in residues believed to be essential for catalysis. While the role of these catalytically compromised proteins remains unclear, it is believed that they may function as regulators of active rhomboid proteases[71] (See additional files 1, 2, 15: Table S1.pdf, Table S2.pdf, Figure SF11.pdf). Of all the rhomboid-like proteins identified in the two plant species, only a few are likely to carry additional domains. Three *Arabidopsis *gene products (At2g41160, At3g56740, At3g58460) and two gene products from rice (LOC_Os01g16330, LOC_Os03g44830) are predicted to possess a ubiquitin associated (UBA) domain, C-terminal to the rhomboid protease domain. An *Arabidopsis *gene product At3g17611 is predicted to have a zinc finger-like module C-terminal to the predicted rhomboid protease domain (Figure 2; see additional files 1, 2: Table S1.pdf, Table S2.pdf). We identified 10 orthologous pairs across *Arabidopsis *and rice, suggesting a high degree of conservation of rhomboid mechanisms across the two plant species (See additional file 4: Table S4.pdf). TargetP[19] predictions suggest that a few rhomboid-like proteins from both the plant species may localise to mitochondria or chloroplasts (See additional files 1, 2: Table S1.pdf, Table S2.pdf). Recently, a rhomboid protease was identified in yeast mitochondria, where it is involved in processing of substrates localised to the mitochondrial intermembrane space[75]. Although rhomboid-like proteins are yet to be identified in chloroplasts, identification of a yeast mitochondrial rhomboid protease coupled with the possibility that a few rhomboid-like proteins in the two plant species may localise to mitochondria and/or chloroplasts suggests that rhomboid-like proteins may be involved in regulatory processes in mitochondria and chloroplasts of higher plants.

**Figure 8:**
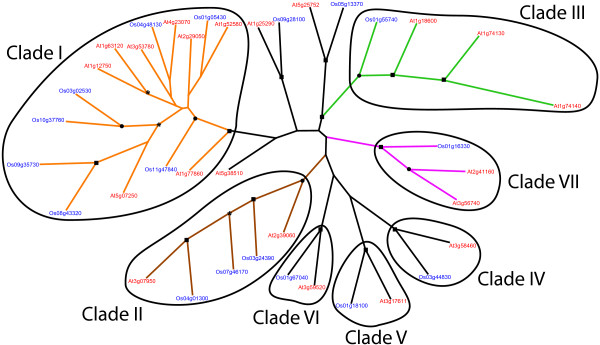
Unrooted N-J tree computed from multiple sequence alignments of *Arabidopsis *(red) and rice (blue) rhomboid protease domains. Rhomboid protease-like domains were aligned using ClustalW [95] program and the alignments were exported to Phylip package [96] for representing the Neighbor-Joining tree (see methods). The colors and circles represent different evolutionary clades identified in the analysis (see text for details). Clade I is represented in orange, Clade II is shaded brown, Clade III in green, Clade IV in purple. Clades V-VIII are shaded in black. For clarity, bootstrap values were replaced with symbols representing bootstrap percentages >50%. Bootstrap values between 50–60% are represented by an asterix, circles represent bootstrap values from 60%–80% while bootstrap values >80% are represented by rectangles. Gene names correspond to those in Additional files 1 and 2. For brevity, rice gene names have been shortened to OsXXg##### instead of LOC_OsXXg#####, XX referring to chromosome 1–12 and a 5 digit number assigned to each gene.

Phylogenetic analysis of *Arabidopsis *and rice rhomboid-like proteins reveals presence of several gene clusters that share low pairwise sequence identity with each other, though the members within a cluster share significant sequence similarity with each other (Figure 8). Clade I is the largest of the clusters supported by high bootstrap values and consists of 15 sequences, most of which, carry seven transmembrane helices (TMHs) and share greater than 40% pairwise sequence identity with each other. Three sequences in Clade I: At1g77860, At1g25290 and At5g07250 have been previously designated as members of RHO subfamily, one of the two major rhomboid subfamilies. The members of RHO subfamily typically have an extra TMH C-terminal to the 6-TMH core typical of most rhomboid-like proteins[71]. Since a majority of sequences that cluster into Clade I carry seven TMHs (data not shown), it is likely that they correspond to the RHO subfamily of rhomboid-like proteins. Clade II is the second largest cluster supported by significant bootstrap values and consists of five sequences (At2g39060, At3g07950, LOC_Os03g24390, LOC_Os04g01300 and LOC_Os07g46170). *Arabidopsis *genome encodes for four gene products (see below in bold) that were earlier identified as members of the PARL subfamily, the other major subfamily of rhomboid-like proteins[71]. The four gene products cluster into four independent groups, Clade III, consisting of four (**At1g18600**, At1g74130, At1g74140 and LOC_Os01g55740; 29–56% pairwise sequence identity), Clade IV, consisting of two (**At3g58460**, LOC_Os03g44830; 72% identical), Clade V, consisting of two (**At3g17611**, LOC_Os01g18100; 66% identical) and Clade VI consisting of two (**At3g59520**, LOC_0s01g67040; 80% identical) sequences respectively (Figure 8). A majority of rhomboid-like proteins identified in the two plant species were found to be single domain proteins, containing a single rhomboid protease domain. However, three *Arabidopsis *gene products (At2g41160, At3g56740 and At3g58460) were found to carry an additional UBA domain and At3g17611 was found to carry a zf-RAN domain C-terminal to the rhomboid protease domain (Figure 2; see additional file 1: Table S1.pdf). Two gene products in the rice genome, LOC_Os03g44830 and LOC_Os01g16330 were found to carry a UBA-domain C-terminal to the rhomboid protease domain (Figure 2; see additional file 2: Table S2.pdf). Interestingly, the UBA domain containing rhomboid-like proteins were observed to fall into two clusters Clade IV (At3g58460 and LOC_Os03g44830) and Clade VII (At2g41160, At3g56740 and LOC_Os01g16330; 69–85% pairwise sequence identity), suggesting that the rhomboid-UBA domain architecture probably evolved early in evolution, prior to divergence of dicot and monocot lineages in higher plants.

The members of individual gene clusters (except Clade II) share high pairwise sequence identities with each other (see above) but share low sequence identity with other rhomboid-like proteins identified in the two plant genomes and are likely to have diverged early in evolution. Our results may in part be explained by previous observations by Koonin and coworkers that reveal a polyphyletic origin for eukaryotic rhomboids and the possibility of a bacterial origin for rhomboid-like proteins and their subsequent dissemination in other lineages through multiple horizontal gene transfer events followed by extensive gene duplication[71].

### Nucleoporin autopeptidases (Family S59)

Nucleoporin autopeptidases are a group of autocatalytic serine endopeptidases belonging to MEROPS[5] family S59. Most nucleoporins are synthesised as precursors and processed by autoproteolysis prior to their association in the Nuclear Pore Complex (NPC). Studies have indicated that the C-terminal domain of the nucleoporins is essential for their localization to the NPC. Mutations that block proteolytic processing within the C-terminal domain prevent their association with the pore and inhibit their ability to form NPCs[76,77]. Structural analysis of the proteolytic cleavage site confirms the presence of a catalytic dyad (H879, S881) within **His**Phe**Ser **motif and an intein-like mechanism for autoproteolysis (See additional file 3: Table S3.pdf)[77].

Members of the S59 family have been identified across eukaryotes, from yeast to humans. Recently, a protein MOS3 with high sequence similarity to human nucleoporin 96 has been identified in *Arabidopsis *and shown to be required for activation of disease resistance response against bacterial pathogens. It was experimentally shown to localise to the nuclear envelope, suggesting that nucleocytoplasmic trafficking may play a significant role in plant disease resistance[78].

Analysis of *Arabidopsis *and rice proteomes led to identification of three genes each coding for nucleoporin-like proteins (See additional files 1, 2, 16: Table S1.pdf, Table S2.pdf, Figure SF12.pdf). As mentioned earlier, MOS3 (At1g80680), a likely homologue of human nucleoporin 96, plays a significant role in *Arabidopsis *resistance to bacterial pathogen[78]. We identified a MOS3 orthologue in rice, LOC_Os03g07580, which is of similar size and may play a similar role in defense response to bacterial pathogens in rice (See additional file 4: Table S4.pdf). In addition, *Arabidopsis *proteome also contains two gene products At1g10390 and At1g59660 that are 76% identical and show high similarity to human Nup98. In humans, Nup96 and Nup98, are encoded by the same gene and synthesised as a part of the same precursor polypeptide while, in *Arabidopsis*, they are encoded at different chromosomal locations. Rice proteome contains two gene products LOC_Os12g06870 and LOC_Os12g06890 that are 95% identical and are very similar to At1g10390 and may be involved in similar functional pathways in rice.

### Domain architectures

A protein domain maybe defined as an independent evolutionary unit that may either occur on its own in a single domain protein or alongside other similar units in a multi-domain protein. A domain may have an independent function or may contribute to the function of a multi-domain protein in cooperation with other domains. Some of the major mechanisms that lead to multi-domain proteins and novel combinations are domain shuffling and gene fusion[79,80].

The serine protease-like proteins belonging to different families identified in the two plant species exhibit 31 different domain architectures (Figure 2; see additional files 1, 2: Table S1.pdf, Table S2.pdf). The different serine protease-like proteins display a preference for co-existing domain(s), when present in multi-domain polypeptides, where the co-existing domains are believed to either facilitate protein-protein interactions or help evolve newer and more diverse functions for serine protease-like proteins in the cellular networks. We observed that a majority of serine protease-like proteins belonging to a specific family exhibit the same domain architectures with a few members exhibiting different domain architectures (Figure 2; see additional files 1, 2: Table S1.pdf, Table S2.pdf). We identified some gene products that contain serine protease-like domains and exhibit novel domain architectures specific to either of the two plant species. The information on co-existing domains described below was obtained from Pfam[37] (See Figure 2 for Pfam[37] accessions).

1. LOC_Os01g17160 (Subtilisin family S8)- Domain architecture SN-S8-PA-zfCCHC (Figure 2; see additional file 2: Table S2.pdf). The putative gene product is associated with a Zinc knuckle motif C -terminal to the subtilisin protease domain, which is associated with nucleocaspid protein of some retroviruses such as HIV. It is required for viral genome packaging and is also found in eukaryotic proteins involved in RNA binding or single stranded DNA binding.

2. LOC_Os04g02960 (Subtilisin family S8)- Domain architecture Ex2-S8-zfCCHC-rve (Figure 2; see additional file 2: Table S2.pdf). The putative gene product carries an Extensin_2 domain N-terminal to the subtilisin protease domain. This domain is associated with a family of plant hydroxyproline-rich glycoproteins (HRGPs) found in plant extracellular matrix that play a major role in cell wall self-assembly and cell extension. The gene product also contains a zhCCHC domain and an RVE domain C-terminal to the protease domain. The RVE (integrase core) domain is associated with viral proteins that mediate the integration of a DNA copy of the viral genome into the host chromosome.

3. LOC_Os06g06800 (Subtilisin family S8)- Domain architecture S8-zfCCHC-rve. The putative gene product carries a zhCCHC domain and an rve domain C-terminal to the protease domain (Figure 2; see additional file 2: Table S2.pdf).

4. LOC_Os06g40700 (Subtilisin family S8)- Domain architecture SN-S8-PA-Arf2-C2. The putative gene product carries an Arf2 and a C2 domain C-terminal to the subtilisin protease domain (Figure 2; see additional file 2: Table S2.pdf). Arf2 domain is associated with members ADP-ribosylation factor family that includes small GTP-binding proteins that are major regulators of vesicle biogenesis in intracellular traffic. C2 domain is a calcium-dependent membrane-targeting module found in many cellular proteins associated with signal transduction or membrane trafficking.

5. LOC_Os06g32740 (serine carboxypeptidase family S10)- Domain architecture S10-Transposase_21 (Figure 2; see additional file 2: Table S2.pdf). The putative gene product is associated with Transposase_21 domain C-terminal to the serine carboxpeptidase domain. The TP21 domain is associated with a group of proteins that include a transposable element.

6. LOC_Os11g27170 (serine carboxypeptidase family S10)- Domain architecture S10-Retrotrans_gag (Figure 2; see additional file 2: Table S2.pdf). The putative gene product is associated with a Retrotrans_gag domain C-terminal to the serine carboxypeptidase protease domain. The Retrotrans_gag domain is associated with groups of proteins that include eukaryotic Gag or capsid-related retrotranposon-related proteins.

7. At5g24810, LOC_Os06g48770 (serine β-lactamse family S12)- Domain architecture ABC1-S12. The putative gene products are associated with an ABC1 domain N-terminal to the serine β-lactamase domain (Figure 2; see additional files 1, 2: Table S1.pdf, Table S2.pdf). ABC1 domains are associated with a family of proteins that include members from yeast and bacteria that display a nuclear or mitochondrial subcellular location in eukaryotes. Some members of the family are believed to regulate mRNA translation and are essential for electron transfer in the bc1 complex.

8. At2g41160, At3g56740, At3g58460, LOC_Os01g16330, LOC_Os03g44830 (rhomboid family S54)- Domain architecture S54-UBA. The putative gene products are associated with an UBA domain C-terminal to the rhomboid protease domain (Figure 2; see additional files 1, 2: Table S1.pdf, Table S2.pdf). UBA-domain is a novel sequence motif found in diverse proteins associated with ubiquitin and the ubiquitination pathway.

9. At3g17611 (rhomboid family S54)- Domain architecture S54-zfRanBP. The putative gene product is associated with a zf-RanBP domain C-terminal to the rhomboid protease domain (Figure 2; see additional files 1, 2: Table S1.pdf, Table S2.pdf). The zf-RanBP domain is associated with a group of proteins that include Ran binding proteins that play a role in transport between the nucleus and the cytoplasm.

The gene products above, unlike other members of their respective serine protease families, are associated with domains forming novel domain combinations. It is likely that the protease domains may be responsible for regulating the activities of the associated domains for some of the gene products.

### Orthologue sequence analysis and gene duplications

Orthologues are genes in different organisms that have evolved from a common ancestor gene by speciation. They normally retain the same function in the course of evolution and the identification of orthologues is important for reliable prediction of gene functions and for comparing genome organizations[81]. Nearly 40% of genes encoding for serine protease-like proteins in the two plant species were identified as orthologues, suggesting that serine protease functions may have been established prior to dicot-monocot divergence (See additional file 4: Table S4.pdf). Comparison of *Arabidopsis thaliana *serine protease-like genes of known functions with similar gene products encoded in the rice genome has enabled the identification of rice serine-protease like gene products likely to be involved in similar physiological processes in rice (See additional file 4: Table S4.pdf).

When comparing multi-gene families between species it is frequently observed that several genes in one species may collectively be orthologues of a single gene in the other, indicating recent duplications exclusive to the former. In such cases, knowledge of gene function of certain members allows confirmation of orthologous and paralogous relationships. One such instance is *Arabidopsis *AIR3 (At2g04160), which has been associated with lateral root emergence[12] and has a putative orthologue (LOC_Os02g10520) in rice (See additional file 4: Table S4.pdf). However, AIR3 also appears to be an orthologue of LOC_Os06g40700, which is most closely related to LOC_Os02g10520 and LOC_Os08g23740, which is also closely related to LOC_Os02g10520 in terms of sequence similarity. Therefore, the aforementioned rice genes represent paralogous genes resulting from recent duplication events and it would be interesting to investigate whether they are also associated with lateral root emergence like their *Arabidopsis *counterpart and whether they display redundancy in function. Similarly, *Arabidopsis *ARA12 [12] (At5g67360) has a putative orthologue LOC_Os03g55350 in rice (See additional file 4: Table S4.pdf) and is likely to be associated with cell wall metabolism. ARA12 also appears to be an orthologue of LOC_Os03g40830 which, is a likely paralogue of LOC_Os03g55350 (Table 3), suggesting a recent duplication event in rice genome with respect to At5g67360 (ARA12) in *Arabidopsis*.

Gene duplication, resulting from regional events or genome-wide polyploidization is a significant aspect of plant genome evolution. It is generally believed that extensive gene duplication is responsible for functional divergence, increased genomic and phenotypic complexity and may constitute a major factor in speciation. Understanding the mechanisms that underlie gene duplication is vital since these provide greater insights into genome-wide aspects of evolutionary processes that shape genome organizations, evolutionary relationships and interactions[82,83]. Comparison of plant serine proteases with those of other major eukaryotic lineages reveals identification of plant specific serine proteases and selective expansion of serine protease families in plants (data not shown). In order to explore possible functional redundancy in plant serine protease-like proteins, we investigated chromosomal locations of serine protease-like proteins from the two plant species and inferred probable gene duplication events that may have facilitated this distribution. We observed that plant serine protease-like proteins are distributed on nearly on all the chromosomes in the two plant species. Several serine protease-like proteins in the two plant species were found to occur in tandem repeats, suggesting that local duplication events may have contributed to expansion of these gene families (See additional files 1, 2: Table S1.pdf, Table S2.pdf). It was also noted that several sequences identified as probable most recent gene duplicates in the two plant species were located on different chromosomes (Tables 2, 3). In order to analyse the role of segmental duplications in generating these gene duplicates, we compared our results on most recent gene duplicates with the TIGR[20] dataset of genes found within segmentally duplicated regions of the *Arabidopsis *and rice genomes and were able to identify instances of segmentally duplicated genes corresponding to certain families of serine proteases in *Arabidopsis *and rice genomes (Tables 2, 3). Therefore, some of the observed most recent gene duplications on different chromosomes in *Arabidopsis *and rice genomes may be attributed to segmental duplications. It seems likely that the expansion of certain serine protease families in the two plant species may be a consequence of extensive segmental and tandem gene duplication events subsequent to speciation (Tables 2, 3). Our analysis appears to be consistent with recent observations that segmental and tandem gene duplication events may have led to selective expansion of several gene families in *Arabidopsis *and rice genomes[84,85]. It has been suggested that, in general, duplicated genes have a tendency to acquire newer and more distinct functions. This is possible mainly due to differential sequence evolution and divergent expression patterns thereby adding to the complexity of cellular networks[15,84-87].

## Conclusion

The current analysis provides an overview and a comparison of serine protease families of *Arabidopsis *and rice, the two plant species with completely sequenced genomes that constitute model plant systems for dicots and monocots, respectively. The genomes of *Arabidopsis thaliana *and rice encode very similar number of serine protease-like gene products despite large differences in gene size and gene content. Similar results have been obtained from comparison of other gene families such as ABC transporters and P-ATPases though unlike serine proteases, a differential distribution of families and subfamilies has been observed in the above two cases. Nearly 40% of genes encoding for serine protease-like proteins in the two plant species were identified as orthologues, suggesting that some essential plant serine protease functions may have been established prior to dicot-monocot divergence. This is further supported by our observation that almost all the phylogenetic clades corresponding to different serine protease families taken up for study in this analysis include both *Arabidopsis *and rice gene products indicating that divergence of these clades occurred prior to the divergence of two species. However, several clades display an unequal representation of gene products from the two species indicating that a significant number of serine protease genes in the two plant species may have evolved by gene duplication events subsequent to speciation. In addition, plants appear to share with prokaryotes, some specific sets of serine protease-like proteins, not identified in other eukaryotes, which may have originated from ancestral chloroplast genomes and may be indicative of ancient endosymbiotic events leading to evolution of chloroplasts. Comparison of *Arabidopsis thaliana *serine protease-like proteins of known functions with similar gene products encoded in the rice genome has enabled the identification of rice serine-protease like proteins likely to be involved in similar physiological processes associated with growth and development in rice. In addition, identification of serine protease-like proteins in either plant species with no apparent counterpart in the other species indicates the existence of *Arabidopsis *and rice specific serine protease-like proteins and by extension, dicot- and monocot-specific serine protease-like proteins. The systematic analysis of the serine protease-like proteins in the two plant species has provided some insight into the functional associations of previously uncharacterised serine protease-like proteins. Further investigation of these aspects may help unravel more specific functional roles for several serine protease-like proteins in various cellular and physiological processes in *Arabidopsis *and rice and may prove beneficial in our understanding of similar processes in commercially significant crop plant species.

## Methods

Sequences encoded by the complete *Arabidopsis *and rice (*Oryza sativa *L.ssp. japonica) proteomes were obtained from The Institute for Genomic research (TIGR[20]).

### Search for serine proteases in the two plant genomes

a. A preliminary search for serine-proteases was performed using BLASTP[88] on *Arabidopsis *and rice proteomes, using Type sequences for each serine-protease family as classified by MEROPS[5] as queries. MEROPS[5] employs a hierarchical structure based classification of proteins and groups the peptidases together on the basis of statistical similarities in amino acid sequence and tertiary structure. Each family has a type example around which the family is built. All the members from the resulting list of similar sequences were retrieved for another round of searching and new accession numbers added to the original list of sequences. The procedure was repeated for five iterations with an E-value threshold of 10^-3^.

b. The protein sequences thus obtained were scanned further against a dataset of hidden Markov models (HMMs) obtained from the Pfam A database[37], employing the Hmmsearch of the HMMER suite[16], with the E-value thresholds set to 0.1[89].

c. A complimentary approach of matching sequences to a database of annotated profiles was also employed. Each *Arabidopsis *and rice genome sequence was matched to sensitive protein family profiles obtained from the PfamA database[37], using IMPALA[17]. A sequence aligning with any of the serine-protease domains considered for study with an E-value of <10^-5 ^by IMPALA[17] was considered as a potential member of that family.

d. A similar approach was carried out where each of *Arabidopsis *and rice genome sequences was scanned against protein family profiles obtained from the NCBI (National Centre for Biotechnology Information) conserved domain database (NCBI-CDD[18]), a compilation of multiple sequence alignments representing protein domains. It has been populated with alignment data from Pfam[37] and SMART [90], plus contributions from NCBI such as COG[91] using RPS-BLAST[18]. The E-value threshold was the same as employed for IMPALA[17].

e. The hits obtained from all the above methods were pooled together and a non-redundant dataset was derived. Proteins in this dataset were clustered using CD-HIT program[92] and MALIGN[93] and the hits sharing 100% sequence identity with other proteins were classified as redundant and not considered for further analysis. Proteins appearing as fragments or identical to larger proteins were also purged from the dataset.

### Sequence properties of *Arabidopsis* and rice serine proteases

a. The cellular localizations of serine protease-like proteins identified in the two plant species was predicted using TargetP[19].

b. Co-existing domains were predicted employing HMMPFAM[16] and RPS-BLAST[18] sequence to profile matching methods. Each serine-protease sequence was matched to protein family profiles corresponding to PfamA[37] and NCBI-CDD[18]. Conservation of domain architectures across various lineages (Figure 2) was determined using NCBI Conserved domain architecture retrieval tool (CDART)[94] and Pfam database[37].

### Orthologue sequence analysis

Each of the *Arabidopsis *and rice serine protease-like proteins was searched against the other proteome and conversely, the nearest homologue was searched back in first proteome. Two sequences were defined as orthologues when each of them was the best hit of the other and if they exhibit the same domain architecture (See additional file 4: Table S4.pdf).

### Chromosomal locations and recent duplications

The chromosomal locations for all *Arabidopsis *and rice serine protease sequences were retrieved from TIGR[20]. Subsequently, the *Arabidopsis *and rice proteomes were searched for gene paralogues using a BLAST[18] based approach similar to the one employed for orthologue sequence analysis and two sequences were defined as most recent paralogues when each of them was the best non-self hit of the other (Tables 2, 3).

### Multiple sequence alignment and phylogenetic analysis

Multiple sequence alignments of the serine-protease domains were performed using CLUSTALW program[95]. In order to compare equivalent regions, the domain regions were retrieved employing HMMALIGN[16], sequence to profile matching method against the PfamA database[37]. Proteins lacking a significant portion of the protease-like domain were not included in alignments. A Blosum 30 matrix, an open gap penalty of 10 and an extension penalty of 0.05 were the parameters employed for multiple sequence alignment. An overall phylogenetic tree was inferred from the multiple sequence alignment with PHYLIP (Phylogeny Inference Package) 3.65[96]. Bootstrapping was performed 100 times using SEQBOOT[96] to obtain support values for each internal branch (to reduce the sampling error, bootstrapping is a method of testing the reliability of a dataset by the creation of pseudo replicate datasets by resampling. Bootstrapping assesses whether stochastic effects have influenced the distribution of amino acids). Pairwise distances were determined with PROTDIST[96]. Neighbor-joining phylogenetic trees were calculated with NEIGHBOR[96] using standard parameters. The majority-rule consensus trees of all bootstrapped sequences were obtained with the program CONSENSE[96]. Representations of the calculated trees were constructed using TreeView[97]. Clusters with bootstrap values greater than 50% were defined as confirmed subgroups, and sequences with lower values added to these subgroups according to their sequence similarity in the alignment as judged by visual inspection. The pairwise percentage identity between the serine protease-like domain regions of any two sequences belonging to the same serine protease family was determined by MALFORM, a constituent of MALIGN multiple alignment program[93].

## Abbreviations

AGI- Arabidopsis Genome Initiative; IRGSP- International Rice Genome Sequencing Project; TIGR- The Institute for Genomic Research

## Authors' contributions

LT carried out the computational sequence analysis. LT and RS conceived of the study and participated in its design and coordination. LT authored the first draft of this manuscript and RS provided comments and revisions to the final version of this text. Both authors read and approved the final manuscript.

## Supplementary Material

Additional file 1 Table S1. An inventory of *Arabidopsis thaliana *serine protease-like proteins. An inventory of *Arabidopsis thaliana *serine protease-like proteins identified by multifold approach (see methods for details). The list includes gene identifiers, predicted subcellular localization, chromosome location, chromosomal nucleotide position and domain architectures of serine proteases identified in current analysis Click here for file

Additional file 2 Table S2. An inventory of rice serine protease-like proteins. An inventory of rice serine protease-like proteins identified by multifold approach (see methods for details). The list includes gene identifiers, predicted subcellular localization, chromosome location, chromosomal nucleotide position and domain architectures of serine proteases identified in current analysis Click here for file

Additional file 3 Table S3. Background information on serine proteases. Additional literature information on serine protease families taken up for study in current analysis. The information is categorized into three parts namely a brief structural overview, enzyme characteristics and functional information where known. Additional references for the material contained in the file have been provided at the end. Click here for file

Additional file 4 Table S4. Orthologous serine protease-like proteins identified in the two plant species. A list of *Arabidopsis thaliana *serine protease-like proteins and their putative orthologues in rice genome (see methods for details). Functional information gathered from literature has been provided for *Arabidopsis *gene products where possible Click here for file

Additional file 5 Figure SF1. Multiple sequence alignment and phylogenetic analysis of *Arabidopsis *and rice DegP protease-like proteins. **A**: Multiple sequence alignment of *Arabidopsis *and rice DegP protease-like proteins. Multiple sequence alignment of the DegP domain region of the annotated *Arabidopsis *and rice DegP protease-like proteins. The catalytic triad residues are indicated. Gene names correspond to those in Additional files 1 and 2. For brevity, rice gene names have been shortened to OsXXg##### instead of LOC_OsXXg#####, XX referring to chromosome 1–12 and a 5 digit number assigned to each gene. **B**: Phylogenetic analysis of *Arabidopsis *and rice DegP protease-like proteins. Unrooted N-J tree computed from multiple sequence alignments of *Arabidopsis *(red) and rice (blue) DegP protease domains. DegP protease-like protease domains were aligned using ClustalW[95] program and the alignments were exported to Phylip package[96] for representing the Neighbor-Joining tree (see methods). The colors and circles represent different evolutionary clades identified in the analysis (see text for details). Clade I is represented in orange, Clade II is shaded green while Clade III is labelled in purple. For clarity, bootstrap values were replaced with symbols representing bootstrap percentages >50%. Bootstrap values between 50–60% are represented by an asterix, circles represent bootstrap values from 60%–80% while bootstrap values >80% are represented by rectangles. Gene names correspond to those listed in Tables 2 and 3. For brevity, rice gene names have been shortened to OsXXg##### instead of LOC_OsXXg#####, XX referring to chromosome 1–12 and a 5 digit number assigned to each gene. Click here for file

Additional file 6 Figure SF2. Multiple sequence alignment of *Arabidopsis *and rice subtilisin-like proteins. Multiple sequence alignment of the subtilisin domain region of the annotated *Arabidopsis *and rice subtilisin protease-like proteins. The catalytic triad residues are indicated. Gene names correspond to those in Additional files 1 and 2. For brevity, rice gene names have been shortened to OsXXg##### instead of LOC_OsXXg#####, XX referring to chromosome 1–12 and a 5 digit number assigned to each gene. Click here for file

Additional file 7 Figure SF3. Multiple sequence alignment of *Arabidopsis *and rice prolyl oligopeptidase -like proteins Multiple sequence alignment of the Prolyl oligopeptidase domain region of the annotated *Arabidopsis *and rice prolyl oligopeptidase-like proteins. The catalytic triad residues are indicated. Gene names correspond to those in Additional files 1 and 2. For brevity, rice gene names have been shortened to OsXXg##### instead of LOC_OsXXg#####, XX referring to chromosome 1–12 and a 5 digit number assigned to each gene. Based on the conservation of residues around the catalytic Ser residue, a few gene products were unambiguously assigned to their respective subfamilies prefixed by figures in parentheses (See text for details) Click here for file

Additional file 8 Figure SF4. Multiple sequence alignment and phylogenetic analysis of *Arabidopsis *and rice serine carboxypeptidase-like proteins **A**: Multiple sequence alignment of *Arabidopsis *and rice serine carboxypeptidase-like proteins. Multiple sequence alignment of the serine carboxypeptidase domain region of the annotated *Arabidopsis *and rice serine carboxypeptidase-like proteins. The catalytic triad residues are indicated. Gene names correspond to those in Additional files 1 and 2. For brevity, rice gene names have been shortened to OsXXg##### instead of LOC_OsXXg#####, XX referring to chromosome 1–12 and a 5 digit number assigned to each gene. **B: **Phylogenetic analysis of *Arabidopsis *and rice serine carboxypeptidase-like proteins. Unrooted N-J tree computed from multiple sequence alignments of *Arabidopsis *(red) and rice (blue) serine carboxypeptidase domains. Serine carboxypeptidase-like protease domains were aligned using ClustalW[95] program and the alignments were exported to Phylip package[96] for representing the Neighbor-Joining tree (see methods). The colors and circles represent different evolutionary clades identified in the analysis (see text for details). Clade I is represented in orange, Clade II is shaded green while the branches connecting the remaining sequences are labelled in purple. For clarity, bootstrap values were replaced with symbols representing bootstrap percentages >50%. Bootstrap values between 50–60% are represented by an asterix, circles represent bootstrap values from 60%–80% while bootstrap values >80% are represented by rectangles. Gene names correspond to those listed in Tables 2 and 3. For brevity, rice gene names have been shortened to OsXXg##### instead of LOC_OsXXg#####, XX referring to chromosome 1–12 and a 5 digit number assigned to each gene. Click here for file

Additional file 9 Figure SF5. Pairwise sequence alignment of *Arabidopsis *and rice serine beta-lactamase -like proteins. Pairwise sequence alignment of the serine beta-lactamase domain region of the annotated *Arabidopsis *and rice serine beta-lactamase-like proteins. The catalytic residues are indicated. Gene names correspond to those in Additional files 1 and 2. For brevity, rice gene names have been shortened to OsXXg##### instead of LOC_OsXXg#####, XX referring to chromosome 1–12 and a 5 digit number assigned to each gene. Click here for file

Additional file 10 Figure SF6. Multiple sequence alignment of *Arabidopsis *and rice serine Lon protease-like proteins. Multiple sequence alignment of the Lon_C protease domain region of the annotated *Arabidopsis *and rice Lon protease-like proteins. The catalytic dyad residues are indicated. Gene names correspond to those in Additional files 1 and 2. For brevity, rice gene names have been shortened to OsXXg##### instead of LOC_OsXXg#####, XX referring to chromosome 1–12 and a 5 digit number assigned to each gene. Click here for file

Additional file 11 Figure SF7. Multiple sequence alignment of *Arabidopsis *and rice serine Type I SPase-like proteins. Unrooted N-J tree computed from multiple sequence alignments of *Arabidopsis *(red) and rice (blue) Type I SPase domains. Type I SPase domains were aligned using ClustalW[95] program and the alignments were exported to Phylip package[96] for representing the Neighbor-Joining tree (see methods). The colors represent the two evolutionary clades identified in the analysis (see text for details). Clade I is represented in green Clade II is shaded orange. For clarity, bootstrap values were replaced with symbols representing bootstrap percentages >50%. Bootstrap values between 50–60% are represented by an asterix, circles represent bootstrap values from 60%–80% while bootstrap values >80% are represented by rectangles. Gene names correspond to those listed in Tables 2 and 3. For brevity, rice gene names have been shortened to OsXXg##### instead of LOC_OsXXg#####, XX referring to chromosome 1–12 and a 5 digit number assigned to each gene. Click here for file

Additional file 12 Figure SF8. Multiple sequence alignment of *Arabidopsis *and rice serine S28 protease-like proteins. Multiple sequence alignment of the family S28 protease domain region of the annotated *Arabidopsis *and rice family S28 protease-like proteins. The catalytic triad residues are indicated. Gene names correspond to those in Additional files 1 and 2. For brevity, rice gene names have been shortened to OsXXg##### instead of LOC_OsXXg#####, XX referring to chromosome 1–12 and a 5 digit number assigned to each gene. Click here for file

Additional file 13 Figure SF9. Multiple sequence alignment of *Arabidopsis *and rice Ctpase-like proteins. Multiple sequence alignment of the Ctpase domain region of the annotated *Arabidopsis *and rice Ctpase-like proteins. The catalytic dyad residues are indicated. Gene names correspond to those in Additional files 1 and 2. For brevity, rice gene names have been shortened to OsXXg##### instead of LOC_OsXXg#####, XX referring to chromosome 1–12 and a 5 digit number assigned to each gene. Click here for file

Additional file 14 Figure SF10. Multiple sequence alignment of *Arabidopsis *and rice protease IV-like proteins. Multiple sequence alignment of the protease IV domain region of the annotated *Arabidopsis *and rice protease IV-like proteins. The active site serine residue is indicated. Gene names are derived from those in Additional files 1 and 2 as follows: A1a73990 (At1g73390 N-terminal domain); A1b73990 (At1g73390 C-terminal domain); O2a49570 (Os02g49570 N-terminal domain); O2b49570 (Os02g49570 C-terminal domain). For brevity, rice gene names have been shortened to OsXXg##### instead of LOC_OsXXg#####, XX referring to chromosome 1–12 and a 5 digit number assigned to each gene. Click here for file

Additional file 15 Figure SF11. Multiple sequence alignment of *Arabidopsis *and rice rhomboid-like proteins. Multiple sequence alignment of the rhomboid protease domain region of the annotated *Arabidopsis *and rice rhomboid protease-like proteins. The catalytic triad residues are indicated. Gene names correspond to those in Additional files 1 and 2. For brevity, rice gene names have been shortened to OsXXg##### instead of LOC_OsXXg#####, XX referring to chromosome 1–12 and a 5 digit number assigned to each gene. Several sequences identified here display mutations in key catalytic residues and may possibly have evolved a regulatory function (See text for details). Click here for file

Additional file 16 Figure SF12. Multiple sequence alignment of *Arabidopsis *and rice nucleoporin autopeptidase-like proteins. Multiple sequence alignment of the nucleoporin autopeptidase domain region of the annotated *Arabidopsis *and rice nucleoporin autopeptidase-like proteins. The catalytic residues are indicated. Gene names correspond to those in Additional files 1 and 2. For brevity, rice gene names have been shortened to OsXXg##### instead of LOC_OsXXg#####, XX referring to chromosome 1–12 and a 5 digit number assigned to each gene. Click here for file

## Figures and Tables

**Table 1:** Serine proteases identified in the two plant genomes, by sensitive sequence search methods

**CLAN**	**FAMILY**	**Pfam[37] Accession**	***Arabidopsis***	**Rice**
PA(S)	S1 (Chymotrypsin)	PF00089	16	15
SB	S8 (Subtilisin)	PF00082	56	63
SC	S9 (Prolyl oligopeptidase)	PF00326	23	23
	S10 (Carboxypeptidase)	PF00450	54	66
	S28 (Lys. Pro-x Carboxypeptidase)	PF05577	7	5
SE	S12 (D-Ala-D-Ala carboxypeptidase B)	PF00144	1	1
SF	S16 (Lon protease)	PF05362	4	4
	S26 (Signal peptidase I)	PF00717	9	7
SK	S14 (Clp Endopeptidase)	PF00574	9	13
SM	S41 (C-terminal processing peptidase)	PF03572	3	3
	S49 (Protease IV)	PF01343	1	1
	S54 (Rhomboid)	PF01694	20	18
SP	S59 (Nucleoporin autopeptidase)	PF04096	3	3

**Table 2:** A list of most recent serine protease gene duplications in* Arabidopsis thaliana *genome. The most recent gene duplicates identified in the segmentally duplicated regions of the* Arabidopsis *genome are suffixed with (S). See text for details

S. No.	Arabidopsis serine protease-like protein	Biological function if known	Most recent duplicate
Family S1 (Deg protease family)			
1.	At1g51150		At5g54745
2.	At1g65640 (S)		At5g36950 (S)
3.	At2g47940		At5g40200
4.	At3g16540		At3g16550
5.	At3g16550		At3g16540
6.	At3g27925		At5g39830
			
Family S8 (Subtilisin family)			
1.	At1g20150		At1g20160
2.	At1g32940		At1g32960
3.	At1g66210		At1g66220
4.	At2g04160		At5g59810
5.	At2g19170 (S)		At4g30020 (S)
6.	At3g14240		At4g34980
7.	At3g46840		At3g46850
8.	At4g00230		At5g03620
9.	At4g10520		At4g10530
10.	At4g10540		At4g10550
11.	At4g20430 (S)		At5g44530 (S)
12.	At4g21630		At4g21640
13.	At5g45640		At5g45650
14.	At5g51750		At5g67360
15.	At5g58820		At5g58830
16.	At5g59090		At5g59120
			
Family S9 (Prolyl oligopeptidase family)			
1.	At1g20380 (S)		At1g76140 (S)
2.	At1g52700		At3g15650
3.	At1g69020		At5g66960
4.	At3g02410 (S)		At5g15860 (S)
5.	At3g23540 (S)		At4g14290 (S)
6.	At4g14570		At5g36210
			
Family S10 (Serine carboxypeptidase family)			
1.	At1g11080 (S)		At1g61130 (S)
2.	At1g28110		At2g33530
3.	At1g61130		At1g11080
4.	At1g73300		At5g36180
5.	At2g22920		At2g22970
6.	At2g24000 (S)		At4g30610 (S)
7.	At2g35780		At3g07990
8.	At3g12230		At3g12240
9.	At3g25420 (S)		At4g12910 (S)
10.	At3g45010		At5g22980
11.	At3g52000		At3g52010
12.	At3g52020		At3g63470
13.	At5g42230		At5g42240
			
Family S14 (Clp protease family)			
1.	At1g09130		At1g49970
2.	At1g66670		At5g45390
			
Family S16 (Lon protease family)			
1.	At3g05790		At5g26860
			
Family S26 (Signal Peptidase family)			
1.	At1g06870		At2g30440
2.	At1g23465		At1g29960
3.	At1g52600 (S)		At3g15710 (S)
			
Family S28 (Lysosomal Pro-X Carboxypeptidase family)			
1.	At2g24280		At5g65760
2.	At4g36190		At4g36195
			
Family S41 (C-terminal processing peptidase family)			
1.	At3g57680		At4g17740
			
Family S54 (Rhomboid family)			
1.	At1g12750 (S)		At1g63120 (S)
2.	At1g74130		At1g74140
3.	At2g41160 (S)		At3g56740 (S)
			
Family S59 (Nucleoporin autopeptidase family)			
1.	At1g10390		At1g59660

**Table 3:** A list of most recent serine protease gene duplications in rice genome. The most recent gene duplicates identified in the segmentally duplicated regions of the rice genome are suffixed with (S). See text for details

S. No.	Rice serine protease-like protein	Gene duplicate
Family S1 (DegP protease family)		
1.	LOC_Os02g50880 (S)	LOC_Os06g12780 (S)
2.	LOC_Os04g38640	LOC_Os05g49380
3.	LOC_Os12g04740	LOC_Os12g04740
		
Family S8 (Subtilisin family)		
1.	LOC_Os01g56320	LOC_Os06g48650
2.	LOC_Os01g58240	LOC_Os01g58260
3.	LOC_Os01g58280	LOC_Os01g58290
4.	LOC_Os01g64860 (S)	LOC_Os05g36010 (S)
5.	LOC_Os02g10520 (S)	LOC_Os06g40700 (S)
6.	LOC_Os02g16940	LOC_Os02g17090
7.	LOC_Os02g17000	LOC_Os02g17060
8.	LOC_Os02g17090	LOC_Os02g16940
9.	LOC_Os02g44590 (S)	LOC_Os04g47150 (S)
10.	LOC_Os02g53910	LOC_Os02g53970
11.	LOC_Os03g02750 (S)	LOC_Os10g38080 (S)
12.	LOC_Os03g31630	LOC_Os07g48650
13.	LOC_Os03g40830	LOC_Os03g55350
14.	LOC_Os04g02980	LOC_Os04g03060
15.	LOC_Os04g03800	LOC_Os04g03810
16.	LOC_Os09g26920	LOC_Os10g25450
		
Family S9 (Prolyl oligopeptidase family)		
1.	LOC_Os01g01830	LOC_Os04g47360
2.	LOC_Os04g47360	LOC_Os01g01830
3.	LOC_Os06g11180	LOC_Os06g11190
4.	LOC_Os07g48970	LOC_Os07g48970
5.	LOC_Os09g28040	LOC_Os09g29950
6.	LOC_Os10g28020	LOC_Os10g28030
		
Family S10 (Serine carboxypeptidase family)		
1.	LOC_Os01g06490	LOC_Os01g61690
2.	LOC_Os01g11670	LOC_Os05g50600
3.	LOC_Os01g22980	LOC_Os05g06660
4.	LOC_Os02g02320	LOC_Os07g29620
5.	LOC_Os02g42310 (S)	LOC_Os04g44410 (S)
6.	LOC_Os03g26920	LOC_Os03g26930
7.	LOC_Os03g52040	LOC_Os03g52080
8.	LOC_Os04g25560	LOC_Os12g15470
9.	LOC_Os04g32540	LOC_Os11g31980
10.	LOC_Os05g50570	LOC_Os05g50580
11.	LOC_Os08g44640	LOC_Os08g44640
12.	LOC_Os10g01110	LOC_Os11g42390
13.	LOC_Os11g24290	LOC_Os11g24410
14.	LOC_Os11g24510	LOC_Os12g39170
15.	LOC_Os11g27270	LOC_Os11g27350
		
Family S14 (Clp protease family)		
1.	LOC_Os01g32350	LOC_Os03g19510
2.	LOC_Os02g42290 (S)	LOC_Os04g44400 (S)
3.	LOC_Os03g22430	LOC_Os05g51450
4.	LOC_Os08g15270	LOC_Os12g10590
		
Family S16 (Lon protease family)		
1.	LOC_Os01g54040 (S)	LOC_Os05g44590 (S)
2.	LOC_Os03g19350	LOC_Os07g48960
3.	LOC_Os03g29540	LOC_Os07g32560
		
Family S24 (Signal peptidase family)		
1.	LOC_Os03g55640	LOC_Os09g28000
2.	LOC_Os05g23260	LOC_Os06g16260
		
Family S28 (Lysosomal Pro-X Carboxypeptidase family)		
1.	LOC_Os01g56150	LOC_Os06g43930
2.	LOC_Os10g36780	LOC_Os10g36780
		
Family S41 (C-terminal processing peptidase family)		
1.	LOC_Os01g47450	LOC_Os06g21380
		
Family S54 (Rhomboid family)		
1.	LOC_Os01g18100	LOC_Os03g44830
2.	LOC_Os03g24390	LOC_Os07g46170 (S)
3.	LOC_Os08g43320 (S)	LOC_Os09g35730 (S)
		
Family S59 (Nucleoporin autopeptidase family)		
1.	LOC_Os12g06870	LOC_Os12g06890
