# Supplementary material for: Cross genome comparisons of serine proteases in Arabidopsis and rice
Source: BMC Genomics. 2006 Aug 9;7:200. doi: 10.1186/1471-2164-7-200 (PMC1560137; doi:10.1186/1471-2164-7-200)
Supplement: Additional file 1 — Table S1. An inventory of Arabidopsis thaliana serine protease-like proteins. An inventory of Arabidopsis thaliana serine protease-like proteins identified by multifold approach (see methods for details). The list includes gene identifiers, predicted subcellular localization, chromosome location, chromosomal nucleotide position and domain architectures of serine proteases identified in current analysis [file 1471-2164-7-200-S1.pdf]

Table S1: An inventory of *Arabidopsis thaliana* serine protease-like proteins identified by multifold approach (see methods for details)

S No.	Accession IDs	Subcellular location*	Chromosme location	Chromosomal nucleotide position	Domain architecture[#]
Peptidase_S1					
1.	At1g28320	-	chr01	9920494 – 9924475	SP1
2.	At1g51150 (DegP6)	C	chr01	18952038 - 18952958	SP1
3.	At1g65630 (DegP3)	M	chr01	24410205 - 24412731	SP1
4.	At1g65640 (DegP4)	M	chr01	24414514 - 24417190	SP1
5.	At2g47940 (DegP2)	C	chr02	19625120 - 19629302	SP1
6.	At3g03380 (DegP7)	-	chr03	799622 - 808529	SP1-PDZ
7.	At3g16540 (DegP11)	M	chr03	5626296 - 5628863	SP1
8.	At3g16550 (DegP12)	M	chr03	5629926 - 5632423	SP1
9.	At3g27925 (DegP1)	C	chr03	10367747 - 10370101	SP1-PDZ
10.	At4g18370 (DegP5)	C	chr04	10149203 - 10151722	SP1
11.	At5g27660 (DegP14)	M	chr05	9789902 - 9792427	SP1-PDZ
12.	At5g36950 (DegP10)	M	chr05	14612211 - 14615570	SP1
13.	At5g39830 (DegP8)	C	chr05	15960048 - 15963051	SP1-PDZ
14.	At5g40200 (DegP9)	C	chr05	16087558 - 16090542	SP1
15,	At5g40560 (DegP13)	S	chr05	16261771 - 16263747	SP1
16.	At5g54745	S	chr05	22257482 - 22258459	SP1
Peptidase_S8					
1.	At1g01900	S	chr01	310318 - 313130	SN-SP8-PA
2.	At1g04110 (SDD1)	S	chr01	1061456 - 1063783	SN-SP8-PA
3.	At1g20150	S	chr01	6987323 - 6990352	SN-SP8-PA
4.	At1g20160	S	chr01	6990785 - 6993882	SN-SP8-PA
5.	At1g30600	S	chr01	10841124 - 10845032	SN-SP8-PA
6.	At1g32940	S	chr01	11937576 - 11940958	SN-SP8-PA

7.	At1g32950	S	chr01	11941418 - 11944740	SN-SP8-PA
8.	At1g32960	S	chr01	11945287 - 11948630	SN-SP8-PA
9.	At1g32970	S	chr01	11948701 - 11951962	SP8-PA
10.	At1g32980 (ALE1)	-	chr01	11954258 - 11955342	SP8
11.	At1g62340	S	chr01	23054667 - 23059337	SN-SP8-PA
12.	At1g66210	S	chr01	24669292 - 24672446	SN-SP8-PA
13.	At1g66220	S	chr01	24674199 - 24677324	SN-SP8-PA
14.	At2g04160 (AIR3)	S	chr02	1401447 - 1407691	SN-SP8-PA
15.	At2g05920	S	chr02	2269513 - 2272226	SN-SP8-PA
16.	At2g19170 (SLP3)	S	chr02	8320584 - 8325678	SN-SP8-PA-D1034
17.	At2g39850	S	chr02	16637704 - 16641331	SN-SP8
18.	At3g14067	S	chr03	4658428 - 4660761	SN-SP8-PA
19.	At3g14240	S	chr03	4741480 - 4744124	SN-SP8-PA
20.	At3g46840	S	chr03	17261996 - 17265098	SN-SP8-PA
21.	At3g46850	S	chr03	17267323 - 17270427	SN-SP8-PA
22.	At4g00230 (XSP1)	S	chr04	93923 - 97449	SN-SP8-PA
23.	At4g10510	M	chr04	6495951 - 6499006	SN-SP8-PA
24.	At4g10520	S	chr04	6499790 - 6502862	SN-SP8-PA
25.	At4g10530	S	chr04	6508596 - 6511666	SN-SP8-PA
26.	At4g10540	S	chr04	6512511 - 6515739	SN-SP8-PA
27.	At4g10550	S	chr04	6516578 - 6519763	SN-SP8-PA
28.	At4g15040	-	chr04	8581368 - 8584117	SN-SP8-PA
29.	At4g20430	S	chr04	11017667 - 11021116	SN-SP8-PA
30.	At4g20850	C	chr04	11160913 - 11169900	SP8
31.	At4g21323	S	chr04	11342504 - 11345642	SN-SP8-PA
32.	At4g21326	-	chr04	11346991 - 11349664	SN-SP8-PA
33.	At4g21630	S	chr04	11492260 - 11495512	SN-SP8-PA
34.	At4g21640	S	chr04	11496846 - 11500630	SN-SP8-PA
35.	At4g21650	S	chr04	11501210 - 11504690	SN-SP8-PA
36.	At4g26330	-	chr04	13320417 - 13323470	SN-SP8-PA
37.	At4g30020	S	chr04	14677298 - 14681962	SN-SP8-PA-D1034
38.	At4g34980 (SLP2)	S	chr04	16656696 - 16659344	SN-SP8-PA
39.	At5g03620	S	chr05	918737 - 921873	SN-SP8-PA
40.	At5g11940	S	chr05	3849284 - 3852418	SN-SP8-PA
41.	At5g19660	M	chr05	6642006 - 6646532	SP8
42.	At5g44530	S	chr05	17955158 - 17958420	SN-SP8-PA
43.	At5g45640	S	chr05	17955158 - 17958420	SN-SP8-PA
44.	At5g45650	S	chr05	18530658 - 18536095	SN-SP8-PA
45.	At5g51750	S	chr05	21037433 - 21040007	SN-SP8-PA
46.	At5g58820	S	chr05	23769182 - 23771999	SN-SP8-PA

47.	At5g58830	-	chr05	23773199 - 23775910	SN-SP8
48.	At5g58840	S	chr05	23776229 - 23779285	SN-SP8
49.	At5g59090	S	chr05	23869131 - 23872501	SN-SP8-PA
50.	At5g59100	S	chr05	23876120 - 23879355	SN-SP8-PA
51.	At5g59120	S	chr05	23881956 - 23885275	SN-SP8-PA
52.	At5g59130	S	chr05	23887418 - 23890917	SN-SP8-PA
53.	At5g59190	-	chr05	23903081 - 23905899	SN-SP8-PA
54.	At5g59810	M	chr05	24114041 - 24117783	SN-SP8-PA
55.	At5g67090	S	chr05	26791337 - 26793547	SN-SP8-PA
56.	At5g67360 (ARA12)	S	chr05	26889117 - 26891805	SN-SP8-PA
Peptidase_S9					
1.	At1g13610	-	chr01	4664005 - 4665951	SP9
2.	At1g20380	-	chr01	7061655 - 7065168	S9N-SP9
3.	At1g26120	-	chr01	9028382 - 9031472	SP9
4.	At1g32190	C	chr01	11592935 - 11595962	SP9
5.	At1g50380	-	chr01	18665977 - 18670146	S9N-SP9
6.	At1g66900	-	chr01	24962997 - 24965279	SP9
7.	At1g69020	M	chr01	25947549 - 25950860	S9N-SP9
8.	At1g76140	-	chr01	28576194 - 28579930	SP9
9.	At2g24320	-	chr02	10352739 - 10353909	SP9
10.	At2g47390	C	chr02	19448742 - 19453435	SP9
11.	At3g01690	C	chr03	256562 - 258554	SP9
12.	At3g02410	-	chr03	492125 - 494884	SP9
13.	At3g30380	-	chr03	11976903 - 11979050	SP9
14.	At3g47560	-	chr03	17536162 - 17538008	SP9
15.	At4g14290	-	chr04	8225476 - 8230277	SP9
16.	At4g14570	-	chr04	8362582 - 8366649	SP9
17.	At4g24760	-	chr04	12761293 - 12763791	SP9
18.	At5g15860	-	chr05	5178508 - 5181293	SP9
19.	At5g20520	S	chr05	6943156 - 6946451	SP9
20.	At5g24260	-	chr05	8234726 - 8238246	SP9
21.	At5g25770	M	chr05	8969219 - 8972049	SP9
22.	At5g36210	C	chr05	14265143 - 14270460	SP9
23.	At5g66960	M	chr05	26753435 - 26756719	S9N-SP9
Peptidase_S10					
1.	At1g11080	S	chr01	3694707 - 3698013	SP10
2.	At1g15000	S	chr01	5168591 - 5170061	SP10
3.	At1g28110	S	chr01	9803628 - 9806883	SP10
4.	At1g33540	S	chr01	12162329 - 12164680	SP10
5.	At1g43780	S	chr01	16566251 - 16569839	SP10
6.	At1g61130	S	chr01	22532689 - 22535449	SP10
7.	At1g73270	S	chr01	27553067 - 27556178	SP10

8.	At1g73280	S	chr01	27556631 - 27558983	SP10
9.	At1g73290	S	chr01	27560058 - 27562434	SP10
10	At1g73300	S	chr01	27563334 - 27565709	SP10
11.	At1g73310	S	chr01	27566476 - 27568838	SP10
12.	At2g05850	S	chr02	2242678 - 2244484	SP10
13.	At2g12480	S	chr02	5076701 - 5079615	SP10
14.	At2g22920 (SNG1/SCT)	S	chr02	9761018 - 9764680	SP10
15.	At2g22960	S	chr02	9777974 - 9780012	SP10
16.	At2g22970	S	chr02	9781933 - 9785606	SP10
17.	At2g22980	-	chr02	9786419 - 9790258	SP10
18.	At2g22990	S	chr02	9793396 - 9797259	SP10
19.	At2g23000	S	chr02	9799292 - 9802940	SP10
20.	At2g23010	S	chr02	9805923 - 9809754	SP10
21.	At2g24000	S	chr02	10216555 - 10221122	SP10
22.	At2g24010	M	chr02	10221794 - 10224622	SP10
23.	At2g27920	S	chr02	11892648 - 11896216	SP10
24.	At2g33530	S	chr02	14204729 - 14207633	SP10
25.	At2g35770	S	chr02	15041116 - 15043597	SP10
26.	At2g35780	S	chr02	15044741 - 15047195	SP10
27.	At3g02110	S	chr03	370777 - 373729	SP10
28.	At3g07990	S	chr03	2552483 - 2554927	SP10
29.	At3g10410	S	chr03	2552483 - 2554927	SP10
30.	At3g10450	S	chr03	3249536 - 3252511	SP10
31.	At3g12203	S	chr03	3891364 - 3893963	SP10
32.	At3g12220	S	chr03	3896538 - 3899026	SP10
33.	At3g12230	S	chr03	3899438 - 3901886	SP10
34.	At3g12240	S	chr03	3902443 - 3904925	SP10
35.	At3g17180	S	chr03	5855867 - 5859163	SP10
36.	At3g25420	S	chr03	9219069 - 9222162	SP10
37.	At3g45010	S	chr03	16477275 - 16479952	SP10
38.	At3g52000	S	chr03	19304049 - 19305680	SP10
39.	At3g52010	S	chr03	19307222 - 19309051	SP10
40.	At3g52020	S	chr03	19310287 - 19312054	SP10
41.	At3g56540	S	chr03	20961802 - 20962871	SP10
42.	At3g63470	S	chr03	23449449 - 23452035	SP10
43.	At4g12910	S	chr04	7550434 - 7553329	SP10
44.	At4g15100	S	chr04	8626265 - 8629528	SP10
45.	At4g30610	C	chr04	14944135 - 14948611	SP10
46.	At4g30810	S	chr04	15003457 - 15006217	SP10
47.	At5g08260	S	chr05	2657168 - 2661413	SP10
48.	At5g09640 (SNG2/SMT)	S	chr05	2988315 - 2991157	SP10
49.	At5g22960	S	chr05	7684017 - 7685055	SP10
50.	At5g22980	S	chr05	7688087 - 7690651	SP10

51.	At5g23210	-	chr05	7811613 - 7815042	SP10
52.	At5g36180	S	chr05	14256407 - 14259294	SP10
53.	At5g42230	S	chr05	16898188 - 16901292	SP10
54.	At5g42240	S	chr05	16905461 - 16908420	SP10
Peptidase_S12					
1.	At5g24810	M	chr05	8516638 - 8522770	ABC1-SP12
Peptidase_S14					
1.	At1g02560 (NClpP1)	C	chr01	537888 - 540109	SP14
2.	At1g09130	C	chr01	2939572 - 2942257	SP14
3.	At1g11750	C	chr01	3967478 - 3969854	SP14
4.	At1g12410 (NClpP2)	C	chr01	4223035 - 4225112	SP14
5.	At1g49970 (NClpP5)	C	chr01	18505201 - 18508277	SP14
6.	At1g66670 (NClpP3)	C	chr01	24867448 - 24869363	SP14
7.	At4g17040	C	chr04	9585724 - 9589381	SP14
8.	At5g23140	M	chr05	7783761 - 7785500	SP14
9.	At5g45390 (NClpP4)	C	chr05	18413530 - 18415343	SP14
Peptidase_S16					
1.	At3g05780	M	chr03	1714947 - 1719614	LON-AAA-SP16
2.	At3g05790	C	chr03	1720160 - 1725188	LON-AAA-SP16
3.	At5g26860	C	chr05	9451082 - 9456872	LON-AAA-SP16
4.	At5g47040	M	chr05	19110583 - 19115942	LON-AAA-SP16
Peptidase_S26					
1.	At1g06870	M	chr01	2108592 - 2110985	SP26
2.	At1g23465	-	chr01	8330044 - 8330927	SP26
3.	At1g29960	-	chr01	10494797 - 10497271	SP26
4.	At1g52600	-	chr01	19594194 - 19596343	SP26
5.	At1g53530	M	chr01	19981821 - 19983751	SP26
6.	At2g30440 (TPP)	M	chr02	12979954 - 12982574	SP26
7.	At3g08980	S	chr03	2740813 - 2742690	SP26
8.	At3g15710	-	chr03	5323365 - 5324915	SP26
9.	At3g24590	C	chr03	8970679 - 8972175	SP26
Peptidase_S28					
1.	At2g18080	-	chr01	7864598 - 7866800	SP28
2.	At2g24280	S	chr02	10340364 - 10344161	SP28
3.	At3g28680	S	chr03	10749988 - 10750971	SP28

4.	At4g36190	S	chr04	17124055 - 17127025	SP28
5.	At4g36195	S	chr04	17124055 - 17127025	SP28
6.	At5g22860	S	chr05	7639359 - 7642987	SP28
7.	At5g65760	S	chr05	26325484 - 26328247	SP28
Peptidase_S41					
1.	At3g57680	C	chr01	21392031 - 21394606	SP41
2.	At4g17740	C	chr04	9866990 - 9869765	SP41
3.	At5g46390	C	chr05	18833831 - 18836574	SP41
Peptidase_S49					
1.	At1g73990 (SppA)	M	chr01	27828094 - 27832587	SP49
Peptidase_S54					
1.	At1g12750 (AtRBL6)	M	chr01	4344983 - 4348325	SP54
2.	At1g18600	M	chr01	6400396 - 6402770	SP54
3.	At1g25290	C	chr01	8867144 - 8869079	SP54
4.	At1g52580 (AtRBL5)	-	chr01	19591306 - 19592721	SP54
5.	At1g63120 (AtRBL2)	-	chr01	23412541 - 23414480	SP54
6.	At1g74130	C	chr01	27877318 - 27879694	SP54
7.	At1g74140	C	chr01	27880589 - 27883245	SP54
8.	At1g77860 (KOM)	-	chr01	29287572 - 29289352	SP54
9.	At2g29050 (AtRBL1)	-	chr02	12485223 - 12487449	SP54
10.	At2g41160	S	chr02	17163107 - 17165936	SP54-UBA
11.	At3g07950	S	chr03	2531858 - 2534510	SP54
12.	At3g17611	M	chr03	6024903 - 6026449	SP54-zfRan
13.	At3g53780 (AtRBL4)	-	chr03	19935546 - 19938004	SP54
14.	At3g56740	S	chr03	21026350 - 21029521	SP54-UBA
15.	At3g58460	-	chr03	21634160 - 21637420	SP54-UBA
16.	At3g59520	-	chr03	22002803 - 22004240	SP54
17.	At4g23070 (AtRBL7)	-	chr04	12090701 - 12092088	SP54
18.	At5g07250 (AtRBL3)	-	chr05	2273555 - 2275991	SP54
19.	At5g25752	M	chr05	8951267 - 8953353	SP54
20.	At5g38510	C	chr05	15435069 - 15437232	SP54
Peptidase_S59					

1.	At1g10390	C	chr01	3407025 - 3412844	SP59
2.	At1g59660	C	chr01	21928358 - 21933089	SP59
3.	At1g80680 (MOS3)	-	chr01	30328900 - 30333650	SP59

*: Subcellular locations for serine protease-like proteins in rice predicted using TargetP[19]. S: Secreted; C: Chloroplast; M:Mitochondria; -: Not predicted

#: Domain architectures observed in serine protease-like proteins identified in rice proteome.
SPxx- Serine protease family SPxx domain, where Sxx refers to the serine protease family as per MEROPS[5] classification (see text for details); PDZ– PDZ domain (Pfam[37] accession: PF00595; PA– Protease associated domain (Pfam[37] accession: PF02225); SN- Subtilisin N-terminal region (Pfam[37] accession: PF005922); D1034- Domain of unknown function (Pfam[37] accession: PF06280); S9N- Prolyl oligopeptidase, N-terminal beta-propeller domain (Pfam[37] accession: PF02897); PD40- WD40-like beta propeller repeat (Pfam[37] accession: PF07676); DPN- Dipeptidyl peptidase (DPP IV) N-terminal region (Pfam[37] accession: PF00930); ABC1- ABC1 family (Pfam[37] accession: PF03109); LON- ATP-dependent protease La (LON) domain (Pfam[37] accession: PF02190); AAA- ATPase family associated with various cellular activities (Pfam[37] accession: PF00004); UBA- UBA/TN-S domain (Pfam[37] accession: PF000627); zf-RanBP- Zinc finger in Ran binding protein and others (Pfam[37] accession: PF00641).
