# Supplementary material for: Cross genome comparisons of serine proteases in Arabidopsis and rice
Source: BMC Genomics. 2006 Aug 9;7:200. doi: 10.1186/1471-2164-7-200 (PMC1560137; doi:10.1186/1471-2164-7-200)
Supplement: Additional file 2 — Table S2. An inventory of rice serine protease-like proteins. An inventory of rice serine protease-like proteins identified by multifold approach (see methods for details). The list includes gene identifiers, predicted subcellular localization, chromosome location, chromosomal nucleotide position and domain architectures of serine proteases identified in current analysis [file 1471-2164-7-200-S2.pdf]

Table S2: An inventory of rice serine protease-like proteins identified by multifold approach (see methods for details)

S No.	Accession IDs	Subcellular location*	Chromosome location	Chromosomal nucleotide position	Domain architecture[#]
	Peptidase_S1				
1.	LOC_Os01g17070	-	chr01	9813579-9819508	SP1
2.	LOC_Os02g48180	-	chr02	29440576-29428572	SP1-PDZ
3.	LOC_Os02g50880	C	chr02	31046301-31041303	SP1
4.	LOC_Os03g62900	M	chr03	35528190-35524751	SP1
5.	LOC_Os04g38640	M	chr04	22578407-22584022	SP1-PDZ
6.	LOC_Os05g05480	C	chr05	2694326-2702362	SP1
7.	LOC_Os05g34460	C	chr05	20245089-20249133	SP1
8.	LOC_Os05g41810	C	chr05	24301942-24296583	SP1
9.	LOC_Os05g49380	C	chr05	28057054-28053696	SP1-PDZ
10.	LOC_Os06g12780	C	chr06	6979076-6986146	SP1
11.	LOC_Os08g04920	C	chr08	2508088-2502760	SP1
12.	LOC_Os11g14170	M	chr11	7898540-7905915	SP1-PDZ
13.	LOC_Os12g04740	-	chr12	2015390-2011885	SP1
14.	LOC_Os12g04750	-	chr12	2021827-2016494	SP1
15.	LOC_Os12g42210	C	chr12	26141895-26146391	SP1
	Peptidase_S8				
1.	LOC_Os01g17160	S	chr01	9859977-9867525	SN-SP8-PA-zf
2.	LOC_Os01g50680	S	chr01	29084547-29088397	SP8-PA
3.	LOC_Os01g52750	S	chr01	30328564-30323978	SN-SP8-PA
4.	LOC_Os01g56320	S	chr01	32430881-32435924	SN-SP8-PA
5.	LOC_Os01g58240	S	chr01	33639461-33642676	SN-SP8-PA
6.	LOC_Os01g58260	-	chr01	33647773-33652165	SN-SP8-PA
7.	LOC_Os01g58270	M	chr01	33654069-33659381	SN-SP8-PA
8.	LOC_Os01g58280	-	chr01	33662847-33667801	SN-SP8-PA
9.	LOC_Os01g58290	S	chr01	33671457-33676190	SN-SP8-PA
10.	LOC_Os01g64850	S	chr01	37626684-37628957	SN-SP8-PA
11.	LOC_Os01g64860	S	chr01	37631499-37634046	SN-SP8-PA
12.	LOC_Os02g10520	S	chr02	5537969-5531255	SN-SP8-PA
13.	LOC_Os02g16940	S	chr02	9657964-9651664	SN-SP8-PA
14.	LOC_Os02g17000	S	chr02	9716988-9713806	SN-SP8-PA
15.	LOC_Os02g17060	-	chr02	9763188-9758964	SP8-PA
16.	LOC_Os02g17080	S	chr02	9771266-9765760	SN-SP8-PA
17.	LOC_Os02g17090	S	chr02	9783134-9779479	SN-SP8-PA
18.	LOC_Os02g17150	-	chr02	9830181-9827336	SP8-PA
19.	LOC_Os02g44520	C	chr02	26898470-26915168	SP8
20.	LOC_Os02g44590	S	chr02	26963552-26966831	SN-SP8-PA
21.	LOC_Os02g53850	S	chr02	32926386-32928732	SN-SP8-PA

22.	LOC_Os02g53860	S	chr02	32932957-32930214	SN-SP8-PA
23.	LOC_Os02g53910	-	chr02	32955698-32953626	SP8-PA
24.	LOC_Os02g53970	S	chr02	32993115-32990503	SN-SP8-PA
25.	LOC_Os03g02750	S	chr03	1016356-1013668	SN-SP8-PA
26.	LOC_Os03g04950	S	chr03	2369497-2371848	SN-SP8-PA
27.	LOC_Os03g06290	-	chr03	3137994-3133190	SP8-PA
28.	LOC_Os03g13930	S	chr03	7544904-7542562	SN-SP8-PA
29.	LOC_Os03g31630	S	chr03	18017049-18019415	SN-SP8-PA
30.	LOC_Os03g40830	S	chr03	22662506-22665022	SN-SP8-PA
31.	LOC_Os03g55350	S	chr03	31432053-31429194	SN-SP8-PA
32.	LOC_Os04g02960	C	chr04	1171479-1184226	Ex2-SP8-zf-rve
33.	LOC_Os04g02980	-	chr04	1196627-1206156	SN-SP8-PA
34.	LOC_Os04g03060	C	chr04	1241288-1247418	SP8-PA
35.	LOC_Os04g03100	S	chr04	1267078-1271986	SN-SP8-PA
36.	LOC_Os04g03710	-	chr04	1625973-1635158	SP8
37.	LOC_Os04g03800	S	chr04	1689195-1697126	SN-SP8-PA
38.	LOC_Os04g03810	S	chr04	1701791-1707259	SN-SP8-PA
39.	LOC_Os04g03850	S	chr04	1723750-1728127	SP8-PA
40.	LOC_Os04g10360	S	chr04	5416994-5414718	SN-SP8-PA
41.	LOC_Os04g35140	S	chr04	21014190-21017349	SN-SP8-PA
42.	LOC_Os04g45960	S	chr04	26845614-26849260	SN-SP8-PA
43.	LOC_Os04g47150	S	chr04	27620092-27622853	SN-SP8-PA
44.	LOC_Os04g47160	S	chr04	27626806-27624524	SN-SP8-PA
45.	LOC_Os04g48420	S	chr04	28504197-28501867	SN-SP8-PA
46.	LOC_Os05g30580	S	chr05	17532343-17534798	SN-SP8-PA
47.	LOC_Os05g36010	S	chr05	21139951-21137405	SN-SP8-PA
48.	LOC_Os06g06800	S	chr06	3203764-3211338	SP8-zf-rve
49.	LOC_Os06g06810	-	chr06	3212064-3216135	SP8
50.	LOC_Os06g40700	S	chr06	24213865-24223110	SN-SP8-PA-Arf-C2
51.	LOC_Os06g41880	M	chr06	25073354-25081347	SN-SP8-PA
52.	LOC_Os06g48650	S	chr06	29389594-29396241	SN-SP8-PA-D1034
53.	LOC_Os07g39020	S	chr07	23396515-23398959	SN-SP8-PA
54.	LOC_Os07g48650	S	chr07	29128580-29131180	SN-SP8-PA
55.	LOC_Os08g23740	S	chr08	14229839-14233606	SN-SP8-PA
56.	LOC_Os08g35090	S	chr08	21978260-21980696	SN-SP8-PA
57.	LOC_Os09g26920	S	chr09	16303448-16306220	SN-SP8-PA
58.	LOC_Os09g30250	-	chr09	18366516-18369186	SN-SP8-PA
59.	LOC_Os09g36110	S	chr09	20442020-20445330	SN-SP8-PA
60.	LOC_Os10g25450	S	chr10	12652431-12655613	SN-SP8-PA
61.	LOC_Os10g38080	S	chr10	19884287-19881747	SN-SP8-PA
62.	LOC_Os11g15520	-	chr11	8787476-8779246	SP8-PA
63.	LOC_Os12g23980	S	chr12	13626256-13623902	SN-SP8-PA
	Peptidase_S9				
1.	LOC_Os01g01830	-	chr01	438108-446043	S9N-SP9

2.	LOC_Os01g42690	C	chr01	24256884-24260436	SP9
3.	LOC_Os01g49510	M	chr01	28458941-28462476	SP9
4.	LOC_Os01g57770	-	chr01	33389377-33395003	SP9
5.	LOC_Os02g18850	-	chr02	11006120-11001174	DPN-SP9
6.	LOC_Os02g55330	-	chr02	33825620-33821765	SP9
7.	LOC_Os03g19410	M	chr03	10903506-10911075	SP9
8.	LOC_Os03g24450	-	chr03	13922956-13915390	SP9
9.	LOC_Os04g47360	-	chr04	27731653-27736499	SP9
10.	LOC_Os05g46210	-	chr05	26552739-26556138	SP9
11.	LOC_Os06g06770	C	chr06	3185463-3190478	SP9
12.	LOC_Os06g11180	C	chr06	5868597-5861189	SP9
13.	LOC_Os06g11190	C	chr06	5875410-5869108	SP9
14.	LOC_Os06g42730	C	chr06	25653946-25657802	SP9
15.	LOC_Os06g51410	M	chr06	31100909-31091896	S9N-SP9
16.	LOC_Os07g41730	S	chr07	25005528-25010886	SP9
17.	LOC_Os07g48970	C	chr07	29302048-29307671	SP9
18.	LOC_Os09g28040	M	chr09	16970889-16963970	S9N-SP9
19.	LOC_Os09g29950	-	chr09	18177653-18171296	SP9
20.	LOC_Os10g04620	C	chr10	2180472-2185695	SP9
21.	LOC_Os10g28020	C	chr10	14026913-14037230	PD40-SP9
22.	LOC_Os10g28030	-	chr10	14037671-14043081	SP9
23.	LOC_Os12g18860	-	chr12	10940985-10934302	SP9
	Peptidase_S10				
1.	LOC_Os01g06490	S	chr01	3051360-3053744	SP10
2.	LOC_Os01g11670	S	chr01	6292773-6294116	SP10
3.	LOC_Os01g22980	S	chr01	12901670-12897795	SP10
4.	LOC_Os01g43890	S	chr01	25124205-25125563	SP10
5.	LOC_Os01g61690	S	chr01	35671089-35667727	SP10
6.	LOC_Os02g02320	S	chr02	772120-767270	SP10
7.	LOC_Os02g26480	S	chr02	15503307-15494377	SP10
8.	LOC_Os02g42310	-	chr02	25392472-25395207	SP10
9.	LOC_Os02g46260	S	chr02	28157275-28162148	SP10
10.	LOC_Os02g55130	-	chr02	33717972-33721178	SP10
11.	LOC_Os03g09190	-	chr03	4771295-4774728	SP10
12.	LOC_Os03g26920	S	chr03	15383132-15385508	SP10
13.	LOC_Os03g26930	S	chr03	15388220-15391649	SP10
14.	LOC_Os03g27480	S	chr03	15755066-15751532	SP10
15.	LOC_Os03g27510	M	chr03	15772720-15767916	SP10
16.	LOC_Os03g27530	M	chr03	15784615-15777538	SP10
17.	LOC_Os03g27550	S	chr03	15789716-15789091	SP10
18.	LOC_Os03g27590	C	chr03	15816908-15811468	SP10
19.	LOC_Os03g52040	M	chr03	29811733-29817416	SP10
20.	LOC_Os03g52070	M	chr03	29823911-29828592	SP10
21.	LOC_Os03g52080	M	chr03	29833771-29840308	SP10

22.	LOC_Os04g09720	S	chr04	5034168-5027622	SP10
23.	LOC_Os04g25560	S	chr04	14638040-14632981	SP10
24.	LOC_Os04g32540	-	chr04	19245848-19249190	SP10
25.	LOC_Os04g44410	S	chr04	25915347-25910742	SP10
26.	LOC_Os05g06660	M	chr05	3412402-3407843	SP10
27.	LOC_Os05g18610	S	chr05	10657189-10655949	SP10
28.	LOC_Os05g18630	M	chr05	10670762-10667439	SP10
29.	LOC_Os05g50570	S	chr05	28734539-28735879	SP10
30.	LOC_Os05g50580	S	chr05	28738704-28740059	SP10
31.	LOC_Os05g50600	S	chr05	28742638-28743966	SP10
32.	LOC_Os06g08720	S	chr06	4352378-4346160	SP10
33.	LOC_Os06g13410	S	chr06	7375933-7375287	SP10
34.	LOC_Os06g13420	-	chr06	7379891-7377699	SP10
35.	LOC_Os06g32740	M	chr06	19038412-19042904	SP10-TP21
36.	LOC_Os06g32780	-	chr06	19060064-19065219	SP10
37.	LOC_Os06g36570	C	chr06	21483520-21482765	SP10
38.	LOC_Os06g51370	S	chr06	31064441-31066448	SP10
39.	LOC_Os07g29620	S	chr07	17412155-17408334	SP10
40.	LOC_Os07g46350	S	chr07	27642075-27637160	SP10
41.	LOC_Os08g44640	S	chr08	27936461-27941793	SP10
42.	LOC_Os09g28830	S	chr09	17456817-17453093	SP10
43.	LOC_Os09g28840	-	chr09	17465186-17460336	SP10
44.	LOC_Os10g01110	M	chr10	82403-79467	SP10
45.	LOC_Os10g01130	-	chr10	91509-89395	SP10
46.	LOC_Os10g01140	S	chr10	95442-95050	SP10
47.	LOC_Os10g39560	-	chr10	20638865-20635789	SP10
48.	LOC_Os11g10750	S	chr11	5891351-5887351	SP10
49.	LOC_Os11g24180	-	chr11	13277262-13272014	SP10
50.	LOC_Os11g24200	S	chr11	13286632-13284630	SP10
51.	LOC_Os11g24290	S	chr11	13342469-13338442	SP10
52.	LOC_Os11g24320	-	chr11	13357680-13353961	SP10
53.	LOC_Os11g24340	S	chr11	13370360-13365693	SP10
54.	LOC_Os11g24370	-	chr11	13397295-13394524	SP10
55.	LOC_Os11g24410	S	chr11	13416731-13413373	SP10
56.	LOC_Os11g24510	S	chr11	13480850-13472958	SP10
57.	LOC_Os11g27170	M	chr11	15141602-15147766	SP10-Retrotrans
58.	LOC_Os11g27200	-	chr11	15155954-15160912	SP10
59.	LOC_Os11g27260	M	chr11	15187092-15190205	SP10
60.	LOC_Os11g27270	-	chr11	15196239-15198815	SP10
61.	LOC_Os11g27320	-	chr11	15215131-15217831	SP10
62.	LOC_Os11g27350	-	chr11	15236511-15241893	SP10
63.	LOC_Os11g31980	S	chr11	18325227-18329071	SP10
64.	LOC_Os11g42390	M	chr11	24965544-24971675	SP10
65.	LOC_Os12g15470	S	chr12	8833520-8828475	SP10
66.	LOC_Os12g39170	S	chr12	24076535-24070416	SP10

	Peptidase_S12				
1.	LOC_Os06g48770	M	chr06	29479186-29471793	ABC1-SP12
	Peptidase_S14				
1.	LOC_Os01g16530	C	chr01	9379941-9375744	SP14
2.	LOC_Os01g32350	C	chr01	17726025-17731752	SP14
3.	LOC_Os02g42290	C	chr02	25376439-25378863	SP14
4.	LOC_Os03g19510	C	chr03	10954941-10951972	SP14
5.	LOC_Os03g22430	C	chr03	12847202-12842042	SP14
6.	LOC_Os03g29810	C	chr03	16958307-16961760	SP14
7.	LOC_Os04g44400	C	chr04	25907642-25908862	SP14
8.	LOC_Os05g51450	C	chr05	29237881-29234239	SP14
9.	LOC_Os06g04530	C	chr06	1948450-1952326	SP14
10.	LOC_Os08g15270	M	chr08	9269071-9269418	SP14
11.	LOC_Os10g43050	C	chr10	22682237-22684378	SP14
12.	LOC_Os11g11210	C	chr11	6193268-6189735	SP14
13.	LOC_Os12g10590	-	chr12	5644637-5643889	SP14
	Peptidase_S16				
1.	LOC_Os03g19350	C	chr03	10884163-10872890	LON-AAA-SP16
2	LOC_Os06g05820	C	chr06	2651070-2660303	AAA-SP16
3.	LOC_Os07g48960	C	chr07	29301026-29291022	LON-AAA-SP16
4.	LOC_Os09g36300	C	chr09	20600328-20593969	LON-AAA-SP16
	Peptidase_S26				
1.	LOC_Os02g58140	S	chr02	35523210-35521470	SP26
2.	LOC_Os03g55640	M	chr03	31626264-31623333	SP26
3.	LOC_Os04g08340	M	chr04	4425111-4427765	SP26
4.	LOC_Os05g23260	M	chr05	13100862-13108531	SP26
5.	LOC_Os06g16260	-	chr06	9261241-9255962	SP26
6.	LOC_Os09g28000	M	chr09	16952405-16949751	SP26
7.	LOC_Os11g40500	M	chr11	23625495-23622304	SP26
	Peptidase_S28				
1.	LOC_Os01g56150	M	chr01	32317717-32314344	SP28
2.	LOC_Os06g43930	S	chr06	26417010-26411495	SP28
3.	LOC_Os10g36760	S	chr10	19169290-19174570	SP28
4.	LOC_Os10g36780	M	chr10	19179375-19184288	SP28
5.	LOC_Os11g05760	S	chr11	2638093-2632684	SP28
	Peptidase_S41				
1.	LOC_Os01g47450	M	chr01	27095326-27099780	SP41
2.	LOC_Os02g57060	M	chr02	34883220-34886513	SP41
3.	LOC_Os06g21380	C	chr06	12344921-12349134	SP41

	Peptidase_S49				
1.	LOC_Os02g49570	M	chr02	30251251-30242321	SP49
	Peptidase_S54				
1.	LOC_Os01g05430	-	chr01	2563506-2566252	SP54
2.	LOC_Os01g16330	M	chr01	9256209-9260637	SP54-UBA
3.	LOC_Os01g18100	-	chr01	10136833-10134126	SP54
4.	LOC_Os01g55740	M	chr01	32074067-32077954	SP54
5.	LOC_Os01g67040	-	chr01	38908306-38909673	SP54
6.	LOC_Os02g22100	-	chr02	13157662-13164618	SP54
7.	LOC_Os03g02530	-	chr03	911926-914460	SP54
8.	LOC_Os03g24390	-	chr03	13875589-13871510	SP54
9.	LOC_Os03g44830	-	chr03	25244972-25238253	SP54-UBA
10.	LOC_Os04g01300	-	chr04	220402-217779	SP54
11.	LOC_Os04g48130	-	chr04	28266232-28267975	SP54
12.	LOC_Os05g13370	C	chr05	7302412-7298240	SP54
13.	LOC_Os07g46170	-	chr07	27549204-27553491	SP54
14.	LOC_Os08g43320	-	chr08	27246100-27243213	SP54
15.	LOC_Os09g28100	C	chr09	17006593-17009062	SP54
16.	LOC_Os09g35730	-	chr09	20200512-20197629	SP54
17.	LOC_Os10g37760	-	chr10	19699448-19701639	SP54
18.	LOC_Os11g47840	-	chr11	28211146-28207320	SP54
	Peptidase_S59				
1.	LOC_ Os03g07580	-	chr03	3841519-3845391	SP59
2.	LOC_ Os12g06870	C	chr12	3337346-3343201	SP59
3.	LOC_ Os12g06890	-	chr12	3347749-3351986	SP59

*: Subcellular locations for serine protease-like proteins in rice predicted using TargetP[19]. S: Secreted; C: Chloroplast; M:Mitochondria; -: Not predicted

#: Domain architectures observed in serine protease-like proteins identified in rice proteome.
SPxx- Serine protease family SPxx domain, where SPxx refers to the serine protease family as per MEROPS[5] classification (see text for details); PDZ– PDZ domain (Pfam[37] accession: PF00595; PA– Protease associated domain (Pfam[37] accession: PF02225); SN- Subtilisin N-terminal region (Pfam[37] accession: PF005922); D1034- Domain of unknown function (Pfam[37] accession: PF06280); Arf- ADP-ribosylation factor family (Pfam[37] accession: PF00025); C2- C2 domain (Pfam[37] accession: PF00168); zf- Zinc knuckle (Pfam[37] accession: PF00098); rve– Integrase core domain (Pfam[37] accession:PF00665); EX2- Extensin-like region (Pfam[37] accession: PF04554); S9N- Prolyl oligopeptidase, N-terminal beta-propeller domain (Pfam[37] accession: PF02897); PD40- WD40-like beta propeller repeat (Pfam[37] accession: PF07676); DPN- Dipeptidyl peptidase (DPP IV) N-terminal region (Pfam[37] accession: PF00930); Transposase_21- Transposase family tnp2 (Pfam[37] accession: PF02992); Retrotrans-Retrotransposon gag protein (Pfam[37] accession: PF03732); ABC1- ABC1 family (Pfam[37] accession:

PF03109); LON- ATP-dependent protease La (LON) domain (Pfam[37] accession: PF02190); AAA- ATPase family associated with various cellular activities (Pfam[37] accession: PF00004); UBA- UBA/TN-S domain (Pfam[37] accession: PF000627)
