# Supplementary material for: Cross genome comparisons of serine proteases in Arabidopsis and rice
Source: BMC Genomics. 2006 Aug 9;7:200. doi: 10.1186/1471-2164-7-200 (PMC1560137; doi:10.1186/1471-2164-7-200)
Supplement: Additional file 3 — Table S3. Background information on serine proteases. Additional literature information on serine protease families taken up for study in current analysis. The information is categorized into three parts namely a brief structural overview, enzyme characteristics and functional information where known. Additional references for the material contained in the file have been provided at the end. [file 1471-2164-7-200-S3.pdf]

Table S3: Additional background information about Serine protease families identified in *Arabidopsis* and rice.

S. No.	Serine protease family	Structural information in brief	Enzyme characteristics	Functional information
1.	DegP protease (S1)	Crystal structure of *E. coli* DegP shows that it is a hexamer formed by two staggered trimeric rings. The proteolytic sites are located in a central cavity and retain twelve PDZ domains – two for each monomer- form the side walls and probably act as gatekeepers of the inner chamber. The proteolytic site is inaccessible at low temperatures and thus, enzyme exists in chaperone conformation[22]		*E. coli* DegP is a heat-shock protein essential for cell viability at higher temperatures and responsible for degradation of misfolded proteins. Bacterial DegP proteases have been implicated in thermal, osmotic and pH tolerance, H_2O_2 resistance and bacterial virulence, while in humans they are believed to be involved in arthiritis, cell growth, unfolded stress response, programmed cell death and aging[1, 2].
2.	Subtilisin (S8)	Crystal structures have been determined for many members of the subtilisin family and they are shown to utilize a highly conserved catalytic triad similar to the members of chymotrypsin and carboxypeptidase clans but have a different order of Asp, His and Ser residues in the sequence (D137, H168, S325) arranged in a α/β protein scaffold and the structures show no other similarity[33]	A majority of subtilisins are synthesized as pre-pro enzymes that are activated subsequent to transfer across the membrane and cleavage of the pro-peptide. Most members of the family are active at neutral to mildly alkaline pH and are many are thermostable due to the presence of two calcium binding sites[33].<br>2. Most members of the family are non-specific proteases exhibiting	Subtilisins have been implicated in diverse processes such as pathogen defense, stomata and leaf development, lateral root emergence, xylem differentiation, stress response, programmed cell death, nodulation and

			broad substrate specificity, with a preference to cleave after hydrophobic residues, but some members of S8B subfamily cleave after paired basic amino acids[33].<br>2. Most of the plant subtilisins are highly abundant proteins of the secretory pathway, although some are predicted to be cytoplasmic or localize to organelles. The PA domain is about 170-210 amino acids long and has a Beta-sandwich structure with two peripheral helices and has been observed in association with some peptidase and receptor families[34, 36].	nitrogen fixation, microsporogenesis, seed development etc[3-6]. However, their exact functions in these pathways remain unclear, mainly due to lack of positive identification of their physiological substrates. Such diversity in proposed function suggests that plant subtilisins, in addition to degradation, may play significant roles in processing of precursor proteins in various signal transduction pathways and may have been recruited for functions in plant secondary metabolism[4-7].
3.	Prolyl Oligopeptidase family (S9)	Crystal structures reveal that these enzymes consist of a peptidase domain, with a α/β hydrolase fold and its catalytic triad (S554, D641, H680) is covered by the central tunnel of a β-	Most members of the family cleave peptide bonds towards C-terminal side of prolyl residues, some like oligopeptidase B, prefer arginine and lysine	

		propeller domain that acts as a gating filter that excludes large peptides and proteins from the central cavity containing the catalytic triad, preventing their proteolysis in the cytosol[SR1, SR2].	residues for cleavage. However, all POPs are synthesized as active enzymes and they cleave only substrates containing less than 30 amino acids. The enzymes are synthesized as active peptidases and lack any zymogen or proenzyme forms[38-40].	
4.	Serine carboxypeptidases (S10)	Crystal structures show that serine carboxypeptidases belong to the α/β hydrolase fold and possess a catalytic triad similar to members of chymotrypsin and subtilisin families in the order Ser, Asp and His (S257, D449, H508)[5].	Serine carboxypeptidases catalyze the hydrolysis of the C-terminal bond in proteins and peptides and unlike most other serine proteases, are usually active only at acidic pH. 2. Most serine carboxypeptidases are synthesized as preproenzymes[5].	Plant SCPLs have been implicated in diverse processes such as mobilization of protein reserves during seed germination, programmed cell death, brassinosteroid signaling, seed development, stress response, herbicide metabolism etc., which have been attributed to their broad substrate specificity[2, 9, 41, 42]. However, like plant subtilisins, their physiological substrates remain largely unidentified and their exact functions remain unclear. The discovery of plant SCP-like enzymes that facilitate a transesterification reaction rather than proteolysis and thereby function as acyltransferases, suggests that over the course of evolution,

				some plant SCPLs may have been recruited for non-proteolytic functions in various biochemical pathways, particularly in plant secondary metabolism[8-11]. Plant SCP-like acyltransferases appear to be indistinguishable from SCPLs at the sequence level and therefore are proposed to be employed for transcacylation, where the acyl-enzyme cleavage is facilitated by a second substrate (L-malate in glucose-ester dependent acyltransferases) instead of water[9, 10]. It has been proposed that the α/β hydrolase fold that forms the core of all SCPLs and related families is responsible for catalytic diversity including the potential for transpeptidation and transesterification in enzymes sharing this structural feature[SR3, SR4, 11].
5.	Serine beta-lacatamases	Tertiary structures have been determined for several	The active site Ser and Lys residues form a	

	(S12)	members of the family and show striking structural similarities. They comprise an all-α and an α/β domain, with the active site situated at the interface of the two domains[46-47].	sequentially conserved motif that is highly conserved across family members. The Tyr active site residue occurs in a conserved Y-x-N motif situated on a loop in the all α-domain, with Tyr residue being replaced by Ser in some proteins[46-48]	
6.	Clp proteases (S14)	1. The barrel like structure of Clp proteases is formed by two heptameric rings and results in a single cavity of ~50 Å that encloses the catalytic triad residues Ser-His-Asp (S111, H136, D185). This allows for degradation of small peptides but precludes the entry of large folded polypeptides[6, 7, 49]<br>2. The ATPase subunit forms a single hexameric ring that associates with either or both ends of ClpP. It acts as the chaperone regulatory subunit, confers substrate specificity and is responsible for the delivery of the unfolded protein into the proteolytic active site via ATP hydrolysis where it is rapidly degraded into fragments that later diffuse out [6, 7, 50]. ClpA and ClpX possess distinct	1. According to MEROPS[5], Clp proteases do not show any strict specificity for the residues at P1 or P1' positions in their substrates, but seem to prefer hydrophobic or non-polar residues at these positions.<br>2. Clp proteases are believed to play a role in degaradation of misfolded nascent peptides. These abnormal peptides are tagged with a unique C-terminal sequence, which targets them for degradation by Clp proteases. In certain cases, proteins are tagged with hydrophobic amino acids at the N-terminus to facilitate their recognition and degradation by Clp proteases[50]	

		substrate specificities [8-10].		
7.	Lon Proteases (S16)	Crystal structure of the proteolytic (P) domain of *E. coli* Lon protease reveals a novel fold consisting of 6 α helices and 10 β strands with monomers forming a hexameric ring. The catalytic residues Ser679 and Lys722 lie within a shallow concavity towards the distal end of the hexamer, which is connected to the proximal surface by a solvent accessible central pore ~32 Å long and ~18 Å in diameter, which might serve a gating function for substrate entry [SR5]	Lon proteases display broad sequence specificity in degrading polypeptides, though they seem to have a slight preference for hydrophobic residues at P1 position[5]	
8.	Signal Peptidases (S26)	Crystal structure of catalytically active soluble fragment of *E. coli* SPase in complex with β-lactam covalently bound as an acyl-coenzyme intermediate to the γ-oxygen of Ser90, demonstrating its role as nucleophile in hydrolytic mechanism of signal peptide cleavage. The structure suggests that Lys145 is employed as a general base in activation of Ser90, explaining the specificity requirement at the signal-peptide cleavage site, and reveals a large exposed hydrophobic surface which could be a site for membrane association [SR6]	Site directed mutagenesis studies have shown that the residues at -1 and -3 position relative to the cleavage site in the C-region (extracytoplasmic region) of the signal peptide are essential for efficient recognition and cleavage of signal peptides by Type I SPases. Subsequent analysis revealed a preference for Ala-X-Ala motif in a majority of secreted proteins, though a Val-X-Ala motif was	

			also found in a significant number of proteins recognized for signal peptide cleavage by the Type I SPases, suggesting a key role for Ala residue at -1 position for Type I SPase mediated signal-peptide cleavage[57, 58].	
9.	Lysosomal Pro-X Carboxypeptidase family family (S28)		Experimental studies have shown that members of this family are exopeptidases that cleave at the C-terminus to the penultimate Proline residue and are mostly active at acidic pH. PCP, however, has been shown to retain enzymatic activity with some substrates at neutral pH. Some members of the family such as PCP are synthesized as preproteins[5].	In human cells, lysosomal Pro-X carboxypeptidase (PCP) functions to regulate the activities of endothelial cell Prekallikrein (PK) and Angiotensin II that are important constituent of two antagonistic systems that regulate blood pressure. In mouse, the members of this family Tssp and Teap are proposed to play a role in thymus function and thymocyte maturation. Dipeptidyl peptidase has been implicated in hydrolysis of dipeptidyl derivatives[4, 62, 63].

	C-terminal processing peptidase family (S41)		1. *E. coli* Tsp is a periplasmic endoprotease that selectively degrades substrates with non-polar C-terminal sequences in some cytoplasmic proteins[64].<br>2. Members of this family recognize a tripeptide sequence, Xaaa-Yaaa-Zaaa in the C-termini of their substrates, where Xaa is usually Ala or Leu, Yaa preferably Ala or Tyr and Zaa mostly Ala. However, a free C-terminal carboxyl group seems essential for substrate recognition since proteins with amidated group are not cleaved by these peptidases[64].	
	SppA/Protease IV family (S49)		Protease IV was found to initiate the degradation of signal peptide, by endoproteolytic cleavage in hydrophobic segment, followed by subsequent degradation of the signal peptide by a metalloprotease oligopeptidase A and other cytoplasmic enzymes[67, 57].	
	Rhomboids (S54)		1. All known rhomboids have multiple transmembrane domains, usually six or seven and are thought to function by forming a charge relay mechanism, typical of	1. Rhomboids were first identified in *Drosophila* genetic screens where mutants had rhomboid-shaped heads and a protein Rhomboid-1 (Rho-1) was subsequently identified as activator

			many serine protease familie[69, 70].<br>2. Rhomboids seem to show a clear distinction form other intramembrane proteases in several aspects. Firstly, rhomboids except perhaps the mitochondrial members do not appear to require substrate cleavage prior to catalysis unlike other intramembrane proteases[69, 70]. Secondly, rhomboids do not function as a part of multiprotein complex unlike other intramembrane proteases such as presenilin[69, 70]. Thirdly, all rhomboids studied so far release protein fragments in the extra cellular or lumenal space unlike most intramembrane proteases[69, 70].	of epidermal growth factor (EGF) receptor signaling in *Drosophila*[72].<br>2. Rhomboids have been identified in nearly all the sequenced genomes and their near ubiquity is quite unlike other membrane proteins. However, phylogenetic analysis suggests that they are unlikely to have existed in the last common ancestor of extant organisms. On the contrary, they have been proposed to have evolved in bacteria and subsequently spread to other lineages through multiple horizontal gene transfer events[71].
	Nucleoporin autopetidase family (S59)	The three dimensional structure of the C-terminal domain of Nup98, which is	An evolutionarily conserved Serine residue (S881) appears to be	

		involved in RNA export, reveals a novel protein fold, and thus a new class of self-catalyzed proteases. The C-terminal domain of Nup98 was found to adopt a half-open, β sandwich-like fold anchored by a large β-sheet that expands across the entire length of the domain[78].	essential for cleavage and a Histidine residue (H879) proximal to the catalytic Ser residue appears to play an important role in cleavage, via hydrogen bonding to S881, for generating an active nucleophile. In addition, a conserved Lysine residue, Lys791, that appears to be important for stabilizing the oxyanion intermediate and the active site geometry have also been implicated in autocatalysis[78].	

Additional References

SR1. Fulop V, Bocskei Z, Polgar L: **Prolyl oligopeptidase: an unusual beta-propeller domain regulates proteolysis**. *Cell* 1998, **94**(2):161-170.

SR2. Fulop V, Szeltner Z, Polgar L: **Catalysis of serine oligopeptidases is controlled by a gating filter mechanism**. *EMBO Rep* 2000, **1**(3):277-281.

SR3. Ollis DL, Cheah E, Cygler M, Dijkstra B, Frolow F, Franken SM, Harel M, Remington SJ, Silman I, Schrag J *et al*: **The alpha/beta hydrolase fold**. *Protein Eng* 1992, **5**(3):197-211.

SR4. Holmquist M: **Alpha/Beta-hydrolase fold enzymes: structures, functions and mechanisms**. *Curr Protein Pept Sci* 2000, **1**(2):209-235.

SR5. Botos I, Melnikov EE, Cherry S, Tropea JE, Khalatova AG, Rasulova F, Dauter Z, Maurizi MR, Rotanova TV, Wlodawer A *et al*: **The catalytic domain of Escherichia coli Lon protease has a unique fold and a Ser-Lys dyad in the active site**. *J Biol Chem* 2004, **279**(9):8140-8148.

SR6. Paetzel M, Dalbey RE, Strynadka NC: **Crystal structure of a bacterial signal peptidase apoenzyme: implications for signal peptide binding and the Ser-Lys dyad mechanism**. *J Biol Chem* 2002, **277**(11):9512-9519.
