# Supplementary material for: Cross genome comparisons of serine proteases in Arabidopsis and rice
Source: BMC Genomics. 2006 Aug 9;7:200. doi: 10.1186/1471-2164-7-200 (PMC1560137; doi:10.1186/1471-2164-7-200)
Supplement: Additional file 4 — Table S4. Orthologous serine protease-like proteins identified in the two plant species. A list of Arabidopsis thaliana serine protease-like proteins and their putative orthologues in rice genome (see methods for details). Functional information gathered from literature has been provided for Arabidopsis gene products where possible [file 1471-2164-7-200-S4.pdf]

Table S4: List of *Arabidopsis thaliana* serine protease-like proteins and their putative orthologs in rice genome

S. No.	Serine protease-like proteins in *Arabidopsis thaliana*	Biological function (if known)	Putative ortholog in rice genome
Peptidase_S1 (DegP protease family)			
1.	At1g28320		LOC_Os05g41810
2.	At3g03380		LOC_Os02g48180
3.	At3g27925 (DegP1)	Thylakoid associated serine protease upregulated in response to elevated temperatures[29, 30]	LOC_Os05g49380
4.	At4g18370		LOC_Os12g42210
5.	At5g27660		LOC_Os11g14170
6.	At5g36950		LOC_Os05g34460
7.	At5g40200		LOC_Os02g50880
Peptidase_S8 (Subtilisin family)			
1.	At1g04110		LOC_Os03g04950
2.	At2g04160 (AIR3)	Possibly involved in lateral root emergence[12]	LOC_Os02g10520
3.	At2g05920		LOC_Os10g25450
4.	At3g14067		LOC_Os02g53860
5.	At3g14240		LOC_Os08g55090
6.	At4g10550		LOC_Os09g36110
7.	At4g20430		LOC_Os01g56320
8.	At4g20850		LOC_Os02g44520
9.	At4g30020		LOC_Os03g13930
10.	At5g19660		LOC_Os06g06810
11.	At5g45640		LOC_Os01g52750
12.	At5g51750		LOC_Os04g48420
13.	At5g67090		LOC_Os05g36010
14.	At5g67360 (ARA12)	Upregulated in response to jasmonic acid, possible role in cell wall metabolism[12]	LOC_Os03g55350
Peptidase_S9 (Prolyl oligopeptidase family)			
1.	At1g50380		LOC_Os06g51410
2.	At1g69020		LOC_Os09g29950
3.	At1g76140		LOC_Os01g01830
4.	At2g24320		LOC_Os02g55330

5.	At2g47390		LOC_Os07g48970
6.	At3g23540		LOC_Os03g24450
7.	At4g14570		LOC_Os10g28020
8.	At4g24760		LOC_Os12g18860
9.	At5g20520		LOC_Os07g41730
10.	At5g24620		LOC_Os02g18850
11.	At5g25770		LOC_Os06g06770
12.	At5g36210		LOC_Os06g11180
13.	At5g66960		LOC_Os09g28040
Peptidase_S10 (Serine carboxypeptidase family)			
1.	At1g15000		LOC_Os05g50600
2.	At1g28110		LOC_Os11g31980
3.	At5g42240		LOC_Os11g10750
4.	At1g73270		LOC_Os10g01110
5.	At2g27920		LOC_Os03g27590
6.	At3g07990		LOC_Os01g61690
7.	At3g10410		LOC_Os02g02320
8.	At3g17180		LOC_Os05g06660
9.	At3g63470		LOC_Os07g46350
10.	At3g63470		LOC_Os07g46350
11.	At4g12190		LOC_Os12g15470
12.	At4g30610		LOC_Os02g55130
13.	At4g30810		LOC_Os06g08720
14.	At5g08260		LOC_Os04g44410
15.	At5g23210		LOC_Os02g42310
16.	At5g42240		LOC_Os11g10750
Peptidase_S12 (Serine beta-lactamase family)			
1.	At5g24810		LOC_Os06g48770
Peptidase_S14 (Clp protease family)			
1.	At1g02560 (NclpP1)	Involved in the formation of a 350-kDa complex in *Arabidopsis* chloroplasts, believed to play a role in degradation of misfolded or unassembled peptides in an ATP-dependent manner[51]	LOC_Os03g19510
2.	At1g09130		LOC_Os03g22430
3.	At1g11750		LOC_Os03g29810
4.	At1g12410 (NclpP2)		LOC_Os06g04530
5.	At1g49970 (NclpP5)		LOC_Os05g51450
6.	At1g66670 (NclpP3)		LOC_Os01g32350
7.	At4g17040		LOC_Os01g16530
8.	At5g23140		LOC_Os04g44400
9.	At5g45390 (NclpP4)		LOC_Os10g43050
Peptidase_S16 (Lon protease family)			
1.	At5g05780		LOC_Os03g19350
2.	At5g26860 (Lon1)	Possible role in	LOC_Os07g48960

		cytoplasmic male sterility[54]	
3.	At5g47060		LOC_Os09g36300
Peptidase_S26 (Signal peptidase family)			
1.	At1g06870		LOC_Os09g28000
2.	At1g52600		LOC_Os06g16260
3.	At1g53530		LOC_Os11g40500
4.	At2g30440 (TPP)	Possibly involved in processing of proteins during their transport across photosynthetic membrane[60]	LOC_Os03g55640
5.	At3g08980		LOC_Os04g08340
Peptidase_S28 (Lysosomal Pro-x Carboxypeptidase family)			
1.	At2g24280		LOC_Os06g43930
2.	At4g36195		LOC_Os10g36760
3.	At5g65760		LOC_Os01g56150
Peptidase_S41 (C-terminal processing peptidase family)			
1.	At3g57680		LOC_Os06g21380
2.	At4g17740		LOC_Os02g57060
3.	At5g46390		LOC_Os01g47450
Peptidase_S49 (Protease IV family)			
1.	At1g73990 (SppA)	Possibly involved in light dependent degradation of antenna and photosystem II complexes [67]	LOC_Os02g49570
Peptidase_S54 (Rhomboid family)			
1.	At1g18600		LOC_Os01g55740
2.	At1g25290		LOC_Os09g28100
3.	At1g63120 (AtRBL2)		LOC_Os03g02530
4.	At2g29050 (AtRBL1)		LOC_Os11g47840
5.	At3g17611		LOC_Os01g18100
6.	At3g58460		LOC_Os03g44830
7.	At3g59520		LOC_Os01g67040
8.	At5g07250		LOC_Os09g35730
9.	At5g25752		LOC_Os05g13370
10.	At5g38510		LOC_Os02g22100
Peptidase_S59 (Nucleoporin2 autopeptidase family)			
1.	At1g10390		LOC_Os12g06870

2.	At1g80680 (MOS3)	Involved in activation of disease resistance response against bacterial pathogen [77]	LOC_Os03g07580
