# Supplementary material for: Cross genome comparisons of serine proteases in Arabidopsis and rice
Source: BMC Genomics. 2006 Aug 9;7:200. doi: 10.1186/1471-2164-7-200 (PMC1560137; doi:10.1186/1471-2164-7-200)
Supplement: Additional file 5 — Figure SF1. Multiple sequence alignment and phylogenetic analysis of Arabidopsis and rice DegP protease-like proteins. A: Multiple sequence alignment of Arabidopsis and rice DegP protease-like proteins. Multiple sequence alignment of the DegP domain region of the annotated Arabidopsis and rice DegP protease-like proteins. The catalytic triad residues are indicated. Gene names correspond to those in Additional files 1 and 2. For brevity, rice gene names have been shortened to OsXXg##### instead of LOC_OsXXg#####, XX referring to chromosome 1–12 and a 5 digit number assigned to each gene. B: Phylogenetic analysis of Arabidopsis and rice DegP protease-like proteins. Unrooted N-J tree computed from multiple sequence alignments of Arabidopsis (red) and rice (blue) DegP protease domains. DegP protease-like protease domains were aligned using ClustalW[95] program and the alignments were exported to Phylip package[96] for representing the Neighbor-Joining tree (see methods). The colors and circles represent different evolutionary clades identified in the analysis (see text for details). Clade I is represented in orange, Clade II is shaded green while Clade III is labelled in purple. For clarity, bootstrap values were replaced with symbols representing bootstrap percentages >50%. Bootstrap values between 50–60% are represented by an asterix, circles represent bootstrap values from 60%–80% while bootstrap values >80% are represented by rectangles. Gene names correspond to those listed in Tables 2 and 3. For brevity, rice gene names have been shortened to OsXXg##### instead of LOC_OsXXg#####, XX referring to chromosome 1–12 and a 5 digit number assigned to each gene. [file 1471-2164-7-200-S5.pdf]

a)

```
                                                 :*    :
At1g28320  ----------QVAIEKAMESVCLITVNDGVWASGIILNE----HGLILTNAHLLEPWRYQTG---HRDIRVRLCHLDSWT
Os05g41810 --------DVSPSLTEAISSVVLVTVSETSWASGIILNK----NGLIMTNAHLLEPWRFKK----DRAISVRLDHGERKT
At3g27925  --------------------TLDVLEVPQGSGSGFVWDK----QGHIVTNYHVIR-------------GASDLRVTLADQ
Os05g49380 --------------------TLDVLEVPQGSGSGFVWDK----SGHIVTNFHVIR-------------GASDLRVTLADQ
At5g39830  --------------------MTGVVEIPEGNGSGVVWDG----QGYIVTNYHVIGNALSRNPSPGDVVGRVNILASDGVQ
Os04g38640 --------------------VTGVVEIPEGNGSGVVWDD----SGHIVTNYHVVGNALSKKPKPGEVVARVNILAADGIQ
At4g18370  ---------------SSGDILTDEENGKIEGTGSGFVWDK----LGHIVTNYHVIAKLATDQF--GLQRCKVSLVDAKGTR
Os12g42210 --VVGRTPGRGGGQAVEAEDGEEGGATVEGTGSGFVWDT----AGHIVTNYHVVAKLAGDGS--AFHRCKVLLEDSSGNS
At5g27660  TIANAAARIGPAVVNLSVPQGFHGISMGKSIGSGTIIDA----DGTILTCAHVVVDFQNIRHS-----SKGRVDVTLQDG
Os11g14170 ------------VVNISSTQETHGWVLEKSIGSGTIIDP----DGTILTCAHVVLDFQSTKPI-----LRGKVSVTLQDG
At5g36950  -------KIFTVSTSPSYFLPWQNKSQRESMGSGFVIS-----GRKIITNAHVVA------------DHSFVLVRKHGSS
Os05g34460 -------KVFTVSSSPNYFLPWQNKAQRESMGSGFVIP-----GRRIITNAHVIA------------DHTFVLVRKHGSP
At5g40560  ----------------------------------------------IITNAHVVA------------NHILVLVIKRGSP
At1g65640  -------KVFTVYSMPSVLQPWRNWPQQESGGSGFVIS-----GKKILTNAHVVA------------DHIFLQVRKHGSP
At1g65630  -------KVFTVSSKPRLFQPWQITMQSESTGSGFVIS-----GKKILTNAHVVA------------NQTSVKVRKHGST
At3g16540  ------VKVFSNSTEYSKSKPWKTLDQKSSRGTGFAIA-----GRKILTNAHVVMAMN----------DHTFVDVKRHGSQ
At3g16550  -------EVFTDSTKYSKVKPWQTLNQESYGGSGFAIA-----GKKILTNAHVVEGMN----------DHIFVHVKRHGSQ
At2g47940  --------VYCTHTAPDYSLPWQKQRQFTSTGSAFMIG-----DGKLLTNAHCVEH------------DTQVKVKRRGDD
Os05g05480 --------VYCTHIAPDYGLPWQKQRQHASTGSAFMIG-----DGKLLTNAHCVEH------------DTQVKVKRRGDD
Os02g50880 --------VFCVHTEPNFSLPWQRKRQYSSSSSGFIIG-----GRRVLTNAHSVEH------------YTQVKLKKRGSD
Os06g12780 --------VFCVHTEPNFSLPWQRKRQYSSSSSGFIIG-----GHRVLTNAHSVEH------------YTQVKLKKRGSD
At5g40200  --------VFCVHTEPNFSLPWQRKRQYSSGSSGFIIG-----GRRVLTNAHSVEH------------HTQVKLKKRGSD
At1g51150  -------KIFSFSREPNVVQPWQTTEKEYS-SSGFAIS-----GRRILTNAHVVG------------DHLYLQVRKHGSP
At5g54745  -------KIFSFSREPNVVQPWQTTEKEYS-SSGFAIS-----GRRILTNAHVVG------------DHSYLQVRKHGSP
At3g03380  -RALGKVVPAVVVLRTTACRAFDTESAGASYATGFIVDKR---RGIILTNRHVVKPGP------------VVAEAMFVNR
Os02g48180 ----------VVVLRTTAPRAFDTEVAGASYATGFVVDKS---RGIILTNRHVVKPGP------------VVAEAMFVNR
Os08g04920 DSVLEWAKERRRIAKLSKKDGGGLISRCTGVVIGWDGANK---RAKILTAASVVCDFHGELHN-----PALKLSVSMPNK
Os12g04750 ------DVASSICENTVALASFNGDKRTFACTGFFIEWNE---CATILTSANLLRDSSDENR----IAENLRIEVLLPNN
Os12g04740 --------QVQETFSNHYKSVVLIRRGSDGIGTGFIIGKTRK-SYVAMTCYHVISGNP----------SGALKVRLPRDT
Os03g62900 --------------------IDGRRVVMHGAGSGFIISSTADGKCIVLTCRHVVKGSKGFDP----ATDLLRIRFLQGVE

At1g28320  WCPANVVYICKEQLDIALLQLEYVPGKLQPITANFSSPPLGTTAHVVGHGLFGPRCGLSPSICSGVVAKVVHAKRRLNTQ
Os05g41810 WCNASVVFISKGPLDVALLQMEKTPIELCAIRPEFVCPTAGSSVYVVGHGLLGPRSGLSSSLSSGVVSKIVKIPSTQHSQ
At3g27925  TTFDAKVVGFDQDKDVAVLRIDAPKNKLRPIPVGVSADLLVGQKVFAIGNPFGLDHTLTTGVISGLRREISSAATG----
Os05g49380 TVYEAQVVGFDQDKDVAVLRIKAPTDKLRPVPVGVSADLLVGQKVFAIGNPFGLDHTLTTGVISGLRREISSAATG----
At5g39830  KNFEGKLVGADRAKDLAVLKVDAPETLLKPIKVGQSNSLKVGQQCLAIGNPFGFDHTLTVGVISGLNRDIFSQTG-----
Os04g38640 KNFEGKLVGADRSKDLAVLKVDAPTDLLKPINVGQSSALRVGQQCLAIGNPFGFDHALTVGVISGLNRDIFSQAG-----
At4g18370  FSKEGKIVGLDPDNDLAVLKIETEGRELNPVVLGTSNDLRVGQSCFAIGNPYGYENTLTIGVVSGLGREIPSPNG-----
Os12g42210 YLKEGRLVGCDPSYDLAVLKVDVDGDKLRPALIGTSKGLRVGQSCFAIGNPYGYEHTLTTGVVSGLGREIPSPNG-----
At5g27660  RTFEGVVVNADLQSDIALVKIKSKTP-LPTAKLGFSSKLRPGDWVIAVGCPLSLQNTVTAGIVSCVDRKSSDLGLG----
Os11g14170 REFEGTVLNADRHSDIAVVKIKSKTP-LPSANLGSSSKLRPGLERIRTSTLITLKLLFDELDFLFKFGILKDE-------
At5g36950  IKHRAEVQAVGHECDLAILVVDSEVFWEGMNALELGDIPFLQEAVAVVGYPQGGDNISVTKGVVSRVEPTQYVHGA----
Os05g34460 TKYKAEVQAVGHECDLALLKVDSEEFWDGMNSLELGDIPFLQEAVAVVGYPQGGDNISVTKGVVSRVEPTQYAHGA----
At5g40560  KKYKAEVKAIGRECDLAILVIESKEFWEDMNPLELGDMPFLQESVNVIGYPTGGENISVTKGVVSRIESMDYAHGA----
At1g65640  TKYKAQVRAIGHECDLAILEIDNEEFWEDMIPLELGEIPSLDESVAVFGYPTGGDSVSITKGYVSRVEYTRYAHGG----
At1g65630  TKYKAKVQAVGHECDLAILEIDNDKFWEGMNPLELGDIPSMQDTVYVVGYPKGGDTISVSKGVVSRVGPIKYSHSG----
At3g16540  IKYKAKVQKISHECDLAILEIDSDEFWKGMNPLELGDIPPLQEVVSVVG----GENICITKGLVLRVETRIYDYSD----
At3g16550  VKYKAKVQKIAHECDLAILEIDSDEFWKGMNPLEFGDIPPLNEIVYVVGYPKAGETICVTKGVVTGVKTGNYLRSS----
At2g47940  RKYVAKVLVRGVDCDIALLSVESEDFWKGAEPLRLGHLPRLQDSVTVVGYPLGGDTISVTKGVVSRIEVTSYAHGS----
Os05g05480 KKYIAKVLARGIECDLALLSVENEEFWRGTEPLQLGRLPCLQDSVTVVGYPLGGDTISVTKGVVSRIEVTPYAHGT----
Os02g50880 TKYLATVLAIGTECDIALLTVDDDEFWEGVLPVEFGSLPALQDAVTVVGYPIGGDTISVTSGVVSRIEILSYVHGS----
Os06g12780 TKYLATVLAIGTECDIAMLTVEDDEFWKGVSPLEFGSLPALQDAVTVVGYPIGGDTISVTSGVVSRIEILSYVHGS----
At5g40200  TKYLATVLAIGTECDIALLTVTDDEFWEGVSPVEFGDLPALQDAVTVVGYPIGGDTISVTSGVVSRMEILSYVHGS----
At1g51150  TKYKAEVKAFRYGCDLAILGIDSEEFWEDINPLELGGIPFIGETVYALGYPRGGDTISVTKGIVTRVEPQKYSHSS----
At5g54745  TKYKAEVKAFGIFG--------ARR------------YTFIGETIYALGYPRDGDIISVTKGIVTRVEPQKYAHSS----
At3g03380  EEIPIYPVYRDPVHDFGFFCYDPSAVQFLTYQEIPLAPEAASVGLEIRVVGNDSGE-KVSILAG------TLARLDRDAP
Os02g48180 EEIPVYPLYRDPVHDFGFFRYDPGAIKFLKYDEIPLAPEAASVGLEIRVVGNDSGEKETSLQLVGSETVDMCQSMVSILA
Os08g04920 TTTEGRLLFYNVHYGIALLEVMGDYKLEVP---SFGSGTNYGQVIFALGRGENMSLMVSHGTISWTDYPVLLRNH-----
Os12g04750 LRTVGTVQHYNLHYNVALVSVKDHCVRQPVKIQPYGHNCRKLLAVGRIFESGRLMAARGQQFPTVVTHDCKFLSYSGCTT
Os12g04740 KDYVAELLYEHQGYDLAIIKVNGVSGECPILQFGDLEGVAHRANVVQLGYILGSQFALNLDPSVSPGSVIRPANQN----
Os03g62900 EDMQGQLILEDPFLDIAFILVSNMPIMLPALRFAPGVDLPVGTPVFLLGNCFLEQLPGCNIQTAIMPTIPTVSPGGISAP
```

```
At1g28320 S---ISQEVAEFPAMLETTAAVHPGGSGGAVLNSSGHMIGLVTSNARHGAGTVIPHLNFSIPCAVLAPIFKFAEDM----
Os05g41810 LSSVVEVNNMDIPVMLQTTAAVHPGASGGVLLDSLGRMVGLITSNAKHGGGSTIPHLNFSIPCKSLEMV----------
At3g27925 ---------RPIQDVIQTDAAINPGNSGGPLLDSSGTLIGINTAIYSP--SGASSGVGFSIPVDTVGGIV----------
Os05g49380 ---------RPIQDVIQTDAAINPGNSGGPLLDSSGNLIGVNTAIYSP--SGASSGVGFSIPVDTVGGIV----------
At5g39830 ---------VTIGGGIQTDAAINPGNSGGPLLDSKGNLIGINTAIFTQ--TGTSAGVGFAIPSSTVLKIV----------
Os04g38640 ---------VTIGGGIQTDAAINPGNSGGPLLDSKGHMIGINTAIFTQ--TGTSAGVGFAIPSSTVLKIA----------
At4g18370 ---------KSISEAIQTDADINSGNSGGPLLDSYGHTIGVNTATFTRKGSGMSSGVNFAIPIDTVVRTV----------
Os12g42210 ---------RPIRGAIQTDAAINSGNSGGPLIDSYGHVIGVNTATFTRKGTGISSGVNFAIPIDTVVQSV----------
At5g27660 ---------GKHREYLQTDCSINAGNSGGPLVNLDGEVIGVNIMKVLA-------ADGLGFSVPIDSVSKII---------
Os11g14170 -----------------------KGNSGGPLVNLDGEIVGVNVMKVWA-------ADGLSFAVPIDSIVKIV---------
At5g36950 ----------TQLMAIQIDAAINPGNSGGPAIMG-NKVAGVAFQNLSG-----AENIGYIIPTPVIKHFI----------
Os05g34460 ----------TQLMAIQIDAAINPGNSGGPAIMG-DKVAGVAFQNLSG-----AENIGYIIPVPVIKRFI----------
At5g40560 ----------INLPAIQTDAAMNPGNSGGPVCIG-NKVVGVAFQTLGH-----SNNIGCLIPAPVVKHFI----------
At1g65640 ----------TTLLAIQTDAAINPGNSGGPAIIG-NKMAGVAFQKDPS-----ADNIGYIIPTPVIKHFL----------
At1g65630 ----------TELLAIQIDAAINNGNSGGPVIMG-NKVAGVAFESLCY-----SDSIGYIIPTPVIRHFLNAI-------
At3g16540 ----------SDLLSIQIDATINDENSGGPVIMG-NKVVGVVYEIG-------------FVIPTPIIKHFI----------
At3g16550 ----------TKLLTIHIDATTYGGNSGGPVITG-DKVLGVLFQILGD-----KKSTGVVIPTPIIRHFI----------
At2g47940 ----------SDLLGIQIDAAINPGNSGGPAFNDQGECIGVAFQVYRSE---ETENIGYVIPTTVVSHFL----------
Os05g05480 ----------SDLLGIQIDAAINPGNSGGPAFNDHGECIGVAFQVFRSD---EAENIGYVIPTTVVSHFL----------
Os02g50880 ----------TELLGLQIDAAINSGNSGGPAFNDRGKCVGIAFQSLKHE---DAENIGYVIPTPVIMHFI----------
Os06g12780 ----------TELLGLQIDAAINSGNSGGPAFNDKGKCVGIAFQSLKHE---DVENIGYVIPTPVINHFI----------
At5g40200 ----------TELLGLQIDAAINSGNSGGPAFNDKGKCVGIAFQSLKHE---DAENIGYVIPTPVIVHFI----------
At1g51150 ----------------------------------------------------IKMYVYTSGGSTNKFYSGQINKKI---------
At5g54745 ----------IEILTIQTDACINGGKSGGPVVMGNKV-AGVV-------FEND----SPSDK------------------
At3g03380 HYKKDGYNDFNTFY--MQAASGTKGGSSGSPVIDWQGRAVALNAGSKSSSASAFFLPLQRVVRAL---------------
Os02g48180 ----------------------------GTLARLDREAPYYKKDGYNDFNTFYMQVRKFI--------------------
Os08g04920 ---NMFLSCDIPEGG-------------SGGPVVDHGGNMIGIAFVENPGP----------VFISIKTIMTCM----------
Os12g04750 TKAGI-----------------------GGPLLCFDGTFVGMNFYDEG-------------VEGTAFLSWC----------
Os12g04740 --------GMMGSQDVVYSAAARHGASGS-AVMFDDKVIGVLYSMSTN------SQVAYARSSTTVHMALKNWL------
Os03g62900 CKVEYG--PHITRREIQFTCPNKAGYSGSPLLHEEKVIGILGRGAYQAS---------LAVCTENLITFL----------
```

b)

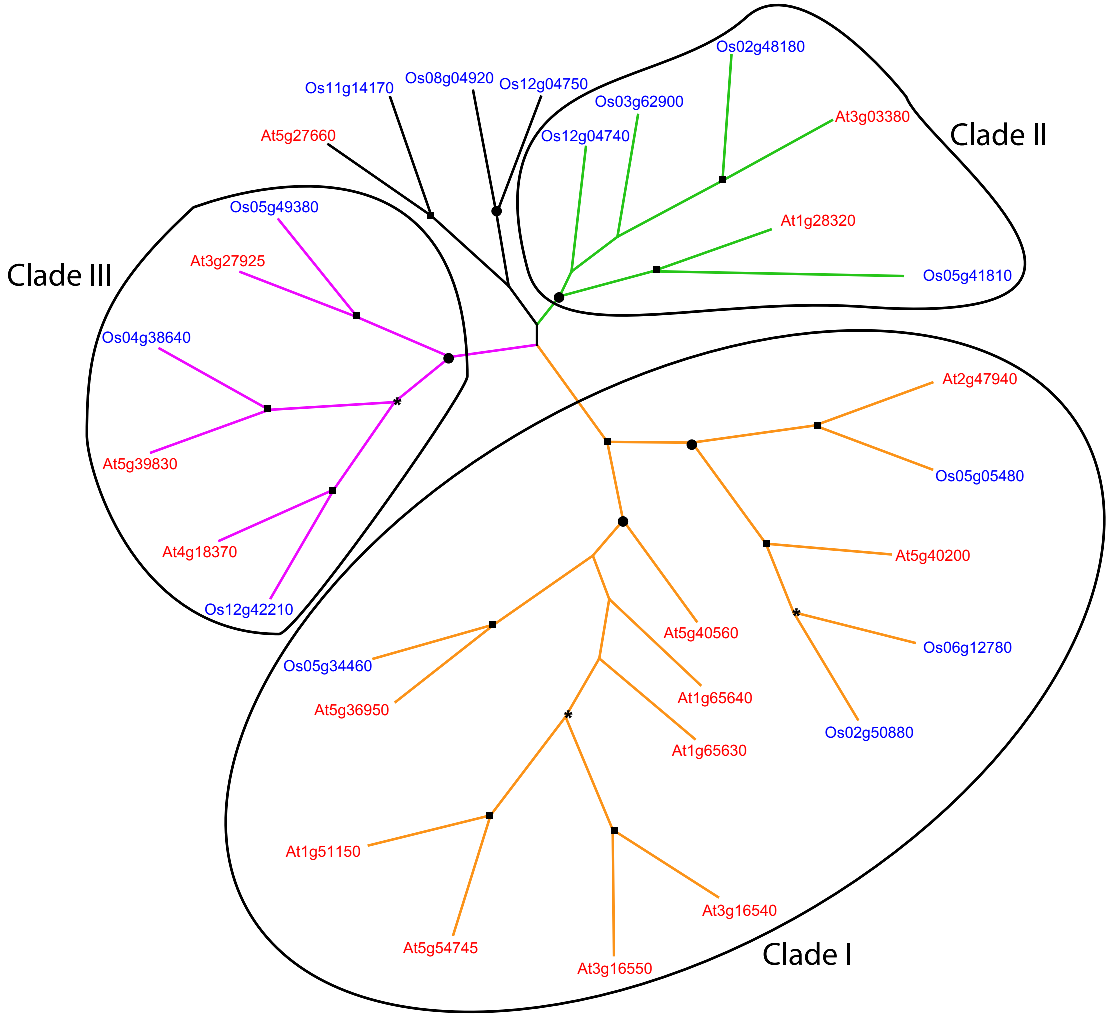
Clade I
Clade II
Clade III
Os11g14170
Os08g04920
Os12g04750
Os03g62900
Os02g48180
At3g03380
At5g27660
Os12g04740
At1g28320
Os05g41810
Os05g49380
At3g27925
Os04g38640
At5g39830
At4g18370
Os12g42210
At2g47940
Os05g05480
At5g40200
Os06g12780
Os02g50880
At5g40560
At1g65640
At1g65630
Os05g34460
At5g36950
At1g51150
At5g54745
At3g16550
At3g16540
