# Supplementary material for: Cross genome comparisons of serine proteases in Arabidopsis and rice
Source: BMC Genomics. 2006 Aug 9;7:200. doi: 10.1186/1471-2164-7-200 (PMC1560137; doi:10.1186/1471-2164-7-200)
Supplement: Additional file 6 — Figure SF2. Multiple sequence alignment of Arabidopsis and rice subtilisin-like proteins. Multiple sequence alignment of the subtilisin domain region of the annotated Arabidopsis and rice subtilisin protease-like proteins. The catalytic triad residues are indicated. Gene names correspond to those in Additional files 1 and 2. For brevity, rice gene names have been shortened to OsXXg##### instead of LOC_OsXXg#####, XX referring to chromosome 1–12 and a 5 digit number assigned to each gene. [file 1471-2164-7-200-S6.pdf]

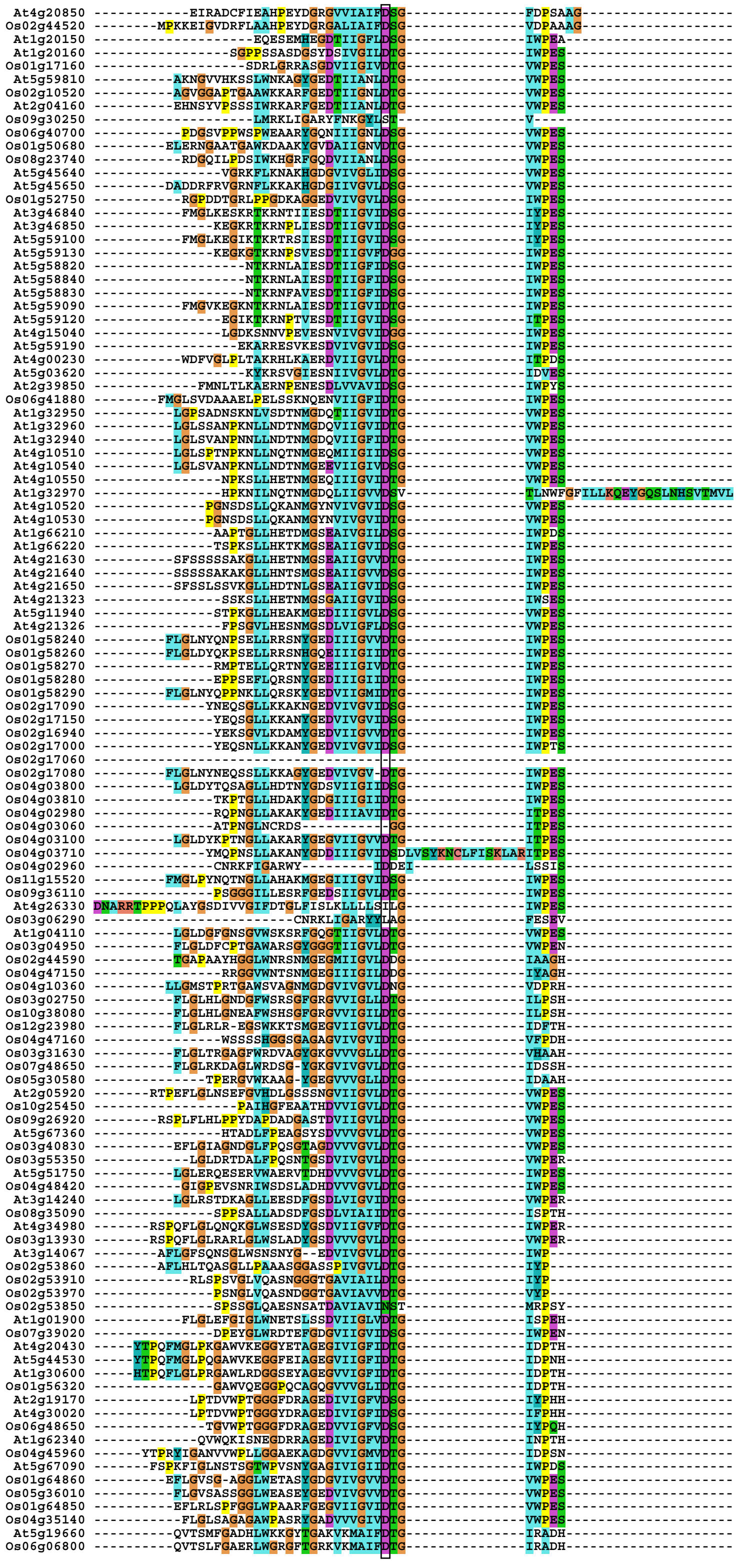

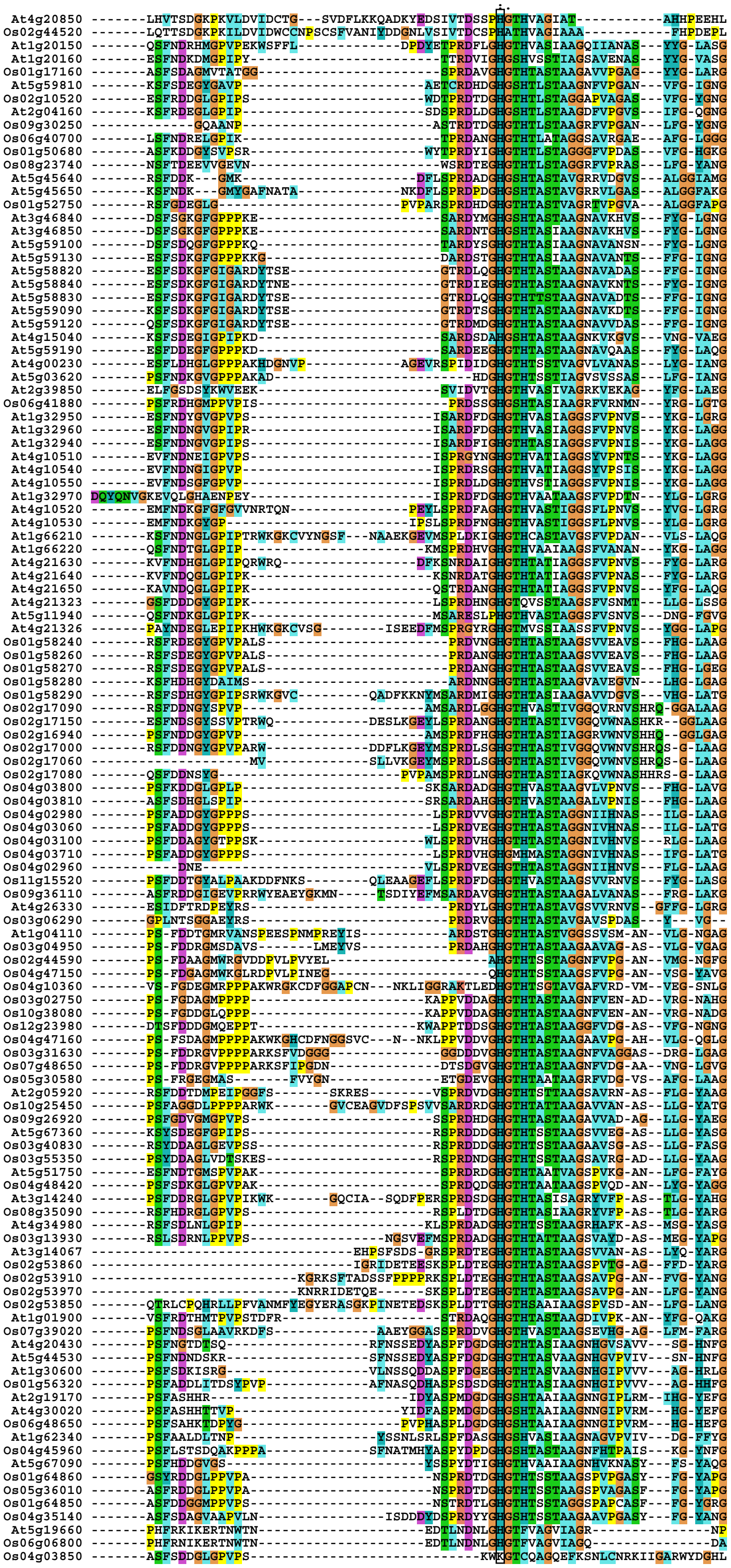

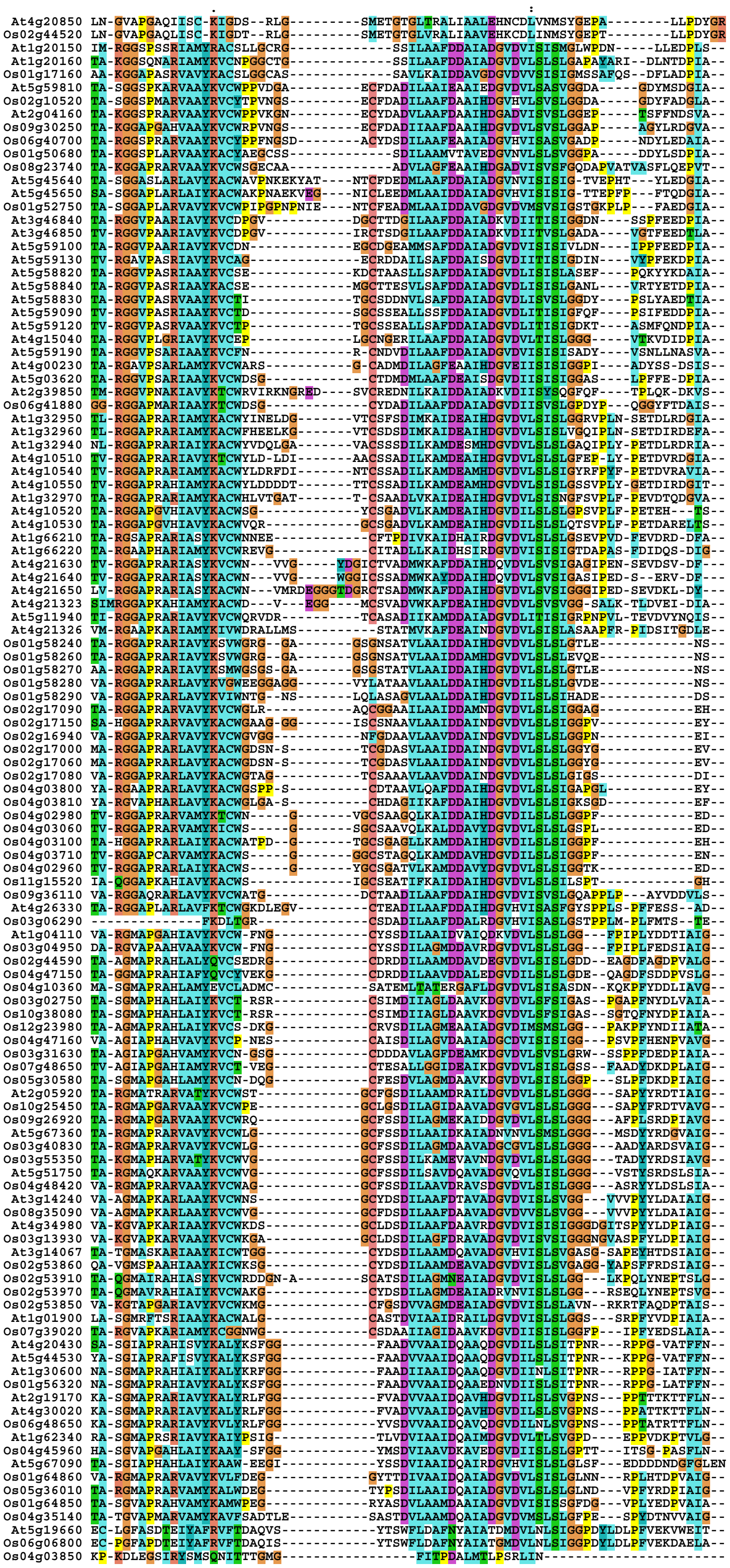
At4g20850
Os02g44520
At1g20150
At1g20160
Os01g17160
At5g59810
Os02g10520
At2g04160
Os09g30250
Os06g40700
Os01g50680
Os08g23740
At5g45640
At5g45650
Os01g52750
At3g46840
At3g46850
At5g59100
At5g59130
At5g58820
At5g58840
At5g58830
At5g59090
At5g59120
At4g15040
At5g59190
At4g00230
At5g03620
At2g39850
Os06g41880
At1g32950
At1g32960
At1g32940
At4g10510
At4g10540
At4g10550
At1g32970
At4g10520
At4g10530
At1g66210
At1g66220
At4g21630
At4g21640
At4g21650
At4g21323
At5g11940
At4g21326
Os01g58240
Os01g58260
Os01g58270
Os01g58280
Os01g58290
Os02g17090
Os02g17150
Os02g16940
Os02g17000
Os02g17060
Os02g17080
Os04g03800
Os04g03810
Os04g02980
Os04g03060
Os04g03100
Os04g03710
Os04g02960
Os11g15520
Os09g36110
At4g26330
Os03g06290
At1g04110
Os03g04950
Os02g44590
Os04g47150
Os04g10360
Os03g02750
Os10g38080
Os12g23980
Os04g47160
Os03g31630
Os07g48650
Os05g30580
At2g05920
Os10g25450
Os09g26920
At5g67360
Os03g40830
Os03g55350
At5g51750
Os04g48420
At3g14240
Os08g35090
At4g34980
Os03g13930
At3g14067
Os02g53860
Os02g53910
Os02g53970
Os02g53850
At1g01900
Os07g39020
At4g20430
At5g44530
At1g30600
Os01g56320
At2g19170
At4g30020
Os06g48650
At1g62340
Os04g45960
At5g67090
Os01g64860
Os05g36010
Os01g64850
Os04g35140
At5g19660
Os06g06800
Os04g03850

```
 At4g20850 FVD-LVTEAVNKRRLIFVSSAG---NSGPALTTV---GAPGGTTSSIIGVG-----------------------------
Os02g44520 FID-LASEVVDKHRIIFISSAG---NNGPALNTV---GAPGGTSSSIIGVG-----------------------------
 At1g20150 ----IGSFHAVERGITVVCSVG---NSGPSSQSVFNAAPWMITVAASTIDL-----------------------------
 At1g20160 ----IGAFHAVEQGILVICSAG---NDGPDGGTVTNTAPWIMTVAANTIDS-----------------------------
Os01g17160 ----LGAFHAHQRGVLVVCSGG---NDGPNPYTVVNSAPWILTVAASSID------------------------------
 At5g59810 ----IGSFHAVKNGVTVVCSAG---NSGPKSGTVSNVAPWVITVGASSMD------------------------------
Os02g10520 ----IGSFHAVRHGIAVVCSAG---NSGPAPGTVSNVAPWLFTAAASTMDR-----------------------------
 At2g04160 ----IGSFHAAKKRIVVVCSAG---NSGPADSTVSNVAPWQITVGASTMDI-----------------------------
Os09g30250 ----IGSFHAVRRGVTVVCSAG---NSGPGAGTVSNTAPWLVTVGASTMDT-----------------------------
Os06g40700 ----IGALHAVKAGITVVCSAS---NFGPDPGTVTNVAPWILTVAASTMDD-----------------------------
Os01g50680 ----IGAFYAVQKGVIVVCSAS---NSGPQPGSVTNVAPWILTVGASTMD------------------------------
Os08g23740 ----LGSLHAAMNGVSVVCSAG---NSGPLEDTVVNAAPWVTTVAASTVD------------------------------
 At5g45640 ----IGALHAVKRDIVVAASAG---NDGPARETLSNPAPWIITVGASSLD------------------------------
 At5g45650 ----MGALHAVKRNIVVAASAG---NSGPKPGTLSNLAPWIITVGASTLD------------------------------
Os01g52750 ----VGALHAAMRGVVLVCSGG---NSGPKPATVSNLAPWMLTVAASSIDRA----------------------------
 At3g46840 ----IGAFHAMAKGILIVNSAG---NSGPEPSTVASIAPWMFTVAASNTN------------------------------
 At3g46850 ----IGAFHAMAKGILTVNGAG---NNGPERRTIVSMAPWLFTVAASNMNP-----------------------------
 At5g59100 ----IGAFHAMAVGVLTVNAAG---NNGPKISTVTSTAPWVFSVAASVTN------------------------------
 At5g59130 ----IGAFHAMSKGILTVNAAG---NTGPDTASITSLAPWLLTVAASTAN------------------------------
 At5g58820 ----IGAFHANVKGILTVNSAG---NSGSFPSTTASVAPWILSVAASNTN------------------------------
 At5g58840 ----IGAFHAMVKGILTVQSAG---NGGPNPGSVMSVAPWILTVAASNTN------------------------------
 At5g58830 ----IGAFHAMAKGILTVHSAG---NAGPNPTTVVSVAPWMLTVAATTTN------------------------------
 At5g59090 ----IGAFHAMAKGILTVSSAG---NSGPKPTTVSHVAPWIFTVAASTTN------------------------------
 At5g59120 ----IGAFHAMAKGVLTVNSAG---NSGPKPISVSGVAPWILTVAASTTN------------------------------
 At4g15040 ----IGSFHAMTKGIVTTVAVG---NAGTALAKADNLAPWLISVAAGSTDR-----------------------------
 At5g59190 ----IGSFHAMMRGIITAGSAG---NNGPDQGSVANVSPWMITVAASGTDF-----------------------------
 At4g00230 ----VGSFHAMRKGILTVASAG---NDGPSSGTVTNHEPWILTVAASGIDP-----------------------------
 At5g03620 ----IGAFHAMKRGILTTCSAG---NNGPGLFTVSNLAPWVMTVAANSLDI-----------------------------
 At2g39850 ----WAFLRALKNGILTSAAAGNYANNGKFYYTVANGAPWVMTVAASLKD------------------------------
Os06g41880 ----IGSFHATSNGILVVSSAG----NAGRKGSATNLAPWILTVAAGTTD------------------------------
 At1g32950 ----TGAFHAVAKGIVVVCAGG---NAGPSSQTVVNTAPWILTVAATTLD------------------------------
 At1g32960 ----TGLFHAVAKGIVVVCAGG---NDGPAAQTVVNIAPWILTVAATTLD------------------------------
 At1g32940 ----TGAFHAVAKGIIVVCAGG---NSGPAAQTVLNTAPWIITVAATTLD------------------------------
 At4g10510 ----TGAFHAVLKGITVVCAAG---NAGPAAQTVGNTAPWILTVAATTLD------------------------------
 At4g10540 ----TGAFHAVLKGITVVCSGG---NSGPAAQTVGNTAPWILTVAATTLD------------------------------
 At4g10550 ----TGAFHAVLKGITVVCSGG---NSGPDSLTVTNTAPWIITVAATTLD------------------------------
 At1g32970 ----VGAFHAVAKGIPVVCAGG---NAGPSSQTISNTAPWIITVAATTQD------------------------------
 At4g10520 ----VGAFHAVAKGIPVVIAAG---NAGPTAQTISNVAPWVLTVAATTQD------------------------------
 At4g10530 ----VGAFHAVAKGIPVVAAAS---NAGPTAQTLSNVAPWVLTVAATTQD------------------------------
 At1g66210 ----IAAFHAVMKGIPVVCAGG---NDGPEKETISNVAPWLITVAATTMDS-----------------------------
 At1g66220 ----FGSFHAVMKGIPVVASAG---NEGPNAQTVDNVAPWIITVAATSLD------------------------------
 At4g21630 ----IAAFHAVAKGITVVAAGG---NDGPGAQNITNAAPWLLTVAATTLD------------------------------
 At4g21640 ----IAAFHAVAKGITVVAAAG---NDGSGAQTICNVAPWLLTVAATTLD------------------------------
 At4g21650 ----IAAFHAVAKGITVVAAAG---NEGPGAHTVDNVAPWLLTVAATTLD------------------------------
 At4g21323 ----IPALHAVNKGIPVVSPAG---NEGSRSSSVINVSPWILTVAATTLD------------------------------
 At5g11940 ----YGAFHAVAKGIPVLSAGG---NFGPGAYTVQNIAPWIITVAATTLD------------------------------
 At4g21326 ----LGSFHAVMKGIPVIAGAS---NTGPEAYTVANVFPWMLTVAATNID------------------------------
Os01g58240 ----FGALHAVQKGITVVYAAT---NFGPAPQVVRNTAPWVITVAASKIDP-----------------------------
Os01g58260 ----FGALHAVQKGITVVYAAG---NSGPVPQVVGNTAPWVITVAASKIDP-----------------------------
Os01g58270 ----FGAQHAVQKGITVVYAAM---NLGPAPQVVQNTAPWVITVAASKID------------------------------
Os01g58280 ----FGALHAVQNGITVVYAGG---NRGPRPQVLYNTAPWVITVAASKIDP-----------------------------
Os01g58290 ----FGALHAVQKGITIVYAGG---NDGPRPQVIFNTAPWVITAAASKIDP-----------------------------
Os02g17090 ----YETLHAVARGIPVVFGGG---NDGPTPQIVRNTVPWVITVAASTID---P--------------------------
Os02g17150 ----LSSRHAVARGIPVVFSAG---NDGPTPQTVGSTLPWVITVAASTID---P--------------------------
Os02g16940 ----HGTLHAVARGITVVFAGG---NDGPTSQTVQNTVPWVITVAAATIDTKFP--------------------------
Os02g17000 ----AGTLHAVARGITVVFAGG---NEGPVPQSVSNAVPWVITVAASTIDRLLR--------------------------
Os02g17060 ----AGTLHAVARGITVVFAGG---NEGPVPQSVSNAVPWVITVAASTIDEFP---------------------------
Os02g17080 ----PGTLHAVASGMTVVFAGG---NAGPAPQTVENVVPWVITVAATTIDDFP---------------------------
Os04g03800 ----PASLQAVKNGISVIFSAG---NEGPAPRTVKNASPWAMSVASATID------------------------------
Os04g03810 ----FSSFHAVKNGITVIFAAG---NEGPAPRTVTNALPWVITVASATID------------------------------
Os04g02980 ----PGTLHVVAKGIPVVYSAG---NDGPIAQTVENSSPWLLTVAAATMD------------------------------
Os04g03060 ----LGTLHVVAKGIPVVYSAG---NDGPVAQTVENSSPWLLTVAAATMDTQTTIGKVTAPKVAAFSSRGPS--------
Os04g03100 ----MGTLHVVANGIAVVYSAG---NDGPIAQTVENSSPWLLTVAAATMD------------------------------
Os04g03710 ----YGTLHVVAKGIPVVYSAG---NDGPITQTVENSSPWLLTVTAATMDR-----------------------------
Os04g02960 ----VGTLHVVANGISVVYAGG---NDGPIAQTVENQSPWLVTVAATTIDVAAK--------------------------
Os11g15520 ----TPAFHAVMKGIPVIYAAG---NDGPYTQTVNSVAPWLLTVAASTMDLCYA--------------------------
Os09g36110 ----IGSFHAVAKGVVVVCSAG---NSGPYSETVINSAPWIVTVAAGTIDS-----------------------------
 At4g26330 ----IGAFHAAERGISVVFSTG---NDGPDPGVVQNVAPWAVSVAASTVDS-----------------------------
Os03g06290 ----IGAFHAMQLGVPAVFSAG---NDGPDAAMVQNVSPWVITVAASTID------------------------------
 At1g04110 ------TFRAMERGISVICAAG---NNGPIESSVANTAPWVSTIGAHGTLDPS---------------------------
Os03g04950 ------SFRATTHGVSVVCAAG---NNGPSPSSVANEAPWVITVGAG-TLDPS---------------------------
Os02g44590 ------AYTAIMRGVFVSSSAG---NNGPNPLTVSNEAPWLLTVAASTTGKSPTAALIYKGTVFGDRKTPEVAPFSSRGP
Os04g47150 ------GYSAAMHGVLVSAAGG---NTGPGPSTVVNEAPWVITVGAGTTDRS----------------------------
Os04g10360 ------SFSAVMAGVFFSTSAG---NAGPTAETVTNCAPWQLTVGASTMGPN----------------------------
Os03g02750 ------TFKAMEHGIFVSSAAG---NDGPVAATVGNGAPWMLTVAAGTMDKA----------------------------
Os10g38080 ------GFKAMERGIVVSCAAG---NSGPDPGTVGNGAPWMLTVAAGTMDKA----------------------------
Os12g23980 ------SFSAMRKGIFVSLAAG---NSGPSSSTLSNEAPWVLTVGASTIDTA----------------------------
Os04g47160 ------TFGAMEKGVFVSMAAG---NAGPNVSSVINDAPWMLTVAASTMD------------------------------
Os03g31630 ------AFSAVARGITVVCAAG---NGGPEPSTVSNDAPWLLTVAAGSVGRS----------------------------
Os07g48650 ------AFSAVSKGIVVVCAAG---NNGPAFATLSNEAPWMVTVAASSVDFL----------------------------
Os05g30580 ------AFGAMSKGIAVVCAGG---NSGPTHFTLSNEAPWMLTVAAGSVDRS----------------------------
 At2g05920 ------AFSAMERGVFVSCSAG---NSGPTRASVANVAPWVMTVGAGTLD------------------------------
Os10g25450 ------AFGAAAAGVFVACSAG---NSGPSGATVANSAPWVATVGAGTLD------------------------------
Os09g26920 ------ALAATRRGIVVACSAG---NSGPSPSSLVNTAPWVITVGAGTLDQV----------------------------
 At5g67360 ------AFAAMERGILVSCSAG---NAGPSSSSLSNVAPWITTVGAGTLDP-----------------------------
Os03g40830 ------AFAAMEQNVLVSCSAG---NAGPGTSTLSNVAPWITTVGAGTLD------------------------------
Os03g55350 ------AYSAMERGIFVSCSAG---NAGPGSATLSNGAPWITTVGAGTLDP-----------------------------
 At5g51750 ------TFGAMEMGVFVSCSAG---NGGPDPISLTNVSPWITTVGASTMDFL----------------------------
Os04g48420 ------SFGAMQMGVFVACSAG---NAGPDPISLTNLSPWITTVGASTMD------------------------------
 At3g14240 ------AFGAIDRGIFVSASAG---NGGPGALTVTNVAPWMTTVGAGTID------------------------------
Os08g35090 ------AFGATEAGIVVSASAG---NGGPGGLTVTNVAPWMATVGAGSMDRAPQ--------------------------
 At4g34980 ------SYGAASKGIFVSSSAG---NEGPNGMSVTNLAPWVTTVGASTIDL-----------------------------
Os03g13930 ------AYGAVSRGVFVATSAG---NEGPTAMSVTNLAPWLATVGAGTID------------------------------
 At3g14067 ------AFGATRHGIVVSCSAG---NSGPNPETATNIAPWILTVGASTVDP-----------------------------
Os02g53860 ------SFHAVSKGIVVSASAG---NSGPGEYTATNIAPWILTVGASTID------------------------------
Os02g53910 ------AFNAIRRGIVVSTSAG---NDGPGTYTANNLAPWVITVGASSIDRF----------------------------
Os02g53970 ------AFNAIRRGIFVSAAAG---NDGPDMSTANNLAPWMVTVGASSINRF----------------------------
Os02g53850 ------GFNAVRKGIVVVASAG---SGGPKESTVTNTAPWLLTVGASSMN------------------------------
 At1g01900 ------GFGAMQKNIFVSCSAG---NSGPTASTVSNGE-------------------------------------------
Os07g39020 ------TFGAQREGVFVALAGG---NSGPRPYTVTNVAPWMTTVGAGAVDR-----------------------------
 At4g20430 -PLDMAMLSAVKAGIFVVQAAG---NTGPSPKSMSSFSPWIFTVGAASHDFND---------------------------
 At5g44530 -PIDMALLSAVKAGIFVVQAAG---NTGPAPKTMSSFSPWIFTVGASSHDFND---------------------------
 At1g30600 -PIDMALLSAVKAGIFVVQAAG---NTGPAPKSMSSFSPWIFTVGATSHDFVD---------------------------
Os01g56320 -PIDMALLSAVKAGIFVVQAAG---NTGPSPKSMSSYSPWIFTVGASAHDLAN---------------------------
 At2g19170 -PFDATLLGAVKAGVFVAQAAG---NGGPFPKTLVSYSPWITTV-AAAIDDQD---------------------------
 At4g30020 -PFDATLLGAVKAGVFVAQAAG---NGGPFPKTLVSYSPWITTV-AAAIDDQD---------------------------
Os06g48650 -PFDAALLSAVKAGVFVAQAAG---NGGPFPKTLVSFSPWITTV-AAGVD-QD---------------------------
 At1g62340 -IFDLAMLLARKAGVFVVQAVG---NNGPSPSSVLSYSPWVVGVAAGNTDRSP---------------------------
Os04g45960 -LLETQLLLATKAGISVVQAVG---NGGPDANSVVSFSPWITSVGASTTDMQA---------------------------
 At5g67090 DPIAVASFAAIQKGVFVVTSGG---NDGPYYWSLINGAPWIMTVGAGTIGTSF---------------------------
Os01g64860 ------SFAAMQHGIFVSTSAG---NDGPGLSVLHNGAPWALTVAAGTVDVSC---------------------------
Os05g36010 ------AFAAMQRGVFVSTSAG---NDGPDPGFLHNGTPWTLTVASGTGDASC---------------------------
Os01g64850 ------AFAAIERGILVSASAG---NDGPRLGTLHNGIPWLLTVAAGMVDEG----------------------------
Os04g35140 ------AFAAVRRGILVTCSAG---NDGSDSYTVLNGAPWITTVGASTIDPVS---------------------------
 At5g19660 ------ASN-----IIMVSAIG---NDGPLYGTLNNPADQSDVIGVGGIDND----------------------------
Os06g06800 ------AAEERRRATAQTAAAA---ARAARLAAAELAAARAEAEAEAAKDAA----------------------------
Os04g03850 --------ATLASGKIVFCYNPAPMTTISPIFYTSKVVKYAKEAGAKGIILAP---------------------------
```

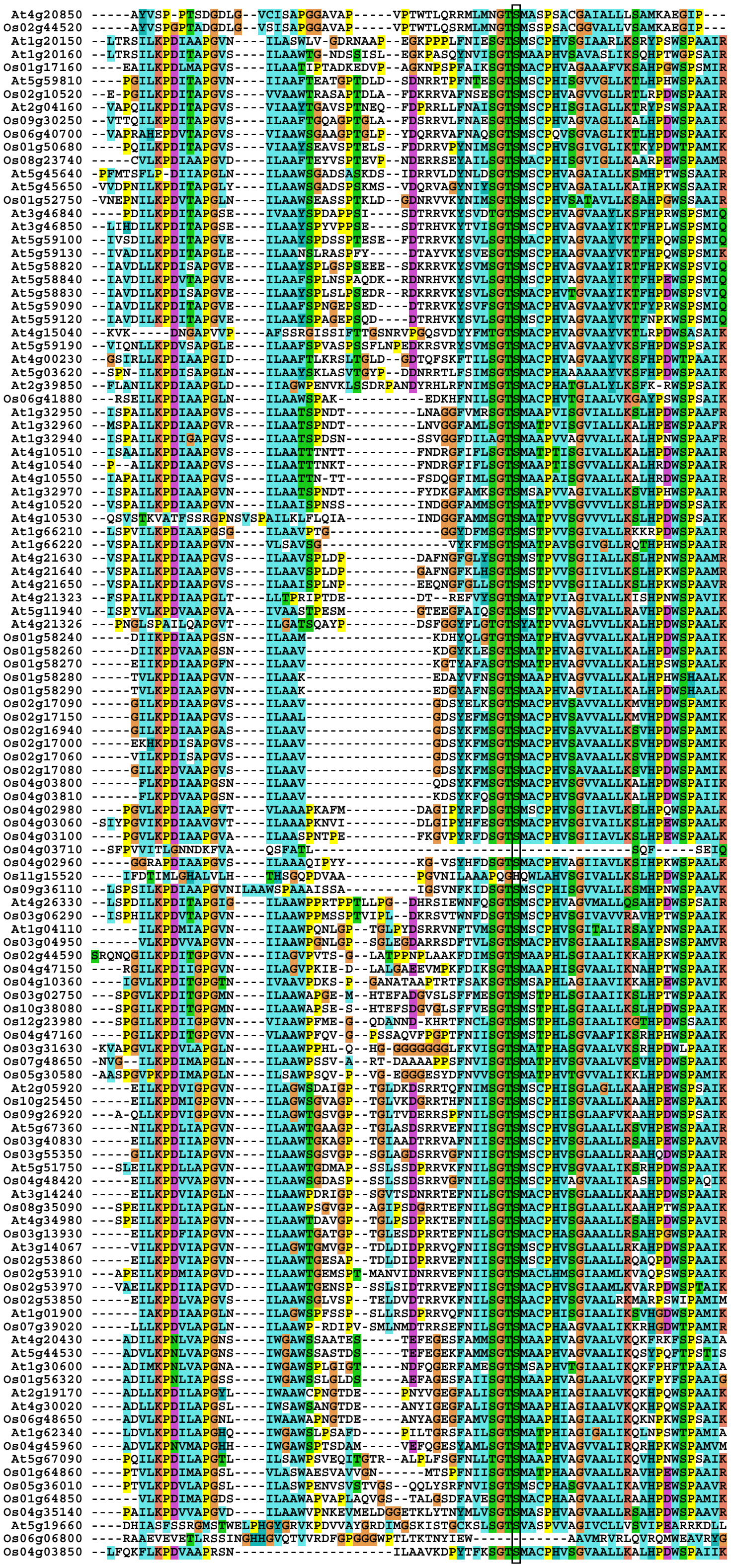

```
 At4g20850 ---VSPYSVRRALENTSTPVG--DLPEDKLTTG---------
Os02g44520 ---LSPYTVRKAIENTAASIS--DVPEEKLTTG---------
 At1g20150 SAIMTTAVQMTNTGSHITTETGEKATPYDFGAG---------
 At1g20160 SAIMTTATQTNNDKGLITTETGATATPYDSGAG---------
Os01g17160 SALMTTATTRNNLGQAVASSTG--------------------
 At5g59810 SAIMTTSRTRNNRRKPMVDESFKKANPFSYG-----------
Os02g10520 SALMTTAVEVDNERHAILNSSFAAANPFGFGAG---------
 At2g04160 SAIMTTATIMDDIPGPIQNATNMKATPFSFGAG---------
Os09g30250 SAIMTTARVKDNMRRPMSNSSFLRATPFSYGAG---------
Os06g40700 SAIMTTATELGNDMRPIMNSSMSPATPFSCGAG---------
Os01g50680 SAIMTTAITGDNDSGKIRDETGAAATPFAYGSG---------
Os08g23740 SAIMTTARTQDNTGAPMRDHDGREATAFAFGAG---------
 At5g45640 SALMTTASMTNEDNEPIQDYDGSPANPFALGSR---------
 At5g45650 SALMTTAWMTNDKKKPIQDTTGLPANPFALGSG---------
Os01g52750 SAIMTTATTSNAEGGPMMDADGTVAGPIDYGSG---------
 At3g46840 SAIMTTAWPMN-----ASTSPFNELAEFAYGAG---------
 At3g46850 SAIMTTAWPMN-----ASTSPSNELAEFAYGAG---------
 At5g59100 SAIMTTAWPMN-----ASGS-GFVSTEFAYGSG---------
 At5g59130 SAIMTTAWSMN-----ASQS-GYASTEFAYGAG---------
 At5g58820 SAIMTTAWPMK-----PNRP-GFASTEFAYGAG---------
 At5g58840 SAIMTTAWPMN-----ATGT-AVASTEFAYGAG---------
 At5g58830 SAIMTTAWQMN-----ATGT-GAESTEFAYGAG---------
 At5g59090 SAIMTTAWPVK-----AKGR-GIASTEFAYGAG---------
 At5g59120 SAIMTTAWPVN-----ATGT-GIASTEFAYGSG---------
 At4g15040 SAIMTTAWAMN-----ASKN---AEAEFAYGSG---------
 At5g59190 SAIMTTATPMN-----LKKN---PEQEFAYG-----------
 At4g00230 SAIITSAKPIS-----RRVN---KDAEFAYG-----------
 At5g03620 SALMTTATPMR-----IKG----NEAELSYG-----------
 At2g39850 SALMTTSSEMT-----DDDN------EFAYGSG---------
Os06g41880 SAIMTTATVLG-----NKRN--------AIATD---------
 At1g32950 SAIVTTAWRTDPFGEQIAAESSSLKVPDPFDYG---------
 At1g32960 SAIVTTAWRTDPFGEQIFAEGSSRKVSDPFDYGGG-------
 At1g32940 SAIVTTAWRTDPFGEQIFAEGSS-------------------
 At4g10510 SAIVTTAWRTDPFGEQIFAEG---------------------
 At4g10540 SAIVTTAWRTDPFGEQIFAEG---------------------
 At4g10550 SAIVTTAWKTDPFGEQIFAEG---------------------
 At1g32970 SAIVTTAWRTDPSGEPIFADGSNRKLADPFDYGGG-------
 At4g10520 SAIVTTAWRTDPSGEPIFADGSS-------------------
 At4g10530 SAIVTTAWRTDPSGEPIFADGSS-------------------
 At1g66210 SALVTTALQTDPSGEPIAAEGSPRKLADPFDYG---------
 At1g66220 SALVTTAWKTDPSGEPIFSEGSTRKLADPFDYGGG-------
 At4g21630 SALVTTAWRTSPSGEPIFAQGSNKKLADPFDYGGG-------
 At4g21640 SALVTTAWRTSPSGEPIFAQGSNKKLADPFDYGGG-------
 At4g21650 SALVTTAWRTSPSGEPIFAEGSNKKLADPFDYGGG-------
 At4g21323 SALVTTAMKTDPYGERLTVDGGNYKVADAFDYG---------
 At5g11940 SALITTASTTDPYG----------------------------
 At4g21326 SAIMTTAWKTDPSGEPIFAEG---------------------
Os01g58240 SAIVTTASVTDERGMPILAE----------------------
Os01g58260 SAVVTTASVTDERGMPILAE----------------------
Os01g58270 SAIVTTASVTDERGMPILAE----------------------
Os01g58280 SAIVTTASTKDEYDTPILAE----------------------
Os01g58290 SAIVTSASTKDEYG----------------------------
Os02g17090 SAIVTTASVTDRFGMPIQAEAV--------------------
Os02g17150 SAIVTTASVTDRFGIPIQAEG---------------------
Os02g16940 SAIVTTASVTDRFGMPIQAEG---------------------
Os02g17000 SAIVTTGMYSCHTTSSVYMPYMS-------------------
Os02g17060 SAIVTTGMYSCHTTSSVYIPYM--------------------
Os02g17080 SAIITTGTLPPLLYQVAVDVQLVTV-----------------
Os04g03800 SALVTTASNEKYGVPILADGLPQKIADPFDYG----------
Os04g03810 SALVTTASNDRYGLPILANG----------------------
Os04g02980 SAIMTTAALTYDNNGMPIQANGKVPKIADPFDYGAG------
Os04g03060 SAIMT-TALTYDNDGMPIQANG--------------------
Os04g03100 SAIMT-TANTFDNNGMPMQANG--------------------
Os04g03710 FYEREDCNAENINSTVKGKIVF-----CF-FG----------
Os04g02960 SAIMTTDYSIPLAGVHQRSGEQ--------------------
Os11g15520 SAIMTTAHITDNNGLPLVADATPNK-----------------
Os09g36110 SALVTTANVHDAYGFE--------------------------
 At4g26330 SAIMTTAYTRDTSYDLILSGGSMKSTDPFDIGAG--------
Os03g06290 SALMTTAYMYDDTSDVMLAGGTLKAADAFDVG----------
 At1g04110 SALMTTADLYDRQGKAIKDGNKPAGVFAIGAG----------
Os03g04950 SAIMTTADVTDRQGKPIMDGNG--------------------
Os02g44590 SAMMTTADTLDRRRRPITDQ-KGNNANMFGLGAG--------
Os04g47150 SAMMTTADYTDNLRKPITDV-DGAPATYYAIG----------
Os04g10360 SAMMTTADVTHRDGTPVIDLSTGAPASYFAMG----------
Os03g02750 SAIMTSSDVADHDGVPIK------------------------
Os10g38080 SAIMTTSDAVDRTGVPIK------------------------
Os12g23980 SAIMTTAYVVDNQKKAILDERYNIAG----------------
Os04g47160 SAIMTTADITDRSGNQILDE----------------------
Os03g31630 SAILTTSDAVDGAGNPILDE----------------------
Os07g48650 SAILTTSDEVDNTGGPILDE----------------------
Os05g30580 SAIMTTSSAVDNDGHAIMDEEHRKARLYSVG-----------
 At2g05920 SALMTTAYVLDNTNAPLHDAADNSLSNPYAHG----------
Os10g25450 SALMTTAYTVDNTNSSLRDAAGGLLATPFAFGAG--------
Os09g26920 SALMTTAYTVDNTGSPIVDAASNTTATPWS------------
 At5g67360 SALMTTAYKTYK---DGKPLLDIATGKPSTPFDHG-------
Os03g40830 SALMTTAYSTYAGAGDANPLLDAATGAPATPFDYGAG-----
Os03g55350 SALMTTSYNGYPNGNG--------------------------
 At5g51750 SALMTTAYVHDNMFKPLTDASGAAPSSPYDHG----------
Os04g48420 SALMTTAYVHDNTYRPMKDAATGKASTPFEHG----------
 At3g14240 SALITTAYTVDNSGEPMMDES-TGNT----------------
Os08g35090 SALMTTAYIKDNSNGTMVDES-TGVVADVFDFG---------
 At4g34980 SAMMTTTNLVDNSNRSLIDES-TGKSATPYDYGSG-------
Os03g13930 SALMTTAVATDNRGEAVGDEAEPGRVATPFDYGAG-------
 At3g14067 SALVTTAYDVENSGEPIEDLATGKSSNSFIHG----------
Os02g53860 SALMTTAYNVDNSSAVIKDLATG-------------------
Os02g53910 SAMMTTAYNVDNDGNAIKDMATGQAARPFELGSG--------
Os02g53970 SAMMTTAYEVDNGGNAIMSS----------------------
Os02g53850 SALTTTAGLDPGLVYDAGVD----------------------
 At1g01900 SAIMTTARITDNRNRPIGDRGAAGAE----------------
Os07g39020 SAMMTTAATLDNTGRDITDE----------------------
 At4g20430 SALSTTSVLFDNKGEAIMAQRAYANPDQTISPATPFDMGNG-
 At5g44530 SALSTTALLNDNKGSPIMAQRTYSNPDQSLYTATPSDMGSG-
 At1g30600 SALSTTASLSDRKGEHIMAQ----------------------
Os01g56320 SALSTTTSLSDREGNP--------------------------
 At2g19170 SALMTTSTVIDRAGRLLQAQQYSDTEAVTLVKATPFDYGSG-
 At4g30020 SALMTTSTVIDRAGRPLQAQQYSETETVTLVKATPFDYGSG-
Os06g48650 SALMTTSNTLDKGSHPLRAQQYSTSEIMTLTRATPFDYGSG-
 At1g62340 SAISTTANEYDSNGEIISAEYYELSR---LFPSNHFDHGAG-
Os04g45960 SAIMTTADVTDRSGRPLMARRDGG------------------
 At5g67090 SAIMTTALTLDNPLAVGAGHV---------------------
Os01g64860 SAMMTTAATLDNTGRSINDMARAGHAATPLAMGSG-------
Os05g36010 SAMMTTASAVDNTNAPIKDMGRANRGATPLAMGSG-------
Os01g64850 SAMMTTATAVDNTFRPIGDAGHG-------------------
Os04g35140 SAMMTTAYVKDNADDADLVSMPGGSPGTPLDYGSG-------
 At5g19660 NPASMKQALVEGAAKLSGPNMYEQG-----------------
Os06g06800 DVDYDEDRRALDALIAAVPPEMQFS-----------------
Os04g03850 SAIVTTASNDRFGLPILADG----------------------
```
