# Supplementary material for: Cross genome comparisons of serine proteases in Arabidopsis and rice
Source: BMC Genomics. 2006 Aug 9;7:200. doi: 10.1186/1471-2164-7-200 (PMC1560137; doi:10.1186/1471-2164-7-200)
Supplement: Additional file 7 — Figure SF3. Multiple sequence alignment of Arabidopsis and rice prolyl oligopeptidase -like proteins Multiple sequence alignment of the Prolyl oligopeptidase domain region of the annotated Arabidopsis and rice prolyl oligopeptidase-like proteins. The catalytic triad residues are indicated. Gene names correspond to those in Additional files 1 and 2. For brevity, rice gene names have been shortened to OsXXg##### instead of LOC_OsXXg#####, XX referring to chromosome 1–12 and a 5 digit number assigned to each gene. Based on the conservation of residues around the catalytic Ser residue, a few gene products were unambiguously assigned to their respective subfamilies prefixed by figures in parentheses (See text for details) [file 1471-2164-7-200-S7.pdf]

```
      At1g66900 ------------------------LSNRLRVNLMGYDYSGYGQSTGQASEC--NTYADIEASYKCLKEKYGVKDDQLIVY
      Os01g49510 ------------------------LSRRLRINLFGYDYSGYGRSTGKPTEC--NTYADIEAAYNCLKEKYGVADEDIILY
      Os02g09770 ------------------------LSAHLNVNLMGYDYSGYGQSSGKPSEH--NTYADIEAVYRCLVETYGASEENIILY
      Os06g42730 ------------------------LSSHLNVNLMGYDYSGYGQSSGKPSEQ--NTYSDIEAAYRCLVETYGATEENIILY
      At3g01690 ------------------------LSIHLKVNLMGYDYSGYGQSTGKPSEH--NTYADIEAVYKCLEETFGSKQEGVILY
      At4g24760 ------------------------LSIHLRVNLMGYDYSGYGQSSGKPTEQ--NTYADIEAAYKCLEENYGAKQENIILY
      Os12g18860 ------------------------LSFNLRVNVLGYDYSGYGQSSGKPSEH--NTYADIEAAYKCLIENFGAKEEEIILY
      At3g30380 ------------------------LSLHLRVNLIGYDYSGYGRSSGKPSEQ--NTYSDIEAVYRCLEEKYGVKEQDVILY
      At2g24320 ------------------------LRAHLRVNIMSYDYSGYGASTGKPTEL--NTYYDIEAVYNCLRTEYGIMQEEMILY
      Os02g55330 ----------------------LELRAHLRVNIMSYDYSGYGASTGKPSEY--NTYCDIEAVYDCLTKVYGIEPEDLILY
      At1g32190 ------------------------LKVNLRVNLMGYDYSGYGASTGKPSEY--DTYADIEAAYECLQTDYGVGQEDLILY
      At1g13610 -------------SDLAQIFYILAELIQLNVNLMGYDYSGYGQSSGKPSEQ--DTYADIEAAYNWLRQTYGTKDERIILY
      At5g20520 ------------GNIAHRLEMVRIMIQKLKCNVFMLSYRGYGASEGYPSQQ--GIIKDAQAALDHLSGRTDIDTSRIVVF
      Os07g41730 ----------------HRLDFVRLMMQRLQCNVFMLSYRGYGESDGYPSQK--GIINDAQAALDHLVQRKDIDTSRIVVF
      At4g14290 ----------------ADASEAAIVLLPSNITIFTLDFSGSGLSGGEHVTLGWNEKDDLKAVVEYLRTDGNVS--LIGLW
      Os03g24450 ----------------ADASEAAIILLPSNITVFTLDFSGSGLSEGEHVTLGWNEREDLKAVVNHLRTDGNIS--CIGLW
      At3g47560 ---------------FEIMKNVAVAIEREGISAFRFDFSGNGESEGSFYYG--NYNYEADDLHSVIQYFSNLNRVVTIIL
      Os01g39800 -----------------LILDLMAALTKKGISVFRFDFSGNGESEGEFEYG--NYRKEADDLHSVVSYLCKEKYDVTAIV
      Os05g46210 ---------------DSILVDLAYALTREGVSAFRFDFAGNGESEGQFQYG--NYRREADDLHSVVSYFTEQEYNIIGLV
      At5g25770 EWLRPWLEAYASRGYVAIGLDSRYHGERADCKTAYRDALISSWRNGNTMPFIFDTVWDLIKLAEYLTQRDDIDPKKIGIT
      Os06g06770 EWLRPLLEAYASRGYISVAIDSRYHGERANNNSTYIDALKSAWRNGDAMPFILDTVWDLIKLGDHLSEREDVDPCRIGIT
      At1g20380 ----------PFFSATRIVLGRHLGTVFCFANIRGGGEYGEEWHKSGALANKQNCFDDFISGAEYLVSAGYTQPRKLCIE
(S9A) At1g76140 ----------PSFSASRIVLSKHLGVVFCFANIRGGGEYGEEWHKAGSLAKKQNCFDDFISGAEYLVSAGYTQPSKLCIE
(S9A)Os01g01830 ----------PSFSVSRVVLCKNMGFVVCVANIRGGGEYGEEWHKAGARAMKQNCFDDFIACAELLISAGYTSYRQLCIE
      Os04g47360 ----------PHFSVTRIVLMRNLGFVSCIANIRGGGEYGEDWHKAGSLANKQNCFDDFIAAGEFLVSAGYTNPSRLCVE
      At1g50380 ----------PYFKASRLSLLDRG-FTFVIAHVRGGGEMGRQWYENGKLLKKKNTFTDFIACAERLIELKYCSKEKLCME
      Os06g51410 ----------PSFGRSRFSLVDRG-FIYVIAHIRGGGEMGRKWYEDGKLLNKRNTFTDFIACAEHLIENKYCSKEKLCIN
      At1g69020 ----------KSWCTNRLSMLDRG-WVIAFADVRGGGSGEFSWHKSGTRSLKQNSIQDFIYSAKYLVEKGYVHRHHLAAV
      Os09g29950 -----------SWCSDRLSLLARG-WVVAFADVRGG--GDSSWHLAGTKANKINSIKDFAACGTHLIKEGFVHKNRLCAI
      At5g66960 ----------KRWRSELKSLLDRG-WVLAYADVRGGGGKGKKWHQDGRGAKKLNSIKDYIQCAKYLVENNIVEENKLAGW
      Os09g28040 ----------KRWRSELKSLLDRG-WVIAFADVRGGGGYGKKWHQDGARTKKMNSIYDFISCGEFLLEKGIIKENKLAGW
      Os10g28020 -----------SSYSKSLAFLYSQGYNLLVVNYRGSLGFGEEALQSLPGNIGSQDVNDVLTALDFVIKKGLIDASKVAVV
      Os10g28030 -----------SSYSKTSAFLASLGFNLLIVNYRGTPGFGEEALQSLPGKVGSQDVQDCLTALDYVIEGGLIDASKVAVI
      At4g14570 -----------CSFSRTMAYLSSIGYSQLIINYRGSLGYGEDALQSLPGKVGSQDVKDCLLAVDHAIEMGIADPSRITVL
      At5g36210 -----------GSLNLNIQYWTSRGWAFVDVNYGGSTGYGREYRERLLRQWGIVDVDDCCGCAKYLVSSGKADVKRLCIS
      Os06g11180 -------------LDLSVQYWTSRGWAYLDVNYGGSTGFGREYRERLLGKWGIVDVDDCCSCARVLVESGKVDERRLCIT
      Os06g11190 -----------GVLDLGVQYWTSRGWAFVDVNYGGSTGYGRKFRERLLGQWGVVDVNDCCSCATFLVETGRVDAQRLCVT
(S9B) At5g24260 -----------NTVDMRTQYLRSRGILVWKLDNRGTARRGLKFESWMKHNCGYVDAEDQVTGAKWLIEQGLAKPDHIGVY
      Os02g18850 -----------STVDMRAQFLRSKGILVWKMDNRGTARRGLQFEGQLKYNIGRVDAEDQLAGAEWLIKKGLAKPGHIGLY
(S9D) At2g47390 ----------AGIGSTSALLWLARRFAILSGPTIPIIGEGDEEAN---DRYVEQLVASAEAAVEEVVRRGVADRSKIAVG
(S9D)Os03g19410 ----------PGIGATSPLLWLARGFAILSGPTIPIIGEGDEEAN---DRYVEQLVTSAEAAAEEVVRRGVAHPDKIAVG
(S9D)Os07g48970 --------------ARIRSNFPLLWFAILADPTIPIIGERDQEAN---DRYIEQLVASAEAAVNEIVRRGVAHPDKIAVG
      At1g26120 --------------AWG-SLLGQQLSERDIIVACIDYRNFPQGSISDMVKDASSGISFVCNHIAEYGGDP----DRIYLM
      At5g15860 --------------AWG-SLLGMQLAERDIIVACLDYRNFPQGTISDMVTDASQGISFVCNNISAFGGDP----NRIYLM
      Os10g04620 --------------FFLDGIARKIASAGYGVFALDYPGFGLSEGLHGFIPSFDTLVDDVAEHFTKVKENPEHRGLPSFLF
      At5g19630 ----------------ALLKGIASELASKGFKSVTFDTRGAGKSTGRATLTGFAEVKDVVAVCRWLCQN--VDAHRILLV
      Os01g42690 ----------------------------AWFDVEDTSVDGRDDIEG----------LDASAAHVANLLSSEPSDVKLGIG
      Os01g57770 -------------------WTYFKVATRLRSAGYRVTAPDLGASGVDPRPLREVPTFRDYTAPLLGLLGSLPPGEKVVLV
```

```
                 * * *.
         At1g66900 GQSVGSGPTVDLASRTPNLRGVVLQCPILSGMRVLYPVKCTYWFDIYKN-------------------------------
         Os01g49510 GQSVGSGPTIDLASRLPNLRGVVLHSPILSGLRVLYPVKRTYWFDIYKN-------------------------------
         Os02g09770 GQSVGSGPTLDLASRLPHLRAVVLHSPILSGLRVMYPVKHTYWFDIYKN-------------------------------
         Os06g42730 GQSVGSGPTLDLASRLPHLRAVVLHSPILSGLRVMYPVKHTYWFDIYKN-------------------------------
         At3g01690 GQSVGSGPTLDLASRLPQLRAVVLHSPILSGLRVMYSVKKTYWFDIYKN-------------------------------
         At4g24760 GQSVGSGPTVDLAARLPRLRASILHSPILSGLRVMYPVKRTYWFDIYKN-------------------------------
         Os12g18860 GQSVGSGPTVDLASRLHRLRAVVLHSPILSGLRVMYPVKRTYWFDIYKN-------------------------------
         At3g30380 GQSVGSGPTLELASRLPNLRAVVLHSAIASGLRVMYPVKRTYWFDIYKN-------------------------------
         At2g24320 GQSVGSGPTLHLASRVKRLRGIVLHSAILSGLRVLYPVKMTFWFDMYKN-------------------------------
         Os02g55330 GQSVGSGPTLHLASRLEKLRGVVLHSAILSGIRVLYPVKVTLWFDIFKN-------------------------------
         At1g32190 GQSVGSGPTLHLASKLPRLRGVVLHSGILSGLRVLCHVKFKFCCDIYSN-------------------------------
         At1g13610 GQSVGSGPSLELASRLPRLRALVLHSPFLSGLRVMYPVKHSFPFDIYKN-------------------------------
         At5g20520 GRSLGGAVGAVLTKNNPDKVSALILENTFTSILDMAGVLLPFLKWFIGGSGTK------------------------SLK
         Os07g41730 GRSLGGAVGAVLAKNNPGKVSALILENTFTSILDMAGIMLPFLRWFIGGSSSK------------------------GPK
         At4g14290 GRSMGAVTSLMYGAEDPSIAAMVLDSPFSDLVDLMMELVDTYKFRLPKFTVSKSKFVVSVSCGICSSYYKIKFAIQYMRR
         Os03g24450 GRSMGAVTSLMYGAEDPSIAGMVLDSPFSNLVDLMMELVDTYKYPLPKFTVK--------------------LAIQHMRK
         At3g47560 GHSKGGDVVLLYASKYHDIPNVINLSGRYDLKKGIGERLGEDFLERIKQQGYIDVKD--------------------GDS
         Os01g39800 GHSKGGDVVTLYASIYDDVRLVINVSGRFDLEKGIEERIGEGSIDRINKEGYLDVKDK------------------SGNV
         Os05g46210 GHSKGGNAVLLYASMNHDIPVIVNISGRFALERGIDGRLGKNFMQRIKKDGYIDVRNR------------------KGEF
         At5g25770 GISLGGMHAWFAAAADTRYSVVVPLIGVQGFRWAIENDEWEARVNSIKPLFEEARIDLG-------------KNIIDKEL
         Os06g06770 GESLGGMHAWFAAVVDTRYSVVVPIIGVQGFRWAIDNNKWQARVDSIKPLFEEARIDLG-------------KSEIDTEV
         At1g20380 GGSNGGILVGACINQRPDLFGCALAHVGVMDMLRFHKFTIG---HAWTSEFGCS--------------------------
  (S9A) At1g76140 GGSNGGLLVGACINQRPDLYGCALAHVGVMDMLRFHKFTIG---HAWTSDYGCS--------------------------
 (S9A)Os01g01830 GGSNGGLLIAACVNQRPDLFGCALAHVGVMDMLRFHKFTIG---HAWTTDYGCS--------------------------
         Os04g47360 GASNGGLLVAACINQRPDLFGCALAHVGVMDMLRFHKFTIG---RAWTCDFGCS--------------------------
         At1g50380 GRSAGGLLMGAVVNMRPDLFKVVIAGVPFVDVLTTMLDPTIPLTTSEWEEWGDP--------------------------
         Os06g51410 GRSAGGLLMGAVLNMRPDLFKAAVAGVPFVDVLTTMLDPTIPLTTSEWEEWGDP--------------------------
         At1g69020 GYSAGAILPAAAMNMHPSLFQAVILKVPFVDVLNTLSDPNLPLTLLDHEEFGNP--------------------------
         Os09g29950 GCSAGGLLVGAVINMLPDLFSAAVLKVPFLDICNTMMDSTLPLTILDYEEFGDP--------------------------
         At5g66960 GYSAGGLVVASAINHCPDLFQAAVLKVPFLDPTHTLIYPILPLTAEDYEEFGYP--------------------------
         Os09g28040 GYSAGGLLVASAINTRPDLFRAVVLKVPFLDVCNTLLHPILPLTAIDYEEFGFP--------------------------
         Os10g28020 GGSHGGFLTTHLIGQAPGTFVAAAARNPVCNLSLMVGTTDIPEWCFVEIYGKEGKNCFS--------------------E
         Os10g28030 GISHGGFLTTHLIGQAPDRFMVAAARNPVCNLSLMIGTTDIPDWCYAVACGSEGRQHAS--------------------E
         At4g14570 GGSHGGFLTTHLIGQAPDKFVAAAARNPVCNMASMVGITDIPDWCFFEAYGDQ--SHYT--------------------E
         At5g36210 GGSAGGYTTLASLAFR-DVFKAGASLYGVADLKMLKEEGHKFESRYIDNLVGD---------------------------
         Os06g11180 GRSAGGYTTLASLAFR-DTFKAGASLYGIGDLSLLRAETHKFESHYTDNLVGN---------------------------
         Os06g11190 GESAGGFTTLACLAFR-QIFKAGSSLYGIADLASLRAGMHKFEAYYIDNLVGN---------------------------
  (S9B) At5g24260 GWSYGGYLSATLLTRYPEIFNCAVSGAPVT-------SWDGYDSFYTEKYMGLP--------------------------
         Os02g18850 GWSYGGFLSAMCLARFPDTFSCAVSGAPVT-------AWDGYDTFYTEKYMGLP--------------------------
  (S9D) At2g47390 GHSYGAFMTANLLAHAPHLFACGIARSGAYN----RTLTPFGFQNEDRTLWEAT--------------------------
 (S9D)Os03g19410 GHSYGAFMTANLLAHAPHLFCCGIARSGAYN----RTLTPFGFQNEDRTLWEAT--------------------------
 (S9D)Os07g48970 GHSYGAFMTANLLAHAPHLFCCGIARSGAYN----RTLTPFGFQKEVRTLWEAT--------------------------
         At1g26120 GQSAGAHIAACTIVEQVIKESGEGDSVSWSSSQINAYFGLSGGYNLLNLVDHFHSRGLYRSIFLS---------------
         At5g15860 GQSAGAHIAACALLEQATKELK-GESISWTVSQIKAYFGLSGGYNLYKLVDHFHNRGLYRSIFLS---------------
         Os10g04620 GQSMGGAVALKIHFKQPNEWDGAILVAPMCKIADDVIPPWPVQQVLIFMARLLPKEKLVPQKDLAELAFKE-KKKQEQCS
         At5g19630 GSSAGAPIAGSAVEQVEQVVGYVSLGYPFGLMASILFGRHHKAILSSPK-------------------------------
         Os01g42690 GFSMGAAAALHSAACYAHGRFTNGVAYPVTLSAVIGLSGWLPCSRTLKSKMDSS--------------------------
         Os01g57770 GHSLGGINVALAAELFPDKIAAAVFLCAFMPDHTSRPSHVLEKFIEGKWLDWMD--------------------------
```

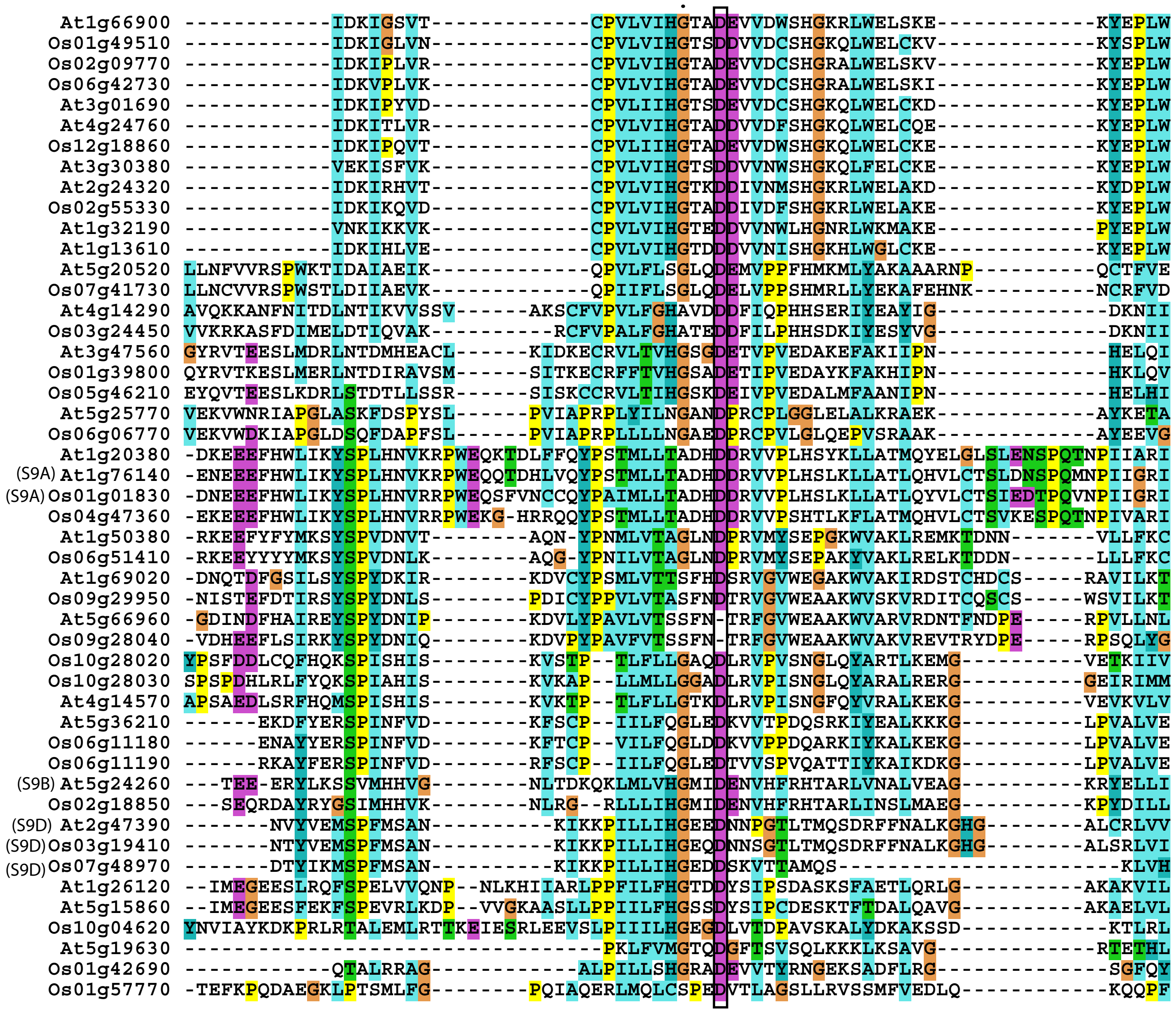
At1g66900 ------------IDKIGSVT-------------CPVLVIHGTADEVVDWSHGKRLWELSKE-------------KYEPLW
Os01g49510 ------------IDKIGLVN-------------CPVLVIHGTSDDVVDCSHGKQLWELCKV-------------KYSPLW
Os02g09770 ------------IDKIPLVR-------------CPVLVIHGTADEVVDCSHGRALWELSKV-------------KYEPLW
Os06g42730 ------------IDKVPLVK-------------CPVLVIHGTADEVVDCSHGRALWELSKI-------------KYEPLW
At3g01690 ------------IDKIPYVD-------------CPVLIIHGTSDEVVDCSHGKQLWELCKD-------------KYEPLW
At4g24760 ------------IDKITLVR-------------CPVLVIHGTADDVVDFSHGKQLWELCQE-------------KYEPLW
Os12g18860 ------------IDKIPQVT-------------CPVLIIHGTADEVVDWSHGKQLWELCKE-------------KYEPLW
At3g30380 ------------VEKISFVK-------------CPVLVIHGTSDDVVNWSHGKQLFELCKE-------------KYEPLW
At2g24320 ------------IDKIRHVT-------------CPVLVIHGTKDDIVNMSHGKRLWELAKD-------------KYDPLW
Os02g55330 ------------IDKIKQVD-------------CPVLVIHGTADDIVDFSHGKRLWELAKE-------------KYEPLW
At1g32190 ------------VNKIKKVK-------------CPVLVIHGTEDDVVNWLHGNRLWKMAKE-------------PYEPLW
At1g13610 ------------IDKIHLVE-------------CPVLVIHGTDDDVVNISHGKHLWGLCKE-------------KYEPLW
At5g20520 LLNFVVRSPWKTIDAIAEIK-------------QPVLFLSGLQDEMVPPFHMKMLYAKAAARNP----------QCTFVE
Os07g41730 LLNCVVRSPWSTLDIIAEVK-------------QPIIFLSGLQDELVPPSHMRLLYEKAFEHNK----------NCRFVD
At4g14290 AVQKKANFNITDLNTIKVVSSV------AKSCFVPVLFGHAVDDDFIQPHHSERIYEAYIG--------------DKNII
Os03g24450 VVKRKASFDIMELDTIQVAK----------RCFVPALFGHATEDDFILPHHSDKIYESYVG--------------DKNII
At3g47560 GYRVTEESLMDRLNTDMHEACL------KIDKECRVLTVHGSGDETVPVEDAKEFAKIIPN--------------HELQI
Os01g39800 QYRVTKESLMERLNTDIRAVSM------SITKECRFFTVHGSADETIPVEDAYKFAKHIPN--------------HKLQV
Os05g46210 EYQVTEESLKDRLSTDTLLSSR------SISKCCRVLTIHGSKDEIVPVEDALMFAANIPN--------------HELHI
At5g25770 VEKVWNRIAPGLASKFDSPYSL------PVIAPRPLYILNGANDPRCPLGGLELALKRAEK-------------AYKETA
Os06g06770 VEKVWDKIAPGLDSQFDAPFSL------PVIAPRPLLLLNGAEDPRCPVLGLQEPVSRAAK-------------AYEEVG
At1g20380 -DKEEEFHWLIKYSPLHNVKRPWEQKTDLFFQYPSTMLLTADHDDRVVPLHSYKLLATMQYELGLSLENSPQTNPIIARI
(S9A) At1g76140 -ENEEEFHWLIKYSPLHNVKRPWEQQTDHLVQYPSTMLLTADHDDRVVPLHSLKLLATLQHVLCTSLDNSPQMNPIIGRI
(S9A) Os01g01830 -DNEEEFHWLIKYSPLHNVRRPWEQSFVNCCQYPAIMLLTADHDDRVVPLHSLKLLATLQYVLCTSIEDTPQVNPIIGRI
Os04g47360 -EKEEEFHWLIKYSPLHNVRRPWEKG-HRRQQYPSTMLLTADHDDRVVPSHTLKFLATMQHVLCTSVKESPQTNPIVARI
At1g50380 -RKEEFYFYMKSYSPVDNVT--------AQN-YPNMLVTAGLNDPRVMYSEPGKWVAKLREMKTDNN-------VLLFKC
Os06g51410 -RKEEYYYYMKSYSPVDNLK--------AQG-YPNILVTAGLNDPRVMYSEPAKYVAKLRELKTDDN-------LLLFKC
At1g69020 -DNQTDFGSILSYSPYDKIR--------KDVCYPSMLVTTSFHDSRVGVWEGAKWVAKIRDSTCHDCS-----RAVILKT
Os09g29950 -NISTEFDTIRSYSPYDNLS--------PDICYPPVLVTASFNDTRVGVWEAAKWVSKVRDITCQSCS-----WSVILKT
At5g66960 -GDINDFHAIREYSPYDNIP--------KDVLYPAVLVTSSFN-TRFGVWEAAKWVARVRDNTFNDPE-----RPVLLNL
Os09g28040 -VDHEEFLSIRKYSPYDNIQ--------KDVPYPAVFVTSSFN-TRFGVWEAAKWVAKVREVTRYDPE-----RPSQLYG
Os10g28020 YPSFDDLCQFHQKSPISHIS--------KVSTP--TLFLLGAQDLRVPVSNGLQYARTLKEMG----------VETKIIV
Os10g28030 SPSPDHLRLFYQKSPIAHIS--------KVKAP--LLMLLGGADLRVPISNGLQYARALRERG----------GEIRIMM
At4g14570 APSAEDLSRFHQMSPISHIS--------KVKTP--TLFLLGTKDLRVPISNGFQYVRALKEKG----------VEVKVLV
At5g36210 ------EKDFYERSPINFVD--------KFSCP--IILFQGLEDKVVTPDQSRKIYEALKKKG----------LPVALVE
Os06g11180 ------ENAYYERSPINFVD--------KFTCP--VILFQGLDDKVVPPDQARKIYKALKEKG----------LPVALVE
Os06g11190 ------RKAYFERSPINFVD--------RFSCP--IILFQGLEDTVVSPVQATTIYKAIKDKG----------LPVALVE
(S9B) At5g24260 ---TEE-ERYLKSSVMHHVG--------NLTDKQKLMLVHGMIDENVHFRHTARLVNALVEAG----------KRYELLI
Os02g18850 ---SEQRDAYRYGSIMHHVK--------NLRG--RLLLIHGMIDENVHFRHTARLINSLMAEG----------KPYDILL
(S9D) At2g47390 -------NVYVEMSPFMSAN----------KIKKPILLIHGEEDNNPGTLTMQSDRFFNALKGHG--------ALCRLVV
(S9D) Os03g19410 -------NTYVEMSPFMSAN----------KIKKPILLIHGEQDNNSGTLTMQSDRFFNALKGHG--------ALSRLVI
(S9D) Os07g48970 -------DTYIKMSPFMSAN----------KIKKPILLIHGEDDSKVTTAMQS-----------------------KLVH
At1g26120 --IMEGEESLRQFSPELVVQNP--NLKHIIARLPPFILFHGTDDYSIPSDASKSFAETLQRLG----------AKAKVIL
At5g15860 --IMEGEESFEKFSPEVRLKDP--VVGKAASLLPPIILFHGSSDYSIPCDESKTFTDALQAVG----------AKAELVL
Os10g04620 YNVIAYKDKPRLRTALEMLRTTKEIESRLEEVSLPIIILHGEGDLVTDPAVSKALYDKAKSSD------------KTLRL
At5g19630 ----------------------------------PKLFVMGTQDGFTSVSQLKKKLKSAVG-------------RTETHL
Os01g42690 ------------QTALRRAG------------ALPILLSHGRADEVVTYRNGEKSADFLRG--------------SGFQY
Os01g57770 -TEFKPQDAEGKLPTSMLFG--------PQIAQERLMQLCSPEDVTLAGSLLRVSSMFVEDLQ------------KQQPF

```
      At1g66900 ISGGGHCDLELYP--DFIRHLKKFVVSLGNK-----
      Os01g49510 LTGGGHCNLELYP--DYIKHLKKFVSSLGKK-----
      Os02g09770 VKGGNHCNLELYP--EYIKHLKKFVGAIEKS-----
      Os06g42730 VKGGNHCNLELYP--EYIKHLKKF------------
      At3g01690 VKGGNHCDLEHYP--EYIRHLKKFIATVERLP----
      At4g24760 LKGGNHCDLELFP--EYIGHLKKFVSAVEKSA----
      Os12g18860 LKGGKHCDLELFP--EYLRHLKKFVNTVEKSP----
      At3g30380 IKGGNHCDLELYP--QYIKHLRKFVSAIEKS-----
      At2g24320 VKGGGHCNLETYP--EYIKHMRKFMNAMEKLA----
      Os02g55330 VKGGGHCNLETYP--EYIRHLRKFINAMEKLSKD--
      At1g32190 IKGGGHCNLEIYP--DYIRHLYRFIQDMENT-----
      At1g13610 LKGRGHSDIEMSP--EYLPHLRKFISAIEKL-----
      At5g20520 FPSGMHMDTWLSGGEVYWKTNLQFLEKYAPEK----
      Os07g41730 FPNGMHMDTWNSGGDRYWRTIQLFLDQYAPEV----
      At4g14290 KFDGDHNSPRPQF---YFDSINIFFHNVLQPP----
      Os03g24450 KFDGDHNSPRPQF---YFDSITIFFHNVLNPP----
      At3g47560 VEGADHCYTNYQS--QLVLTVMEFIKSHCEEK----
      Os01g39800 IEGANHNYTAHRE--ELADAVVDFITSN--------
      Os05g46210 IAEANHRYTGHEK--ELKAFVLDFIKSQPNFS----
      At5g25770 SPGNFKFKAEDGVGHEATSFMIKESSDWFDKFLKQE
      Os06g06770 SADKFMFIAEPGIGHQMTANMVKEASDWFDR-----
      At1g20380 EVKAGHGAGRPTQKMIDEAADRYSFMAKMVDAS---
(S9A) At1g76140 EVKAGHGAGRPTQKMIDEAADRYSFMAKMVNAS---
(S9A) Os01g01830 DVKSGHGAGRPTKKMIDEVADRYSFMANMLDAS---
      Os04g47360 DRKSGHGCGRSTQKIIDEAADRYAFAAKTMGIS---
      At1g50380 ELGAGHFSKSGRFEKLQEDAFTFAFMMKVLDMI---
      Os06g51410 ELGAGHFSKSGRFEKLREDAFTYAFILKALGMA---
      At1g69020 NMNGGHFGEGGRYAQCEETAFDYAFLLKVMGHH---
      Os09g29950 NMQSGHFGEGGRFMHCDETAFEYAFLMKALGLD---
      At5g66960 TTDIVEEN---RFLQTKESALEIAFLIKMMES----
      Os09g28040 LMIWKPKF---LSTGDRLTLIKSVLFALPVHYLSVL
      Os10g28020 FPEDMHGLDKPQSDFESFLNIGVWFKKHMS------
      Os10g28030 FPDDIHEINIPQSDFESFLNIGVWFKKHLSIS----
      At4g14570 FPNDNHPLDRPQTDYESFLNIAVWFNKYC-------
      At5g36210 YEGEQHGFRKAENIKYTLEQQMVFFARVVGGF----
      Os06g11180 YEGEQHGFRKAENIKFTLEQQMVFFARLVGNF----
      Os06g11190 YEGEQHGFRKAENIKFTLEQQMVFFARLVGHF----
(S9B) At5g24260 FPDERHMPRKKKDRIYMEQRIWEFIEKN--------
      Os02g18850 FPDERHMPRRLGDRIYMEERIWDFVERN--------
(S9D) At2g47390 LPHESHGYSARESIMHVLWETDRWLQKYCVPNL---
(S9D)Os03g19410 LPFESHGYSARESIMHVLWETDRWLQKYCLSG----
(S9D)Os07g48970 LAFAQIFHQEVKK-----------------------
      At1g26120 YEGKTHTDLFLQDPMRGG------------------
      At5g15860 YSGKTHTDLFLQDPLRGG------------------
      Os10g04620 YKDAYHAILEGEPDEAIFQVLDDIISWLDQHSTKK-
      At5g19630 IEGVSHFQMEGPEYDSQVTDIICKFISSL-------
      Os01g42690 LNFKPYNGLGHYTIPEEMDDVCKWLSSRLGLD----
      Os01g57770 TEGRYGSVRKVYVVVNQDLAIPEGFQRWMIGNS---
```
