# Supplementary material for: Cross genome comparisons of serine proteases in Arabidopsis and rice
Source: BMC Genomics. 2006 Aug 9;7:200. doi: 10.1186/1471-2164-7-200 (PMC1560137; doi:10.1186/1471-2164-7-200)
Supplement: Additional file 8 — Figure SF4. Multiple sequence alignment and phylogenetic analysis of Arabidopsis and rice serine carboxypeptidase-like proteins A: Multiple sequence alignment of Arabidopsis and rice serine carboxypeptidase-like proteins. Multiple sequence alignment of the serine carboxypeptidase domain region of the annotated Arabidopsis and rice serine carboxypeptidase-like proteins. The catalytic triad residues are indicated. Gene names correspond to those in Additional files 1 and 2. For brevity, rice gene names have been shortened to OsXXg##### instead of LOC_OsXXg#####, XX referring to chromosome 1–12 and a 5 digit number assigned to each gene. B: Phylogenetic analysis of Arabidopsis and rice serine carboxypeptidase-like proteins. Unrooted N-J tree computed from multiple sequence alignments of Arabidopsis (red) and rice (blue) serine carboxypeptidase domains. Serine carboxypeptidase-like protease domains were aligned using ClustalW[95] program and the alignments were exported to Phylip package[96] for representing the Neighbor-Joining tree (see methods). The colors and circles represent different evolutionary clades identified in the analysis (see text for details). Clade I is represented in orange, Clade II is shaded green while the branches connecting the remaining sequences are labelled in purple. For clarity, bootstrap values were replaced with symbols representing bootstrap percentages >50%. Bootstrap values between 50–60% are represented by an asterix, circles represent bootstrap values from 60%–80% while bootstrap values >80% are represented by rectangles. Gene names correspond to those listed in Tables 2 and 3. For brevity, rice gene names have been shortened to OsXXg##### instead of LOC_OsXXg#####, XX referring to chromosome 1–12 and a 5 digit number assigned to each gene. [file 1471-2164-7-200-S8.pdf]

a)

```
Os01g11670 ----------FEALPTKSGYLPIPTAN-ASLFFAYYEATHLLT----PPASTPIILWLQGG----------PGCSGLTGN
Os05g50600 ---------PKEALPTNSGYLPITTTN-ASLFFAYYEATHPLT----PPASTPLLLWLQGG----------PGCSGLAGN
Os06g36570 --------------------------------------------------------------------------------
Os05g50570 ---------PRGAIPTKSGYLPIPPAN-ASLYFAFYEATEPVT----PPATTPLLVWLEGG----------PGCSGFLSN
Os05g50580 ---------PRGALPTKSGYLPIPPAANASLYFAFYEATEPVT----PLATTPLLVWLEGG----------PGCSGFLSN
Os01g43890 ---------PEEARPTRSGYLNVTSTN--SLYFAFYEATDPVTT---QPAAVPLLVWLQGG----------PGCSSLIGS
At1g15000 ------PLFPDEALPTKSGYLPVKPAPGSSMFYAFYEAQEPTT----PLPDTPLLVWLQGG----------PGCSSMIGN
At1g11080 ------PGQPDVSFRHYAGYVPVDESNGRAMFYWFFEAMDLPK-------EKPLVLWLNGG----------PGCSSVGYG
At4g15100 ------------------------------------------------------------------------GCSSVGYG
At1g61130 ------PGQPKVSFRHYAGYVTVNIISGRALFYWFFEAMTHPN-------VKPLVLWLNGG----------PGCSSVGYG
Os01g22980 -----PGQPRSPQMSQFSGYITVNSQNGRALFYWFFEAQALPS-------KKPLLLWLNGG----------PGCSSVGYG
Os05g06660 -----PGQPASPAVSQFAGYVGVDERHGRALFYWFFEAQASPAP-----EKKPLLLWLNGG----------PGCSSIGYG
At3g17180 -----PEQPLNPKISHFSGYVNVNQENTRSLFFWFFEALSESP------STRPLVLWLNGG----------PGCSSIGYG
At5g08260 ----PGQPPVN-FKHYAGYVNLGPEQKQKALFYWFFEAQQNSS-------RRPLVLWLNGG----------PGCSSIAYG
Os04g44410 ----PGQPDVR-FRHYAGYVGVG---NGKALFYWFFEAEKEPE-------KKPLLLWLNG--------------------
At5g23210 ---------------RPGCSSIGFGAAEELGPFFPQNSSQPKL-------KLNPYSWNKA--------------------
Os02g42310 --------------------------------------------------------------------------------
Os10g39560 ----PGQPAYSPEFRQYSGYVTTDEYLGKALFYWFLEATDKPD-------EKPLVLWLNGG----------PGCSSIGFG
Os06g51370 -----PGQPAELGFRQFAGYVTVNETHGRALFYWFFEAASDVA-------TKPLVLWLNGG----------PGCSSLGYG
At2g24000 ------PGQPKVGFSQFSGYVTVNESHGRSLFYWLTESS-SHSP-----HTKPLLLWLNGGWIFFLPTFPRPGCSSIAYG
At2g24010 ------PGQPQVGFSQFSGYVTVNESHGRSLFYWLTESP-SSS------HTKPLLLWLNG----------GPGCSSIGYG
At4g30610 ------PGQPKVAFSQYSGYVNVNQSHGRALFYWLTESS-SPSP-----HTKPLLLWLNG----------GPGCSSIAYG
Os02g55130 -----------------------------------------------------------------------PGCSSIAYG
At3g02110 ------PGQPNVTFEQFSGYVTVDKLSGRSLFYWLT-EA-SDLP-----LSKPLVIWLNG----------GPGCSSVAYG
At3g07990 -----PGQPSNVDFRQYSGYVTVHEERGRALFYWLVESPLARDP-----KSRPLVLWLNG----------GPGCSSVAYG
Os01g61690 -----PGQPAKVRFSQYSGYVTVNQAHGRALFYWLVEAVPAAGP------IAPLVLWLNG----------GPGCSSVGYG
At2g35780 -----PGEPNDVSFSHFSGYITVNESAGRALFYWLTESPPSENP-----ESKPLVLWLNG----------GPGCSSVAYG
Os01g06490 -----PGQP-RVNFSMYSGYVTVDAAAGRALFYWLIEA---ADP-----ASAPLVLWLNG----------GPGCSSVGYG
At4g30810 -----PGQNFNVSFAHYSGFVATNEQLGRALFYWLFEA--VEDA-----KSKPLVLWLNG----------GPGCSSVAYG
Os06g08720 -----PGQDFDVGFAQYAGYVAVSEERGASLFYWFFEA--ADDP-----ASKPLVLWLNG----------GPGCSSIAYG
At2g35770 -----PGQPPNLNFSQFSGYVTVDPAAGRALFYWLTEAP--RPS-----GTKPLVLWLNG----------GPGCSSIAYG
Os09g28830 -----PGQPKGVGFNQYG--------------------------------------------------------------
Os09g28840 -----PGQPCGVNFAQFAGYVTVDRKNGRELFYYFVESPYDASTKPLILWLNGVVLIEFIG----------PGCSSLGFG
Os07g46350 -----PGQPAGVGFAQYAGYVTVDAAAGRALFYYLAEADGG----AAASSKAPLLLWLNGG----------PGCSSLGYG
Os03g26920 -----PGQPGRAGFDQYAGYVTVNATSGKALFYYFAEATDD-------PSTKPLVLWLNGG----------PGCSSLGDG
Os03g26930 -----PGQMEEAEFNQYAGYVTVDAKAGRALFYYFVEAPHD-------PLKKPLVLWLNGG----------PGCSSFGAG
Os03g09190 -----PGQPPDVKLQQYSGYINVNETSGKSLFYYFVEASVD-------AAHKPLLLWLNGG----------PGCSSMGIG
At3g52000 -----PGQPSDVTFKQYGGYVAVNKPAGRFLYYYFVETIKP-------GNTTPLVIWFNGG----------PGCSSLGG-
At3g52010 -----PGQPSGVSFRQYGGYVPVNEPSSRFLYYYFVEAIKP-------NTSTPLVIWFNGG----------PACSSLGG-
At2g05850 -----PGQPSGISFRQYGGYVAVNEPATRFLYYYFVEAIKP-------SKSTPLVLWFNGG----------PGCSSVGFG
At3g52020 -----PGQPS-VSFRQYGGYVTVNESAGRSLYYYFVEATKT-------KKSLPLVLWLNGG----------PGCSSLYG-
At3g63470 -----PGQPP-VSFDQYGGYVTVNESAGRSFFYYFVEASKS-------KDSSPLLLWLNGG----------PGCSSLAYG
At1g28110 ------PGQPRVGFQQYSGYVTVDDKKQRALFYYFAEAETNPSS-------KPLVLWLNGG----------PGCSSLGVG
At2g33530 ------PGQPRVGFQQYSGYVTIDEKKQRALFYYLAEAETKPIS-------KPLVLWLNGG----------PGCSSLGVG
Os04g32540 ------PGQPNVSFAQYSGYVTVDAARRRELFYYFAEAELDPAT-------KPLVLWLNGG----------PGCSSVGVG
Os11g31980 ------PGQPEVSFGQYAGYVGVDDKGRRALFYYFVEAELDPAT-------KPLVLWLNGG----------PGCSSLGVG
At2g12480 ------PGQPNVGFRQFAGYVDVDSENGRSLFYYYVEAVKEPDT-------KPLTLWLNGG----------PGCSSVGGG
At5g42240 ------PGQPTVGFKQYAGYVDVDVKAGRSLFYYYVEAVKQPDS-------KPLTLWLNGG----------PGCSSIGGG
At5g42230 ------PGQPKVVFRQYAGYVDLDLNAGRSLFYYFVEAEKHPDT-------KPLTLWLNGG----------PGCSSVGGG
At1g43780 ------PGQPEVAFRQFAGYVDIDVKAGRSLFYYFVEAEKQPHS-------KPLTLWLNGG----------PGCSSIGGG
Os11g10750 ------PGQPVVGFRQFAGYVDVDEKAGRSLFYYFAEAAEGAAA-------KPLTLWLNGG----------PGCSSVGGG
At1g73300 -----PGFEGPLPFELETGYIGIGEE-EEVQLFYYFIKSER------NPKEDPLILWLTGG----------PGCSSISGL
At5g36180 -----PGFEGQLPFELETGYIGVGEE-EEVQLFYYFIKSER------NPKEDPLILWLTGG----------PGCSAISGL
At1g73280 -----PGFEGPLPFELETGYIGVGEE-EEVQLFYYFIKSER------NPKEDPLLLWLSGG----------PGCSSISGL
At1g73310 -----PGFEGPLPFELETGYIGVGEE-EEVQLFYYFIKSER------NPKEDPLLLWLTGG----------PGCSAISGL
At1g73290 -----PGFEGSLPFELETGYIGIGEE-EEVQLFYYFIKSER------NPKEDPLLLWLSGG----------PGCSSISGL
At1g73270 -----PGFEGPLPFELETGYIGVGEE-EEVQLFYYFIKSER------NPKEDPLLLWLTGG----------PGCSAISGL
At3g10450 -----PGFDGPLPFELETGYIGVGEE-EEVQLFYYFIKSER------NPQEDPLLLWLSGG----------PGCSSISGL
At2g22920 -----PGFEGPLPFELETGYIGIGEE-EDVQLFYYFIKSER------NPKEDPLLLWLSGG----------PGCSSITGL
At2g22970 -----PGFEGPLPFELETGYIGIGEE-EEVQLFYYFIKSER------NPKEDPLLLWLSGG----------PGCSSITGL
At2g23000 -----PGLEGRLPFELETGYIGIGEE-EDIQLFYYFIKSEN------NPKEDPLLLWLDGG----------PGCSSLGGL
At2g23010 -----PGFKGPLPFELETGYIGIGEE-ENVQFFYYFIKSDK------NPQEDPLIIWLNGG----------PGCSCLSGL
At2g22990 -----PGFEGPLPFELETGYIGIGED-ENVQFFYYFIKSEN------NPKEDPLLIWLNGG----------PGCSCLGGI
At2g22980 --------------------------------------------------------------------------------
At3g12230 -----PGFEGPLPFELETGYIGVGDE-DEDQMFYYFIKSES------NPEEDPLLVWLSGG----------PGCSSFTGL
At3g12240 -----PGFEGPLPFELETGYIGVGQK-EEDQLFYYFIKSEN------NPEEDPLLVWLTGG----------PGCSSFSGL
At3g12220 -----PGFEGPLPFELETGYIGVGEE-DEDQMFYYFIKSES------NPKTDPLLLWLSGG----------PGCSSFTGL
At3g12203 -----PGFQGPLPFELETGYIGVGEA-EKDQMFYYFIKSES------NPEKDPLLLWLSGG----------PFCSSFTAL
At1g33540 -----PGFEGLLPFHLETGYIGVGEG-EKVQLFYYFIKSEN------NPEEDPLIIWLTGG----------PACTALSAL
At5g09640 -----PGFEGPLPFELETGYVSIGES-GDVELFYYFVKSER------NPENDPLMIWLTGG----------PGCSSICGL
Os10g01110 -----PGFDGALPFELETGYVEVDRI-AGVRLFYYFIRSES------SPADDPLLLWLTGG----------PGCSAFSGL
Os11g42390 -----PGFDGPLPFELETGYVEVDHI-AGVRLFYYFIRSERRP----AADDDPLLLWLTGG----------PGCSAFSGL
Os04g09720 -----PGFAGPLPFSLETGYVAVG----EARFFYYFIESER------SPEEDPVLLWLTGG----------PGCSAFSGL
Os02g46260 -----PGFDGPLPFYLETGYVGVEEE-TGTELFYYFVESE--R----SPRSDVVLLWLSGG----------PRCSVFSGF
Os03g52080 -----PGFHGRLRFYLETGYVSVDEE-TGTELFYYFVESE--R----SPSTDPVILWLTGG----------PLCSGFTAL
Os11g24510 -----PGLDGALPSRFETGYVTVDEE-NGGELFYYFIESE--G----DPGADPVLLWINGG----------NRCSVLSAL
Os12g39170 -----PGYDGELPFRLETGYVAVDEEEHGAELFYYFVESES-G----DPRRDPLLLWLTGG----------ARCSVLSGV
Os10g01130 ---------------------------------------EEEA----DTMTRWLAELVALN----------VAGQQRIAS
Os11g27200 --------------------------------------------------------------------------------
Os02g26480 -----PGFDGDLPSKHYAGYITVGHQPLKRHMYYYFATSER------NSTTDPVIIWINGG----------PACSGFSAF
At3g25420 -----PGFNGTFPSKHYAGYVAIDKH-RNKNLWYYFVESER------NASVDPVVLWLNGG----------PGCSSMDGF
At4g12910 -----PGFEGTFPSKHYSGYVTIDKE-HGKNLWYYFIESEK------NPSKDPVVLWLNGG----------PGCSSMDGF
Os04g25560 -----PGFHGAFPSKHYSGYVTVDEG-SGRRLFYYLVTSER------DAAADPVVLWLNGG----------PGCSSLDGF
Os12g15470 -----PGFDGALPSKHYAGYVTVEEQ-HGRNLFYYLVESER------DPAKDPLVLWLNGG----------PGCSSFDGF
Os02g02320 ----PGLPQGVGDLGHHAGYYRLPNTHDARMFYFLFESRGKK--------EDPVVIWLTGG----------PGCSSELAV
Os07g29620 ------GGSSAEDLGHHAGYYRLPNTHDARLFYFFFESRGSKGE------DDPVVIWLTGG----------PGCSSELAL
At3g10410 PNILADGGPTVDDLGHHAGYYKLPKSRGASMFYFFFESRNKK--------DAPVVIWLTGG----------PGCSSELAV
At3g45010 --------PSVQDFGHHAGYYKLPNSKAARMFYFFFESRTNK--------ADPVVIWLTGG----------PGCSSELAL
At5g22980 --------PSVQEFGHYAGYYSLPHSKSAKMFYFFFESRNKT--------TDPVVIWLTGG----------PGCSSSVAM
Os03g27480 --------GGTNDGSERWGYVQVRPKAHLFWWYYRSPQRVSSPG----GKPWPTILWLQGG----------PGASGVGLG
Os08g44640 -------AGGTPDGSEEWGYVQVRPKAHMFWWLYRSPQRVNNKG----STPWPTVLWLQGG----------PGASGVGYG
At2g27920 -------------GSEAWGYVEVRPKAHMFWWHYKSPYRVENP-----SKPWPIILWLQGG----------PGASGVGIG
Os03g27510 --------------------------------------GISRTT----RWVWATATPTTGA----------GGLATT---
Os03g27530 -------------------------------------------------------PERAGD----------DGLAGG---
```

```
Os01g11670 FFELGP---------------------------YFVNHDALSLSPNPFAWNRRFGLLFIDNPLGTGFSAAPSPAA----I
Os05g50600 FFELGP---------------------------YFVNRDALSLSPNPFSWNRRFGLLFIDNPLGTGFSAAPSPAA----I
Os06g36570 -------------------------------------------------------------------------------F
Os05g50570 FLQIGP---------------------------YLLAG--GSLSPNPFAWNRRFGLLFIDSPLGTGFSVAPSPAA----I
Os05g50580 FLQIGP---------------------------YLFAG--GSLSPNPFAWNRRFGLLFIDSPLGTGFSVAPSPAN----I
Os01g43890 FAELGP---------------------------YLLLDSTSALARNDNRWNRRFGVIFIDNPLGAGFSAPASGDD----I
 At1g15000 FYELGP---------------------------WRVVSRATDLERNPGAWNRLFGLLFVDNPIGVGFSIAASQQD----I
 At1g11080 ATQEIG--------------------------PFLVDTNGNGLNFNPYAWNKEANMLFLESPVGVGFSYSNTSSD---YQ
 At4g15100 ATQEIG--------------------------PFLADTNEKGLIFNPYAWNKEVNMLFLESPVGVGFSYSNTSSD---YL
 At1g61130 ATQEIG--------------------------PFLVDNKGNSLKFNPYAWNKEANILFLESPAGVGFSYSNTSSD---YR
Os01g22980 AASELG--------------------------PLMVNGNGTGLEFNKFAWNNEANLLFLESPVGVGFSYTNTSSD---LE
Os05g06660 AASELG--------------------------PLRVARQGAALEFNQYGWNKEANLLFLESPVGVGFSYTNTSSD---LS
 At3g17180 AASELG--------------------------PFRVVENGTSLSFNQYSWVQEANMLFLESPVGVGFSYTNSSSD---LE
 At5g08260 AAQELG--------------------------PFLVHDNGGKLTYNHFSWNKEANMLFLEAPVGVGFSYTNNSMD---LQ
Os04g44410 ----------------------------------------------------AVNLLFLEAPVGVGFSYTNRTSD---LR
 At5g23210 -----------------------------------------------------ANLLFLESPVGVGFSYTNTSRD---IK
Os02g42310 ---------------------------------------------------------MFLESPVGVGFSYTNTSSD---LQ
Os10g39560 QAQELG--------------------------PFLVKKDVAELELNPYAWNQVANLLFLDSPAGVGFSYTNTSFG---KD
Os06g51370 ALEELG--------------------------PLLVNNN-DTLIINPESWNKEANLLFVESPAGVGFSYTNTTTD---LA
 At2g24000 ASEEIG--------------------------PFRISKTGCNLYLNNFSWNTEANLLFLESPVGVGFSYTNTSSD---FE
 At2g24010 ASEEIG--------------------------PFRINKTGSNLYLNKFTWNTEANILFLESPAGVGFSYTNTSSD---LK
 At4g30610 ASEEIG--------------------------PFRINKTGSNLYLNKFAWNKDANLLFLESPAGVGYSYTNTSSD---LK
Os02g55130 ASEEIG--------------------------PFRIKTNGTGLYLNKYSWNREANLLFLESPAGVGFSYSNTTSD---LK
 At3g02110 ASEEIG--------------------------PFRISKGGSGLYLNKFAWNSISNLLFLEAPAGVGFSYTNRSSD---LF
 At3g07990 AAEEIG--------------------------PFRVGSDGKTLHSKLYAWNKLANLLFLESPAGVGFSYSNTTSD---LY
Os01g61690 ASEEVG--------------------------PFRIRPDGKTLYLNPNSWNKAANLLFLESPAGVGFSYSNKTLD---LY
 At2g35780 AAEEIG--------------------------PFRINPDGKTLYHNPYSWNKLANLLFLESPAGVGFSYSNTTSD---LY
Os01g06490 ASEELG--------------------------AFRINPDGRSLYLNPYPWNRVANMLFLDSPAGVGYSYSNTTSD---LF
 At4g30810 EAEEIG--------------------------PFHIKADGKTLYLNQYSWNQAANILFLDAPVGVGYSYSNTSSD---LK
Os06g08720 VAEEVG--------------------------PFHVNADGQGVHLNPYSWNQVANILFLDSPVGVGYSYSNASDD---IL
 At2g35770 ASEEVG--------------------------PFRVNPDGKTLRLNLYAWNKVANVLFLDSPAGVGFSYTNTSSD---EL
Os09g28830 ----------------------------------GINSDNKTLSRNEYAWNNVANVLFLESPAGVGFSYSNTSS---DYD
Os09g28840 AMKELG--------------------------PFRVNPDGKTLSRNKHAWNNVANVIFLESPAGVGFSYSMNSS---DYS
Os07g46350 AMEELG--------------------------PFRVKSDGVSLYRNPYSWNNVANVMFLESPIGVGFSYSNTTA---DYS
Os03g26920 AMLEIG--------------------------PFLVNGDNRTLSINRYAWNNVANMLFLESPAGVGFSYSNTTS---DYD
Os03g26930 AMLELG--------------------------PFSVRSDNKTLYNKQHAWNTVANMLFVDVPAGVGYSYSNTTS---DYY
Os03g09190 AFQEIG--------------------------PFRVDTDGKTLCRNPHSWITAANLLFLESPVGVGFSYAAVKPQ-EVYS
 At3g52000 AFKELG--------------------------PFRVHSDGKTLFRNPYSWNNEANVLFLETPVGTGFSYSNSPIN----G
 At3g52010 AFLELG--------------------------PFRVHSGGRKLFRNPYSWNNEANVLFLESPVTTGFSYSSNPIDLEELG
 At2g05850 AFEELG--------------------------PFRVHSDGKTLYRNPYSWNNEANMLFFEGPISVGFSYSSTPFDWEIFG
 At3g52020 AFQELG--------------------------PFRIYGDGKTLYTNPYSWNNVANILFLESPVGTGFSYTNTESDLE---
 At3g63470 ALQELG--------------------------PFRVHSDGKTLFRNRYAWNNAANVLFLESPAGVGFSYTNTTSDLEK--
 At1g28110 AFSENG--------------------------PFRPKGP--ILVKNQHSWNQEANMLYLETPVGVGFSYSTQSSH---YE
 At2g33530 AFSENG--------------------------PFRPKGS--ILVRNQHSWNQEANMLYLETPVGVGFSYANESSS---YE
Os04g32540 AFSENG--------------------------PFRPSGN--VLVRNEYSWNKEANMLYLESPAGVGFSYSTDPSF---YG
Os11g31980 AFSENG--------------------------PFRPSGQ--VLVKNEYSWNKEANVIYLETPAGVGYSYSADAAY---YQ
 At2g12480 AFTELG--------------------------PFYPTGDGRGLRLNSMSWNKASNLLFVESPAGVGWSYSNRSSD---YN
 At5g42240 AFTELG--------------------------PFYPTGDGRGLRVNSMSWNKASHLLFVESPAGVGWSYSNKSSD---YN
 At5g42230 AFTELG--------------------------PFYPTGYGRGLRINSMSWNKASNLLFVDSPAGVGWSYSNRSSD---YN
 At1g43780 AFTELG--------------------------PFYPTGDARGLRRNPKSWNKASNLLFVDSPAGVGWSYSNTTSD---YT
Os11g10750 AFTELG--------------------------PFYPRGDGRGLRLNKKSWNKVSNLLFVESPAGVGWSYSNTSSD---YN
 At1g73300 LFENGP---------------------LTMKLD-VYNGTLPSLVSTTYSWTKTSSMIFLDQPVGTGFSYSR----TQQFN
 At5g36180 LFENGP---------------------LTMKLD-VYNGTLPSLVSTTYSWTKTSSIIFLDQPVGTGFSYSR----TQQFN
 At1g73280 LFENGP---------------------LAMKLD-VYNGTLPSLVSTTYSWTKASSMIFLDQPVGAGFSYSR----TQLLN
 At1g73310 LYQNGP---------------------LAMKLD-VYNGTLPSLVSTTYSWTKTSSMIFLDQPVGTGFSYSR----TQLFN
 At1g73290 LFENGP---------------------LAMKLD-VYNGTLPSLVPTTYSWTKTSSMIFLDQPVGTGFSYSR----TQQYN
 At1g73270 LYENGP---------------------LTMKLD-VYNGTLPSLVSTTYSWTKNSSMIFLDQPVGTGFSYSR----TELFN
 At3g10450 LYENGP---------------------VNVKIE-VYNGTLPSLVSTTYSWTKVSSIIYLDQPVGTGFSYSR----TKLVN
 At2g22920 LFENGP---------------------LALKSK-VYNGSVPSLVSTTYSWTKTANIIFLDQPIGAGFSYSR----IPLID
 At2g22970 LFQNGP---------------------LALKSE-VYNGSVPSLVSTTYSWTKTANIIFLDQPVGAGFSYSR----APLID
 At2g23000 LFENGP---------------------VALKSA-VYNGSNPSLFSTTYSWTKMANIIYLDQPVGSGFSYSR----TPIGK
 At2g23010 FFENGP---------------------LALKNK-VYNGSVPSLVSTTYSWTKTANIIFLDQPVGSGFSYSK----TPIER
 At2g22990 IFENGP---------------------VGLKFE-VFNGSAPSLFSTTYSWTKMANIIFLDQPVGSGFSYSK----TPIDK
 At2g22980 -----------------------------------------------------MANIIFLDQPVGSGFSYSR----TPLVD
 At3g12230 VYENGP---------------------LGFKVE-AYNGSIPTLVSTTYSWTKVANIIYLDQPVGAGFSYSR----NPFAD
 At3g12240 VYENGP---------------------LAFKVE-TYNGSVPTLVSTTYSWTKVANIIYLDQPVGTGFSYSR----NPFAD
 At3g12220 IYENGP---------------------LGFKVE-AYNGSIPTLVSTTYSWTKVANIIYLDQPVGTGFSYSR----NPLAD
 At3g12203 IYENGP---------------------IAFKAE-EYNGSIPSLVSTTYAWTKVASILYLDQPVGTGFSYSR----NPLAD
 At1g33540 AFEIGP---------------------LTFKTE-GYNGGLPSLVSTSYSWTKVASIIFLDQPVGTGYSYST----TPLSY
 At5g09640 LFANGP---------------------LAFKGD-EYNGTVPPLELTSFSWTKVANILYLEAPAGSGYSYAK----TRRAF
Os10g01110 VYEVGP---------------------LTFDVHGHGHGQLPRLLYKPESWTKRTNVIFLDSPVGTGFSYAD----TDAGF
Os11g42390 VYEVGP---------------------LTFDLHHGRHGGLPRLLYKPESWTKRASVIFLDSPVGTGFSYAADADTDGAGF
Os04g09720 IYEIGP---------------------LFFDFHGHKG-GLPTLHYKANSWTKISNVIFVDSPPGTGFTYAT----TAEGL
Os02g46260 VYEIGP---------------------VMFVAERYSGGTVPRLEYNPYSWTKLASILFVDSPVGSGFSYAH----DPKGY
Os03g52080 VFEVGP---------------------MNFVLAPYNG-SLPRLVNNQYSWTKIASIIFLDTPVGSGFSYAR----DPKGY
Os11g24510 FFEIGENLNHHRSPAGAPPLTRLCAAGPVKLAIEPYDGGVPRLRYNPYTWTKVASVLFVDSPVGAGFSFSR----DPRGY
Os12g39170 FFEVGP------------------VRLALEHHRPYDAGELPRLRYHPHGWTKAASVLFVDSPVGAGWSFSR----HPDGY
Os10g01130 VAETGP----------------------LKFDVAGYTEGFPQLFYFQDSWTKVSNVIFLDAPVGTGFSYAR----EEQGY
Os11g27200 ---------------------------------------------PSPTWRQLT----------CGIKGTP----WEDLR
Os02g26480 LHSIGP---------------------LKIEGPMIHARDEPRTKLNPFSWTKMSSVLLVDSPAGVGYSYSE----NEDDY
 At3g25420 VYEHGP----------------------FNFEPKKKNS--HLLHLNPYSWSKVSNIIYLDSPVGVGFSYSN----DNADY
 At4g12910 VYEHGP----------------------FNFELPKKNNSLPLLHLNPYSWSKVSNIIYLDSPVGVGFSYSN----NKSDY
Os04g25560 VYENGP----------------------FNFERGSDPGGLPNLELNPYSWSKVSNVVYLDSPAGVGMSYSL----NKSDY
Os12g15470 VYEHGP----------------------FNFESGGSAKSLPKLHLNPYSWSKVSSVIYLDSPAGVGLSYSK----NTSDY
Os02g02320 FYENGP----------------------------FTISNNMSLAWNKFGWDTISNIIFVDQPTGTGFSYSSDDRD----T
Os07g29620 FYENGP----------------------------FHIADNMSLVWNDFGWDQESNLIYVDQPTGTGFSYSSNPRD----T
 At3g10410 FYENGP----------------------------FKITSNMSLAWNEYGWDQVSNLLYVDQPVGTGFSYTTDKSD----I
 At3g45010 FYENGP----------------------------FTVSNNSSLSWNEFGWDKASNLIYVDQPVGTGFSYTSDQSD----L
 At5g22980 FYENGP----------------------------FKISKDLSLYWNDFGWDKVSNIIYVDQPVGTGFSYTSDESD----I
Os03g27480 NFLEVG-------------------------------PLDGDLKPRGSTWLQKADLIFVDNPVGTGYSYVEDD----ALL
Os08g44640 NFMEIG-------------------------------PLDTNLKPRPSTWLSKADLLFVDNPVGTGFSYVEGGDR--TLL
 At2g27920 NFQEVG-------------------------------PLDTFLKPRNSTWLKKADLLFVDSPVGAGYSFVEGNQK--DLY
Os03g27510 --------------------------------------------------------------------------------
Os03g27530 --------------------------------------------------------------------------------
```

```
Os01g11670 PTNQFVVAAHLFTALQSFFAL-QPGFRSRPFFLTGESYAGKYVPAAGSYILAVNPTLP----KRLRVNLHGVAIDNGLTH
Os05g50600 PTNQSVVAAHLFAALQSFFAL-QPGSRSRPFFLTGESYAGKYIPAAGSYILAVNPTLP----TRLRVNLHGVAIGNGLTH
Os06g36570 NLLSLSFPSSLLSSYFSPFSD-SAGGRRRA------SAAG-----------DGR--------------------------
Os05g50570 PTNQSVVADHVLAALQSLYSL-EPSFRARPLYLTGESYAGKTIPAAGALIVATNPTLP----EKKRINLRGVAIGNGMTH
Os05g50580 PTNQSVVADHVLAALQSFLSL-EPSFRARPLYLTGESYAGKTIPAAGALIVATNPTLP----EQKRINLRGVAIGNGMTH
Os01g43890 PTDERTIAAHLLAALQSFMAL-DPAFRARPLFLTGESYAGKYIPAAASHILDANAKLT----DDRRVNLQGIAIGNGMTH
 At1g15000 PTNQRQVAEHLYAALVEFLEQ-NPSFENRPVYFTGESYAGKYVPAIGYYILKEKP--------NGKVNLKGLAIGNGLTD
 At1g11080 KLGDDFTARDAYTFLCNWFEK-FPEHKENTFYIAGESYAGKYVPELAEVVYDNNNNNK-KNGSSFHINLKGILLGNPETS
 At4g15100 NLDDHFAKKDAYTFLCNWFEK-FPEHKGNEFYIAGESYAGIYVPELAELVYDNNEKN---NDLSLHINLKGFLLGNPDIS
 At1g61130 KLGDDFTARDSYTFLQKWFLR-FPAYKEKDFFIAGESYAGKYVPELAEVIYDKNKDN---ENLSLHINLKGILLGNPLTS
Os01g22980 SIDDRFVAEDTYNFLVNWFKR-FPQYKNHDFYISGESYAGHYVPQLADVVYERNKHV----ETNQHINLKGFIVGNAETD
Os05g06660 NLNDDFVAEDAYSFLVNWFKR-FPQYKDNEFYISGESYAGHYVPQLADLVYERNKDK----RASTYINLKGFIVGNPLTD
 At3g17180 NLNDAFVAEDAYNFMVAWFAR-YPQYKSRDFFIAGESYAGHYSPQLAELIYDRNKVQ----PKDSFINLKGFIVGNPLTD
 At5g08260 KLGDEVTASDSLAFLINWFMK-FPEFRSSEFYISGESYAGHYVPQLAEVIYDRNKKV----TKDSSINLKGFMIGNAVIN
Os04g44410 RLGDRVTAQDSYSFLLNWLNK-FPEFKNRDFYIAGESYAGHYVPQLAELIYDGNKGA----SRDRVINIKGFMIGNAVLN
 At5g23210 QLGDTVTARDSYNFLVNWFKR-FPQYKSHDFYIAGESYAGHYVPQLSELIYKENKIA----SKKDFINLKGLMIGNALLD
Os02g42310 QLGDKITADDAYIFLLNWFKR-FPQYKSHDFYIAGESYAGHYVPQLSEKIFDGNKQG----PKENYINFKGFMIGNALMD
Os10g39560 PPGDNSTAYGSYTFLIRWFQR-FPQHKMKEFYIAGESYAGHYVPQLANVIVDQNKIA----PKENYINLKGIMIGNAYMD
Os06g51370 HFGDNLTAHDAHAFLVNWLER-FPQFKGHDLYIAGESYAGHYVPQLATKILHFNKKKKEHDDDDRIINLKGIMIGNAAID
 At2g24000 ESGDERTAQENLIFLISWMSR-FPQYRYRDFYIVGESYAGHYVPQLAQKIHEYNNAYK----N-PVINLKGFMVGNPEMD
 At2g24010 DSGDERTAQENLIFLIKWMSR-FPQYQYRDFYIVGESYAGHYVPQLAKKIHLYNKAFN----NTPIINLKGFMVGNGDMD
 At4g30610 DSGDERTAQDNLIFLIKWLSR-FPQYKYRDFYIAGESYAGHYVPQLAKKINDYNKAFS----K-PIINLKGFLVGNAVTD
Os02g55130 TSGDERTAQDALQFLISWMSR-FPQYRHRDFYIAGESYAGHYVPQLARKIVEFNKASP----Y-PFINLKGILVGNGVTD
 At3g02110 NTGDRRTAKDSLQFLIQWLHR-FPRYNHREIYITGESYAGHYVPQLAKEIMNYNKRSK----N--PLNLKGIMVGNAVTD
 At3g07990 TTGDQRTAEDSYIFLVNWFER-FPQYKHREFYIVGESYAGHFVPQLSKLVHERNK-GF----KNPAINLKGFMVGNAVTD
Os01g61690 VAGDAKTASDAYAFLVNWLER-FPQYKYREFYIAGESYAGHYVPQLAQLIYEQNK-GI----QNPIINLKGFMVGNAVTD
 At2g35780 TAGDQRTAEDAYVFLVKWFER-FPQYKHREFYIAGESYAGHYVPQLSQIVYEK---------RNPAINFKGFIVGNAVID
Os01g06490 TAGDNKTAHDSYAFLVNWLER-FPQYKYRDFYIAGESYGGHYVPQLSQLVYRNNK-DV----EKPILNFKGFMVGNAVID
 At4g30810 SNGDKRTAEDSLKFLLKWVER-FPEYKGRDFYIVGESYAGHYIPQLSEAIVKHNQ-GS----DKNSINLKGYMVGNGLMD
Os06g08720 NNGDARTANDSLTFLTKWIER-FPQYKGREFYVTGESYAGHYVPQLAQAIKRHHE-AT----GDKSINLKGYMAGNALFD
 At2g35770 TVGDKRTGEDAYRFLVRWLER-FPEYKERAFYIAGESYAGHYIPELAQLIVNRNK-GA----KNPTINLKGILMGNPLVD
Os09g28830 KSGDQRTANDSYIFLVNWLER-FPEYKGRAFYISGESYAGHYAPQLAATILTHNMESK-----RMIINLQGILVGNPCLD
Os09g28840 DVGDQITAEDTYVFLLNWFNR-FPEYKGRDFYIAGESYGGHYVPQIATIVTFINHLFDG----NTPFNLRGIFVGNPLLD
Os07g46350 RMGDNSTAEDAYKFLVNWMER-FPEYKGRDFYLAGESYAGHYVPQLAHAILRHSSAAAGGKPSSSPINLKGIMIGNAVIN
Os03g26920 NTGDTSTAADAYTFLTNWLER-FPEYKGRDFFITGESYGGHYIPQLANAILSNNNITN-----VTIINLKGVAIGNAYLD
Os03g26930 NIGDKKTTDDAYIFLINWMKK-FPEYQGHDFFITGESYAGHYIPELANLIVSNNRAIN-----STNIKLKGVAIGNADLH
Os03g09190 TIGDNMTAHDSHTFLLRWLDR-FPEYKTRDLFIVGESYAGHYVPELAVTILDNNLLPH-----ATPIKLKGIAIGNGILE
 At3g52000 KQGDKATAEDNYMFLVNWLER-FPEYKGRDIYIAGQSYAGHYVPQLAQIILHRN-NQTLIN-------LRGILIGNPSLN
 At3g52010 EKGDKATAEDNYIFLMNWLER-FPEYKGRDIYIAGQSYAGHYVPQLAQIIIHRN-KKTLVN-------LRGILIGNPSLL
 At2g05850 EQADKLTAEDNYMFLVNWLER-FPEYKGRDVYISGQSYAGHYIPQLAQIILHRN-NQTFIN-------LRGISIGNPGLD
 At3g52020 NPGDMKAAADKYIFLVKWLER-FPEYKGREFYIAGESYAGHYVPQLAQTILVHNKNQNFIN-------LRGILIGNPTLN
 At3g63470 -HGDRNTAADNYIFLVNWLER-FPEYKGRDLYIAGESYAGHYVPQLAHTILLHH--RSFFN-------LKGILIGNAVIN
 At1g28110 GVNDKITARDNLVFLQRWFLK-FPHYLNRSLFITGESYAGHYVPQLAELMIQYNK-------KHHLFNLRGIAIGNPVLE
 At2g33530 GVNDKITAKDNLVFLQKWFLK-FPQYLNRSLFITGESYAGHYVPQLAQLMIQYNK-------KHNLFNLKGIAIGNPVME
Os04g32540 GVGDSRTARDNLRFLQGWFAK-FPQYKGRDLYITGESYAGHYVPQLAQRMVEFNK-------KEKLFNLKGIALGNPVLE
Os11g31980 GVDDKMTAMDNMVFLQRWLEK-FPQYKGRELYISGESYAGHYIPQLADVMVEFNK-------KNKIFNLKGIALGNPVLE
 At2g12480 -TGDKSTVNDMLVFLLRWFNK-FPELKSRDLFLTGESYAGHYIPQLADVILSYNS-RS----SGFKFNVKGIAIGNPLLK
 At5g42240 -TGDKSTANDMLVFLLRWFEK-FPKLKSRDLFLTGESYAGHYIPQLADAILSYNS-HS----SGFKFNIKGVAIGNPLLK
 At5g42230 -AGDKSAASDMLVFLLRWFDK-FPELKSHDLFLTGESYAGHYIPQLADAILSYNS-RS----SGFKFNIKGIAIGNPLLK
 At1g43780 -TGDESTAKDMLVFMLRWLEK-FPQFKTRNLFLAGESYAGHYVPQLADVILEYNAQRS----NRFKFNLKGIAIGNPLLK
Os11g10750 -TGDAQTANDMYKFLLGWYKK-FPEYRSRGLLLSGESYAGHYIPQLTDVLLTHNE-KS----NGFKFNIKGVAIGNPLLK
 At1g73300 KPSDSGEAKRIHEFLQKWLGK-HQEFSSNPFYVAGDSYSGLVVPATVQEISKGNYECC-----NPPINLQGYVLGNPLTD
 At5g36180 KPSDSGEAKRIHEFLQKWLGK-HQVFSSNPFYVAGDSYSGLVVPATVQEISKGNYECC-----NPPINLQGYVLGNPLTD
 At1g73280 KPSDSGEAKRIHEFLQKWLGK-HQEFSSNPFYVGGDSYSGMVVPATVQEISKGNYECC-----NPPINLQGYVLGNPLTD
 At1g73310 KPSDTGEAKRIHEFLQKWLGK-HQEFSSNPFYVGGDSYSGLVVPATVQEISKGNCQCC-----NRPINLQGYVLGNPLTD
 At1g73290 KPSDSGEAKRIHEFLQKWLSK-HQEFSSNPFYVAGDSYSGMVVPATVQEISKGNYQCC-----SPPINLQGYVLGNPITE
 At1g73270 KPSDTGEAKRIHEFLQKWLGK-HQEFSSNPFYVGGDSYSGITVPATVQEISKGNYQCC-----KPPINLQGYMLGNPITD
 At3g10450 KPSDSGEAKRIHEFLHKWLGK-HQEFSSNPFYVGGDSYCGMVIPALVQEISKGNYVCC-----KPPINLQGYILGNPSTE
 At2g22920 TPSDTGEVKNIHEFLQKWLSK-HPQFSSNPFYASGDSYSGMIVPALVQEISKGNYICC-----KPPINLQGYILGNPITY
 At2g22970 TPTDTGEVKRIHEFLQKWLSK-HPQFSSNHFYAGGDSYSGMIVPALVQEISKGNYICC-----NPPINLKGYVLGNPITH
 At2g23000 S-SDTSEVKRIHEFLQKWLSK-HPQFFSNPFYVTGDSYSGMIVPALVQEISKGNYICC-----KHLINLQGYVLGNPITY
 At2g23010 T-SDTSEVKKIHEFLQKWLIK-HPQFLSNPFYVVGDSYSGMIVPALVHEISKGNYICC-----NPPINLQGYVLGNPITH
 At2g22990 T-GDISEVKRTHEFLQKWLSR-HPQYFSNPLYVVGDSYSGMIVPALVQEISQGNYICC-----EPPINLQGYMLGNPVTY
 At2g22980 KISDTGEVKRIYEFLQKWLSK-HQQFFSNPFYVGGDSYSGMIVPPLVQEIGKG----------NYQINLQGYILGNPITD
 At3g12230 RPSDTGSAKLVNEFVRKWLAK-HPDYFSNPFYVTGNSYSGKVIPAIVQEISNGNYICC-----KPQINLQGYVIGNPVAY
 At3g12240 IPSDTGSVKRVNEFVRKWLAK-HPEYFSNPFYVTGNSYSGKVIPAIVQEISNGNYICC-----KPQINLQGYVIGNPVAY
 At3g12220 IPSDTGSAKRVDEFLRKWLTK-HPEYFSNPFYAGGNSYSGKMVPVIVQEISNGNCIYG-----KPQIRLQGYVLGSPVTD
 At3g12203 IPSDTGVAKPVNEFLHKWLDK-HPEFLSNPLYVAGNSYSGIVIPTIVQEISNGNHLDS-----KPQINLQGFVLGNPATD
 At1g33540 KPSDTGEAKQTYEFLQKWLVE-NPQFVSNPIYVGGDSYAGIVVPAIVQQISIGNEHGY-----KPQINLKGYILGNPSTD
 At5g09640 ESSDTKQMHQIDQFLRSWFVK-HPEFISNPFYVGGDSYSGKIVPGAVQQISLGNEKGL-----TPLINIQGYVLGNPVTD
Os10g01110 RTGDTIAVHHILVFLNNWFQEVHPDFLSNPLYIAGDSYSGMIVPAVTFGIAT---SSP-----KPSLNLKGYLLGNPVTD
Os11g42390 RTGDTIAVRHILVFLRKWLQEVHPVAKPGFG-----------------------------------YRGCWPGDGYLLGNPVTD
Os04g09720 KSSDTIVVHQLYTFIQKWFDD-HPQFSSNPLYVSGDSYSGIIIPTLTMEIAKGKESSD-----ERHLNLKGYIAGNPLTD
Os02g46260 DVGDISSSMQIVKFLRKWLDD-HPKYLSNPFYIGGDSYAGKVVPLITQYLSEGIEEMR-----HPIINLKGYLVGNPSAG
Os03g52080 NVGDISSSLQVVTFLKKWFND-HPSYLSNHFYVGGSSYAG---------------------------KGYMVGSPLTD
Os11g24510 DVGDVSSTLQLTKFVNKWFSQ-HREFLSNPLYVGGESYAGKLVPFLLQKISEDVEAGV-----KPVLNLKGYLVGNPGTG
Os12g39170 LVGDVSASLQLKHFLAKWISD-HPEYLANPFYIGGDSYAGKIVPFLAQKISEDIEAGV-----RPIVDLKGYLVGNPVTG
Os10g01130 NVTLTQTGQQLVVFLTKWLGD-HPEFASNPLYIGGDSYSGYTVPVTALQIANDDDARA-----R--LNLKGYLVGNAATD
Os11g27200 HRISEPTHPPQLTKPELWLHD-HPQFLLSPLYIGGDSYSGIIVPTLALAIDESNDSGD-----KPILNLMGYVAGNPVTD
Os02g26480 VTNDTSRVLDLYDFLSKWFSE-YLEFLSNPFYIAGCSYSGVIVPVLAQEILKRNEDNG-----RIKINFKGYSLCNPAVD
 At3g25420 TTDDTKTASDTHTFLLEWFKM-FPEFQSNPFFISGESYAGIYVPTLAAEVVKGHKNVT-----KPVINFKGYLVGNGVTD
 At4g12910 ITGDIKTAVDSHAFLLKWFQM-FPEFQSNPFFISGESYAGVYVPTLASEVVIGNKNGV-----KPALNFKGYLVGNGVAD
Os04g25560 TTGDLKTAADAHTFLLKWFEL-YPEFQSNPFYMSGESFAGIYIPTLADEVVKGIEKDL-----KPRINFKGYLIGNGATD
Os12g15470 NTGDLKTAADSHTFLLKWFQL-YPEFLSNPFYIAGESYAGVYVPTLSHEVVKGLHDGV-----KPTINFKGYMVGNGVCD
Os02g02320 RHDETGVSNDLYSFLQVFFKK-HPEFAKNDFFITGESYAGHYIPAFASRVHQGNKANE-----GIHINLKGFAIGNGLTD
Os07g29620 RHDEAGVSNDLYAFLQAFFTE-HPNFAKNDFYITGESYAGHYIPAFASRVYKGNKNSE-----GIHINLKGFAIGNGLTD
 At3g10410 RHDETGVSNDLYDFLQAFFAE-HPKLAKNDFYITGESYAGHYIPAFASRVHKGNKANE-----GVHINLKGFAIGNGLTD
 At3g45010 RHDEDGVSNDLYDFLQAFFKE-HPQFVKNDFYITGESYAGHYIPALASRVHRGNKNKE-----GTHINLKGFAIGNGLTN
 At5g22980 RNDEDGVSNDLYDFLQAFFKE-HPKFVKNDFFITGESYAGHYIPALASRVHSGNKKKE-----GIPINLKGFAIGNGLTN
Os03g27480 VTTDGEAAADLAALLRALAAKEIPTLQSSPLFLVAESYGGKYAATLGVSLARAIRAGG------LKLTLAGVALGDSWIS
Os08g44640 ARTDAEAATDLTTLLSQLYRSNNTRLQGSPLYIVAESYGGKFAVTTALAALKAIHAGR------LAASLAGVALGNSWIS
 At2g27920 VKSDEEAAQDLTKLLQQLFNKN-QTLNQSPLFIVAESYGGKIAVKLGLSVIDAVQSGK------LKLHLGGVILGDSWIS
Os03g27510 ---DVQAAADATELVKALATRILPDLKSSPLFIVGQAYGGKLAAMIGASLAKPIRAGD------IDLTLGGVVIGNGWIS
Os03g27530 ---QGRRRAHIKALP-----DEIPALKCSPLYHVGESYGGKLAAMIGVSLTKSIHAGD------LDLTLGGVVIRDGWIS
Os01g22970 SHNSNQAFMSINRSLSN-----------------------------------------------WILDSGVSRHVTAALALL
```

```
Os01g11670 PVAQV-----------------------ATHADTAYFMGLINAKQRRELEALQARAVELTNAARWSEAADARELVLSLLE
Os05g50600 PVAQV-----------------------ATHADTAYFMGLINAKQKRELEALQARAVELTNAARWSEAADARGLVLSWLE
Os06g36570 -RGGW-----------------------PTRGS--------------------S----CCRCWRTRRADTRELVLSLLE
Os05g50570 PVTQV-----------------------TTHADIAYFMGLINGKQKREVEAMQARAVELIKAERWSEAYVAREGLLKWME
Os05g50580 PVAEV-----------------------TTHADIAYFMGLINAKQKRAAEAMQAEAVALTREERWREASAARARLMSWLE
Os01g43890 PVAQV-----------------------TVHADQAYFAGLINAEQKAKVEEMQDKTVSLIKSKKWAAARRERNRIIAFLK
 At1g15000 PVTQV-----------------------QTHAVNVYYSGLVNAKQRVELQKAQEISVALVKSQKWREAADARTELLTLLS
 At1g11080 DAEDW-----------------------RGWVDYAWSHAVISDETHRIITRTCN---FSSDNTWSNDECNEAVAEVLKQY
 At4g15100 NPDDW-----------------------RGWVDYAWSHAVISDETHRNINRLCN---FSSDDVWNNDKCNEAIAEVDKQY
 At1g61130 YAEDW-----------------------TGWVDYAWNHAVVSDETYRVIKQSCN---FSSDTTWDVKDCKEGVDEILKQY
Os01g22980 DYYDY-----------------------KGLVEFAWSHSVISDQLYKHVNNVCD---FRLSPRS--NECNHVMGYIYDQY
Os05g06660 DYYDS-----------------------KGLAEYAWSHAIVSDQVYERIKKTCN---FKNSNWT--DDCNAAMNIIFSQY
 At3g17180 DEYDN-----------------------KGILEYAWSHAVISDHLYDSAKHNCD---FKSSNWS--EPCNVAMNTVFTKY
 At5g08260 EATDM-----------------------AGLVDYAWSHAIISDEVHTSIHGSCS---FEEDTTNKTEQCYNNFKGFMDAY
Os04g44410 DATDQ-----------------------MGMVEYAWSHAIISDELYSAVRRECDSFKEEEDGGKPSKGCSPAVRAFLRAY
 At5g23210 DETDQ-----------------------KGMIEYAWDHAVISDALYEKVNKNCD-----FKQKLVTKECNDALDEYFDVY
Os02g42310 DETDQ-----------------------TGMIDYAWDHAVISDRVYADVKKYCN-----FSMENVTDACDSALTEYFAVY
Os10g39560 GDTDL-----------------------LGIVDSAWHHALISDKLYSDFQKFCN-----FSLVDLSKECNAAIDQFNALY
Os06g51370 SSSDD-----------------------RGLVEYAWDHAVISDEIYAAIKGNCTFP----DDGNETDKCNTAWNGFFTAM
 At2g24000 KNNDR-----------------------LGTITYWWSHAMISDASYNRILKNCDFT----ADRFSKECD-SAIYVAAADF
 At2g24010 KHYDR-----------------------LGAAMYAWSHAMISDKTYKSILKHCSFT----ADKTSDKCN-WALYFAYREF
 At4g30610 NQYDS-----------------------IGTVTYWWTHAIISDKSYKSILKYCNFT----VERVSDDCDNAVNYAMNHEF
Os02g55130 NYYDN-----------------------IGTVTYWWTHAMISDTTYKAIMSSCNFT----SANVSRLCNRAMSYAMNHEF
 At3g02110 NHYDN-----------------------LGTVSYWWSHAMISDRTYHQLISTCDFS----RQKESDECETLYSYAMEQEF
 At3g07990 DYHDY-----------------------IGTFEYWWNHGLISDSTYHQLKTACYSV----SSQHPSMQCMVALRNAELEQ
Os01g61690 DYHDY-----------------------LGTFEYWWTHGLISDNTYHNLKKTCLLE----SSEHPSPECLKNLNLASSEE
 At2g35780 DYHDY-----------------------VGLFEYWWAHGLISDLTYHNLRITCEFG----SSEHPSSKCTKAMEAADLEQ
Os01g06490 DYHDY-----------------------VGTFEYWWTHGLISDDTYQKLQVACDFE----SSAHASEACNKIYEVAEAEQ
 At4g30810 DFHDR-----------------------LGLFQYIWSLGFISDQTYSLLQLQCGFE----SFIHSSKQCNKILEIADKEI
Os06g08720 DFHDH-----------------------LGIFQFMWTNGLISDQTYRLLNVFCDYE----SFVHTSSQCNKILDIASDEA
 At2g35770 DYNDN-----------------------KGMRDYWWNHGLISDESYNDLTKWCLND----SILFPKLNCNAALNQALSEF
Os09g28830 EFKNL-----------------------KGQIDYLWSHGVISDEVLANITKNCRFSP--SDGK------ACSDAMDAFDS
Os09g28840 EYKNG-----------------------EGNLEFLWSHGVISDEVWGKILANCTFTS--SDDW------PCFVAAHSFQR
Os07g46350 DWTDT-----------------------KGMYDFFWTHALISDEANDGITKHCNFTD--GADA--NSLCDDATSLADDCL
Os03g26920 DSTNT-----------------------RATIDYYWTHALISKETHLAVQRNCSFN---GTY---MAQCRNALAEADTEK
Os03g26930 DNVTL-----------------------RASFDYYWRHAMISDRVYRAIQTSCGFN---ETY---TNDCQNAMNLANKEK
Os03g09190 FAAEQ-----------------------TQLYEYLWQHAFISDSAHALITQSCKYP---DDHP--SALCESARKAAYSRI
 At3g52000 REIQD-----------------------DFGYKFMFSHGLISQQQMDNYNKFCTDSD--LYD---WDKCHLASQKIEAQK
 At3g52010 TSIQD-----------------------PYGYEFMLSHGLMSQQQMDNYNQFCLRDD--LYD---NDKCALSVKTIDDAK
 At2g05850 LLIEA-----------------------DNENKFILSHGLVSQKDFEEYSKVCDFAN--YDM---DECPKIMPKFSIEHN
 At3g52020 DIVET-----------------------TGSFDYLVSHALLSQDSLLSYKENCATDT--PKM---EVDCIALSMKIDDDI
 At3g63470 DETDL-----------------------MGMYDFFESHALISEDSLARLKSNCDLKT--ESASVMTEECAVVSDQIDMDT
 At1g28110 FATDF-----------------------NSRAEYFWSHGLISDSTYKMFTSYCNYSRYVSEYYRGSMSSMCSKVMSQVST
 At2g33530 FATDF-----------------------NSRAEYFWSHGLISDPTYKLFTSSCNYSRFLSEYHRGSVSSMCTKVLSQVGI
Os04g32540 FATDF-----------------------NSRAEFFWSHGLISDSTYHSFTTVCNYSRYVSEYYHGSLSSACDTVMTQVAR
Os11g31980 FTTDF-----------------------NSRAEYFWSHGLISDSTYRIFTSVCNYSRYVTEYYGGSLTPLCARVMNQVTR
 At2g12480 LDRDF-----------------------AAAYEYFWSHGMISDEVRLTIMNQCDFAN-----PKNMSN-ACIYAIVESS-
 At5g42240 LDRDS-----------------------PATYEFFWSHGMISDELKLTITSQCDFDDYTFASPHNVST-ACNEAISETEN
 At5g42230 LDRDI-----------------------PAVYEFFWSHGMISEVVGRTIKIQCDFSHYTYAYPHNVSD-ACNDAIREAGD
 At1g43780 LDRDV-----------------------PAIYEFFWSHGMISDELGLTIMNQCDFEDYTFTDSHNISK-LCEAAVNQAGT
Os11g10750 LDRDV-----------------------PATYEYFWSHGMISDEIFLSISHSCDFEDYTFSNPHNESK-SCNDAIAEANS
 At1g73300 YAIDS-----------------------NSRIPFAHGMALISDELYESLKKTCKGEYTNVHPR--NTQCLKFIEEFNKCT
 At5g36180 YTTGS-----------------------NSRIPFAHGMALISDELYESLKKTCKGEYTNVHPR--NTQCLKFVEEFNKCT
 At1g73280 FVYDY-----------------------NSRIPFAHGMALISDELFESLKKTCKGDYRNVHPR--NTECLKFIEEFNKCT
 At1g73310 CVYDC-----------------------NYRVPFAHKMALISDELYESLKRTCRGEYVNVHPH--DTECLKFVEEFNKLT
 At1g73290 HAIDY-----------------------NYRIPFAHGMALISDELYESLKRVCKGEYVDPR----DTECLKLVEEFSKCT
 At1g73270 SKIDG-----------------------NSQIPYAHGMALISDELYESLKRICKGEYEHVDPY--NTECLKLLEEFNECT
 At3g10450 NEVDI-----------------------NYRIPYAHGMALISDELYESMKRICKGKYENVDPR--NTKCLKLVGEYQKCT
 At2g22920 FEVDQ-----------------------NYRIPFSHGMALISDELYESIRRDCKGNYFNVDPR--NTKCLKLVEEYHKCT
 At2g22970 EDDPN-----------------------YRIPFSHGMALISDELYESIREACKGNYFNVDPR--NTKCLKLVEEFHKCT
 At2g23000 AEHEK-----------------------NYRIPFSHGMSLISDELYESLKRNCKGNYENVDPR--NTKCVRLVEEYHKCT
 At2g23010 IEFEQ-----------------------NFRIPYAHGMSLISDELYESLKRICKGNYFSVDPS--NKKCLKLVEEYHKCT
 At2g22990 MDFEQ-----------------------NFRIPYAYGMGLISDEIYEPMKRICNGNYYNVDPS--NTQCLKLTEEYHKCT
 At2g22980 TESEQ-----------------------NYQIPYAHGMALISDELYKSMERICKGNYVKVDSL--NTKCYKLIKDYQKCI
 At3g12230 YDHDK-----------------------DSRIPFAHGVALISDELFESLKRSCGGSYSIVDPL--NTECLKLIKDYHKCV
 At3g12240 YDHDK-----------------------DFRIPFAHGVALISDELFESLKASCGGSYSVVDPL--NTECLKLIEDYDKCV
 At3g12220 YDLDR-----------------------NSRIQFAHGMALISNELYESMKRTCGGNYIFVDPL--NTECLELIKDYDNCV
 At3g12203 TDIDL-----------------------NSRIPFAHGKALISDEHYESLKRSCQGNYISVNPR--NTKCLKLLEDFKKCV
 At1g33540 LDSDH-----------------------NSKIPYAHRMGLISDELYESLKRTCQGNYVKVDPT--NTKCLKLMEDYGKCV
 At5g09640 KNIET-----------------------NYRVPFAHGMGLISDELFESLERSCGGKFFNVDPS--NARCSNNLQAYDHCM
Os10g01110 HNFDA-----------------------PSKIPFAHGMGLISDQLYQAYKKSCS-VKHNTQQQ--SVQCTNTLDAIDECV
Os11g42390 PNFDT-----------------------PSKIPFTHGMGLISDELY-------------------------------ECV
Os04g09720 TTHDD-----------------------NSKFPFLHSLGIIDDELYEVARKNCK-GDYMTPPN--S-QCANSVQAIRDCI
Os02g46260 DKIDV-----------------------NSRVPYSHSFGVISDQLYEAALANCKG--DYENPT--NKPCTDVMQTINNLM
Os03g52080 PKYDR-----------------------NSIIPYAHGVGIISDQLYEAAVANCKG--DYVNPT--NEICANVLNAVDNLM
Os11g24510 ESIDY-----------------------ESKVPYAHGVGIISDQLYETILEHCGRE-DYANPK--NATCAQALNRFSELM
Os12g39170 ESIDF-----------------------DSRVPYAHGVGIISDQLYETIMEHCQGE-DYSNPK--NAICRQALDRFNELL
Os10g01130 VKYDS-----------------------GGKVPFMHGMGLISDEMYEAARSSCRGDYVS-TPT--NADCANALQAISMAT
Os11g27200 SQFDE-----------------------DGKIPCLHGMGLISNELYEHAKETCRGKYS--APG--NARCEQSIQAIRNCT
Os02g26480 VEIEN-----------------------NAFVPYAFRMGLISDELYQNLVSTCNGKYWN--NK--GPSCLANLEQFHKQI
 At3g25420 EVFDG-----------------------NALVPFTHGMGLISDELYEETKLVCNGTYYTGGQSGVSKECAGKLKTVSDTV
 At4g12910 PKFDG-----------------------NAFVPFAHGMGLISDELFENVTKACKGNFYEIEG----LECEEQYTKVNDDT
Os04g25560 QDYDF-----------------------NSFVPFAHGMGLISTELFEDASTACHGTFWGKVN----NLCQEKIDRVHWEL
Os12g15470 TVFDG-----------------------NALVPFAHGMALISDDIYQEAQTACHGNYWNTTT----DKCENALYKVDTSI
Os02g02320 PAIQY-----------------------KAYTDYALDMNLIKKSDYDRINKFIPPCEFAIKLCGTNGK--ASCMAAYMVC
Os07g29620 PAIQY-----------------------KAYTDYSLDMGLITKSQFNRINKIVPTCELAIKLCGTSGT--ISCLGAYVVC
 At3g10410 PALQY-----------------------PAYPDYALEMGLITQKEHDRLEKIVPLCELSIKLCGTDGT--TSCLASYLVC
 At3g45010 PEIQY-----------------------GAYADYALDMNLITQSDHDNLNRYYATCQQSIKECSADGGEGDACASSYTVC
 At5g22980 PEIQY-----------------------GAYGDYALQMKLISESDHESLKQDYVECQNITKKCSLGGG--LVCDSAVEVC
Os03g27480 PEDFA-----------------------LSYGPLLRQVSRLDSNGADSASKKAQVVKQRIASGQWKLAQYALSSMLTSIV
Os08g44640 PEDSV-----------------------LSWGPLLYQVSRLDENGLYLSDSLAQQIKAQVKAAQFLEAENTWQSLESIIL
 At2g27920 PEDFV-----------------------FSWGPLLKHVSRLDDNGLDSSNSLAEKIKTQIKNGEYVGATQTWMDLENLIS
Os03g27510 PADFSTFTGRDTAISYFKFSLVIRVPIRLTYARMLSDVSWLDGNAIDDVNKMAGKVKEQTAAGQFATSLQTFTDLLYLID
Os03g27530 PTDFS-----------------------LTYARLLDDVSRLDENAIDDANKLAEKVSEQSAAGQFAASLQTFTGLIDLID
```

```
Os01g11670 NATGLATQFDAAKQR-----------------------------------------------------------------
Os05g50600 NATGLATLFDAAKKR-----------------------------------------------------------------
Os06g36570 NATGLATLFDAAKQR-----------------------------------------------------------------
Os05g50570 NASGVPSLFDVEVTTSL---------------------------------------------------------------
Os05g50580 NATGVVTLLDVEVQQSV---------------------------------------------------------------
Os01g43890 NATGVATPFNYAREK-----------------------------------------------------------------
 At1g15000 NMTGLATLYNTARAIP----------------------------------------------------------------
 At1g11080 HEIDIYSIYTSVCIGDSARSSYFDS-------------------------------AQFKTNSRISSKRMP-PRLMGGYD
 At4g15100 NEIDIYSLYTSACKGDSAKSSYFAS-------------------------------AQFKTNYHISSKRMP-PRRLAGYD
 At1g61130 KEIDQFSLYTPICMHHSS-----------------------------------------KVDSYANYKTTI-PRLFDGFD
Os01g22980 DMIDIFNVYAPKCNTDDSSLFSTS----------------------------------YSTADMNAKKRLKGTRMYSGYD
Os05g06660 NQIDIYNIYAPKCLLNSTSASSPD----------------------------------RAFFANNQEQFRWRIKMFSGYD
 At3g17180 KEIDIYNIYAPKCISNSSSGASYLG-------------------------------FGVNDKSPAVKDWFKRVRWFEGYD
 At5g08260 NDIDIYSIYTPVCLSSLLSS--------SPRK--PKI-------------------VVSPRLLTFDDLWD---KFPAGYD
Os04g44410 DDIDIYSIYTPTCLSSSSSSPASA----SPRRSSPGL-------------------VAAPRLFSKHEAWRRMQRVPAGYD
 At5g23210 KILDMYSLYAPKCVPTSTNSSTSHSVAGNRPLPAFRS-------------------ILRPRLISHNEGWR---RMAAGYD
Os02g42310 RLIDMYSLYTPVCTEVSSSAAFGQ-------RQVAVH-------------------GAAPKIFSKYHGWY---MRPAGYD
Os10g39560 SIIDIYSLYTPRCELG----------------------------------------YPNFNSSFAAQIGRTSSRIPMGYD
Os06g51370 GDIDIYSLYTPSCTAALNGTTTITN--------------------------------GTRSRFADKVLRLRRGLPYNTYN
 At2g24000 GDIDQYSIYTPKCVPPQDQTNQTK-------------------------------------FEQMMQMHTTKRFLEDQYD
 At2g24010 GKVNGYSIYSPSCVHQTNQT---------------------------------------------KFLHGRLLVEEYEYD
 At4g30610 GDIDQYSIYTPTCVAAQQKKNTTG-------------------------------------FFVRMKNTLLRRRLVSGYD
Os02g55130 GDIDQYSIYTPSCAAAAAANATGRRR-GK-------------------------------AAVLRFKDTFLRRRSFG-YD
 At3g02110 GNIDQYNIYAPPCNKSSDGGGSYNGSSGR-------------------------------RSMRLPHLPHSVLRKISGYD
 At3g07990 GNIDPYSIFTKPCNSTVALKRFLKG---------------------------------------------RYPWMSRAYD
Os01g61690 GNIDPYSLYTKPCNNTASLKLGLGG---------------------------------------------RYPWLSRAYD
 At2g35780 GNIDPYSIYTVTCKKEAAALRSRFSRV-------------------------------------------RHPWMWRAYD
Os01g06490 GNIDAYSIYTPTCKKTSFLKRRLIRG--------------------------------------------NSPWLPRGYD
 At4g30810 GNIDQYSVFTPACVANASQS-NMLLKKR-----------------------------------------PMTSRVSEQYD
Os06g08720 GNIDSYSIFTPTCHASFASSRNKVMKRL-----------------------------------------HSVGKMGERYD
 At2g35770 GDIDPYNINSPACTTHASSNEWMQAWR------------------------------------------------YRGND
Os09g28830 GNTDPYDIYGPVCINAPDGK------------------------------------------------FFPSR-IVPGYD
Os09g28840 VNIDRYNIYAPVCLHEQDGT------------------------------------------------FRSSG-YLPGYD
Os07g46350 QDIDIYNIYAPNCQSPGLVV------------------------------------------------SPPVTPSIESFD
Os03g26920 GVIDPYNIYAPLCWNASNPR------------------------------------------------QLHG--SAINVD
Os03g26930 GNVDDYNIYAPQCHDASNPS------------------------------------------------PSGSSDSVAFGD
Os03g09190 GNIDIYNIYSSTCHEQKVR--------------------------------------------------PSASKCMDLAD
 At3g52000 THLDIYNIYAPLCLNSTLS------------------------------------------------SEPKKCTTIMKAD
 At3g52010 KHLDTYNIYAPVCLNSTLS------------------------------------------------RISKKCTTVLEVD
 At2g05850 KHLDVYNIYAPVCLNSTLS------------------------------------------------SEPKKCTTIMEVD
 At3g52020 KKMNLYNILTPTCINATLTPL---------------------------------------------TNQSKECTTVLQYE
 At3g63470 YYLDIYNIYAPLCLNSTLT------------------------------------------------RRPKRGTTIREFD
 At1g28110 ETSRFVDKYDVTLDVCIPSVLSQSKV-----------------------------------------VSPN--QVGESVD
 At2g33530 ETSRFIDKYDVTLDVCIPSVLSQSKV-----------------------------------------VSPQPQQVGETVD
Os04g32540 ETSRFVDKYDVTLDVCVSSVLMQSKS-----------------------------------------LAPQ-RGS-RELD
Os11g31980 ETSRFVDKYDVTLDVCLSSVLSQSKI-----------------------------------------LTPH-QQVGQRID
 At2g12480 VLTEYINSYHILLDVCYPSIVQQELR-----------------------------------------LKKMN--------
 At5g42240 IITEYVNNYDVLLDVCYPSIVQQELR-----------------------------------------LKKMATKMSMGVD
 At5g42230 ITTEYVNTFDVLPDLCYPSIALQELR-----------------------------------------LKQMATKMSMGVD
 At1g43780 IITQYVNYYDILLDVCYPSLFEQELR-----------------------------------------LKKMGTRMSFGVD
Os11g10750 IVGDYVNNYDVILDVCYPSIVMQELR-----------------------------------------LRKYVTKMSVGVD
 At1g73300 NRILQQLILDPLCETET-----------------------------------------------------------PDCY
 At5g36180 NRIFQQLILDPLCETET-----------------------------------------------------------PDCY
 At1g73280 NSICQRRIIDPFCETET-----------------------------------------------------------PNCY
 At1g73310 NRVCERHILHSCCETET-----------------------------------------------------------PSCY
 At1g73290 KGVCQEVVIKPLCVTET-----------------------------------------------------------PNCY
 At1g73270 SKLYRSHILYPLCEMTN-----------------------------------------------------------PDCY
 At3g10450 KRINKALIITPECVDTS-----------------------------------------------------------PDCY
 At2g22920 DELNEFNILSPDCDTTS-----------------------------------------------------------PDCF
 At2g22970 DKLNEFHILSPDCDTAS-----------------------------------------------------------PDCY
 At2g23000 DKINTQHILIPDCDKKGHGITS------------------------------------------------------PDCY
 At2g23010 DNINSHHTLIANCDDSNTQHIS------------------------------------------------------PDCY
 At2g22990 AKINIHHILTPDCDVTN--VTS------------------------------------------------------PDCY
 At2g22980 HKLNKYHILLPDCDITS-----------------------------------------------------------PDCF
 At3g12230 SGIYQELILKPKCETTSP-----------------------------------------------------------DCY
 At3g12240 SGIYEELILKSKCEHTSP-----------------------------------------------------------DCY
 At3g12220 SGIYENLILVPKCDLTSP-----------------------------------------------------------DCH
 At3g12203 SGISEEYILKPDCMWLY------------------------------------------------------------SCM
 At1g33540 SRINEGLILIALCDLASPNPYSG---EHGGRSYL------------------------------QTLVQSDLSLPTPDCY
 At5g09640 SEIYSEHILLRNCKVDYVLADTPNIRTDRRRVMK------------------------------EFSVNDSSSLPPPSCF
Os10g01110 KDIYGNHILEPYCTFASPHNPRIDKPFTSG--------------------------------------------------
Os11g42390 KDIYQNHILEPYCTLASPHNPRIDKPFTSGGRQMLQL---------------------------QEDQDLHLSEISSECR
Os04g09720 RDVNDLHILEPRCEEDGISLMSDNSASSHDRR-----------------------------------TKLLESAVSSICR
Os02g46260 SEVLEPACPFDWPWPMPGRDASN---RKSLTEEHYW------------------------------LGDPPVEPPFSCFA
Os03g52080 SELDNGDILLDKCAGRLIPKPINGVSSRALLEEYSR------------------------------LSEPTARPTINCFS
Os11g24510 GEVSEAHILYKKCIYVSPKPDDGTIGRKILEEIVVG------------------------------NHRPPRPPMD----
Os12g39170 GESSGGHILYNYCIYDSDIDG----------SIQEK------------------------------RKIPPFPPRE----
Os10g01130 FAINPVHILEPICGFALRGRAMPETTMDQRLRLGLP----------------------------------------VECR
Os11g27200 KDINMLHVLEPLCEEVWSPRIHNTSATDGMSRLMLESARAADDDIIEFN-------------FPWIALDLRKSNLAYLLQ
Os02g26480 SGINMEHILCPPCRYQMG--------------------------------------------------------ITKEAN
 At3g25420 NLLNLYNILEPCYHGTSLSALDIEF--LPKSLLTLGKTEKPMAVRKRMFGRAWPLGAVVRPGIVPSWSQLLAGFG---VP
 At4g12910 NQLNIYNILEPCYHGTSLSAFDIRS--LPSSLLQLGKTEKRLPIRKRMFGRAWPVRAPVHPGIVPSWSQLLADVT---VP
Os04g25560 KDLNKYNILAPCYHHPEIQELEFKNSSLPSSFRKLGETEKRFPVRKRMAGRSWPLRAPVTRGRMTMWPELGGRS----LP
Os12g15470 NDLNIYDILEPCYHSKTIKKVTPANTKLPKSFQHLGTTTKPLAVRTRMHGRAWPLRAPVRAGRVPSWQEFARGSRPSGVP
Os02g02320 NSIFSSIMKLVGTKNYYDVR-----------------------------------------------------------K
Os07g29620 NLIFSSIETIIGKKNYYDIR-----------------------------------------------------------K
 At3g10410 NSLFSGVMSHAGGVNYYDIR-----------------------------------------------------------K
 At3g45010 NNIFQKIMDIAGNVNYYDVR-----------------------------------------------------------K
 At5g22980 TSIFNKIVAKKSGLNYYDIR-----------------------------------------------------------K
Os03g27480 ASSGHVDVYNFLLDTGMDPVAAG-------AAPARS-------------------------------FPPAYSAY--LDS
Os08g44640 EQANFIDFYNFLKDDSSSDANLEQQQRQRLLASLGQ-------------------------------SRRRYSSY--LSS
 At2g27920 SKSNFVDFYNFLLDTGMDPVSLTTSLKIKKEEKIKK-------------------------------YSRYLNDMRSLSD
Os03g27510 SKSDSVVKHVQLHDWHRHGHDANNGR-QHTGSCSSP-------------------------------LTRYLGRDIS---
Os03g27530 KSSGGVNIFNFLFNTSGVDLRVLAAEDKQKEVRGSP-------------------------------LMRFVGQDLSGSS
Os01g22970 LGPKGHKLSKKEKFVVGSIPCPMSSPIAQEQEWSKPYEEENLQVYTSITR------------------------------
```

```
Os01g11670 PYETGPVGKFVNRAEVKAALGARTD--------------VEWEDCSDTVGAAMHGDVMKSVKPGVEALLRG---------
Os05g50600 PYETGPVGKFVNRAEVKAALGARGD--------------VEWEECSDAVGAAMHGDVMKSVKPEVEALLRG---------
Os06g36570 PYETGPVGKFVNRVEVKAALGARGD--------------MEWEECSDAVGAAMHGDVMKSVKPKVEALLRG---------
Os05g50570 EAEAAALAPLLNGAEAKAALGARGD--------------AEWKMCSAAVGAAQHKDVMKSVKPEVEALLRR------GTT
Os05g50580 AVMAAGLADFLSTAEVKAAVGARPD--------------VAWEACSAAVGAAQEEDVMKSAKRDVEALLHRGASPTATPT
Os01g43890 GYPTRPLRDFLNTGEAKAALGARSD--------------VEWARCSEAVSAALADDIMRSARGDVEAVFLAPDG------
At1g15000 -YRTDLVVDLLNQREAKRVLGVSET--------------VRFEECSDEVEDVLRADVMKSVKFMVEYALER---------
At1g11080 PCLDDYARVFYNRADVQKSLHASDG--V---------NLKNWSICNMEIFNNWTGS--NPSVLPIYEKLIA-GG------
At4g15100 PCLDDYVKVYYNRADVQKALHASDG--V---------NLKNWSICNMEIFHNWTYV--VQSVLPIYQKLIA-GG------
At1g61130 PCLDDYAKVFYNRADVQKALHATDG--V---------HLKNWTICNDDILNHWNWTDSKRSVLPIYKKLIA-GG------
Os01g22980 PCYSSHIEDYMNKMDVQKSLHANTSGLI---------KDRKWSICSYSIFDNYDIT--VFSVLPIYSKLIK-AG------
Os05g06660 PCYSSYAEDYFNKHDVQEAFHANASGLL---------PG-KWQVCSDQILNSYNFS--VLSILPIYSKLIK-AG------
At3g17180 PCYSNYAEEYFNRVDVRLSLHATTR------------NVARWKVCNDSILQTYHFT--VSSMLPTYSKLIK-AG------
At5g08260 PCTESYAENYFNRKDVQVALHANVTN-----------LPYPYSPCSGVIK-RWSDA--PSTMIPIIQKLLT-GG------
Os04g44410 PCTEEYVKGYFNREDVQRALHANRTG-----------LSYPYSPCSEAIS-KWNDS--PSTVLPILKKLMG-AG------
At5g23210 PCASEYTEKYMNRKDVQEALHANVTN-----------ISYPWTHCSDTVS-FWSDA--PASMLPTLRTLVS-AG------
Os02g42310 PCTSDHAEVYFNRADVQEALHANVTN-----------IGYNWTHCSDVIG-KWRDA--PFSTLPIIRKLVA-GG------
Os10g39560 PCSQTYATEYFNRKDVQKALHANIP--------------GAYSLCHNSINRAWNDS--DMTVLPIVKKLTQ-SG------
Os06g51370 PCVDYRVIDYLNRGDVQAALHANVSG-G---------IPYSWAPCSDALTNWTDAP---PSTLPDIAALVR-AG------
At2g24000 PCTENYAEIYYNRPEVQRAMHANHTAI-----------PYKWTACSDSVFNNWNWRDSDNSMLPIYKELIA-AG------
At2g24010 PCTESYAEIYYNRPDVQRAMHANLTSI-----------PYKWTLCNMVVNN--NWKDSEFSMLPIYKELTA-AG------
At4g30610 PCTESYAEKYFNRPDVQRAMHANVTGI-----------RYKWTACSDVLIK--TWKDSDKTMLPIYKELAA-SG------
Os02g55130 PCTETYAEKYYNRPDVQKAMHANITGI-----------PYRWTACSDVLIK--TWRDSEFSMLPTYKLLMK-AG------
At3g02110 PCTERYAEIYYNRPDVQKALHANTTKI-----------PYKWTACSEVLNR--NWNDTDSTVLPIYREMIA-GG------
At3g07990 PCTERYSNVYFNRLDVQKALHANVTRL-----------SYPWKACSDIVGS--YWDDSPLSMLPIYKELIT-AG------
Os01g61690 PCTERYSSIYYNRPEVQIAMHANTTGI-----------QYSWKTCSDIVGS--YWADSPKSMLPIYQELIA-AG------
At2g35780 PCTEKYSGMYFNSPEVQKAMHANITGL-----------AYPWKGCSDIVGE--KWADSPLSMLPIYKELIA-AG------
Os01g06490 PCTEKYSTKYYNLPEVQKAFHANVTGI-----------PYAWTTCSDDLFY--YWKDSPRSMLPIYRELIA-AG------
At4g30810 PCTEKHTTVYFNLPEVQKALHVPPGLA-----------PSKWDTCSDVVSE--HWNDSPSSVLNIYHELIA-AG------
Os06g08720 PCTEKHSTVYFNLAEVQKALHVSPIIN-----------KSKWETCSDVVNT--NWKDCERSVLHIYHELIQ-YG------
At2g35770 ECVVGYTRKYMNDPNVHKSFHARLNG------------STPWTPCSRVIRK--NWKDSPKSMLPIIKNLLQ-AH------
Os09g28830 PCSNYYIHAYLNNPVVQKALHA-----R---------VTT-WLGCKKSYNMKRRKYISAKMQACGKYSNRT--V------
Os09g28840 PCIDYYIPRYLNNPDVQKALHA-----R---------ADTNWSGCN----------------------------------
Os07g46350 PCTDYYVEAYLNNPDVQKALHANIT--R---------LDHPWSACSG--VLRRWVDSASTVLPIIKELLKN--N------
Os03g26920 PCSRYYVESYLNRPEVQRTLHANTT--G---------LKQPWSGCSNIITPENWKDAPVSMLPSIQGLISS--G------
Os03g26930 PCTNHYVSSYLNNPEVQRALHANTT--G---------LNYPWMDCSGLIFDN-WKDSPETMLPSIKTLISS--G------
Os03g09190 PCSQYFVEAYMNQPQVQKTIHAN-T--E---------LKYPWTRCR-VYNLDHFGDSPKSMLPYIKAVITG--R------
At3g52000 PCSGNYLKAYLNIKEVQEAIHANTT--K---------IPYEWTSCNTKLLWEWNEKDRYVSLTPILQELMG-KG------
At3g52010 PCSKDYLKAYLNRKKVQKAIHANTT--K---------LPYEWTSCNNELTENWSENDRDTPMIPILHELMG-EG------
At2g05850 PCRSNYVKAYLNSENVQEAMHANTT--K---------LPYEWKACNHYLNSVWIDADKDASMVPILHDLMG-EG------
At3g52020 PCGMQYIAAYLNREDVQRSMHV--T--K---------LPHTWMLCNEATGFNWNQTDYSASMLPILKELMKHDQ------
At3g63470 PCSDHYVQAYLNRPEVQAALHANAT--K---------LPYEWQPCSSVIKKWN---DSPTTVIPLIKELMG-QG------
At1g28110 VCVEDETVNYLNRRDVQEALHARLIG------------VREWTVCSNVLDYQLLDVEIPTINIVGSLVKAG---------
At2g33530 VCLEDETVNYLNRRDVQKALHARLVG------------TRKWTVCSDVLDYEVLDVEVPTINIVGSLVKAG---------
Os04g32540 VCVEDETMGYLNRKDVQEAMHARLEGG-----------VPKWTVCSSVLEYKQLDLQIPTINIVGGLVKSG---------
Os11g31980 VCVEDETVNYLNRKDVQEALHAKLIG------------VKNWAVCSSVLEYELLNLQIPTINIVGSLVKSG---------
At2g12480 ------------------ALHANRTRL-----------PYEWTMCSNRLNYSGIDGYIDMLPSLKRIIQNQ---------
At5g42240 VCMTYERRFYFNLPEVQKALHANRTHL-----------PYSWSMCSGVLNYSDIDGNIDMLPILKRIILNK---------
At5g42230 VCMNYERQFYLNIPEVQMALHANRTNL-----------PYSWSLCSNLLNYSAIDVNTNMLPTLKRIIQNK---------
At1g43780 VCMSFEEQLYLNLPEVQKALHANRTKL-----------PYEWSMCSSLLNYKYTDGNANMLPILKRIVKSK---------
Os11g10750 VCMTYERYFYFNLPEVQQALHANRTHL-----------PYGWSMCSDVLNYSNKDGNINILPLLQRIVEHK---------
At1g73300 IYRYLLTTYWANDATVREALQINKE------------SIGEWVRCY---RTIPYDNDIK-SSMPYHVNNSISG-------
At5g36180 IYRYLLTTYWANDATVREALQINKE------------SIGEWVRCY---YSIPYNNDIK-SSMPYHVNNSISG-------
At1g73280 IYRFLLAAYWANDETVRKALQIKKE------------TIGEWVRCH---YGIPYNYDIK-SSIPYHMNNSING-------
At1g73310 SYRFMLTTYWANDETVRKALQINKE------------SIGEWTRCY---RGIPYNHDIK-SSVPYHMNNSIDG-------
At1g73290 IYRYLLTTYWVNDVNVRKALQINKE------------SIGEWVRCY---FGIPYTHDIK-SSVPYHMNNSING-------
At1g73270 IYRYSLSHYWVNDETVRKALQINKE------------SIREWKRCD---WSKPYTKDII-SSVPYHMNNSING-------
At3g10450 MYRYLLTTYWANDENVQRALHVNKG------------SIGEWVRCY---FEIPYNHDIK-SSVPYHMNNSIDG-------
At2g22920 LYPYYLLGYWINDESVRDALHVNKS------------SIGKWERCTY-QNRIPYNKDIN-NSIPYHMNNSISG-------
At2g22970 LYPFYLISFWANDESVRDALHVNKR------------SIGKWERCNY-LS-KPYNKDIK-SSVPYHMNNSVSG-------
At2g23000 YYLYFLIECWANNERVREALHVTKG------------TKGQWQRC---NWTIPYDNNII-SSVPYHMDNSING-------
At2g23010 YYPYHLVECWANNESVREALHVDKG------------SIGEWIRD---HRGIPYKSDIR-SSIPYHMNNSING-------
At2g22990 YYPYHLIECWANDESVREALHIEKG------------SKGKWARC---NRTIPYNHDIV-SSIPYHMNNSISG-------
At2g22980 LYRYTLITFWANNKSVREALQVNKG------------SIGKWVQCN--YKNISYNYDIK-SSVAYHMKNSIDG-------
At3g12230 TYRYLLSIYWANNEIVRRALKVVEG------------SKGKWERCD---LSVRSNQDIK-SSIPYHMNNSIKG-------
At3g12240 TYRYLLSEYWADNETVRRALKVVKG------------SKGTWERCD---YRVLSNQDIK-SSIPFHINNSIRG-------
At3g12220 SYRSMLSDYWANNESVRRALKVVEG------------TTGRWERCK---WTLQNNKDIK-SSIPYHKKNSIQG-------
At3g12203 ANLHSLSEYWANEKSVRKALLVNEG------------TVRKWIRCN---TEIAYNKDIR-SSVPYHKYISIEG-------
At1g33540 MYRYLLASHWANDEDVRRELHVVKG------------SIGKWMRCN---WDLPYEKDIK-SSVPYHRNNSIIGD------
At5g09640 TYRYFLSAFWANDENVRRALGVKK-------------EVGKWNRCN--SQNIPYTFEIF-NAVPYHVNNSLKG-------
Os10g01110 TAEYTMSRIWANNDTVREALGIHQG------------TVPSWQRCN---YDILYTYDIK-SSVRYHLDLTTRG-------
Os11g42390 TARYTMSRIWANNDTVREALGIHQG------------TVPSWQRCN---FDIPYTRDIK-SSIRYHLDLTARG-------
Os04g09720 NATYVLSKIWANDEAVRESLGIHKG------------TVTTWERCN---HDLLYKKQIV-SSVEYHLSLITQG-------
Os02g46260 AYRYYLSYFWANDNATRAALGIKEG------------TVTEWIRCP--TGLP-YTRDLP-SSIECHFNVTTRG-------
Os03g52080 -YRFYLLNIWMNDKATRDALKIKKG------------TVGVWTRCN--TEVFPYARDVP-STIQYHLNLTTRG-------
Os11g24510 ----------------------CSG------------TVDEWVRCH--DDGLPYSQDIE-SSIKYHQNLTSRG-------
Os12g39170 ----------------------CIG------------SVDEWLRCH--NGDLPYSMDIK-SNIKFHHNVTTKG-------
Os10g01130 DNGYRLSYLWADDPEVRATLGIHEG------------SIASWSRCT---ALPLFRHDVD-SAIPYHAELTQRG-------
Os11g27200 KASYVVLKIWANDKTVRESLGVHKG------------TVGKWTRCN---YDIDYIKDVY-STVEYHLTLMREG-------
Os02g26480 EEYDFGQMFELLSESSEYGLECNIE------------ILQKWKRCP---NFIQYTRDIP-TLTEYHLNVTSKG-------
At3g25420 CIDDTVATKWLNDPAVRKAVHAKEVSIQFIIFLSISISIGNWELCS---SNLEYRHDTG-SMIEYHRNLTLSG-------
At4g12910 CIDDRVATAWLNDPEIRKAIHTKEE-----------SEIGRWELCS---GKLSFYHDAG-SMIDFHRNLTLSG-------
Os04g25560 CTSDELANAWLDDEDVRAAIHAEPK-----------SLIGSWELYT---ARIEYYHDTGDSMVKYHKKFTAMG-------
Os12g15470 CMSDEVATAWLNNDDVRAAIHAQPV-----------SSIGSWLICT---NVLDFIHDAG-SMISYHKNLTGQG-------
Os02g02320 ECEGKLCYDFSNLEKFFGDKAVKEAIG-----------VGDLEFVSCSTTVYQAMLTDWMRNLEVGIPALLEDG------
Os07g29620 PCVGSLCYDLSNMEKFLQLKSVRESLG-----------VGDIQFVSCSPTVYQAMLLDWMRNLEVGIPELLEND------
At3g10410 KCVGSLCYDFSNMEKFLNLQSVRKSLG-----------VGDIDFVSCSTSVYQAMLVDWMRNLEVGIPTLLEDG------
At3g45010 QCEGSLCYDFSNMENFLNQKSVRKALG-----------VGDIEFVSCSTAVYEAMQMDWMRNLEVGIPALLQDG------
At5g22980 KCVGSLCYDFSRMEIFLNKENVRKALG-----------VGDIKFVSCSSTVYDAMIEDWMQNLEVKIPSLVNDG------
Os03g27480 KLSVGDSIRSVMNGAIKEKLKIIPK-------------DVVWEEQSYTVYNALINDFMKPRIQEVDELLSYGVN------
Os08g44640 KVTTEGGFEGIMNTVIRDKLRIIPK-------------NVTWSEQSDDVFEALAGDFMKPRILEVDELLNLGLN------
At2g27920 VEDVEGDLDKLMNGVIKKKLKIIPN-------------DLIWGNNSDDVFTAMEAAFMKPVIEDVDELLATGVD------
Os03g27510 ---------TIMNGVIKRKLKIIPK-------------DLVWQQFSLDVYEAMKNDFMRPAINVVDELLSLGVN------
Os03g27530 GPNTI---EGIMNGVIKEKLKIIPN-------------NLVWQLATVAVFNALENEFMKPAINEVDELLSLGVN------
```

```
Os01g11670 -TRVLLYQGIRDLNLRDGVVSTEAWMRELKWDGLTAFLVA--------------DRAVWR-IGEE---LAGYVQRSGP--
Os05g50600 -TRVLLYQGIRDL--RDGVVSTEAWMRELEWDGLPAFLDA--------------DRAVWR-IGEE---LAGYVQRSGP--
Os06g36570 -TRVLLYQGIRDL--RDGVVSTEAWMRELKWDGLAVFLDA--------------DCAVWR-IGEE---LAGYVQRSGP--
Os05g50570 PTRVLLYGGIRDV--KDGPVSTEAWVRELEWDGLAAFQDA--------------DRAVWRHRGGE---LAGSVQRHGA--
Os05g50580 PTRVLLYEGIRDV--GNGPVCAEAWLRELEWDGLAAFQDA--------------GRAVWRSGGG----LAGYVQRHDA--
Os01g43890 -VRVLLFQGVFDL--HSGPASVEAWVRELAWPGLGAFLAA--------------ERAVWRLGDEQ---LAGYVQRSGA--
At1g15000 -TQVLLYQGMLDL--RDGVVSTEEWMKTMNWSGLGMFSTA--------------ERRVWKDEDGV---VAGYVQRWGN--
At1g11080 -LRIWVYSGDTDG--RVPVLATRYSLNALELP----IKTA---------------WRPWYHEK----QVSGWLQEYEG--
At4g15100 -LRIWVYSGDTDG--CIPVLGTRYSLNALGLP----IKTA---------------WRPWYHEK----QVSGWVQEYDG--
At1g61130 -FRVWVYSGDTDG--RVPVLSTRYCINKLELP----IKTA---------------WRPWYHET----QVSGWFQEYEG--
Os01g22980 -LRIWVYSGDVDG--RVPVIGSRYCVEALGLP----VKSQ---------------WQPWYLNN----QVAGRFVEYQG--
Os05g06660 -LRVWLYSGDADG--RVPVISSRYCVEALGLP----IKTD---------------WQSWYLDK----QVAGRFVEYHG--
At3g17180 -LKIWVYSGDADG--RVPVIGSRYCVEALGIS----VKSE---------------WRSWFHNH----QVGGRITEYEGG-
At5g08260 -LRIWIYSGDTDG--RVPVTSTRYSIKKMGLK----VESP---------------WRSWFHKS----QVAGWVETYAGG-
Os04g44410 -LRIWVYSGDTDG--RVPVTSTRYSLNTMKLR---PRLMRKTAGDGAGEESEWGGWRAWYDRQ----QVGGWAVEYEEG-
At5g23210 -LRVWVFSGDTDG--RIPVTATRYSLKKLGLK----IVQD---------------WTPWYTKL----QVGGWTVEYDG--
Os02g42310 -IRVWVFSGDTDG--RIPVTSTRLTLNKLGLK----TVQE---------------WTPWYDHQ----QVGGWTILYEG--
Os10g39560 -LRIWIYSGDTDA--RIPTTSTRYTLKKLGLP----IKED---------------WSPWFHHK----QVGGWSVVFDG--
Os06g51370 -LRVWVFSGDTDD--RVPVTSTRYALRKLKLKTVRP-------------------WKQWFTSD----QVGGYTVLYDG--
At2g24000 -LRIWVYSGDTDS--VIPVTATRYSLGKLNLRVKT-------------------RWYPWYSGN----QVGGRTEVYEG--
At2g24010 -LRIWVFSGDTDA--VVPVTGTRLALSKLNLPVKT-------------------PWYPWYSEK----QVGGWTEVYEG--
At4g30610 -LRIWIFSGDTDS--VVPVTATRFSLSHLNLPVKT-------------------RWYPWYTDN----QVGGWTEVYKG--
Os02g55130 -LRIWVFSGDTDS--VVPVTATRFALSHLGLKTKI-------------------RWYPWYSAG----QVGGWSEVYEG--
At3g02110 -IRVWVFSGDVDS--VVPVTATRYSLARLSLSTKL-------------------PWYPWYVKK----QVGGWTEVYEG--
At3g07990 -LKIWVFSGDTDA--VVPITATRYSVDALKLATIT-------------------NWYPWYDHG----KVGGWSQVYKG--
Os01g61690 -IRIWVFSGDTDA--VVPVTATRYSIDALKLPTMV-------------------NWYPWYDHG----KVGGWSQVYKG--
At2g35780 -LRIWVFSGDTDS--VVPITGTRYSIRALKLQPLS-------------------KWYPWNDDG----QVGGWSQVYKG--
Os01g06490 -LRIWVFSGDADS--VVPLTATRYSIDALYLPTVT-------------------NWYPWYDDE----EVAGWCQVYQG--
At4g30810 -LRIWVFSGDADA--VVPVTSTRYSIDALNLRPLS-------------------AYGPWYLDG----QVGGWSQQYAG--
Os06g08720 -LRIWVFSGDTDA--VLPVTSTRYSINALKLPTVT-------------------PWNAWYDDDG---EVGGWTQGYKG--
At2g35770 -LRIWIFSGDSDA--VLPLSGTRHSINAMKLKSSK-------------------RWYPWYHSHG---LVGGWSQVYEDGL
Os09g28830 -TSTIFLSGDLDS--VCPLTATRYSVGDLGLAVTE-------------------PWRPWTAN----REVGGYVQQYTGG-
Os09g28840 --------GDMDS--ICSLTATRYSVKDLNLTITH-------------------KWRPWYTPD---NEVGGYVQQYEGG-
Os07g46350 -IRVWVYSGDTDG--RVPVTSSRYSVNQLNLPVAA-------------------KWRPWFSNTQGVGDVGGYIVQYKGN-
Os03g26920 -VSTWLYSGDIDA--VCPVTSTLYSLDILELPINS-------------------SWRPWYSDDN---EVAGYVVGYKG--
Os03g26930 -TRIWLYSGDMDA--VCSVTSTQYALDILGLPVET-------------------SWRPWR-IDN---EVAGYVVGYRG--
Os03g09190 -IRIWIFSGDLDA--MVPVTATRQSMERLQLRVAA-------------------DWRPWSADGK---DVAGYVIAYDG--
At3g52000 -VRVMLYNGDVDL--VIPFTSTLAVVKTMNLTVVK-------------------EWRPWFTGGH----VGGFTEDYKGN-
At3g52010 -VRVMIYNGDVDL--EIPFASTLAVVKEMNLTVVK-------------------EFRPWFTGGQ----LGGFTEDYKGN-
At2g05850 -VRVLVYSGDVDA--AIPFTATMAVLKTMNLTVVN-------------------EWRPWFTGGQ----LGGFTEDYERN-
At3g52020 -LRVWVYTGDTDT--VIPLTVTMHALKMMNLTAVT-------------------DWLPWFSEGQ----VGGFTEEYKGN-
At3g63470 -VRVWVFSGDTDG--RIPVTSTKYSLKKMNLTAKT-------------------AWHPWYLGGE----VGGYTEEYKGK-
At1g28110 -VPVLVYSGDQDS--VIPLTGSRTLVSRLAKQLGLRTSVP---------------YRVWFAGQQ----VGGWTQVYG-NV
At2g33530 -VPVFVYSGDQDS--VIPLTGSRTLVKRLAEELGLRTTVP---------------YRVWFAGQQ----VGGWTQVYG-NT
Os04g32540 -VPVLVYSGDQDS--VIPLTGSRTVVQRLAGWLRLGTTAAG--------------YRVWFEGRQ----VGGWTQSFGGGA
Os11g31980 -IRVLVYSGDQDS--VIPLTGSRTLVQNLARDLGLKTSVP---------------YRVWFEGQQ----VGGWTQVYG-DM
At2g12480 -TPVWIFSGDQDS--VIPLQSSRTLVRELAEDLNFKTTIP---------------YGAWFHKEQ----VGGWVTEYG-NL
At5g42240 -TPIWIFSGDQDS--VVPFGGSRTLVRELAQDLNFKTTVP---------------YGAWFHKSQ----VGGWAIEYG-KL
At5g42230 -IPVRIFSGDQDS--VVPFLGTRTIVGELANDLNFKTTVP---------------YGVWFHKRQ----VGGWAIEYG-NL
At1g43780 -VPVWVFSGDEDS--VIPLLGSRTLVKELADDLNFNTTVP---------------YGAWFDKGQ----VGGWVVEYG-NL
Os11g10750 -IPVWVFSGDQDS--VVPLLGSRTLVRELAHDMGLHVTVP---------------YSSWFRRGQ----VGGWVTEYG-NI
At1g73300 -YRSLIYSGDHDL--EVPYLGTQAWIRSLNYSIID-------------------DWRPWMIK----NQIAGYTRTYANK-
At5g36180 -YRSLIYSGDHDF--EVPYLGTQAWIRSLNYSIID-------------------DWRPWMVK----NQIAGYTRTYANK-
At1g73280 -YRSLIYSGDHDF--EVPFLGTQAWIRSLNYSVID-------------------DWRPWMIK----DQIAGYTRTYANK-
At1g73310 -YRSLIYSGDHDI--QVPFLGTQAWIRSLNYSIID-------------------DWRPWMIK----DQIAGYTTSYVNK-
At1g73290 -YRSLIYSGDHDL--NVPFLATQAWVRSLNYSIID-------------------NWRPWMIK----DQIGGYTKTYANK-
At1g73270 -YRSLIFSGDHDF--EVPLIGTQVWIKSLNYAIVD-------------------KWRPWMIN----NQVAGYTRTYANK-
At3g10450 -YASLIFSGDHDM--EVPYLGTQAWIRSLNYSLID-------------------DWRPWMIG----DQIAGYTRTYANK-
At2g22920 -YRSLIYSGDHDL--VVPFLATQAWIKSLNYSIIH-------------------EWRPWMIK----DQIAGYTRTYSNK-
At2g22970 -YRSLIYSGDHDL--VVPFLATQAWIKSLNYSIID-------------------EWRPWMIR----DQITGYTRTYSNK-
At2g23000 -YRSLIYSGDHDI--TMPFQATQAWIKSLNYSIVD-------------------DWRPWMIN----DQIAGYTRTYSNK-
At2g23010 -YRSLIFSGDHDI--TMPFQATQAWIKSLNYSIID-------------------DWRPWMIK----GQIAGYTRTYSNK-
At2g22990 -YRSLIYSGDHDI--AVPFLATQAWIRSLNYSPIH-------------------NWRPWMIN----NQIAGYTRAYSNK-
At2g22980 -YRSLIYNGDHDM--MVPFLATQAWIRSLNYSITD-------------------DWKPWMIN----DQIAGYTRSYSNK-
At3g12230 -YRSLVISGDHDM--TIPFLGTQAWIRSLNYSITE-------------------KWRPWMIL----DQVAGYTKTYANK-
At3g12240 -YRSLVISGDHDM--TIPFLGTQAWIRSLNYSITE-------------------KWRPWMIL----DQVAGYTKTYANK-
At3g12220 -YRSLIFSGDHDM--LTPYVGTQDWIRSLNYSIID-------------------KWRPWMIL----DQVAGYTTTYANK-
At3g12203 -YRSLVFSGDHDM--LVPFLGTQAWIRSLNYSIVD-------------------DWRPWMVQ----NQVAGYTRTYANK-
At1g33540 -YRSLVYSSDHDM--MVPYIGTEAWIKSLNYSITD-------------------DWRPWFVN----NQGG----------
At5g09640 -FRSLIYSGDHDS--MVPFSSTQAWIRALNYSIVD-------------------DWRPWMMSS---NQVAGYTRTYANK-
Os10g01110 -YRSLIYSGDHDM--IIPFIGTQAWIRSLNFSVVD-------------------EWRPWFVD----GQVAGYIRSYSNN-
Os11g42390 -YRSLIYSGDHDM--AIPFIGTQAWIKSLNFSVVD-------------------KWRPWFVD----GQVGGYTRSYSNN-
Os04g09720 -YRGLVYSGDHDS--VVSLIGTQGWLRSLNLSITH-------------------GWRPWYVN----SQVVGFTRTYSNN-
Os02g46260 -YRALVYSGDHDP--IVPFSGTQAWIRSLNFSIVD-------------------DWRAWHLD----GQAAGFTIKYANN-
Os03g52080 -YRALVFCGDHDL--MVPFLGTQAWIRSLNFTIID-------------------DWRAWHLD----GQAAGFTVMYDNN-
Os11g24510 -YRVLVYSGDHDS--VVPFLGTQAWVRSLNYPIVD-------------------DWRAWHID----GQSAGFTITYAND-
Os12g39170 -YRALVYSGDHDA--MIPFLGTQAWVRSLNFSVVD-------------------DWRAWHVD----GQSAGFTITYMNN-
Os10g01130 -YRALVYNGDHDL--DMTFVGTQQWIRTLGYNVVT-------------------AWRPWYSN----RQVAGFTTVYDHN-
Os11g27200 -YRSLIYSGDHDC--GIPFTSTQAWIRFLNLSVVD-------------------DWRPWYVA----GQVAGTTQTLTTH-
Os02g26480 -YRVFLYSGDHAL--LVPFSATLEWLKTLNYKEIE-------------------KWHPWFVE----KQIAGYSVRYENN-
At3g25420 -FRALIFSGDHDM--CVPYTGSEAWTKAMGYKVVD-------------------EWRPWMSN----NQVAGFTQGYANN-
At4g12910 -YRALIYSGDHDM--CVPFTGSEAWTKSLGYKVID-------------------EWRAWISN----DQVAGYTQGYANN-
Os04g25560 -YRALIYSGDHDL--CIPYVGTEAWVRSMGYRVID-------------------HWRPWYFG----GQVAGYTQGYEHN-
Os12g15470 -YRAFIYSGDHDM--CVPYTGTEAWTRSLGYGVID-------------------SWRPWHLN----GQVSGYTQGYEHG-
Os02g02320 -INVLIYAGEYDL--ICNWLGNSRWVHSMEWSGQKDFVSS--------------HESPFVVDGA------EAGVLKSHGP
Os07g29620 -IKVLIYAGEYDL--ICNWLGNSRWVNSMEWSGKEAFVSS--------------SEEPFTVDGK------EAGILKSYGP
At3g10410 -ISLLVYAGEYDL--ICNWLGNSRWVNAMEWSGKTNFGAA--------------KEVPFIVDGK------EAGLLKTYEQ
At3g45010 -IKLLVYAGEYDL--ICNWLGNSKWVHEMEWSGQKEFVAA--------------ATVPFHVDNK------EAGLMKNYGS
At5g22980 -INLLVYAGEYDL--ICNWLGNSRWVDQMNWSGQKGFGSA--------------KNVSFLVDGK------EAGLLKNHGP
Os03g27480 ---VTVYNGQLDV--ICSTVG--AEAWVQKLKWDGLKNFLRL------------PRQPLHCGSSEVT--KGFVRSYKN--
Os08g44640 ---VTIYSGQLDL--ICATKG--TLDWIQKLKWDGLKNFTNS------------RRVPLYCSGGEAAGTQAFLKSYKN--
At2g27920 ---VTIYNGQLDV--ICSTSG--TEAWVHKLRWEGLEEFKKM------------EREPLFCESDRAT--RGFTKSYKN--
Os03g27510 ---VTLYNGQ---R--KP---------LH--------------------FCLPYYIP-----------NGFVKAHKN--
Os03g27530 ---VTVYNGQ------RYTHTIEQCELLICAMGLVGWNGLNDFFRV-LREPMHYFCYPGHDSTAFRRIYKN------LQY
```

```
Os01g11670 LSHVIVDGAGHLVPADNGRVAQEMIEDWVLQ
Os05g50600 LSHVVVYGAGHLMPADNGRAAQEMIEDWVLQ
Os06g36570 LSHVVVYGAGHLLPADNGHAAQEMVKDWVLQ
Os05g50570 LVNVAVYGAGHFVPFSQGRAAQEMIEDWVFG
Os05g50580 LVHVAVYGAGHFVPYSQGRVAQEMIEDWVFR
Os01g43890 LANAVIVGAGHMAAGDNRPAAQAMIEGWVLQ
 At1g15000 LCHVAVTGAGHFVPTDKAVNSRDMIEGWVLG
 At1g11080 LTFATFRGAGHAVPCFKPSSSLAFFSAFLSG
 At4g15100 LTFATFRGAGHTVPSFKPSSSLAFISAFVKG
 At1g61130 LTFATFRGAGHDVPSFKPSESLAFFSAFLNG
Os01g22980 LTMATVRGAGHAVPQDKPEQALVVINSFLSG
Os05g06660 MTMVTVRGAGHLVPLNKPAEGLMLINAFLHG
 At3g17180 LTFVTVRGAGHLVPLNKPEEALALFRSFLNG
 At5g08260 LNFVTVRGAGHQVPALAPAQSLTLFSHFISS
Os04g44410 LTLVTVRGAGHQVPLFAPRRSLAMLYHFLRG
 At5g23210 LMFVTVRGAGHQVPTFKPREALQLIHHFLGN
Os02g42310 LTFVTIRGAGHEVPLHAPRQALSLFSHFLAD
Os10g39560 LTFVTVRGAGHMVPSIMPEQALELFKYFLAN
Os06g51370 LTFVTIRGAGHMVPMITPVQARQLFAHFLAG
 At2g24000 LTFVTVRGAGHEVPFFQPQSALILLRSFLAG
 At2g24010 LTFATIRGAGHEVPVLQPERALTLLRSFLAG
 At4g30610 LTFATVRGAGHEVPLFEPKRALILFRSFLAG
Os02g55130 LTFASVRGAGHEVPLFQPRRAFRMFQSFLAG
 At3g02110 LTFVTVRGAGHEVPLFKPRAAFELFKYFLRG
 At3g07990 LTLVTVAGAGHEVPLHRPRQAFILFRSFLES
Os01g61690 LTLVTIAGAGHEVPLHRPREALILFRHFLQN
 At2g35780 LTLVTIHGAGHEVPLFRPRRAFLLFQSFLDN
Os01g06490 LTLVTIRGAGHEVPLHRPRQALKLFEHFLQD
 At4g30810 LNFVTVRGAGHEVPLHRPKQALALFKAFISG
Os06g08720 LNFVTVRGAGHEVPLHRPKQALILIKSFLAG
 At2g35770 LTYTTVRAAGHEVPLSQPRLALFLFTHFLAN
Os09g28830 LVFISVRGAGHQVPYFQPEKALIVVSSFLRG
Os09g28840 FTLASVRGAGHLVPSFQPKRSLVLLYSFLKG
Os07g46350 LSLVTVRGAGHEVPSYQPQRALVLVQYFLEG
Os03g26920 LVFATVRESGHMVPTYQPQRALTLFSSFLQG
Os03g26930 LVFATVRGAGHMVPYYQPRRALALLSSFLEG
Os03g09190 LVFATVRGSGHMAPIDQPERALVLVSSFIRG
 At3g52000 LTFVTVKGAGHSVPTDQPIHALNIFTSFIRN
 At3g52010 LTFVTVKGAGHSVPTDQPIHALNIFTSFIRN
 At2g05850 LTYATVKGSGHSVPLDQPVHALNLFTSFIRN
 At3g52020 FRYATVIGAGHEVPLYKPKAALTLFKHFIRN
 At3g63470 LTFATVRGAGHQVPSFQPKRSLSLFIHFLND
 At1g28110 LSFATVRGASHEVPFSQPERSLVLFKAFLDG
 At2g33530 LAFATVRGAAHEVPFSQPARALVLFKAFLGG
Os04g32540 LSFATVRGASHEAPFSQPERSLVLFAAFLAG
Os11g31980 LSFATIRGASHEAPFSQPERSLVLFRAFLQG
 At2g12480 LTFATVRGAAHMVPYAEPSRALHMFSSFMNG
 At5g42240 LTFATVRGAAHMVPYAQPSRALHLFSSFVSG
 At5g42230 LTFATVRGAAHAVAYTQPSRALHLFSTFLRG
 At1g43780 LTFATVRGAAHMVPYSQPSRALHLFTSFVLG
Os11g10750 LTFATVRGASHMVPFAQPDRALGLFQSFALG
 At1g73300 MTFATIKGGGHTIE-FKPEEASIMFQRWING
 At5g36180 MTFATIKGGGHTAE-SKPEEASIMFQRWING
 At1g73280 MTFATIRGGGHTIE-FKPEEASIMFQRWIKG
 At1g73310 MTFATVTGGGHTAE-FTPKETFMMFQRWING
 At1g73290 MTFATVRASGHTAE-YKPYETYIMFHRWING
 At1g73270 MTFATVKGGGHTAE-YKPDETFIMFQRWING
 At3g10450 MAFATIKGGGHTPE-YKPEESYIMFQRWISG
 At2g22920 MTFATVKGSGHTAE-YKPNETFIMFQRWISG
 At2g22970 MTFATVKGSGHTAE-NKPQESFIMFRRWING
 At2g23000 MTFATVKGGGHTAE-YLPNESSIMFQRWISG
 At2g23010 MTFATVKGGGHTAE-YLPEESSIMFQRWISG
 At2g22990 MTFATIKGGGHTAE-YRPNETFIMFQRWISG
 At2g22980 MTFATIKGSGHTAE-YKPKETSIMFKRWISA
 At3g12230 MTLATVKGGGHTLE-YKPEENSILFKRWISG
 At3g12240 MTLATVKGGGHTLE-YKPEENSVLFKRWISG
 At3g12220 MTFATVKGGGHTLD-YKPDENSILFKRWISG
 At3g12203 MTFATVKGGGHTSE-YKPVETYIMIKRWLSG
 At1g33540 ---------GHTAE-YKPEESFMMFQRWISG
 At5g09640 MTFATIKGGGHTAE-YTPDQCSLMFRRWIDG
Os10g01110 LTFATVKGGGHTAPEYMPKQCLAMLARWVSG
Os11g42390 LTFATVKGGGHTAPEYMPKQCLTMLASGFFS
Os04g09720 LTYATVKGAGHTAPEYMPKECLAMVDRWLSG
Os02g46260 LTFATIKGGCHIPPENRPKESFIMAKRWLAG
Os03g52080 LTFATLKGSGHAPISYKPKQGFAMGQRWLDH
Os11g24510 LTFATVKGGGHTAPEYQPERCLAMFGRWISE
Os12g39170 MTFATVKGGSHTVPEFEPERSLAMFKRWISN
Os10g01130 LTFATVKGGGHTAPEYRPKECLDMLDRWTSP
Os11g27200 A-----------NP--TP---ISIFEDWASK
Os02g26480 ILFATIKGAGHVPSDYLPFEVFVAYQRWIDG
 At3g25420 LTFLTIKGAGHTVPEYKPRESLDFYSRFLAG
 At4g12910 LTFLTIKGAGHTVPETNRG------------
Os04g25560 LTFLTIKGAGHTVPEYKPKETLAFYSHWLSG
Os12g15470 LTFATIKGAGHTVPEYKPQESLAFYSRWLAG
Os02g02320 LSFLKVHNAGHMVPMDQPKASLEMLRRFTQG
Os07g29620 LSFLKVHDAGHMVPMDQPKVALEMLMRWTSG
 At3g10410 LSFLKVRDAGHMVPMDQPKAALKMLKRWMEN
 At3g45010 LTFLKVHDAGHMVPMDQPKAALQMLQNWMQG
 At5g22980 LTFLKVYNAGHMVPMDQPKASLQMLQNWMQG
Os03g27480 LHFYWILGAGHFVPVDQPCIALDMIGSITQS
Os08g44640 LKFYWILGAGHMVPIDNPCPALKMLGDITQS
 At2g27920 LHFYWILGAGHFVPVDEPCVALKMVGEITKS
Os03g27510 LQHYSILQAGQAVPVDQPCTVLHMIGAIMQS
Os03g27530 YWILEAGHVLAT-------------------
```

b)

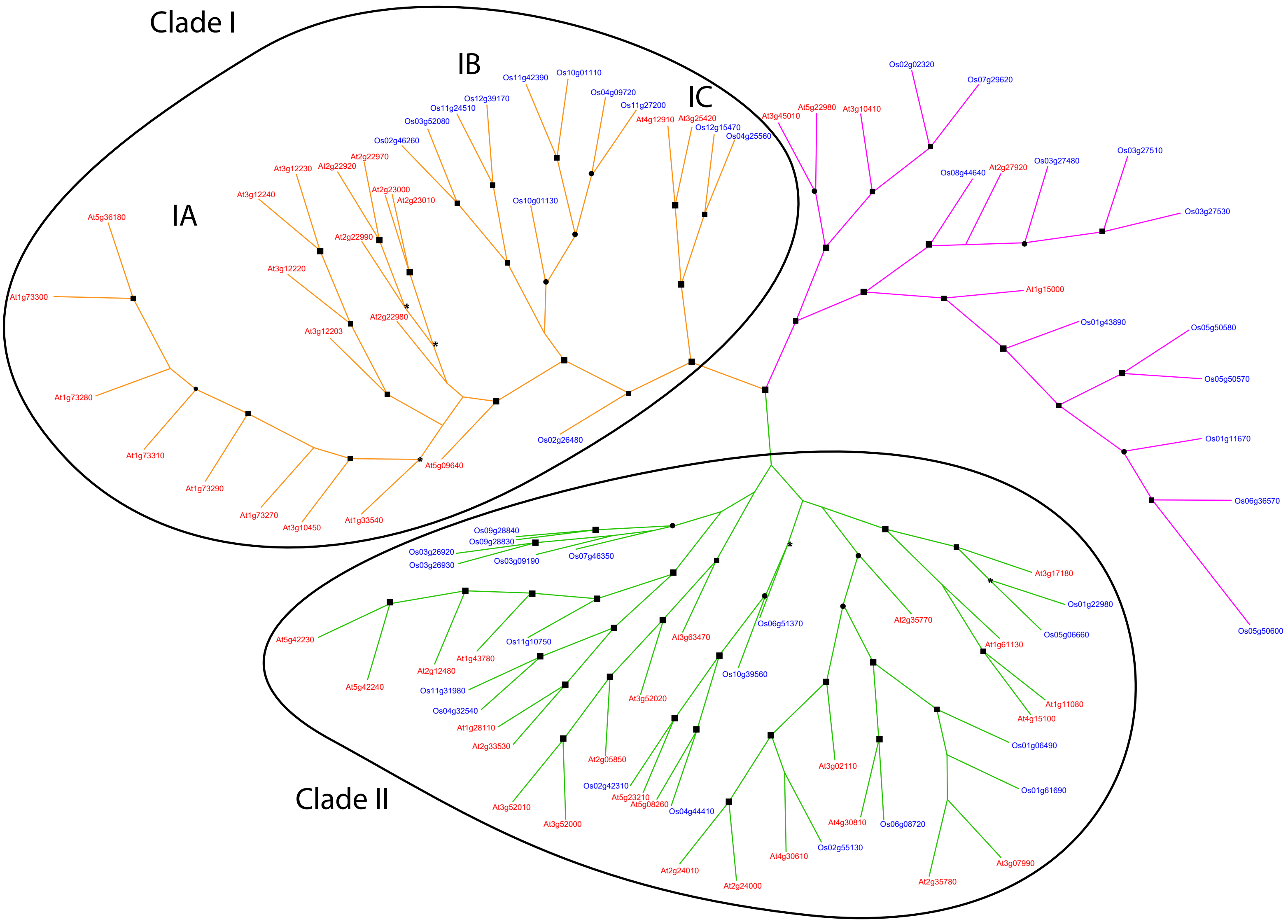

Clade I
IA
IB
IC
Os11g42390
Os10g01110
Os12g39170
Os11g24510
Os04g09720
Os11g27200
Os03g52080
Os02g46260
At4g12910
At3g25420
Os12g15470
Os04g25560
At3g12230
At2g22920
At2g22970
At3g12240
At2g23000
At2g23010
Os10g01130
At5g36180
At2g22990
At3g12220
At1g73300
At2g22980
At3g12203
At1g73280
Os02g26480
At1g73310
At5g09640
At1g73290
At1g73270
At3g10450
At1g33540
Os02g02320
Os07g29620
At3g45010
At5g22980
At3g10410
Os08g44640
At2g27920
Os03g27480
Os03g27510
Os03g27530
At1g15000
Os01g43890
Os05g50580
Os05g50570
Os01g11670
Os06g36570
Os05g50600
Os09g28840
Os09g28830
Os03g26920
Os03g26930
Os03g09190
Os07g46350
At3g17180
Os01g22980
Os05g06660
At5g42230
At2g12480
At1g43780
Os11g10750
At3g63470
Os06g51370
At2g35770
At1g61130
At5g42240
Os11g31980
Os04g32540
At3g52020
Os10g39560
At1g11080
At4g15100
At1g28110
At2g33530
At2g05850
Os01g06490
At3g02110
Os02g42310
At5g23210
At5g08260
Os04g44410
Os01g61690
At3g52010
At3g52000
At4g30810
Os06g08720
Os02g55130
At4g30610
At2g24010
At2g24000
At3g07990
At2g35780
Clade II
