# Supplementary material for: Cross genome comparisons of serine proteases in Arabidopsis and rice
Source: BMC Genomics. 2006 Aug 9;7:200. doi: 10.1186/1471-2164-7-200 (PMC1560137; doi:10.1186/1471-2164-7-200)
Supplement: Additional file 9 — Figure SF5. Pairwise sequence alignment of Arabidopsis and rice serine beta-lactamase -like proteins. Pairwise sequence alignment of the serine beta-lactamase domain region of the annotated Arabidopsis and rice serine beta-lactamase-like proteins. The catalytic residues are indicated. Gene names correspond to those in Additional files 1 and 2. For brevity, rice gene names have been shortened to OsXXg##### instead of LOC_OsXXg#####, XX referring to chromosome 1–12 and a 5 digit number assigned to each gene. [file 1471-2164-7-200-S9.pdf]

```
            :*:** **** :**********************.**:********************:****:****** **: ::***
At5g24810   VRKLLAELGSIQKILGIQVCAYKDGKVIIDTAAGVLGRYDPRPVQPDSLFPVFSVTKGVTAGMIHWLVDKRKLQLDQTVA
Os06g48770  LRNLLLELGS-NKILGIQVCAYKDGKVIIDTAAGTLGKYDPRPVQPDSLFPVFSVTKGITAGMVHWLVDKGKLKYEETVA

            *:** **:* *: **         ***:******::*                 .*  : :*.*
At5g24810   NMWPGFGSNGKDTIK---------VHHVLNHTSGMQNSFDPVGENPLLICDWDECLKRIANSSPETEPGSQQSYHYLTFG
Os06g48770  NIWPKFGTNKKELIKTLICNFLLKVHHLLNHTSGLHN-----------------ALGDVMKSDP----------------

                  * :********:***:**:**:*:****************:**.* :*:.***.: : *::*. : .: : ***
At5g24810   WLCGGILEYASGKKLQEILEESIVKPLNIDGELYIGIPPGVESRLATLTFDTDEMSKLSSIASQPELPSTFQPDKIIQMA
Os06g48770  ------LLHASGKKLQEVLEEAIVHPLHINGELYIGIPPGVESRLAALTVDMEELEKLSGFRAGPDVPQELLSN-VAQMA

            *.*********:****:*****************:*. ** :*****.  *:******.*.***.:: ...*** *.:
At5g24810   TNLPVLFNTLNVRRAIIPAANGHCSARALARYYATLADGGLVPPPHSS-LSQPPLGSHTHVPKFTSLKDTTKKKKGKEMA
Os06g48770  TGLPVLFNTLNIRRAILPAANGHCSARALARYYAALGAGGAIPPPHSGGGSKPPLGSHVHTPKFPTMPSKKKKKGGSKND

            .   .*.   * *           :*. ..: .::: :*  *.*.. :.:  *          ...:: *****.**:**
At5g24810   ATEKGKSKDHQERKLYDEKQFMSASSSRESNTESLARLVDTNSSAGKTEINSDDHQHDIHNMFSNPRIHDAFMGAGDYSG
Os06g48770  VGVADKDGYTQLR-----------TSDGSDEGSTVSAVVAGNGSGSGSSMFVD----------GGTKMLDAFMGVGDFSG

            :: *:*******:*     *:      . *****:********::: :::***:****:**** .:*:***.
At5g24810   LVVPDGKFGLGFKRAISQDGS-----LVGFGHSGLGGSTGFCDINNRFSIAVTLNKMSMGGVTANIVKLVCS
Os06g48770  MIHPNGKFGLGFRRYGYGAGAGEKVATTTFGHSGMGGSTGFCDVEHGLAMAVTVNKMSLGGVTRRVVRLVCE
```
