# Supplementary material for: Cross genome comparisons of serine proteases in Arabidopsis and rice
Source: BMC Genomics. 2006 Aug 9;7:200. doi: 10.1186/1471-2164-7-200 (PMC1560137; doi:10.1186/1471-2164-7-200)
Supplement: Additional file 10 — Figure SF6. Multiple sequence alignment of Arabidopsis and rice serine Lon protease-like proteins. Multiple sequence alignment of the Lon_C protease domain region of the annotated Arabidopsis and rice Lon protease-like proteins. The catalytic dyad residues are indicated. Gene names correspond to those in Additional files 1 and 2. For brevity, rice gene names have been shortened to OsXXg##### instead of LOC_OsXXg#####, XX referring to chromosome 1–12 and a 5 digit number assigned to each gene. [file 1471-2164-7-200-S10.pdf]

```
           : . .   . :* .              .: :.:  .  *
 At5g47040 VVDETMLEKILGPPRFDDSEAADRVASAGVSVGLVWTTFGGEVQFVEATSMVG---KGEMHLTGQLGDVIKESAQLALTW
Os09g36300 IVDEAMLEKVLGPPRFDDSEAADRVASPGVSVGLVWTSFGGEVQFVEATAMVG---KGDLHLTGQLGDVIKESAQLALTW
 At3g05780 MIDESNLADYVGKPVFQEEKIYEQTP-VGVVMGLAWTSMGGSTLYIETTFVEEGLGKGGLHITGQLGDVMKESAQIAHTV
 At3g05790 MIDESNLSDYVGKPVFQEEKIYEQTP-VGVVMGLAWTSMGGSTLYIETTFVEEGEGKGGLHITGRLGDVMKESAEIAHTV
 At5g26860 MIDESNLADYVGKPVFHAEKLYEQTP-VGVVMGLAWTSMGGSTLYIETTVVEEGEGKGGLNITGQLGDVMKESAQIAHTV
Os03g19350 VVDASNLDNFVGKAVFQPERIYDQTP-VGVVMGLAWNAMGGSTLYIETAKVEDGEKKGALVVTGQLGDVMKESAQIAHTI
Os07g48960 VIDSSNLGDYVGKPVFQAERIYEQTP-VGVVMGLAWTAMGGSTLYIETTKVEEGDGKGALVMTGQLGDVMKESAQIAHTV
Os06g05820 VSGSPRNGEVVGIPRNR----------ADIIISVLMKQAG----------------------------------------

 At5g47040 VRARASDFKLALAGDMNVLDGRDIHIHFPAGAVPKDGPSAGVTLVTALVSLFSQKRVRADTAMTGEMTLRGLVLPVGGIK
Os09g36300 VRARAADLNLSPTSDINLLESRDIHIHFPAGAVPKDGPSAGVTLVTSLVSLFSHRKVRADTAMTGEMTLRGLVLPVGGVK
 At3g05780 ARR----IMFEKEPENLFFANSKLHLHVPEGATPKDGPSAGCTMITSFLSLAMKKLVRKDLAMTGEVTLTGRILPIGGVK
 At3g05790 ARR----IMLEKEPENKLFANSKLHLHVPAGATPKDGPSAGCTMITSLLSLALKKPVRKDLAMTGEVTLTGRILAIGGVK
 At5g26860 ARK----IMLEKEPENQFFANSKLHLHVPAGATPKDGPSAGCTMITSLLSLATKKPVRKDLAMTGEVTLTGRILPIGGVK
Os03g19350 CRS----ILHEKEPNNTFFTKSKLHLHVPAGATPKDGPSAGCTMVTSMLSLAMGKLVKKDLAMTGEVTLTGRILPIGGVK
Os07g48960 GRA----ILLDKEPENLFFANSKVHLHVPAGSTPKDGPSAGCTMITSMLSLAMGKPVKKDLAMTGEVTLTGRILPIGGVK
Os06g05820 ----------LKLQDNAVFLNVVSGFMLTETAG-------DLAIAASICSSFLEYPIPNDIAFIGEVGLGGELRTVPRMD

           .: :*  :   *  :.*  .     ::   :   *::   .   .: : .*
 At5g47040 DKILAAHRYGIKRVILPQRNSKDLVEVPAAVLSSLEVILAKRMEDVLENAFEG
Os09g36300 DKVLAAHRYGIKRVILPERNMKDLAEVPAPILSGLEILLVKRIEEVLDHAFEG
 At3g05780 EKTIAARRSQIKTIIFPEANRRDFEELAENMKEGLDVHFVDEYEKIFDLAFNY
 At3g05790 EKTIAARRSQVKVIIFPEANRRDFDELARNVKEGLEVHFVDEYEQIFELAFGY
 At5g26860 EKTIAARRSQIKTIIFPEANRRDFDELAENVKEGLNVHFVDDYGKIFELAFGY
Os03g19350 EKTIAARRSGIKTIIFPAANRRDFDELAPNVKEGLEVHFVDKYSEIYDLAFPS
Os07g48960 EKTIAARRSAVKTIVFPAANKRDFDELAPNVKEGLEVHFVDTYNEIFDIAFQS
Os06g05820 KRVLAIAKLGYKKCVVPKTS----EKLLRPLNLELEILPCSNLKEVINTVFRP
```
