# Supplementary figures and images for: Cross genome comparisons of serine proteases in Arabidopsis and rice
Source: BMC Genomics. 2006 Aug 9;7:200. doi: 10.1186/1471-2164-7-200 (PMC1560137; doi:10.1186/1471-2164-7-200)

Clade I

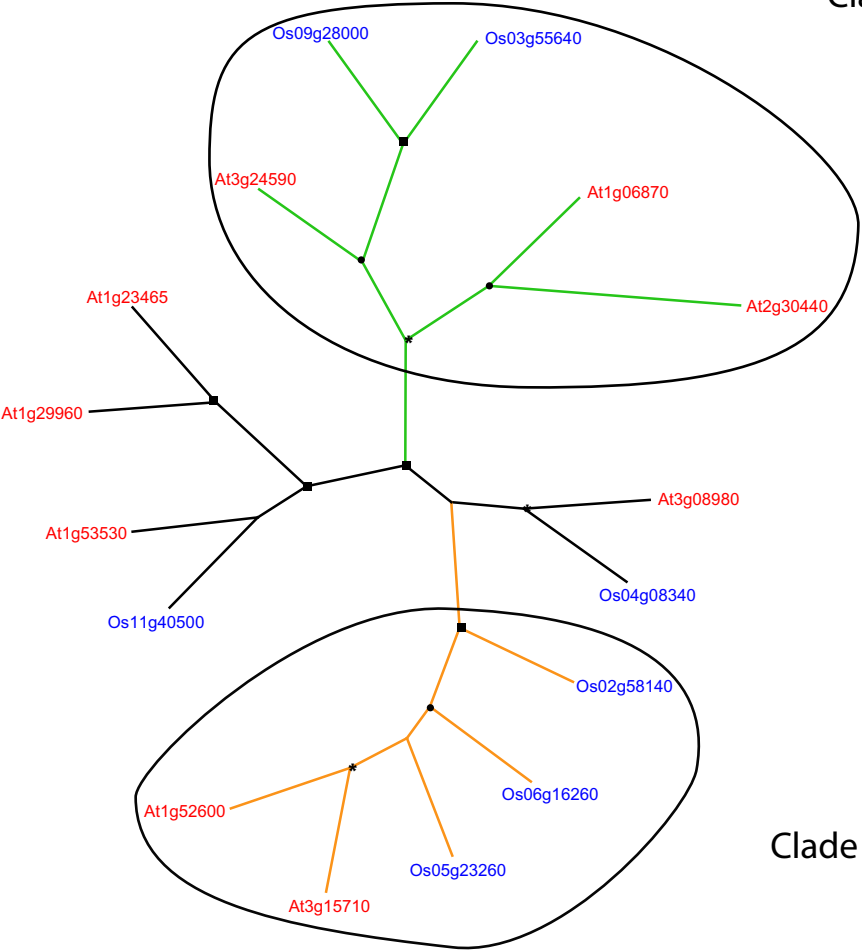

Clade II

Supplement: Additional file 11 — Figure SF7. Multiple sequence alignment of Arabidopsis and rice serine Type I SPase-like proteins. Unrooted N-J tree computed from multiple sequence alignments of Arabidopsis (red) and rice (blue) Type I SPase domains. Type I SPase domains were aligned using ClustalW[95] program and the alignments were exported to Phylip package[96] for representing the Neighbor-Joining tree (see methods). The colors represent the two evolutionary clades identified in the analysis (see text for details). Clade I is represented in green Clade II is shaded orange. For clarity, bootstrap values were replaced with symbols representing bootstrap percentages >50%. Bootstrap values between 50–60% are represented by an asterix, circles represent bootstrap values from 60%–80% while bootstrap values >80% are represented by rectangles. Gene names correspond to those listed in Tables 2 and 3. For brevity, rice gene names have been shortened to OsXXg##### instead of LOC_OsXXg#####, XX referring to chromosome 1–12 and a 5 digit number assigned to each gene. [file 1471-2164-7-200-S11.pdf]
