# Supplementary material for: Cross genome comparisons of serine proteases in Arabidopsis and rice
Source: BMC Genomics. 2006 Aug 9;7:200. doi: 10.1186/1471-2164-7-200 (PMC1560137; doi:10.1186/1471-2164-7-200)
Supplement: Additional file 12 — Figure SF8. Multiple sequence alignment of Arabidopsis and rice serine S28 protease-like proteins. Multiple sequence alignment of the family S28 protease domain region of the annotated Arabidopsis and rice family S28 protease-like proteins. The catalytic triad residues are indicated. Gene names correspond to those in Additional files 1 and 2. For brevity, rice gene names have been shortened to OsXXg##### instead of LOC_OsXXg#####, XX referring to chromosome 1–12 and a 5 digit number assigned to each gene. [file 1471-2164-7-200-S12.pdf]

```
                                 :               .*::     *.         . :
 At2g24280 QNLDHFSFTPDSYKVFHQKYLINNRFWRK--------GGPIFVYTGNEGDIDWFASNTGFMLDIAPKFRALLVFIEHRFY
Os06g43930 QELDHFTFTPNASAVFYQKYLVNDTFWRRSAAAGETPAGPIFVYTGNEGDIEWFATNTGFMFDIAPSFGALLVFIEHRFY
 At5g65760 QQLDHFSFAD--LPKFSQRYLINSDHWLG-----ASALGPIFLYCGNEGDIEWFATNSGFIWDIAPKFGALLVFPEHRYY
Os01g56150 QRLDHFSFLE--EEGEEGDGFFQQRYLVGRGGGWAGAGGPIFFYCGNEGDIAWFAANSGLVWEAATRFAALLVVFAEHRYY
Os11g05760 QRLDHFNELPASNGTFRQRYLVNGTFWGG-------AAAPVFVYAGNEGDVALFASNTGFMWEAAPRFRAMLVFVEHRYY
 At5g22860 QTLDHFTFTPESYMTFQQRYAIDSTHWGG-----AKANAPILAFLGEESSLDSDLAAIGFLRDNGPRLNALLVYIEHRYY
 At4g36190 QTLDHYSPSDHRKFRQRYYEYLDHLRVPD---------GPIFLMICGEGPCNGITN--NYISVLAKKFDAGIVSLEHRYY
 At4g36195 QTLDHYSPSDHREFKQRYYEYLDHLRVPD---------GPIFMMICGEGPCNGIPN--DYITVLAKKFDAGIVSLEHRYY
 At2g18080 ----------------------DYFRSPD---------GPMFMIICGEGPCSGIAN--DYINESLNKKLN----------
Os10g36760 -------SADHRQFKQRYYEFLDYYRAPK---------GPIFLYICGESSCNGIPN--SYLAVMAKKFGAAVVSPEHRYY
Os10g36780 QRLDHFSPTDHRQFKQRYYEFADYHAGG----------GPVFLRICGESSCNGIPN--DYLAVLSKKFGAAVVTPEHRYY

                                                      :     . ..:* **.* *::*:*:*:**:. *::**
 At2g24280 GESTPFG---KKSHKSAETLGYLNSQQALADYAILIRSLKQNLS-SEASPVVVFGGSYGGMLAAWFRLKYPHITIGALAS
Os06g43930 GESKPFG---NESNSSPEKLGYLTSTQALADFAVLITSLKHNLS-AVSSPVVVFGGSYGGMLASWFRLKYPHVTIGAVAS
 At5g65760 GESMPYGSR-EEAYKNATTLSYLTTEQALADFAVFVTDLKRNLS-AEACPVVLFGGSYGGMLAAWMRLKYPHIAIGALAS
Os01g56150 GESMPFGSK-DKAYNNSKSLAYLTAEQALADYAVLLTDLKKNLS-SEGSPVVLFGGSYGGMLAAWMRLKYPHIAVGALAS
Os11g05760 GESLPFGGTRAAAFADASAAGYLTTAQALADFAELILSLKSNLT-ACKAPVVIFGGSYGGMLAAWMRMKYPHIVMGAVAS
 At5g22860 GETMPFG-SAEEALKNASTLGYLNAAQALADYAAILLHVKEKYS-TNHSPIIVIGGSYGGMLAAWFRLKYPHIALGALAS
 At4g36190 GKSSPFKSLATKNLKYLSSKQALSDLATFRQYYQDSLNVKFNRSSNVENPWFFFGVSYSGALSAWFRLKFPHLTCGSLAS
 At4g36195 GKSSPFKSLATENLKYLSSKQALFDLAAFRQYYQDSLNVKFNRSGDVENPWFFFGASYSGALSAWFRLKFPHLTCGSLAS
 At2g18080 -----------------------------------------ISSGGSDNPWFFFGISYSGALSAWFRLKFPHLTCGSLAS
Os10g36760 GKSSPFESLTTENLRFLSSKQALFDLAVFRQYYQETLNAKYNRS-GADSSWFVFGGSYAGALSAWFRLKFPHLTCGSLAS
Os10g36780 GKSSPFESLTTENLRFLSSKQALFDLVAFRQHYQEILNARYNRSSGFDNPWFVFGVSYSGALSAWFRLKFPHLTCGSLAS

           *.             :     :  .  .  *  .::     :              : :  .       .
 At2g24280 SAPILHFDNIVPLTSFYDAISQDFKDASINCFKVIKRSWEELEAVSTMKNGLQELSKKFRTCKGLHSQYSARDWLSGAFV
Os06g43930 SAPILQFDYITPWSSFYEAVSQDYKSESFNCFSVIKAAWDLIDERGSTDAGLLQLSKTFRACKTVKSVYSFRNWLWTAFV
 At5g65760 SAPILQFEDVVPPETFYDIASNDFKRESSSCFNTIKDSWDAIIAEGQKENGLLQLTKTFHFCRVLNSTDDLSDWLDSAYS
Os01g56150 SAPILQFEDVVPSTIFYDLVSNDFKRESLSCFQTIKDSWKALDAQGNGQDGLLKLSKTFHLCKTIKNTGELSDWLSSAYS
Os11g05760 SAPILGLNGLSDPYSFYNVVSNDFKSESKHCYDVLRNSWSEMYKALATDAGRARLNQTFNMCKGN--VDDIPGLVEKALI
 At5g22860 SAPLLYFEDTRPKFGYYYIVTKVFKEASERCYNTIRNSWIEIDRVAGKPNGLSILSKQFKTCAPLNGSFDIKDFLDTIYA
 At4g36190 SA------VVRAVYEFPEFDQQIAESAGPECETALQETNKLLELGLKVNN---RAVKALFNATELDVDADFLYLIADAGV
 At4g36195 SA------VVRAVYEFPEFDQQIGESAGPECKAALQETNKLLELGLKVNN---RAVKALFNATELDVDADFLYLIADAEV
 At2g18080 SA------VVRAIYEFSEFDQQIGESAGQECKLALQETNKLLELGLKVKN---KAVKSLFNATELDVDADFLYLTADAAV
Os10g36760 SG------VVLSVYNYTDFDKQIGESAGPECKAALQETTKLVDGQLQSGR---NAVKQLFGASTLANDGDFLFLLADAAA
Os10g36780 SG------VVLAVYNFTDFDKQVGDSAGPECKAALQEVTRLVDEQLRLDS---RSVKVLFGAEKLKNDGDFLFFLADAAA

                .           *     :    :         .             .                          *
 At2g24280 YTAMVNYPTAANFMAPLPGYPVEQMCKIIDGFPRGS--SNLDRAFAAASLYYNYSGSE--KCFE---MEQQTDDHGLDGW
Os06g43930 YTAMVDYPTPANFLMNLPAYPIKEMCKIIHGFPAGA--DIVDKAFAAASLYYNYTGDQ--TCFQ---LEDGEDPHGLSGW
 At5g65760 YLAMVDYPYPADFMMPLPGHPIREVCRKIDGAGSNA--SILDRIYAGISVYYNYTGNV--DCFK---LDD--DPHGLDGW
Os01g56150 YLAMVDYPMPADFMMPLPGNPIKELCTKIDNQPDGT--SILERIYAGVNVYYNYTGTV--DCFD---LND--DPHGMDGW
Os11g05760 YGSMMDYPTPSNFLTSLPAYPVREICRAIDKPTSGN--DTVSRIKDAMTIYYNSTGGL--ACFPGAGAEDDDPYGMFPGW
 At5g22860 EAVQYNR---------GPNFWVAKVCNAINANPPNRRYNLLDRIFAGVVALVGNRTCYDT-------KMFAQPTNNNIAW
 At4g36190 MAIQYGNPDK----LCVPLVEAQKNGGDLVEAYAKYVREFCMGVFGQSSKTYSRKHLLDTAVT---------LESADRLW
 At4g36195 MAIQYGNPDK----LCVPLVEAQKNRDDLVEAYAKYVREFCVGVFGLSSKTYSRKHLLDTAVT---------PESADRLW
 At2g18080 MAFQYGNPDK----LCVPLVEAKKNGSDLVVTYSTYVREYCMRIWGLRVRTYNRKHLRNTVVT---------ADSAYRLW
Os10g36760 IAFQYGNPDA----LCSPIVEAKKNGTDLVETFARYVKDYYIGTFGASVASYDQEYLKNTTPPP--------AESAYRLW
Os10g36780 IGFQYGSPDA----VCSPLINAKKTGRSLVETYAQYVQDFFIRRWGTTVSSYDQEYLKNTTP----------DDTSSRLW

            :* *:*:         :                       * *        :*             .  .::*:* **
 At2g24280 QYQACTEMVMPMSCS--NQSMLPPYENDSEAFQEQCMTRYGVKPRPHWITTEFGGMR-----IETVLKRFGSNIIFSNGM
Os06g43930 GWQACTEMVMPMTIS--NESMFPPFTFTYEGKSDDCFQSYGVRPRPHWITTEYGGNR-----IDLVLKRFGSNIIFSNGM
 At5g65760 NWQACTEMVMPMSSNQ-ENSMFPGYGFNYSSYKEECWNTFRVNPRPKWVTTEFGGHD-----IATTLKSFGSNIIFSNGL
Os01g56150 DWQACTEMVMPMSYS--EDSMFPADKFNYTSYEKDCINSFGVEPRPQWITTEFGGHN-----ISLVLERFGSNIIFFNGL
Os11g05760 TWQACTEVIMTMSYGIGNATVFPPDPFNLTAYLAGCLATTGVPPRPHWIQSYFGGYD-----IRNVLKRSGSNIIFFNGL
 At5g22860 RWQSCSEIVMPVGYDK-QDTMFPTAPFNMTSYIDGCKSYHGVTPRPHWITTYFGIQE-----VKLILQKFGSNIIFSNGL
 At4g36190 WFQVCTEVAYFQVAPANDSIRSHQINTEYHLDLCKSLFGKGVYPEVDATNLYYGSDKIAVSSHLLILNFVATKIIFTNGS
 At4g36195 WFQVCTEVAYFQVAPANDSIRSHQINTEYHLDLCKSLFGKGVYPEVDATNLYYGSD-----------RIAATKIIFTNGS
 At2g18080 WFQACTELGYFQVAPKYDSVRSHQINTTFHLDLCKSLFGKDVYPKVDATNLYYGGD-----------RLAATKIIFTNGS
Os10g36760 WYQVCSEVAYFQVAPKNDSVRSAKIDTRYHLDLCRNVFGEGVYPDVFMTNLYYGGT-----------RIAGSKIVFANGS
Os10g36780 WFQVCSEVAYFQVAPKNDSIRSTEINTGYHLDLCRNVFGEGVYPDVFMTNLYYGGT-----------RIAASKIVFTNGS

            **:  ..  :  :  : :        .*  *:
 At2g24280 QDPWSRGGVLKNISSSIVALVTKKG--AHHADLRA------------ATKDDPEWLKEQRRQE--
Os06g43930 RDPWSRGGVLKNISSSIIALVTEKG--AHHLDFRS------------ATKDDPDWVVEQRRQE--
 At5g65760 LDPWSGGSVLKNLSDTIVALVTKEG--AHHLDLRP------------STPEDPKWLVDQREAE--
Os01g56150 LDPWSGGGVLKNISESVVAIIAPLG--AHHIDLRP------------ASKDDPDWLVRLRESE--
Os11g05760 RDPWSAGGILKSISNSIIALVEPKG--GHHVDLRF------------STKEDPEWLKKVRRQE--
 At5g22860 SDPYSVGGVLEDISDTLVAITTKNG--SHCLDITL------------KSKEDPEWLVIQREKE--
 At4g36190 QDPWRHAS-KQTSSPDLPSYIMTCHNCGHGSDLRGCPQS---------------------AMV--
 At4g36195 QDPWRHAS-KQTSSPELPSYIVTCHNCGHGSDLRGCPQSPMVIGGDSKNCSSPDAVNKVRQHI--
 At2g18080 EDPWRHAS-KQNSTHEMPSYIIKCRNCGHGSDIRGCPQSPMVIEGKSNN----------------
Os10g36760 QDPWRHAS-KQKSSKELPSYLIECSNCGHCSDLSGCPQAPSHIEGDSSNCSSPEAVNKVRKQI--
Os10g36780 QDPWRHAS-KQKSSKYMPSYIIKCRNCGHGTDLRGCPQLPFRIEGDPSNCSSPAAVSTVRKQI--
```
