# Supplementary material for: Cross genome comparisons of serine proteases in Arabidopsis and rice
Source: BMC Genomics. 2006 Aug 9;7:200. doi: 10.1186/1471-2164-7-200 (PMC1560137; doi:10.1186/1471-2164-7-200)
Supplement: Additional file 14 — Figure SF10. Multiple sequence alignment of Arabidopsis and rice protease IV-like proteins. Multiple sequence alignment of the protease IV domain region of the annotated Arabidopsis and rice protease IV-like proteins. The active site serine residue is indicated. Gene names are derived from those in Additional files 1 and 2 as follows: A1a73990 (At1g73390 N-terminal domain); A1b73990 (At1g73390 C-terminal domain); O2a49570 (Os02g49570 N-terminal domain); O2b49570 (Os02g49570 C-terminal domain). For brevity, rice gene names have been shortened to OsXXg##### instead of LOC_OsXXg#####, XX referring to chromosome 1–12 and a 5 digit number assigned to each gene. [file 1471-2164-7-200-S14.pdf]

```
           : .:  .:..   :..   **:. *.  : *   :     *:..    *  : *::.:: :    *:* .      *
A1a73990 -NFKKSGKFIVGYISICGLKEYYLGCACNELFAPPSAYSFLYGLTVQASFLGGVFEKVGIEPQVQRIGKYKSAGDQLSRK
O2b49570 -DFKKSGKFVVGYMPVCGEKEYYLACACGELYAPPSAYVALFGLTVQQTFLRGVLEKVGIEPEIQRIGRYKSAGDQLARK
A1b73990 KLLAETKPVIASMSDVAASGGYYMAMAANAIVAENLTLTGSIGVVTARFTLAKLYEKIGFNKETISRGKYAELLGAEERP
O2a49570 RLLADTKPVVASMSDVAASGGYYMAMAAPVIVAEKLTLTGSIGVVTGKFILQKLYERIDFNKEIISKGRYAELNAADQRP

           .:*  *::    :: *  :  *  :   .   :.:* . :  *:      . * :  :   ...::: *::
A1a73990 SISEENYEMLSVLLDNIYSNWLDGVSDATGKKREDVENFINQGVYEIEKLKEAGLIKDIRYDDEVITMLKERLGVE
O2b49570 SMSNEVREMLATLLDNIYGNWLDTISSKHGKKKEEIEEFINSGVYQVARLKEEGWITDLLYDDEVMAMLKERVAQK
A1b73990 LKPEE-AELFEKSAQHAYQLFRDKAALSRSMPVDKMEEVAQGRVWTGKDAHSRGLIDAVGGLSRAIAIAKQKANIP
O2a49570 LRPDE-AELFEKSAQNAYALFRDKAAMSRSMNVDQMETVAQGRVWSGQDAASRGLVDSLGGFSQALAIAKQKANIP
```
