# Supplementary material for: Cross genome comparisons of serine proteases in Arabidopsis and rice
Source: BMC Genomics. 2006 Aug 9;7:200. doi: 10.1186/1471-2164-7-200 (PMC1560137; doi:10.1186/1471-2164-7-200)
Supplement: Additional file 15 — Figure SF11. Multiple sequence alignment of Arabidopsis and rice rhomboid-like proteins. Multiple sequence alignment of the rhomboid protease domain region of the annotated Arabidopsis and rice rhomboid protease-like proteins. The catalytic triad residues are indicated. Gene names correspond to those in Additional files 1 and 2. For brevity, rice gene names have been shortened to OsXXg##### instead of LOC_OsXXg#####, XX referring to chromosome 1–12 and a 5 digit number assigned to each gene. Several sequences identified here display mutations in key catalytic residues and may possibly have evolved a regulatory function (See text for details). [file 1471-2164-7-200-S15.pdf]

```
                                                                *
At1g18600 -----FKSGRLHTLITSAFSHIDIGHIVSNMIGLYFFGTSIAR-NFGPQFLLKLYLAGALGGSVFYLIHHAYMAATSPKG
Os01g55740 -----FKSGRLHTLLTNAFSHAESGHLISNMIGLYFFGSSISN-MFGPAFLLKLYVAGALAGSAFFLLEKAFLAPRR---
At1g74130 -----FMTGRIHTLITSGFSHVGATHIILNMMGLCYFGARIAR-SFGPRYLLKLYFAGALGGSVFFLSSHALSVISL-KG
At1g74140 GFQKRGWKSWINGANGVVFGLVIANAAVFTMWRVSDRSWMIAR-TLGPLYLLKLYFAGALGGSVCFLSYHALLATLK-GE
At1g12750 -----VQGNEKWRLITAMWLHAGIIHLVMNMFDVIIFGIRLEQ-QFGFIRIGLIYLISGFGG----SILSALFLQK----
At1g63120 -----VHEHQGWRLLSCMWLHAGIIHLLTNMLSLIFIGIRLEQ-QFGFIRVGLIYLISGLGG----SILSSLFLQE----
Os03g02530 -----VHEHQGYRLITSIWLHAGVLHLVANMLSLIFIGLRLEQ-QFGYVRIGAIYLLSGLGG----SVLSSLFIRN----
Os10g37760 -----VHEHQGWRLVTCIWLHAGVVHLLANMLSLVLIGLRLEQ-QFGYMRIGAIYLLSGLGG----SVLSSLFIRN----
At5g07250 -----VEKKEGWRLLTCIWLHAGVIHLGANMLSLVFIGIRLEQ-QFGFVRIGVIYLLSGIGG----SVLSSLFIRN----
Os08g43320 -----VHQHQAWRLISCIWLHAGLIHLIVNMLSLLFIGLRLEQ-QFGFVRIGIIYLLSGFGG----SVLSVLFLRNN---
Os09g35730 -----VHQHQGWRLISCIWLHAGLIHLVVNMLSLLFIGIRLEQ-QFGFVRIGAIYLLSGFGG----SVLSALFLRNN---
Os04g48130 -----VHGNQAWRLETSTWLHAGLIHLAANMISLLIIGIRLEQ-QFGFWKVGLVYLVSGFGG----SVLSVLFISRN---
At3g53780 -----VKGDEGWRLLSCNWLHGGVVHLLMNMLTLLFIGIRMER-EFGFIRIGLLYLISGFGG----SILSALFLRS----
At4g23070 -----VHKRQVWRLLTCMWLHAGVIHLLANMCCVAYIGVRLEQ-QFGFVRVGTIYLVSGFCG----SILSCLFLED----
At2g29050 -----VHKHEVWRLFTCIWLHAGVFHVLANMLSLIFIGIRLEQ-EFGFVRIGLLYMISGFGG----SLLSSLFNRA----
Os11g47840 -----VHGHQGWRLITCIWLHAGVVHLLINMLCLLFIGIRLEQ-EFGFVRIGLVYLISGLGG----SLMSALFIRS----
At1g52580 -----VEEGERWRLISCIWLHGGFLHLMANMISLMCIGMRLEQ-EFGFMRIGALYVISGLGG----SLVSCLTDSQG---
Os01g05430 -----TNDHEGWRLITCIWLHAGVVHILANMLSLLLIGIRLEK-EFGFMRIGTLYVISGVGG----SLLSALFMVSN---
At1g77860 -----TENHEIWRILTSPWLHSGLFHLFINLGSLIFVGIYMEQ-QFGPLRIAVIYFLSGIMG----SLFAVLFVRN----
At1g25290 -----IERGQLWRLATASVLHANPMHLMINCYSLNSIGPTAES-LGGPKRFLAVYLTSAVAKPILRVLGSAMSYWFN---
Os09g28100 -----IDRGELWRLATSSLLHANLAHLAFNCFSLNSIGPMVEM-LTGPRRYLAVYFTSALAG-------SLMSYRYC---
At5g25752 -----HNFPAWYQFVTATFCHANWNHLSSNLFFLYIFGKLVEE-EEGNFGLWLSYLFTGVGAN----LVSWLVLPRN---
Os05g13370 ----YHAFPSWFQFVTSTFCHANWNHLSSNLFFVYIFGKLVEE-EEGSFALWMSYILTGAGSN----LISWLVLPTS---
At3g17611 -----FKHKDLKRLFLSAFYHVNEPHLVYNMMSLLWKGIKLET-SMGSSEFASMVFTLIGMSQGVTLLLAKSL-LLLFDY
Os01g18100 -----IHYCDLTRFFLSAFYHLSETHFFFNMSSLLWKGIQLET-SMGSVEFASMVAALLGMSQGITLLLSKG--LLLFGN
At3g58460 -----ISRFQVYRFYTAIIFHGSLLHVLFNMMALVPMGSELER-IMGSVRLLYLTVLLATTNAVLHLLIASLAGYNPFYQ
Os03g44830 -----ASRFQVYRFYTSVVFHGSLLHVLFNMLALVPLGTELER-IMGSVRLLFLMFLLATTNAIFHLIIAFLAAYNPLYP
At3g59520 -----AIEGHYWRMITSALSHISVLHLVFNMSALWSLGVVEQLGHVGLGTAYYLHYTLVLVVFSGVLVIGIYHLLIARFK
Os01g67040 -----MEGGQYWRIITSAFSHISVVHLVFNMSALWSLGVVEQLGQIGLGVEYYLHYTLVLVVLSGLLVLGFYHVMIQKFK
Os03g24390 -----RTIPFAWNLVTSGYIEQTIPGVIVSIVGLLVLGKVLEP-LWGAKELLKFIFLVNLSTSACVFVTAIILYYITQ--
Os07g46170 -----RTIPFAWNLITGGYVELTIPGVIISIVGLLLFGKLLEP-LWGSKELSKFIFVVNFSTSLCVFITAIALYYTTQ--
At3g07950 -----RTIPFAWNLITSGYFELSVYGVVFSTVSLLFMGKFLEP-VWGSTEFLKFIFVVNFLTYLCVFVTAIALYYITR--
Os04g01300 -----KTIPFVWTVFTAGYIEQVLPGAIGSSLGLLFCGKDIEP-VWGRKEFLKFIILVNSICGVLAFCFAVALYYVTG--
At2g41160 -----FEKFRIWKLIISAFAFSSTTQLLSGLYLLLYFFRVFERQ--IGSNKYSVFIFFSGFVSLILETILLSLTKDP---
At3g56740 -----FEKFRIWKLIMSTFAFSSTPELMFGLYLLLYYFRVFERQ--IGSNKYSVFILFSGTVSLLLEVILLSLLKDT----
Os01g16330 -----VKNFRLWKLFASGFAFQSTPELLFGVYLLLYYFRVFERQ--IGSNKYSVFSLFTISVSLLLEILSLVLLKDTN---
At5g38510 -----ILAGEWWRLVTPMFLHSGIPHVALSSWALLTFGPKVCR-DYGLFTFCLIYILGGVSG----NFMSFLHTADP---
At2g39060 -----ISINTFGCFIEISYLFLYILYAPREAKISTLKLIVICN--IGGLGLLILLVNLLVPKQHRVSTV----------

                      .
At1g18600 QGAFVRDPSRTP-G--LGASGAVNAIMLLDIFLHPRATLYLEFFIPVPAMLLGIFLIGKDILRITEGNSNISG-------
Os01g55740 QFYGGWDNSRTPA---LGASAAANAIILLDIFLYPKKLVYLYFFIPIPAAIMGAILIGADLLRVKNVLAF----------
At1g74130 QRVVPKDQLKVPIGK-LGANGPVYAITLLDMLLYPKVTTYFGLMLRVPVF-AGIYSLGLNIIKMLEGKNNNTLTSLD---
At1g74140 GVVIKDHQSTAPISQLLGADGSMFAIALLDMFIYPKVTTYFALMLRVHR----IINLGVEILNIPEGGPNHIASS-----
At1g12750 -------------SISVGASGALLGLMGAMLSELLTNWTIYKSKLCALLSFLFIIAINLAIGLLPW------------VD
At1g63120 -------------SISVGASGALFGLLGAMLSELLTNWTIYANKAAALITLLFIIAINLALGMLPR------------VD
Os03g02530 -------------HISVGASGALFGLLGAMLSELLTNWTIYTNKVAAVITLLFVIAVNLALGILPH------------VN
Os10g37760 -------------SISVGASGALFGLLGAMLSELFTNWTIYTNKAAALVTLLIVIAINLAIGILPH------------VD
At5g07250 -------------SISVGASGALFGLLGSMLSELFTNWTIYSNKIAALLTLLFVILINLAIGILPH------------VD
Os08g43320 -------------YISVGASGALFGLLGSMLSELIMNWTIYSNKAAAIITLLFIIAINLAIGILPH------------AD
Os09g35730 -------------YISVGASGALFGLLGSMLSELIMNWTIYSNKAAAIITLLFIIAINLAIGILPH------------AD
Os04g48130 -------------GITVGASGALFGLLGAMLSELITNWTIYSNRCAAMVNLIIIAAINLALGILPR------------VD
At3g53780 -------------NISVGASGAVFGLLGGMLSEIFINWTIYSNKVVTIVTLVLIVAVNLGLGVLPG------------VD
At4g23070 -------------AISVGASSALFGLLGAMLSELLINWTTYDNKGVAIVMLLVIVGVNLGLGTLPP------------VD
At2g29050 -------------GISVGASGALFGLLGAMLSELLTNWTIYANKFAALLTLIFIIAINLAVGILPH------------VD
Os11g47840 -------------SISVGASGALFGLIGSMLSELITNWSLYANKVAALLTLVFVIVVNLALGILPR------------VD
At1g52580 ------------ERVSVGASGALFGLLGAMLSELITNWTIYENKCTALMTLILIIVLNLSVGFLPR------------VD
Os01g05430 --------------ISVGASGALFGLLGSMLSELITNWTIYENKVHHWIHIGTERVQCQRALFLVP------------LP
At1g77860 -------------IPSISSGAAFFGLIGAMLSALAKNWNLYNSKISALAIIFTIFTVNFLIGFLPF------------ID
At1g25290 ------------KAPSVGASGAIFGLVGSVAVFVIRHKQMVRGGNEDLMQIAQIIALNMAMGLMSRR-----------ID
Os09g28100 ------------ASPAVGASGAIFGLVGAYAVYTWRHRRFLGHGKESLEHIGRVVILNMGMGLLTRG-----------ID
At5g25752 -------------AVSVGASGAVFGLFAISVLVKMSWDWRKILEVLILGQFVIERVMEAAQASAGLSGTIYGGYSLQTVN
Os05g13370 -------------SVSLGASGAVFGLFTISVLVKMSWDWRKILEVLILGQFVVDKVMEAARATTVTG-------HSLQVN
At3g17611 DRAYYN-------EYAVGFSGVLFAMKVVLNSQA--EDYSSVYGILVPTKYAAWAELILVQMFVPN------------AS
Os01g18100 DEAYYDQ-------YAVGFSGVLFGMKVVLNAWS--DDYVFLHGVVIPAKYAAWAELLIQAFIPG------------TS
At3g58460 YDHLMNE-------CAIGFSGILFSMIVIETSLSGVTSRSVFGLFNVPAKLYPWILLIVFQLLMTN------------VS
Os03g44830 LHFLVDE-------CSIGFSGVIFSMIVIETSLSGVQTRSVFGLFNVPANWYAWILLVLFQFLASN------------VS
At3g59520 IDYFRRV-------TAVGYSCVVFGWMTILSVKQPSSKLNLFGLLSLPISFAPFESLIFTSIIVPQ------------AS
Os01g67040 VEYFRRV-------TAVGYSCVVFGWMTILATKQPSSKLNIFGVLSLPISFAPFESLIFTSIMVPQ------------AS
Os03g24390 QEIYLYT-------PLSGFYGVLSGFLVGIKQILPDQEI-TLFLLNIKAKWIPSLVAFISVSLSFFM-----------KD
Os07g46170 EESYLYA-------PLSGFYGVLTGLLVGIKQLMSEQEL-NLFVLKIKAKWIPSLVALISVIVSFFV-----------KE
At3g07950 LEVYLYMPFAGFHGVLAGLLVGIKQIIPDQEILLLKIKAKWLPSIMLILSIASSFFTLDSAAYLPT-------------
Os04g01300 KESFLVTPLSGFHGALAGFLVGLKQLLPNLELPMCFFWKIKAKWMPFFVLCFSTIMAFIV------------------
At2g41160 ----------TANLLTSGFYALVFASFVPFFLDIPVTKRFGVLGVHFSDKSFIYLAGVQLLLSSWKRSIFT---------
At3g56740 ----------TANLLTSGFYGLIFASFIPFYLDIPVSTRFRVFGVNFSDKSFIYLAGVQLLLSSWKRSIFP---------
Os01g16330 ----------YLSTLACGFYGLIFASFIPFFLDIPVTSRFRIFGVNFSDKSFIYLAGLQLLLSSGKRSLIP---------
At5g38510 ------------TVGGTGFAFALIGAWLVDQNQNKEMIKSNEYEDLFQKAIIMTGFGLILSHFGPIDDWT----------
At2g39060 ---------------GWVCAAYSLAVFASPLSVMRKVIKTKSVEYMPFLLSLSLTLNAVMWFFYGLLIKDKFIAMP--
```

```
 At1g18600 -SAHLGGAAVAAIAWARIRKGRF-------------
Os01g55740 ---------------------PG-------------
 At1g74130 ---QLGGVVVAVIAWARIRKGRF-------------
 At1g74140 -SGQLGGVVVAAMAWARIKKGRF-------------
 At1g12750 NFAHIGGLLTGFCLGFILLMQPQ-------------
 At1g63120 NFAHIGGFLTGFCLGFVLLVRPQ-------------
Os03g02530 NFAHIGGFLTGFLLGFVLLMRPH-------------
Os10g37760 NFAHIGGFLTGFLLGFIFLMRPH-------------
 At5g07250 NFAHVGGFVTGFLLGFILLARPQ-------------
Os08g43320 NFAHIGGFVTGFLLGFVLLARPQ-------------
Os09g35730 NFAHIGGFVTGFLLGFVLLARPQ-------------
Os04g48130 NFAHIGGFATGFLLGFVLLIQPQ-------------
 At3g53780 NFAHIGGFATGFLLGFVLLIRPH-------------
 At4g23070 NFAHIGGFFGGFLLGFLLLIHPQ-------------
 At2g29050 NFAHLGGFTSGFLLGFVFLIRPQ-------------
Os11g47840 NFAHIGGLISGFLLGFVMFIRPQ-------------
 At1g52580 NSAHFGGFLAGFFLGFVLLLRPQ-------------
Os01g05430 ELCTYFKVELQHAKQLLHVNDECLYFDDKYFS----
 At1g77860 NFANIGGFISGFLLGFVLLFKPQ-------------
 At1g25290 NWGHIGGLLGGTAMTWLLGPQWK-------------
Os09g28100 NWGHLGGLLGGMAMAWFLGPAWQ-------------
 At5g25752 HIAHLSGALVGVVLVWLLSKFPS-------------
Os05g13370 NIAHLSGALIGAALVFLINRIPF-------------
 At3g17611 FLGHLGGILAGIIYLKLKGSYSG-------------
Os01g18100 LIGHLGGILAGLAYLWLKRSFSG-------------
 At3g58460 LLGHLCGILSGFSYSYGLFNFLM-------------
Os03g44830 LLGHLCGILSGFAYTYGLFNYLL-------------
 At3g59520 FLGHLSGILVGYAISWGLIGGMN-------------
Os01g67040 FIGHLSGIIVGYSIAWGLVHGMN-------------
Os03g24390 SVSYIPIILFGIYLSWIYLRYFQ-------------
Os07g46170 LVSYLPVILFGIYMSWIYLRYFQ-------------
 At3g07950 ---LIFGTYMGWLYLRYLQRRPE-------------
Os04g01300 PDSINFLPTLLSGMYVSWIYLRYFQ-----------
 At2g41160 --GICGIIAGSLYRLNIFGIRK--------------
 At3g56740 --GICGIIAGSLYRLNILGIRK--------------
Os01g16330 --GICGLIAGSLYRLNVLGIRR--------------
 At5g38510 ---NLGALIAGIVYGFFTCPVLQ-------------
 At2g39060 ---NILGFLFGVAQMILYMMYQG-------------
```
