# Supplementary material for: Cross genome comparisons of serine proteases in Arabidopsis and rice
Source: BMC Genomics. 2006 Aug 9;7:200. doi: 10.1186/1471-2164-7-200 (PMC1560137; doi:10.1186/1471-2164-7-200)
Supplement: Additional file 16 — Figure SF12. Multiple sequence alignment of Arabidopsis and rice nucleoporin autopeptidase-like proteins. Multiple sequence alignment of the nucleoporin autopeptidase domain region of the annotated Arabidopsis and rice nucleoporin autopeptidase-like proteins. The catalytic residues are indicated. Gene names correspond to those in Additional files 1 and 2. For brevity, rice gene names have been shortened to OsXXg##### instead of LOC_OsXXg#####, XX referring to chromosome 1–12 and a 5 digit number assigned to each gene. [file 1471-2164-7-200-S16.pdf]

```
           .     * *   :*: :* ::**. :*  . .** ** **.:** .** *:* *:*:*  :**: :*:*  :
 At1g10390 --VYEHGADIEALMPKLRQSDYFTEPRIQELAAKERADPGYCRRVRDFVVGRHGYGSIKFMGETDVRRLDLESLVQFNTR
 At1g59660 SHSSPSGADIESLMPKLHHSEYFTEPRIQELAAKERVEQGYCKRVKDFVVGRHGYGSIKFLGETDVCRLDLEMVVQFKNR
Os12g06870 --RHGNGTSVERLAPKLVHADYYTEPSLEELAAKERAEPGYCSRVRDFAVGRHDYGSIKFIGETNVRGLDLESIVEFNNR
Os12g06890 --RHGNGTSVERLVPKLVHADYYTEPSLEELAAKERAEPGYCSRVRDFAVGRHDYGSIKFIGETDVRGLDLESIVEFNNR
 At1g80680 --LCEHSKEIIDSLPMLNSPDYFLKPCINELVEREIESPDYCSRVPDFTIGRIGYGYIRFLGNTDVRRLDLDHIVKFHRH
Os03g07580 --------SSDPVFPVLRHGDYFTKPSIDELVEREAADPGYCSRVPDFVVGRVGYGRIHFPGDTDVRGMDLNGIVKFGRH

           .* ** *::.** :*:****.* ***:                         *  .:  *** *:*::  .* *.* * *
 At1g10390 EVIVYMDESKKPAVGQGLNKPAEVTLLNIKCIDKKTGKQFTEGERVEKYKMMLKKKAEAQGAEFVSFDPVKGEWKFRVEH
 At1g59660 EVNVYMDESKKPPVGQGLNKPAVVTLLNIKCMDKKTGTQVMEGERLDKYKEMLKRKAGEQGAQFVSYDPVNGEWTFKVEH
Os12g06870 EVIVYKDDSKKPPVGEGLNKAAVVTLLNIKCMNKKTGEQYTEGPRVGKYKEILVKKAEEQGAEFISFDAAKGEWKFRVKH
Os12g06890 EVIVYKDDSKKPPVGEGLNKAAVVTLLNIKCMNKKTGDQYTEGPRVDKYKEMLVKKAEEQGAEFISFDAVNGEWKFRVKH
 At1g80680 EVIVYDDESSKPVVGEGLNKAAEVTLVVN------IPDLTWGKQQVNHIAYKLKQSTERQGATFISFDPDNGLWKFFVPH
Os03g07580 SVEVYKDEASKPPLGQGLNKPAEVTLMLN------LSVLPEP----SALGELLKCQTRKQGARFVSFNHSSGRWKFEVDH

           ** : : :
 At1g10390 FSSYKLGDEDEEDGV-----
 At1g59660 FSSYKLGDEYD---------
Os12g06870 FSSYGFGEAEIDS-------
Os12g06890 FSSYGFGEAEIVS-------
 At1g80680 FSRFGLSDDEAEDIAMDDAP
Os03g07580 FSRFGLVDEEEEDVVMDEVV
```
