# Supplementary material for: Cross genome comparisons of serine proteases in Arabidopsis and rice
Source: BMC Genomics. 2006 Aug 9;7:200. doi: 10.1186/1471-2164-7-200 (PMC1560137; doi:10.1186/1471-2164-7-200)
Supplement: Additional file 13 — Figure SF9. Multiple sequence alignment of Arabidopsis and rice Ctpase-like proteins. Multiple sequence alignment of the Ctpase domain region of the annotated Arabidopsis and rice Ctpase-like proteins. The catalytic dyad residues are indicated. Gene names correspond to those in Additional files 1 and 2. For brevity, rice gene names have been shortened to OsXXg##### instead of LOC_OsXXg#####, XX referring to chromosome 1–12 and a 5 digit number assigned to each gene. [file 1471-2164-7-200-S13.pdf]

```
             **:::  *.  *   :  *:. :  .... :::***:* ** .  *:: *::::: .               :    .
At3g57680  KTGYVKLTAFSQTAASDMENAVHEMENQDVQSYILDLRNNPGGLVRAGLDVAQLWLDGDE-------------TLVYTID
Os06g21380 KTGYVRLAAFSQTAAAEMESAIKKMEDEGVQSYILDLRNNPGGLVKAGLDVAQMWLDGNE-------------TLVNTVD
At4g17740  KIGYIKLTTFNQNASSAVREAIETLRGNNVNAFVLDLRDNSGGSFPEGIEIAKFWLDKGV-------------IVYICDS
Os02g57060 KVGYIKLTTFNQNAAGSVKEALQKLRENNVKSFVLDLRNNSGGLFPEGIEIAKIWMDKGV-------------IVYICDS
At5g46390  SVGYIRLKEFNALARKDLVIAMKRLLDKGASYFVMDLRDNLGGLVQAGIETAKLFLDEGD-------------TVIYTAG
Os01g47450 AIGYIHIKEFNAVAKKDLVSALKRLQNSGASYFVLDLRDNLGGLVQAGIETAKLFLNKGDTLKVTVHGYYIKMKVIYTAG

           :                  ** *:**: :******:*.**:** :*:: *. *:*** ***:  * ***.: :*:.:* :
At3g57680  REGVTSPINMINGHAVTHDPLVVLVNEGSASASEILAGALHDNGRAILVGNRTFGKGKIQSITELNDGSALFVTVAKYLS
Os06g21380 REGNVLPINMARGHSLTHDPLVVLVNEGSASASEILAGALHDNGRAILVGHRTFGKGKIQSVTELDDGSALFITVAKYLS
At4g17740  RGVRDIYDTDGSNAIATSEPLAVLVNKGTASASEILAGALKDNKRALVYGEPTYGKGKIQSVFELSDGSGLAVTVARYET
Os02g57060 QGVRDIYEADGISTVAASEPLVVLVNKGTASASEILAGALKDNKRAVIYGEPTYGKGKIQSVFALSDGSGLAVTVARYET
At5g46390  RDPEAQKTVVSDKKPLITAPLIVMVNNRTASASEIVASALHDNCKAVLVGERTYGKGLIQSVYELRDGSGVVVTIGKYVT
Os01g47450 RDRQVQNTIVAEREPLVTTPLMVLVNNRTASASEIVASALHDNCKAVLVGEKTFGKGLIQSVFELHDGSGIVVTVGKYVT

           *   :*:  *: **
At3g57680  PSLHEIDQVGIAPDVQC
Os06g21380 PALHEIDQVGIQPDIQC
At4g17740  PAHTDIDKVGVTPDHPL
Os02g57060 PAHTDIDKVGVIPDRPL
At5g46390  PNHMDINGGGIEPDFRN
Os01g47450 PNHKDINGNGIEPDYRR
```
